# Supplementary material for: Cell Painting PLUS: expanding the multiplexing capacity of Cell Painting-based phenotypic profiling using iterative staining-elution cycles
Source: Nat Commun. 2025 Apr 24;16:3857. doi: 10.1038/s41467-025-58765-8 (PMC12022024; doi:10.1038/s41467-025-58765-8)
Supplement: Supplementary file 1 — Supplementary Information [file 41467_2025_58765_MOESM1_ESM.pdf]

SUPPLEMENTARY INFORMATION

Cell Painting PLUS: Expanding the multiplexing capacity of Cell Painting-based phenotypic profiling using iterative staining-elution cycles

Elena von Coburg[1,2], Marlene Wedler[1,3], Jose M. Muino[1, 4], Christopher Wolff[5], Nils Körber[6], Sebastian Dunst[1*], Shu Liu[1*]

[1] *German Centre for the Protection of Laboratory Animals (Bf3R), German Federal Institute for Risk Assessment (BfR), Berlin, Germany*

[2] *Department of Food Chemistry, University of Potsdam, Potsdam, Germany*

[3] *Institute of Biology, Free University of Berlin, Berlin, Germany*

[4] *Institute of Clinical Pharmacology and Toxicology, Charité – Universitätsmedizin Berlin, corporate member of Freie Universität Berlin, Humboldt-Universität zu Berlin, and Berlin Institute of Health, Berlin, Germany*

[5] *Screening Unit, Leibniz-Forschungsinstitut für Molekulare Pharmakologie (FMP), Berlin, Germany*

[6] *Centre for Artificial Intelligence in Public Health Research, Robert Koch Institute, Berlin, Germany*

* Equal contribution, correspondence: sebastian.dunst@bfr.bund.de, shu.liu@bfr.bund.de

TABLE OF CONTENTS

Supplementary figures and figure legends

References

SUPPLEMENTARY FIGURES AND FIGURE LEGENDS

Supplementary Fig. 1. CPP optimization and evaluation.

(A) Evaluation of spectral crosstalk (emission bleed-through and cross excitation) and signal stability for selection of optimal CPP dyes. Spectral crosstalk: numbers and heatmaps show relative signal intensities (mean of all wells) normalized to unstained control channels (signal-to-noise ratio); N_{Tech} = 3 (with 5 fields/well); N_{Biol} = 1. Signal stability: box-and-whiskers plots show relative signal intensities (median of all wells) normalized to day 0; boxes: 25th to 75th percentiles with median and min to max whiskers; light grey area: baseline intensity (day 0) ± 10% deviation; N_{Tech} = 14 (with 7 fields/well); N_{Biol} = 1.

(B) Evaluation of exposure times and signal intensities for optimization of CPP dye concentrations (benchmark: signal-to-noise ratio of at least 5). Numbers (mean of all wells) and heatmaps show relative signal intensities (median of all wells) normalized to unstained control channels (signal-to-noise ratio); N_{Tech} = 3 (with 5 fields/well); N_{Biol} = 1. Working concentrations used in the CPP assay are shown in comparison to the original and updated CP method.[1,2]

(C) Histograms from single images of three published antibody elution buffers used for elution of CPP dyes in MCF-7 cells.

(D) Representative images comparing staining of MCF-7 cells with CPP Actin dye to other Actin dyes or an Actin antibody with or without prior elution step. N_{Biol} = 1. Scale bars = 20 µm.

(E) Enlarged regions of representative images shown in Fig. 1A-B comparing Mito staining of nuclear regions between CP and CPP in MCF-7 cells. N_{Biol} = 4. Scale bars = 10 µm.

(F) Evaluation of the influence of the number of cells and imaging fields at 20x magnification on the variance of feature data in CP and CPP. Plots show the median coefficient of variation (CV) across wells and plates of unprocessed, Harmony-extracted CP or CPP features depending on the number of fields and number of randomly chosen cells.

Source data are provided as a Source Data file.

Supplementary Figure 1

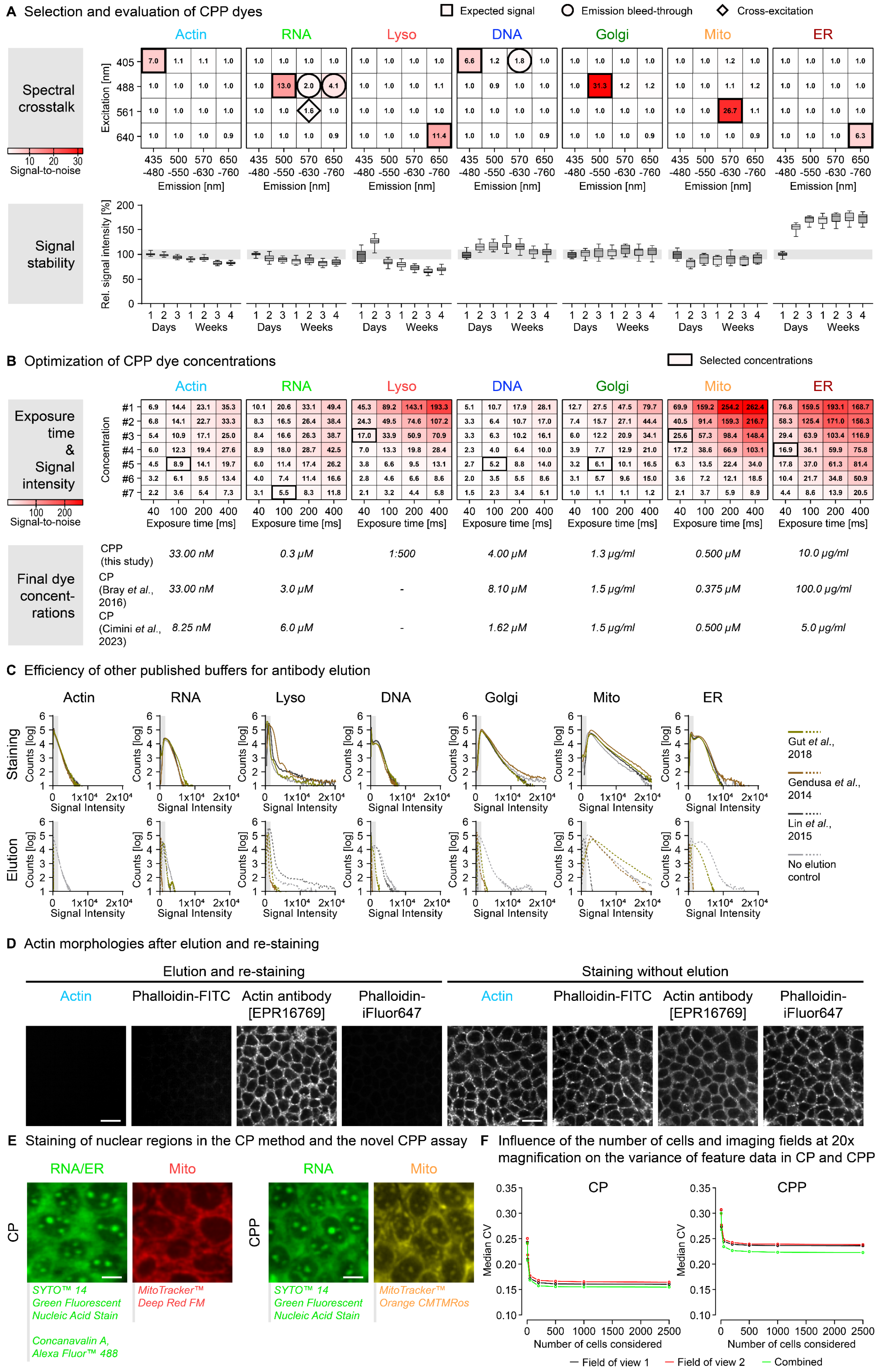

Supplementary Fig. 2. CPP image analysis workflow, compound activity profiles for CPP in HepG2, U2OS, or RPTEC cells, and comparison of selected CPP and CP channels in MCF7 cells.

(A) Representative images from MCF-7 cells illustrating the image analysis workflow, which includes the subsequent imaging of cycle 1 and cycle 2 dyes, combination of cycle 1 and cycle 2 images into a single multi-channel stack based on the Mito channel, and finally identification of different cell regions for feature extraction as described in Fig. 1E. During image registration, combination of cycle 1 and cycle 2 images may lead to some cropping of the original images (highlighted in red) due to imaging position shifts. N_{Biol} = 4. Scale bars = 20 µm.

(B) Compound activity profiles showing activities of all reference compounds at eight tested concentrations in HepG2, U2OS, and RPTEC cells as described in Fig. 2C. Heatmaps visualize calculated robust z-scores (median of all N_{Tech} = 3 and N_{Biol} = 4) for each feature (feature-level) extracted from CPP images captured at 20x magnification. High-resolution versions of heatmaps are included in the Supplementary Data 16-18.

(C) Direct comparison of selected imaging channels from the compound activity profiles for CPP and CP shown in Fig 2C. Scatter plots comparing robust z-scores of all features from the Generic, DNA, and Mito channels that are common in CPP and CP (left plot), or only the cytoplasm features from the separated RNA and ER channels in CPP with the merged RNA/ER channel in CP (right plot). The x-axis corresponds to the robust z-scores for CP and the y-axis the corresponding values for CPP. Pearson correlation is performed using the robust z-score (median of all N_{Tech} = 3 and N_{Biol} = 4) of each feature (feature-level) extracted from images captured at 20x magnification.

Source data are provided as a Source Data file.

Supplementary Figure 2

A Cell Painting PLUS image analysis workflow

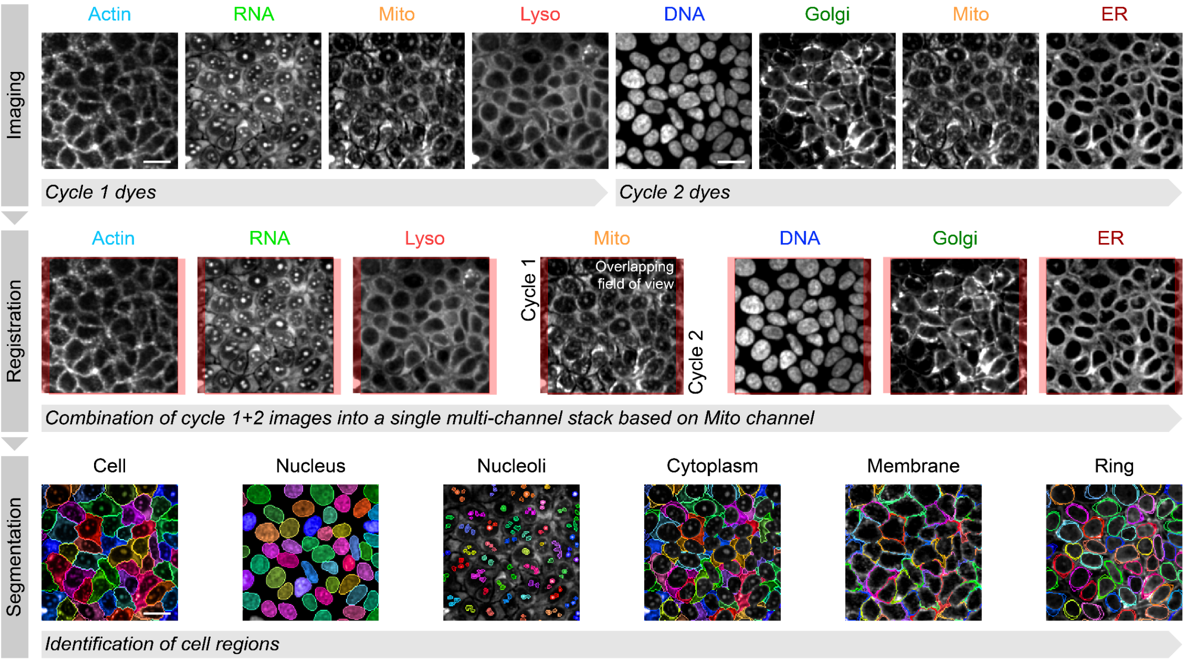

B Compound activity profiles (robust z-scores, feature-level) of all reference compounds at eight tested concentrations

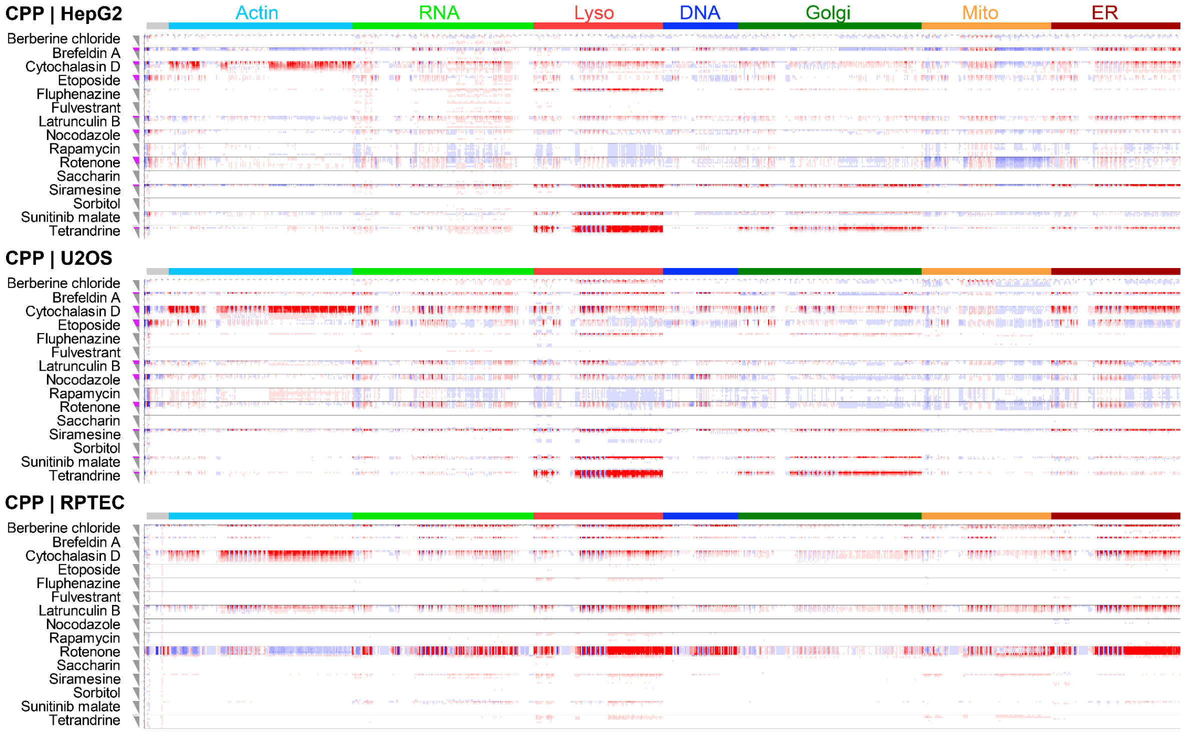

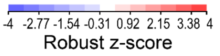

C Comparison of CPP and CP compound activity profiles (robust z-scores, feature-level) of all reference compounds tested at eight concentrations in MCF-7 cells

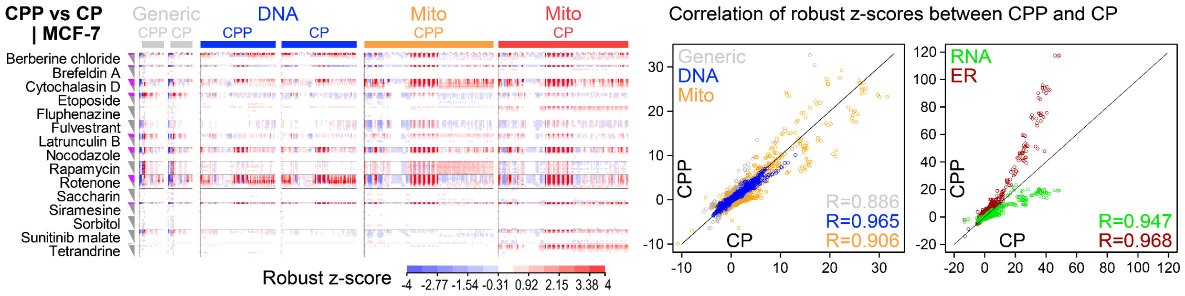

Supplementary Fig. 3. Reproducibility and Proportion BMC profiles for CPP in HepG2, U2OS, and RPTEC cells.

(A-B) Pearson correlation matrices for HepG2, U2OS, and RPTEC cells comparing intra-plate/technical and inter-plate/biological variability of (A) robust z-score data and (B) BMC data for CPP images taken at 20x magnification as described in Fig. 2F and Fig. 3C. *AllRep*: median of all $N_{Tech} = 3$ and $N_{Biol} = 4$; *BRep*: median of all $N_{Tech} = 3$ for each biological replicate; *TRep*: Median of all $N_{Biol} = 4$ for each technical replicate.

(C) Proportion BMC profiles showing relative activities of all reference compounds across four different cell lines using the CPP method with images captured at 20x magnification as described in Fig. 3B and Fig. 4B. For example, the feature category *Mito* includes all Mito-related features, whereas the feature categories *Mito Cytoplasm* or *Mito Texture* include the subset of those Mito-related features that are additionally related to specific cell regions or analysis modules.

Source data are provided as a Source Data file.

Supplementary Figure 3

A Reproducibility of robust z-scores in different cell lines

B Reproducibility of BMCs in different cell lines

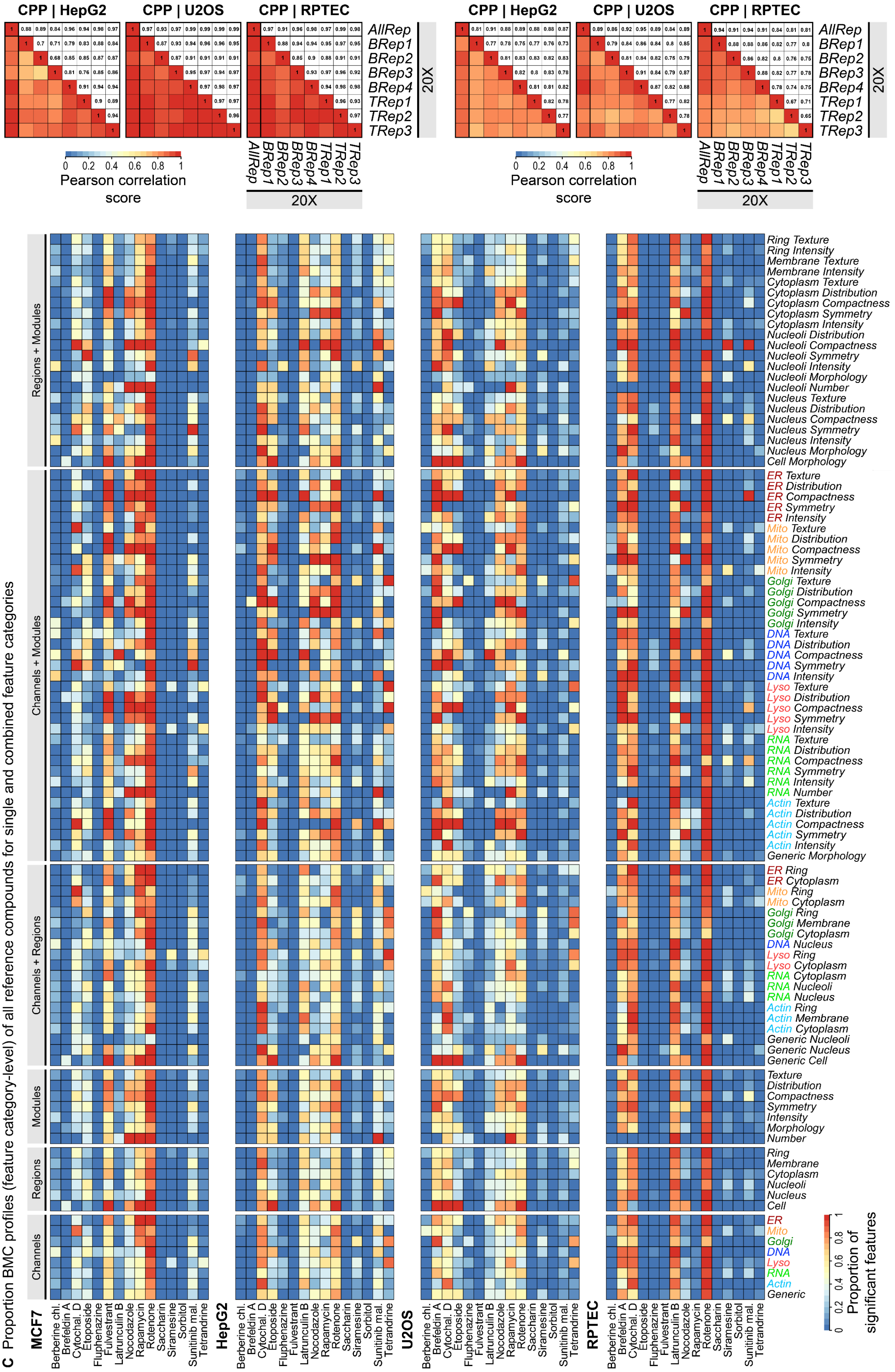

Supplementary Fig. 4. BMC accumulation and magnitude plots for CPP across all cell lines.

(A-O) BMC accumulation and magnitude plots showing the concentration-dependent sequence (rank) and maximum effect size (normalized magnitude, i.e., the maximum robust z-score) of all reference compounds on feature categories and single features (extracted from CPP images [using Harmony software for image analysis] captured at 20x magnification and) across all cell lines as described in Fig. 3D.

Source data are provided as a Source Data file.

Supplementary Figure 4A

BMC accumulation and magnitude plots (feature category-level)

Substance:	Staining method:	Image analysis software:
**Berberine chloride**	**Cell Painting Plus**	**Harmony**

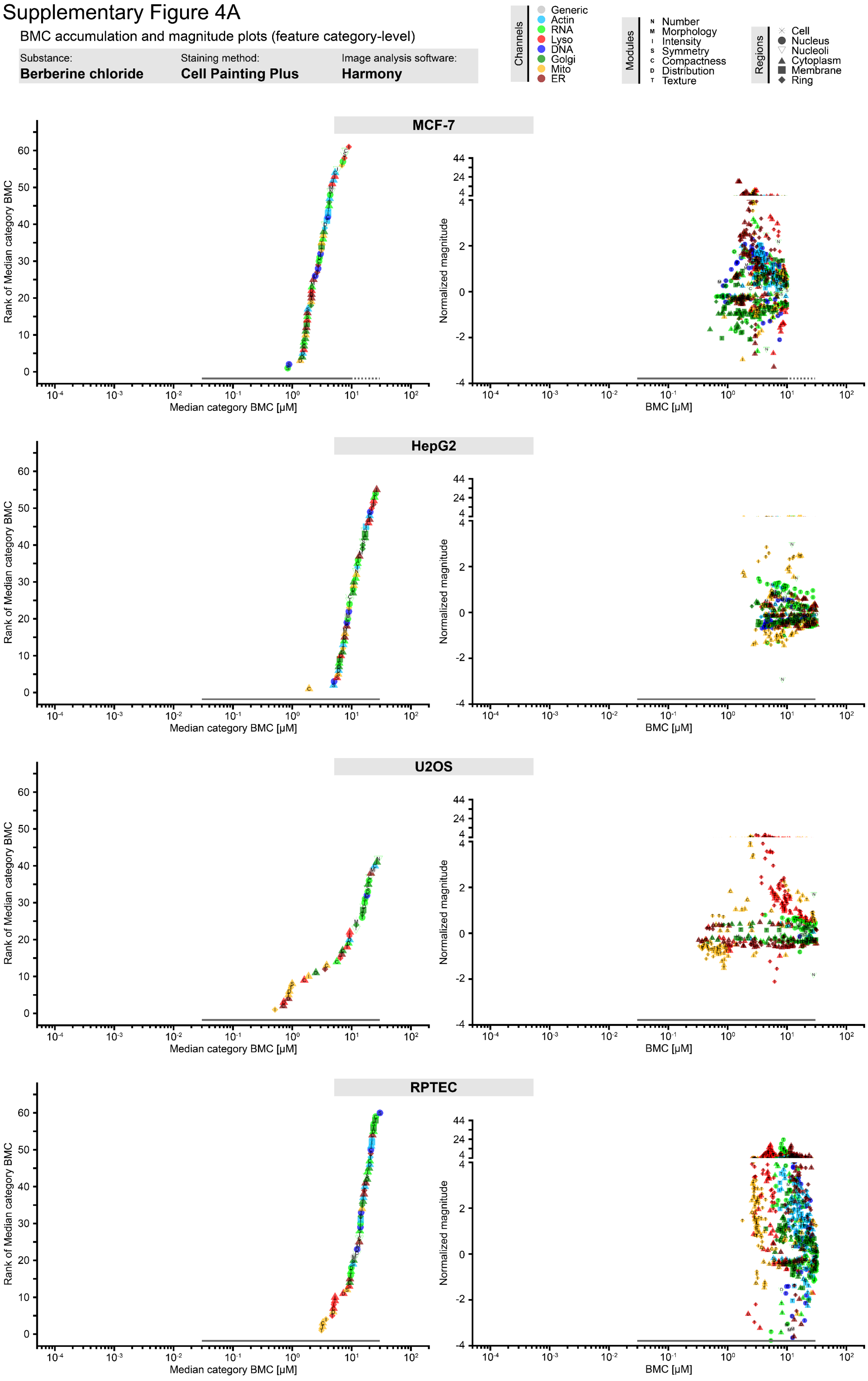

Supplementary Figure 4B

BMC accumulation and magnitude plots (feature category-level)

Substance:	Staining method:	Image analysis software:
**Brefeldin A**	**Cell Painting Plus**	**Harmony**

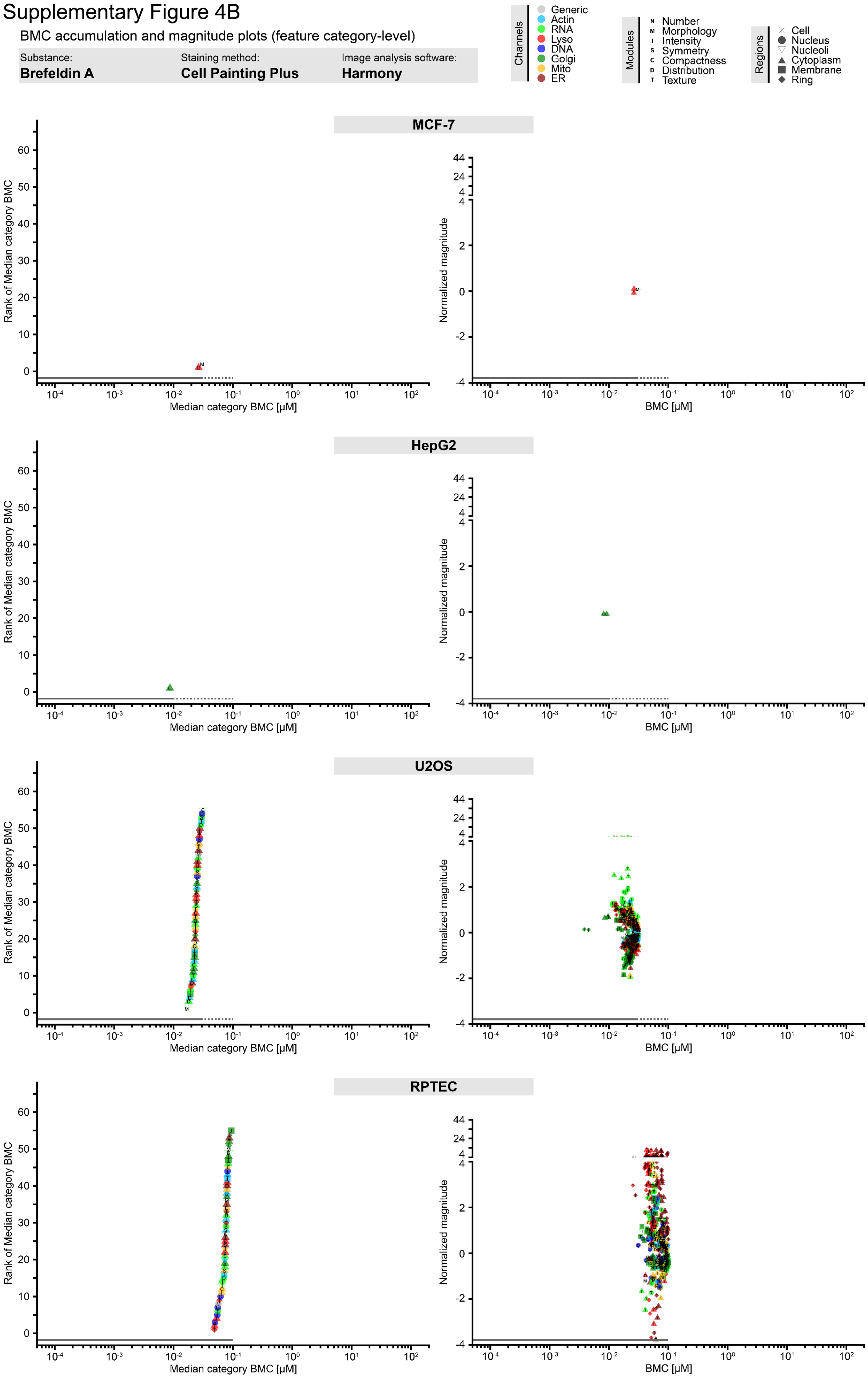

Supplementary Figure 4C

BMC accumulation and magnitude plots (feature category-level)

Substance:	Staining method:	Image analysis software:
**Cytochalasin D**	**Cell Painting Plus**	**Harmony**

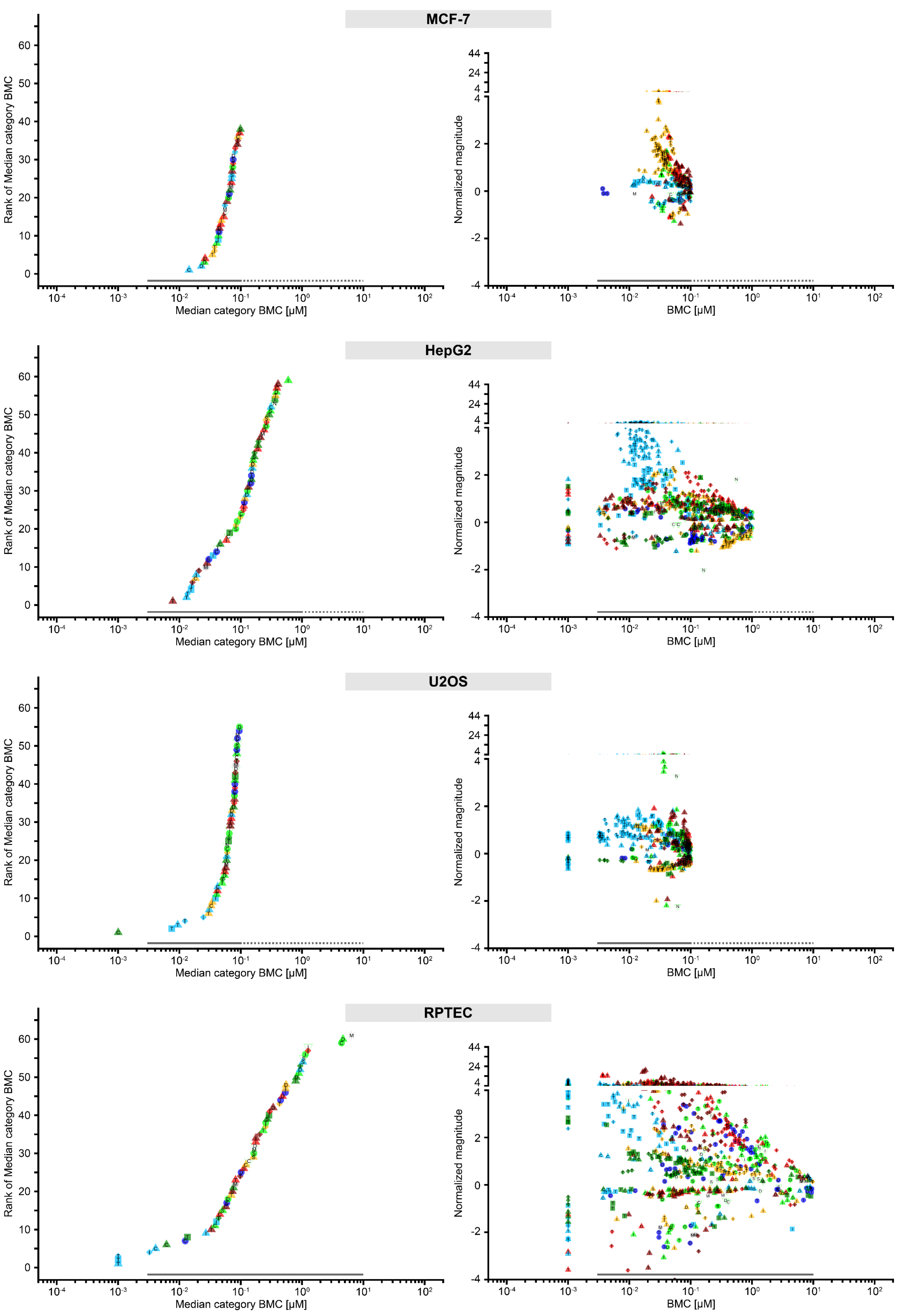

Supplementary Figure 4D

BMC accumulation and magnitude plots (feature category-level)

Substance:	Staining method:	Image analysis software:
**Etoposide**	**Cell Painting Plus**	**Harmony**

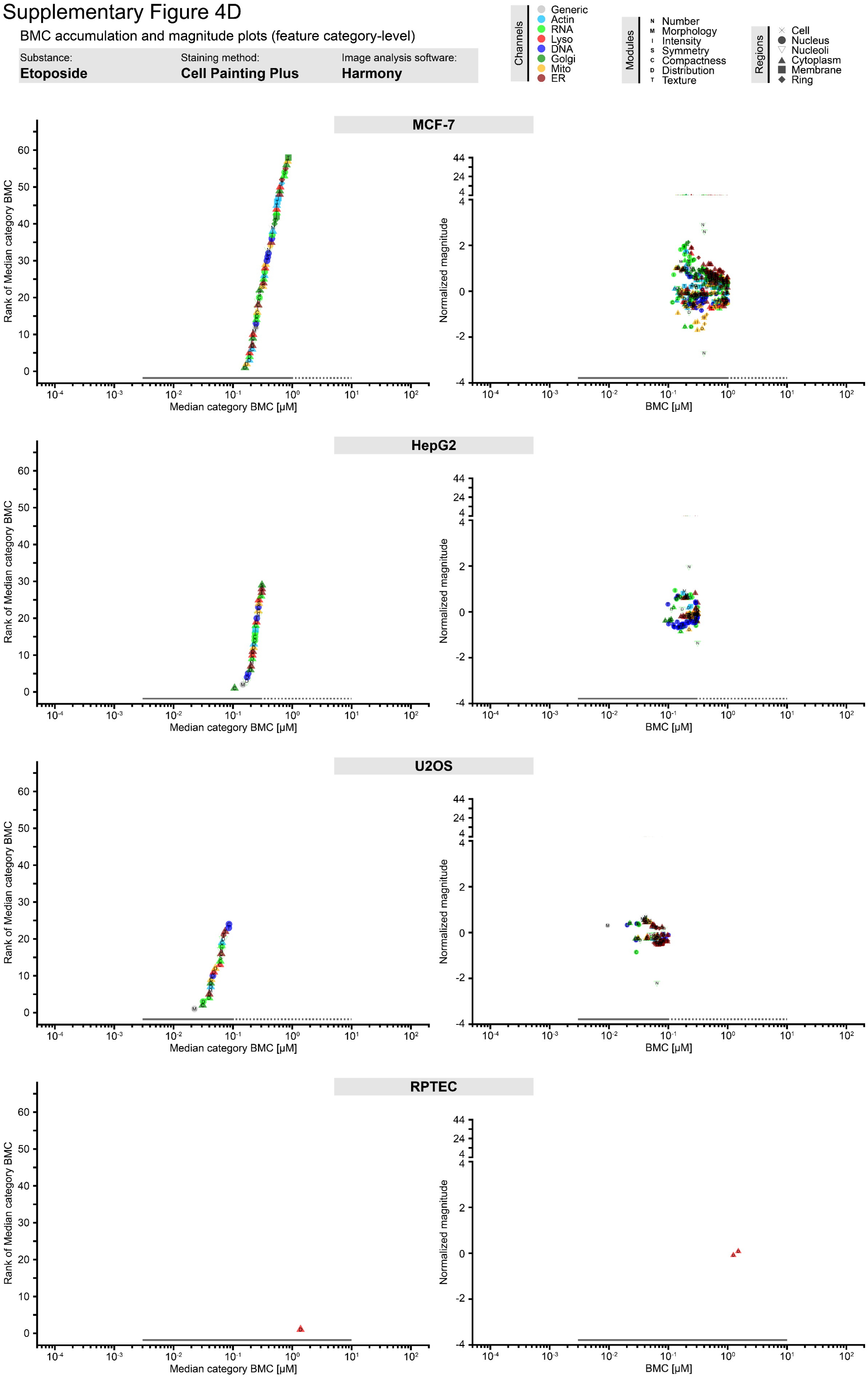

Supplementary Figure 4E

BMC accumulation and magnitude plots (feature category-level)

Substance:	Staining method:	Image analysis software:
**Fluphenazine**	**Cell Painting Plus**	**Harmony**

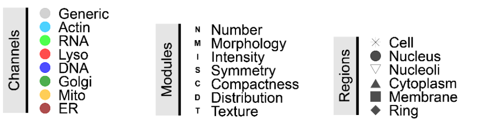

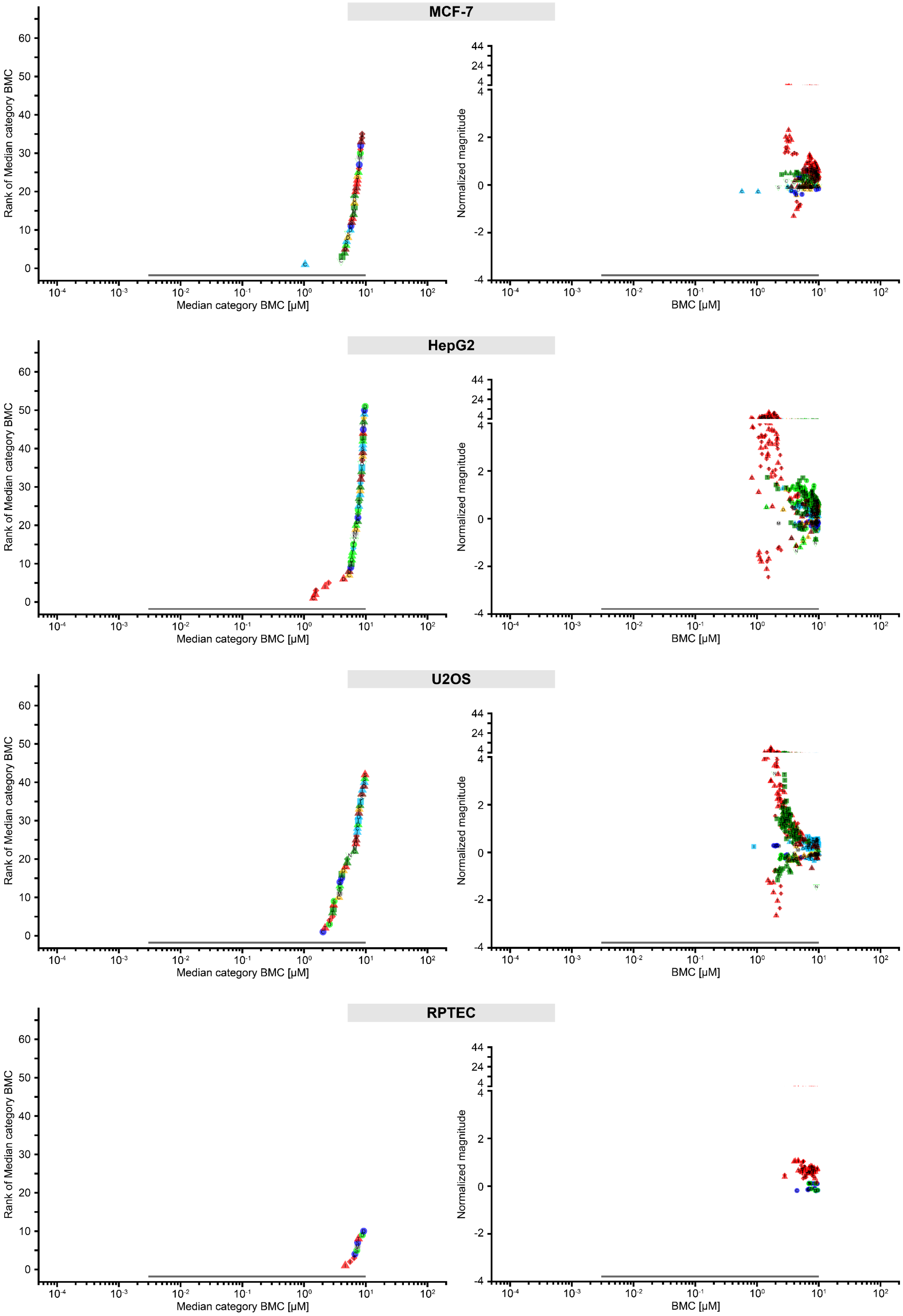

Supplementary Figure 4F

BMC accumulation and magnitude plots (feature category-level)

Substance:	Staining method:	Image analysis software:
**Fulvestrant**	**Cell Painting Plus**	**Harmony**

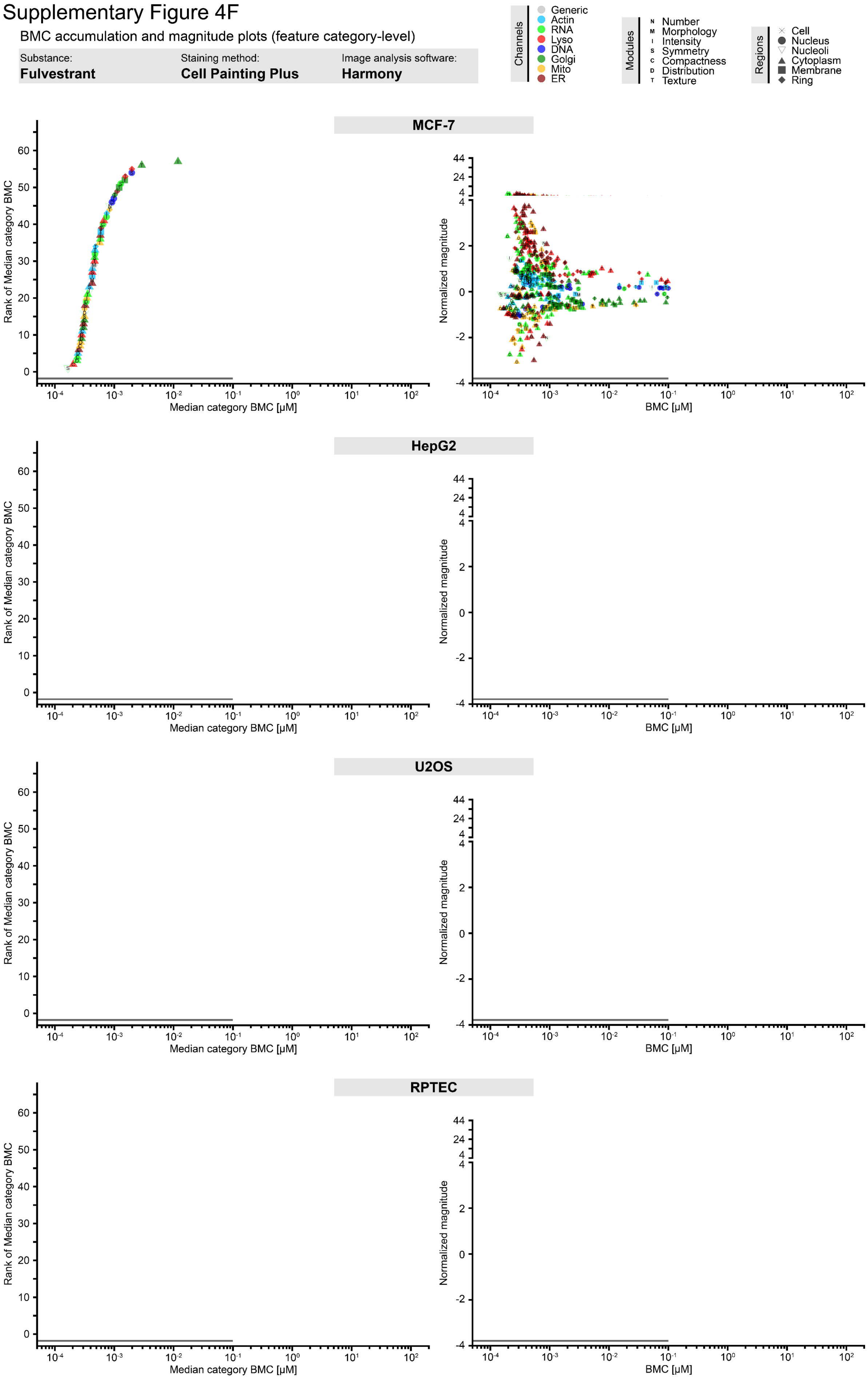

Supplementary Figure 4G

BMC accumulation and magnitude plots (feature category-level)

Substance:	Staining method:	Image analysis software:
**Latrunculin B**	**Cell Painting Plus**	**Harmony**

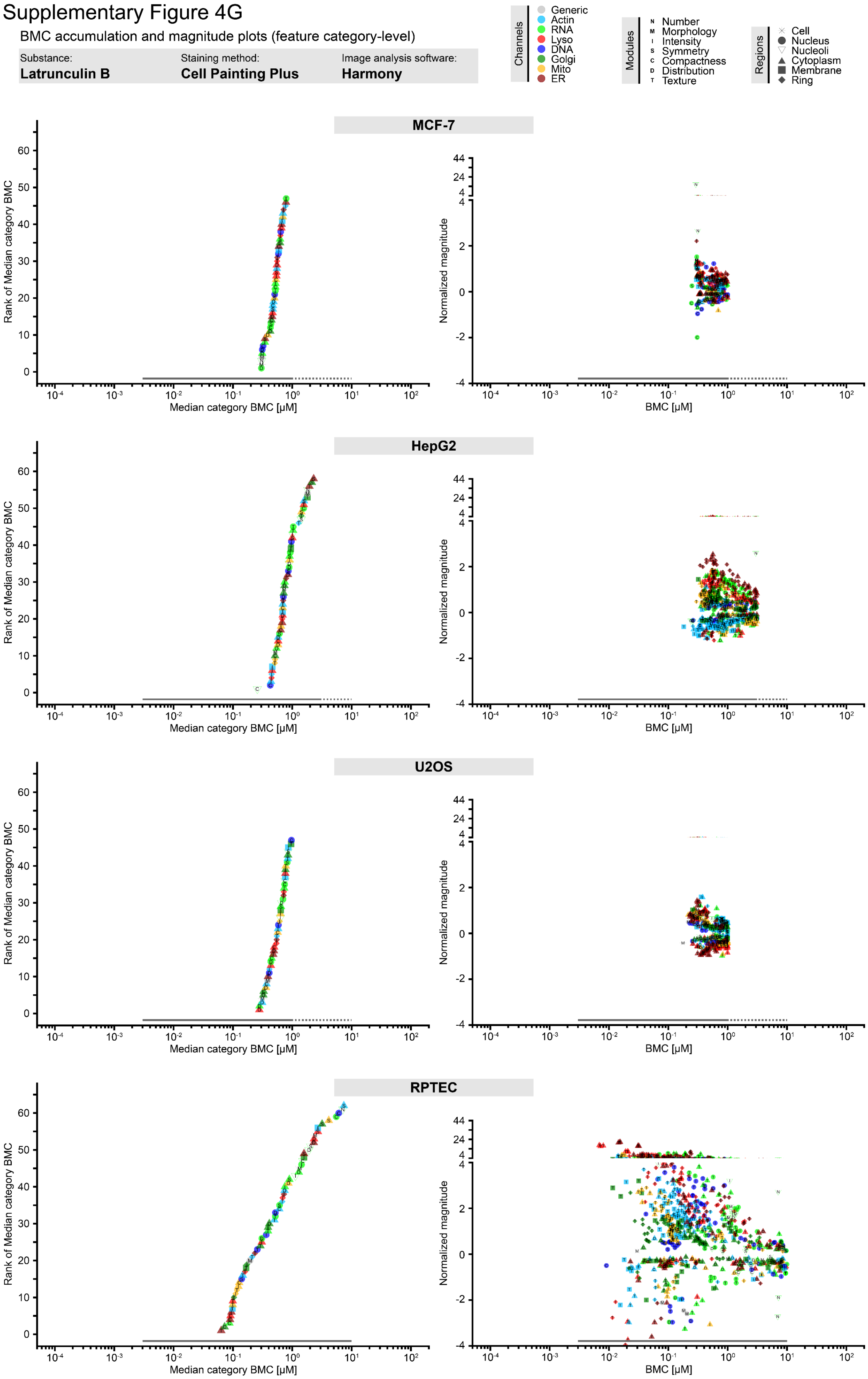

Supplementary Figure 4H

BMC accumulation and magnitude plots (feature category-level)

Substance:	Staining method:	Image analysis software:
**Nocodazole**	**Cell Painting Plus**	**Harmony**

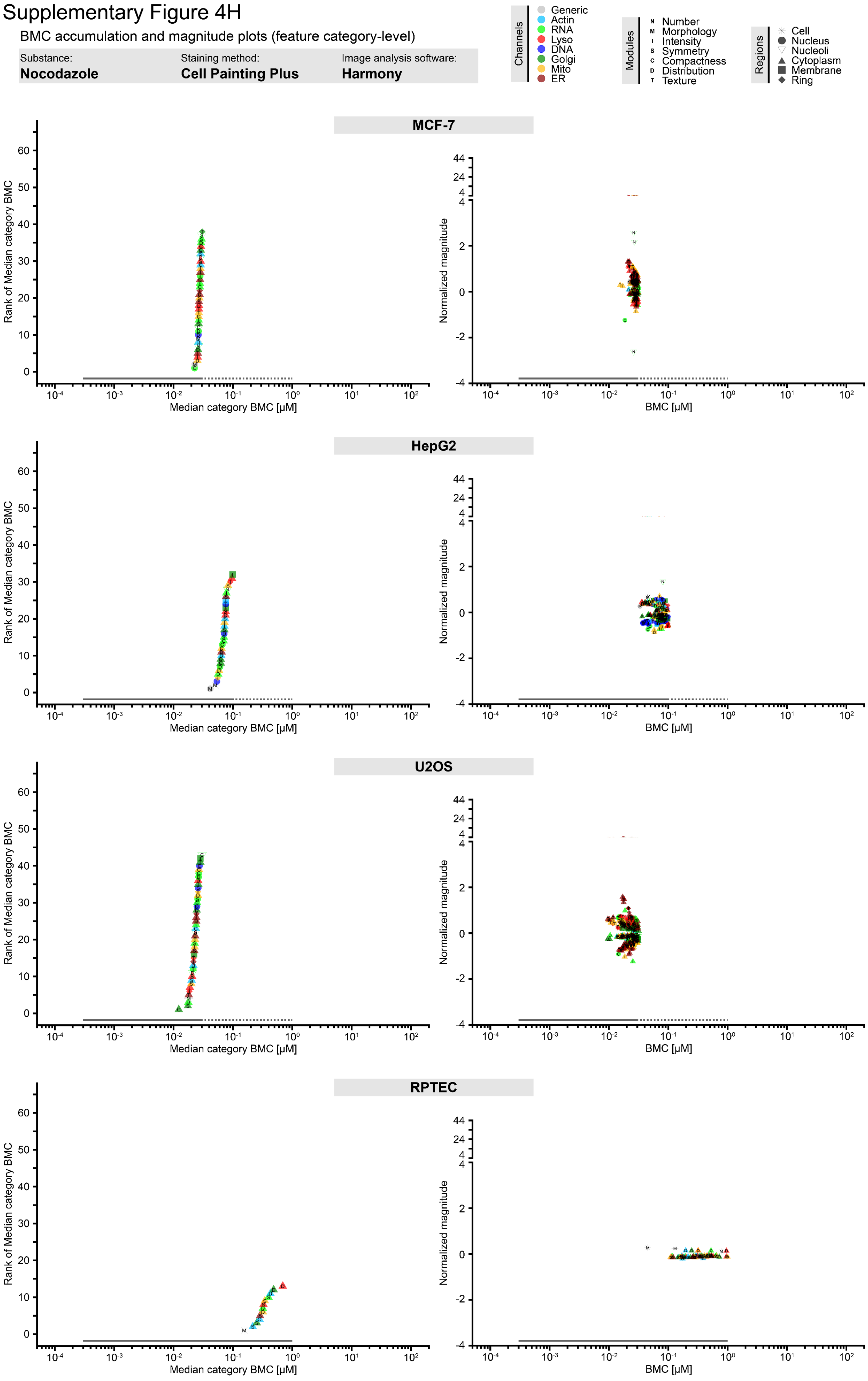

Supplementary Figure 4I

BMC accumulation and magnitude plots (feature category-level)

Substance:	Staining method:	Image analysis software:
**Rapamycin**	**Cell Painting Plus**	**Harmony**

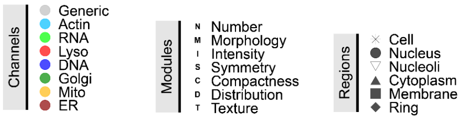

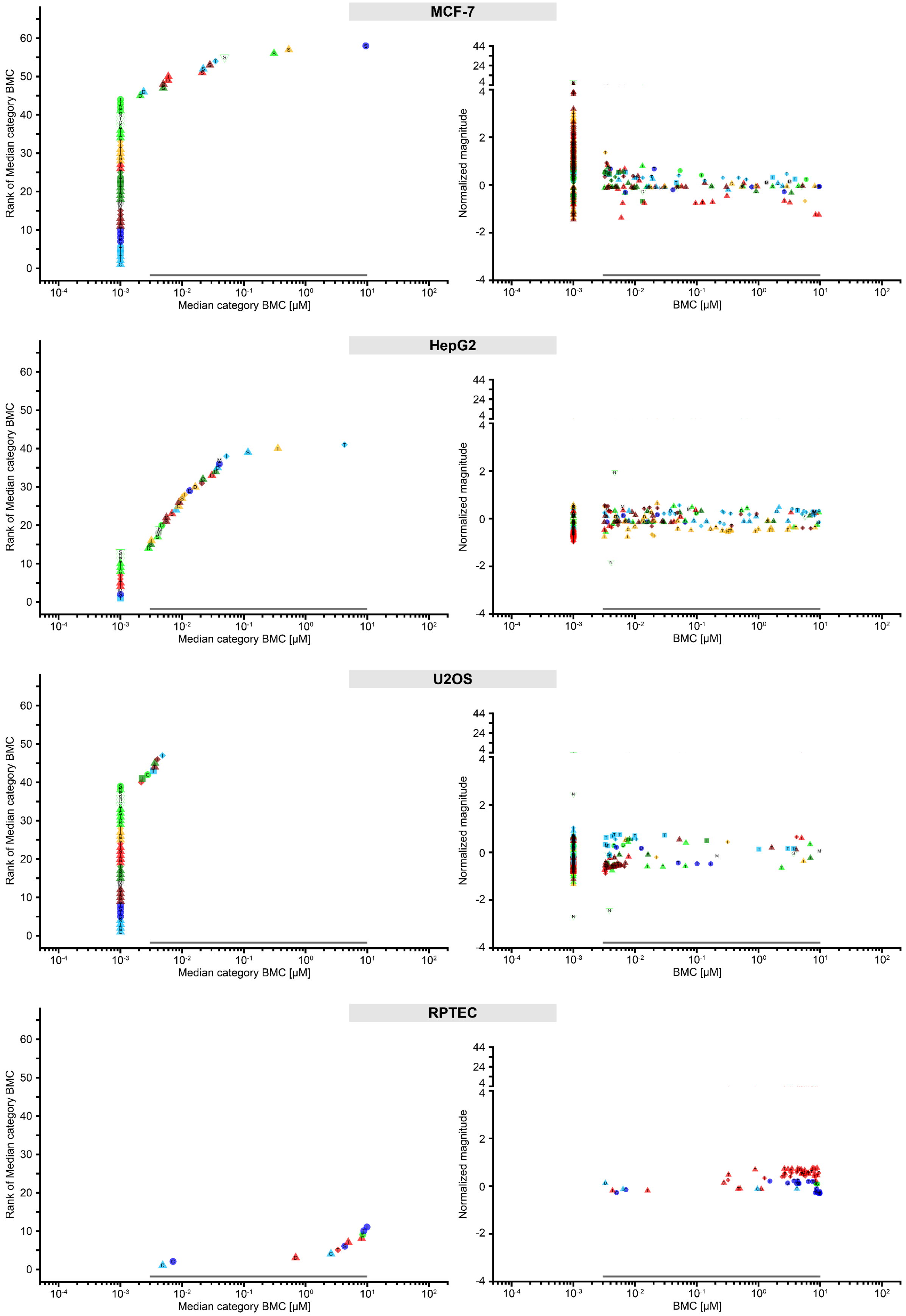

Supplementary Figure 4J

BMC accumulation and magnitude plots (feature category-level)

Substance:	Staining method:	Image analysis software:
**Rotenone**	**Cell Painting Plus**	**Harmony**

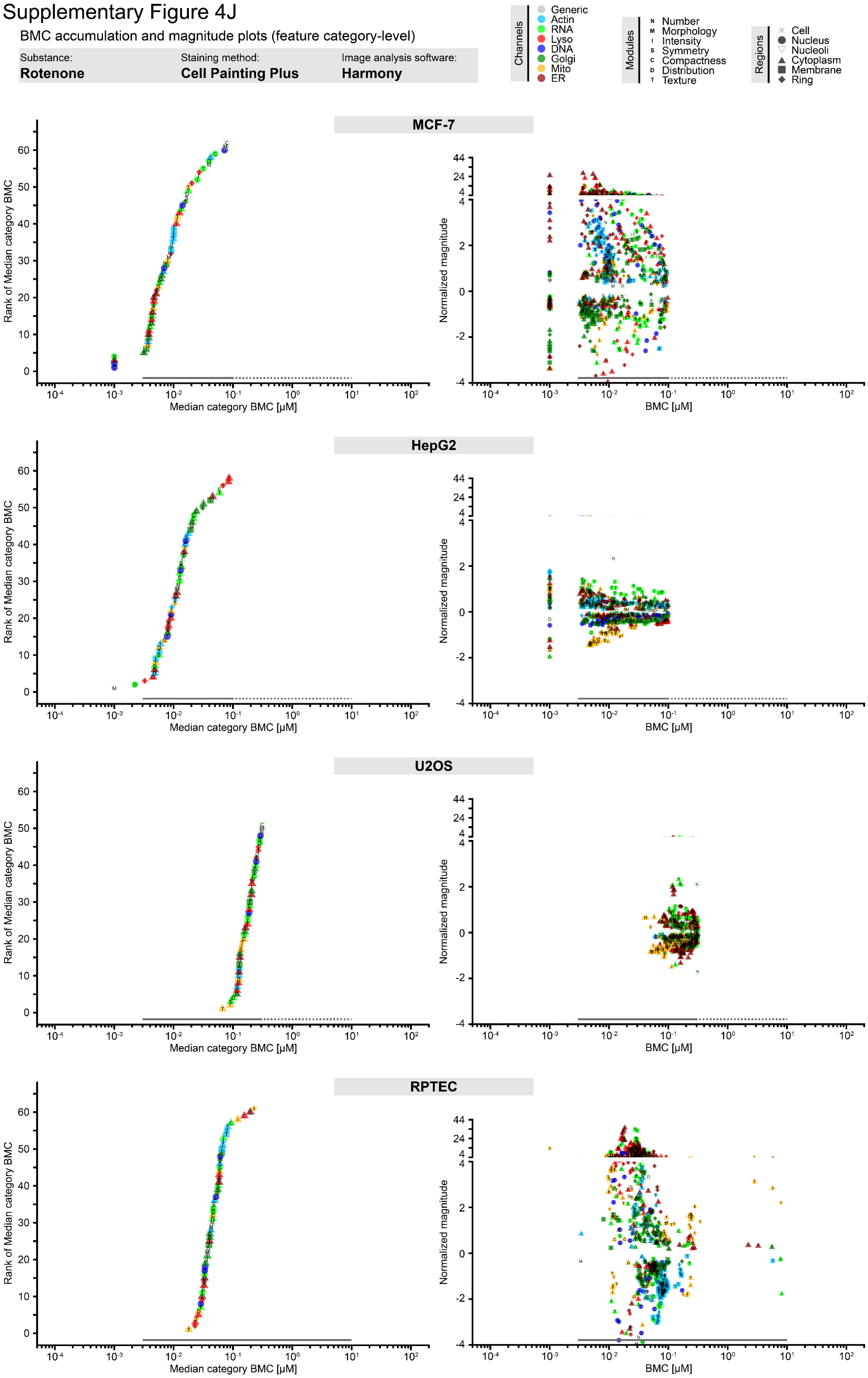

Supplementary Figure 4K

BMC accumulation and magnitude plots (feature category-level)

Substance:	Staining method:	Image analysis software:
**Saccharin**	**Cell Painting Plus**	**Harmony**

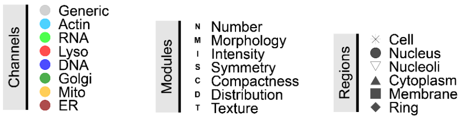

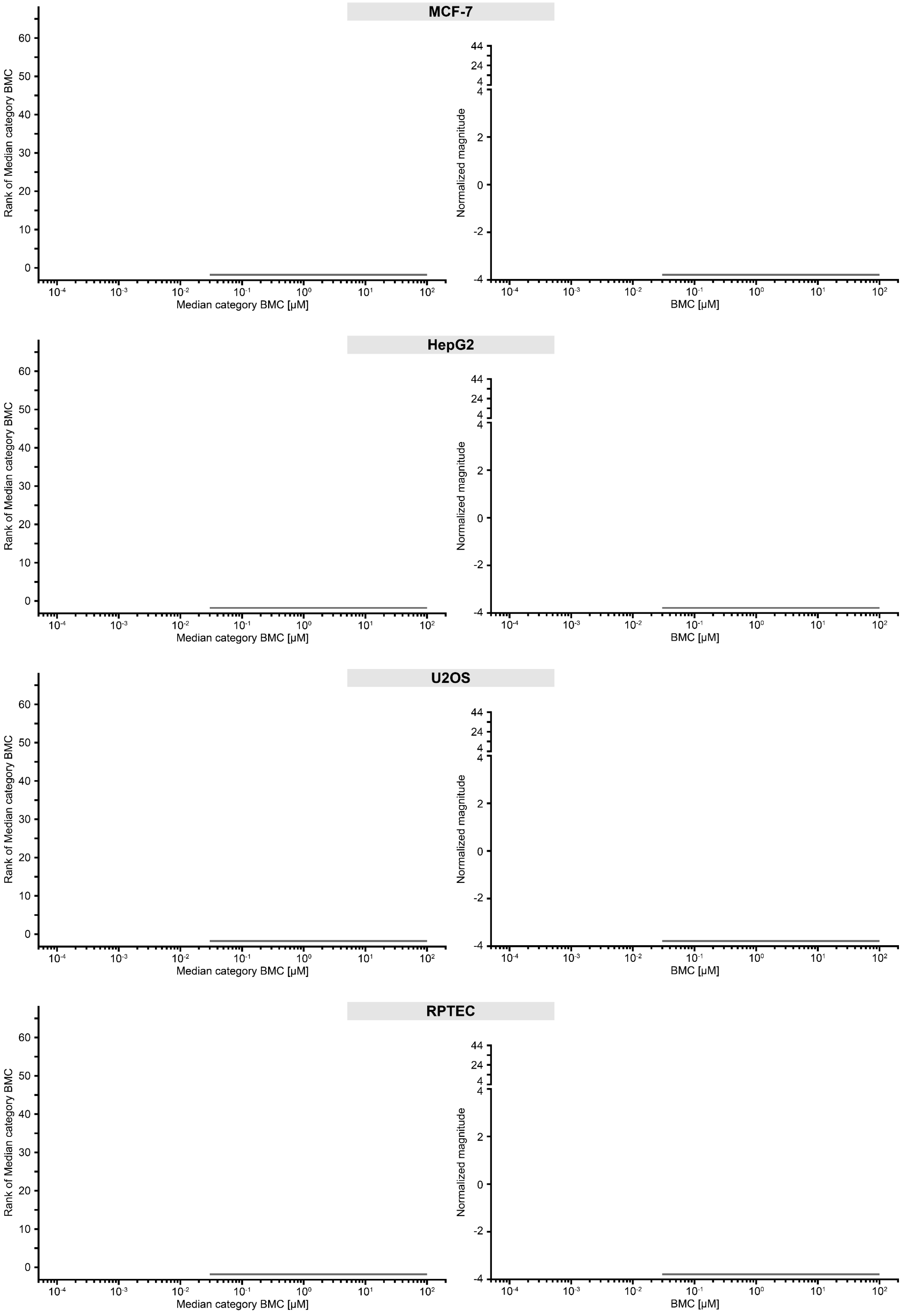

Supplementary Figure 4L

BMC accumulation and magnitude plots (feature category-level)

Substance:	Staining method:	Image analysis software:
**Siramesine**	**Cell Painting Plus**	**Harmony**

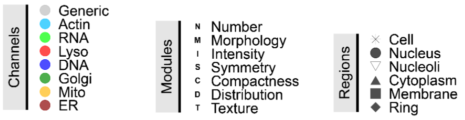

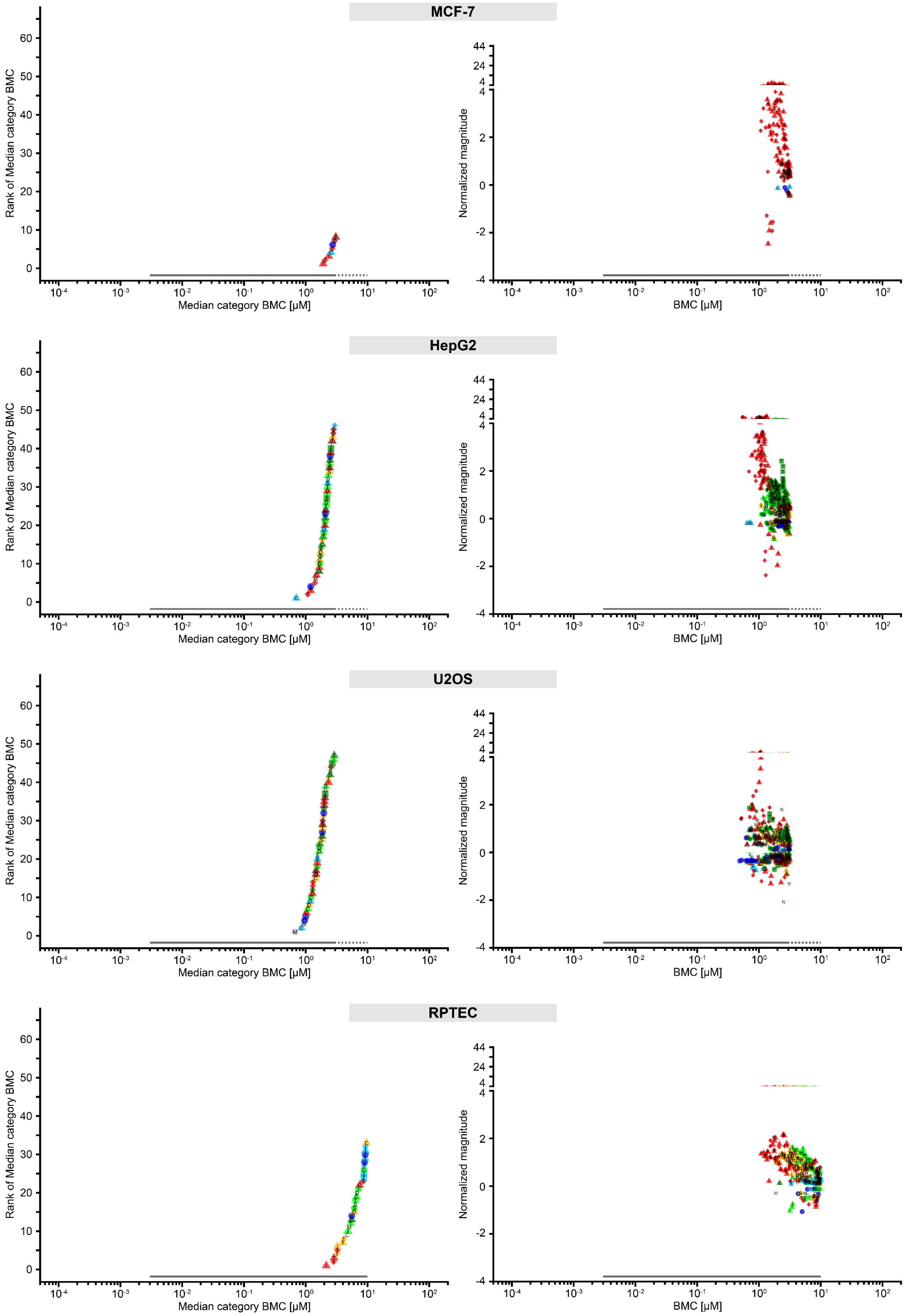

Supplementary Figure 4M

BMC accumulation and magnitude plots (feature category-level)

Substance:	Staining method:	Image analysis software:
**Sorbitol**	**Cell Painting Plus**	**Harmony**

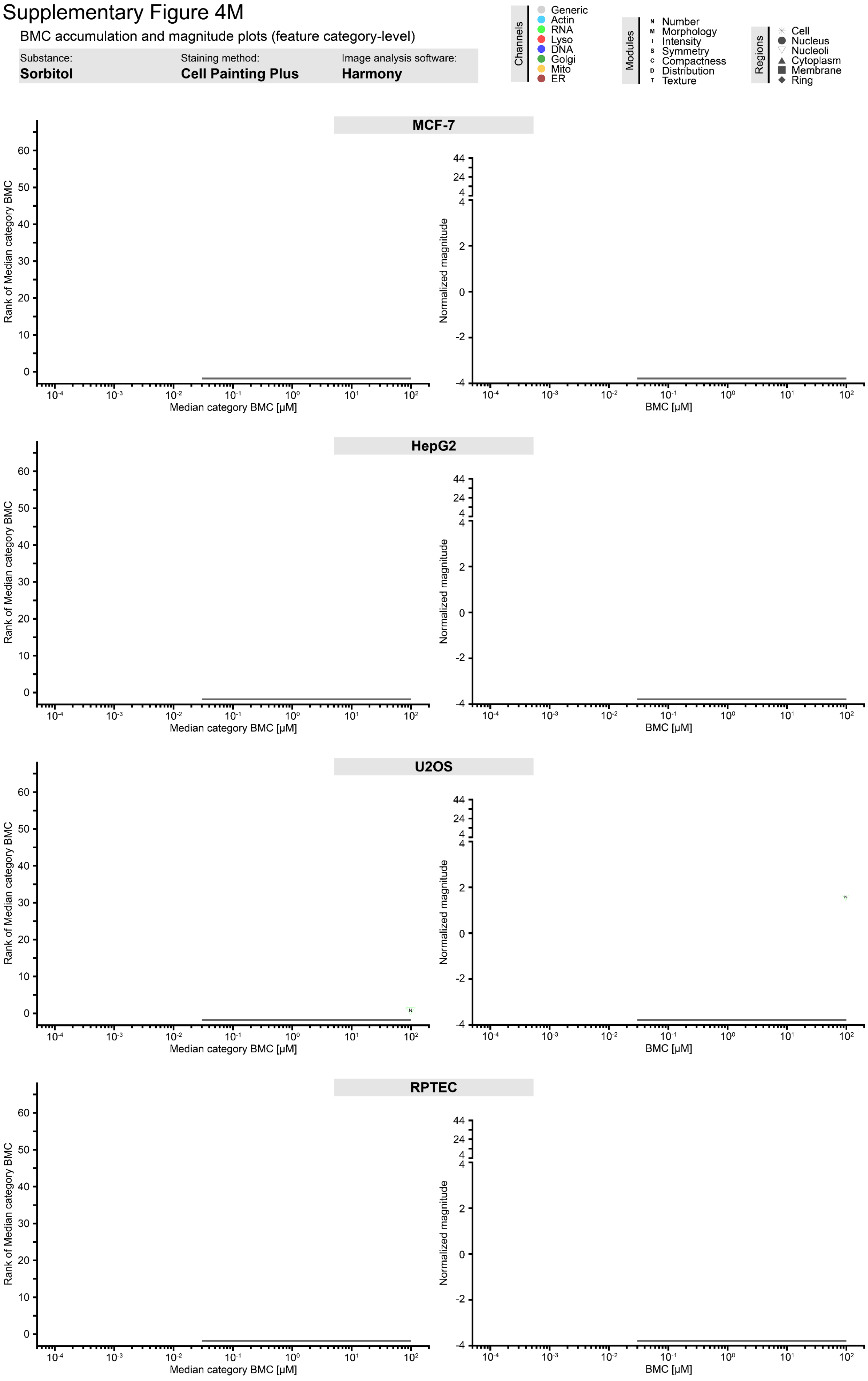

Supplementary Figure 4N

BMC accumulation and magnitude plots (feature category-level)

Substance:	Staining method:	Image analysis software:
**Sunitinib malate**	**Cell Painting Plus**	**Harmony**

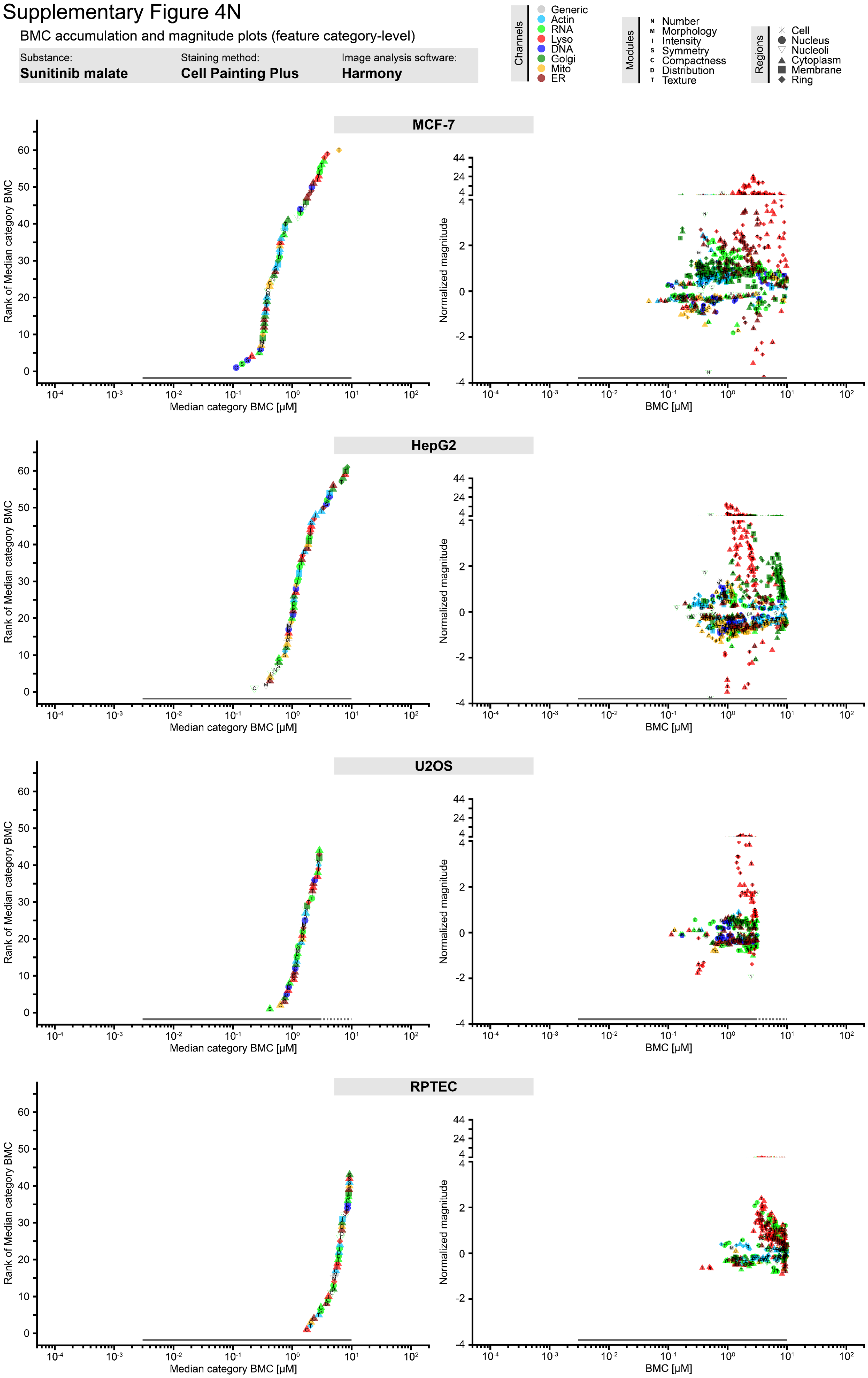

Supplementary Figure 4O

BMC accumulation and magnitude plots (feature category-level)

Substance:	Staining method:	Image analysis software:
**Tetrandrine**	**Cell Painting Plus**	**Harmony**

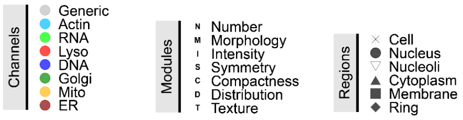

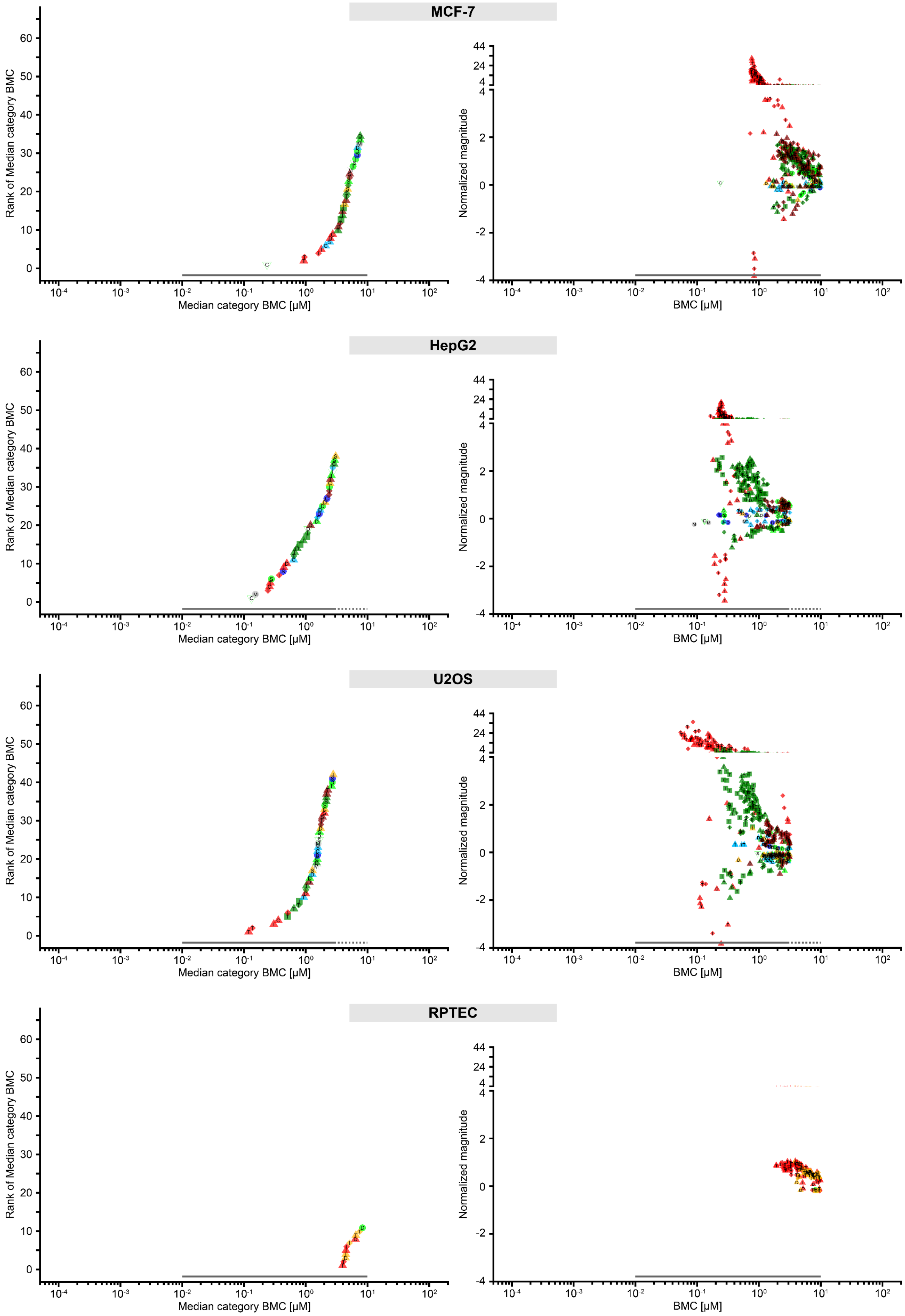

Supplementary Fig. 5. BMC accumulation and magnitude plots for CP in MCF-7 cells and CPP (using Harmony or Cell Profiler software for image analysis) in MCF-7 cells.

(A-O) BMC accumulation and magnitude plots showing the concentration-dependent sequence (rank) and maximum effect size (normalized magnitude, i.e., the maximum robust z-score) of all reference compounds on feature categories and single features (extracted from CP images [using Harmony software for image analysis] or CPP images [using Harmony or Cell Profiler software for image analysis] captured at 20x magnification) in MCF-7 cells as described in Fig. 3D.

Source data are provided as a Source Data file.

Supplementary Figure 5A

BMC accumulation and magnitude plots (feature category-level)

Substance:	Staining method:	Image analysis software:
**Berberine chloride**	**Cell Painting**	**Harmony**

Channels: Generic, DNA, RNA/ER, AGP, Mito
Modules: N Number, M Morphology, I Intensity, S Symmetry, C Compactness, D Distribution, T Texture
Regions: Cell, Nucleus, Nucleoli, Cytoplasm, Membrane, Ring

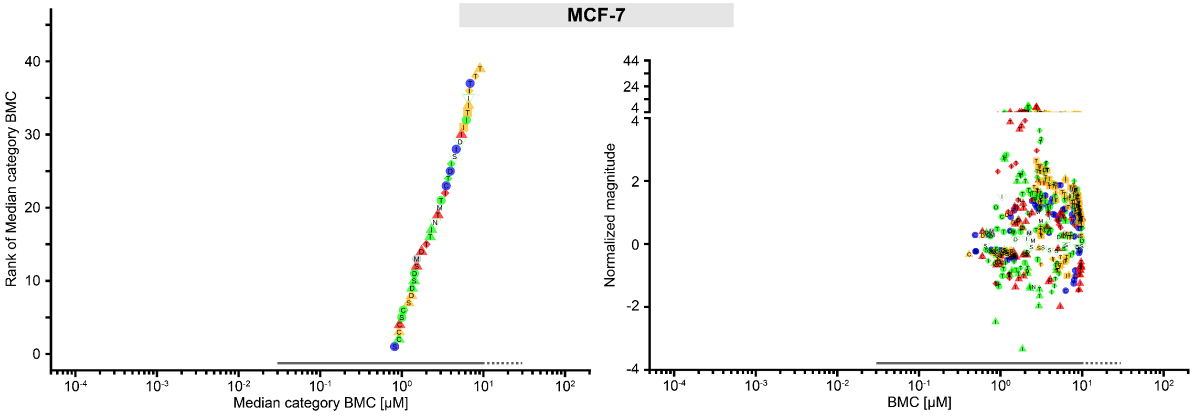

BMC accumulation and magnitude plots (feature category-level)

Substance:	Staining method:	Image analysis software:
**Berberine chloride**	**Cell Painting Plus**	**Cell Profiler**

Channels: Generic, Actin, RNA, Lyso, DNA, Golgi, Mito, ER
Modules: N Number, M Morphology, I Intensity, G Granularity, D Distribution, T Texture
Regions: Cell, Nucleus, Nucleoli, Cytoplasm, Membrane, Ring

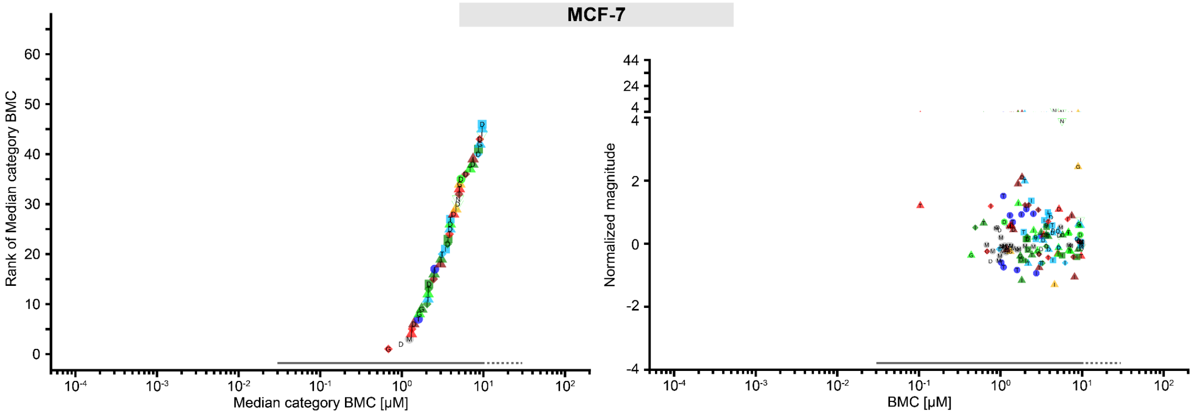

For comparison (from Supplementary Figure 4A):

BMC accumulation and magnitude plots (feature category-level)

Substance:	Staining method:	Image analysis software:
**Berberine chloride**	**Cell Painting Plus**	**Harmony**

Channels: Generic, Actin, RNA, Lyso, DNA, Golgi, Mito, ER
Modules: N Number, M Morphology, I Intensity, S Symmetry, C Compactness, D Distribution, T Texture
Regions: Cell, Nucleus, Nucleoli, Cytoplasm, Membrane, Ring

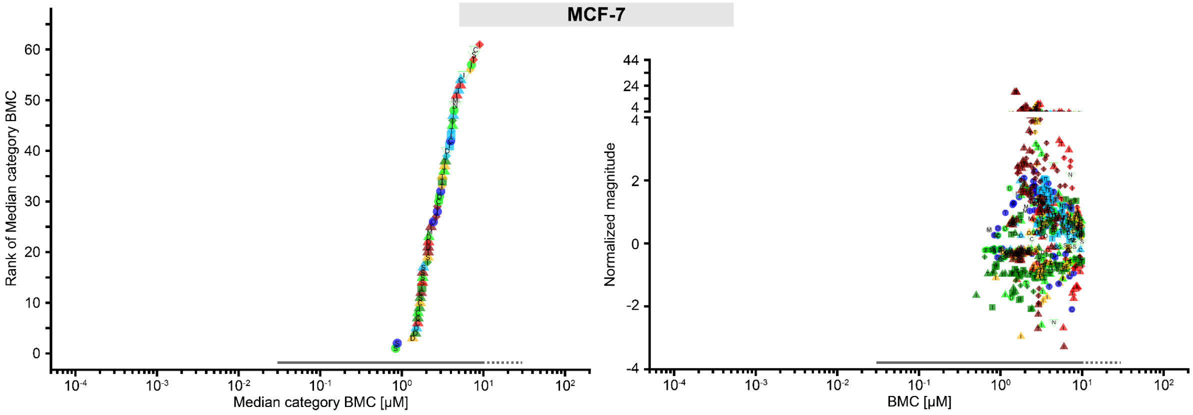

Supplementary Figure 5B

BMC accumulation and magnitude plots (feature category-level)

Substance:	Staining method:	Image analysis software:
**Brefeldin A**	**Cell Painting**	**Harmony**

Channels: Generic, DNA, RNA/ER, AGP, Mito

Modules: N Number, M Morphology, I Intensity, S Symmetry, C Compactness, D Distribution, T Texture

Regions: Cell, Nucleus, Nucleoli, Cytoplasm, Membrane, Ring

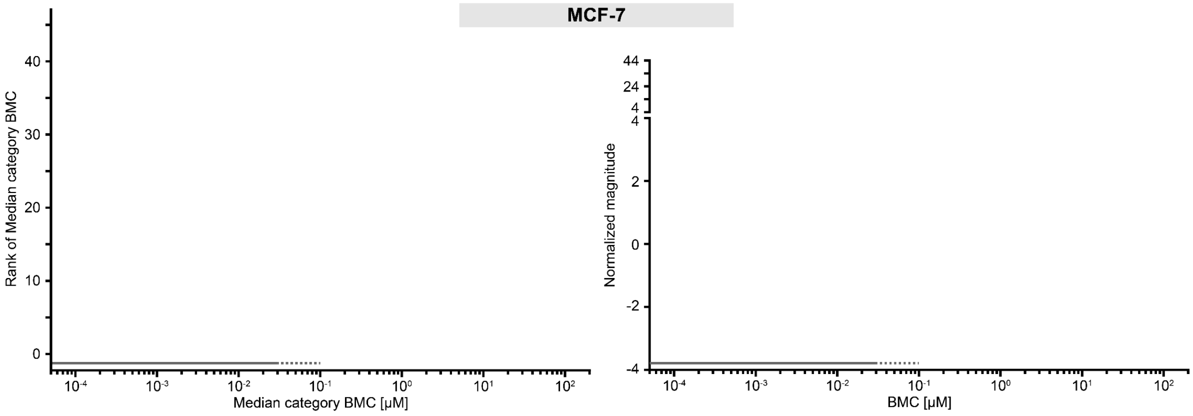

BMC accumulation and magnitude plots (feature category-level)

Substance:	Staining method:	Image analysis software:
**Brefeldin A**	**Cell Painting Plus**	**Cell Profiler**

Channels: Generic, Actin, RNA, Lyso, DNA, Golgi, Mito, ER

Modules: N Number, M Morphology, I Intensity, G Granularity, D Distribution, T Texture

Regions: Cell, Nucleus, Nucleoli, Cytoplasm, Membrane, Ring

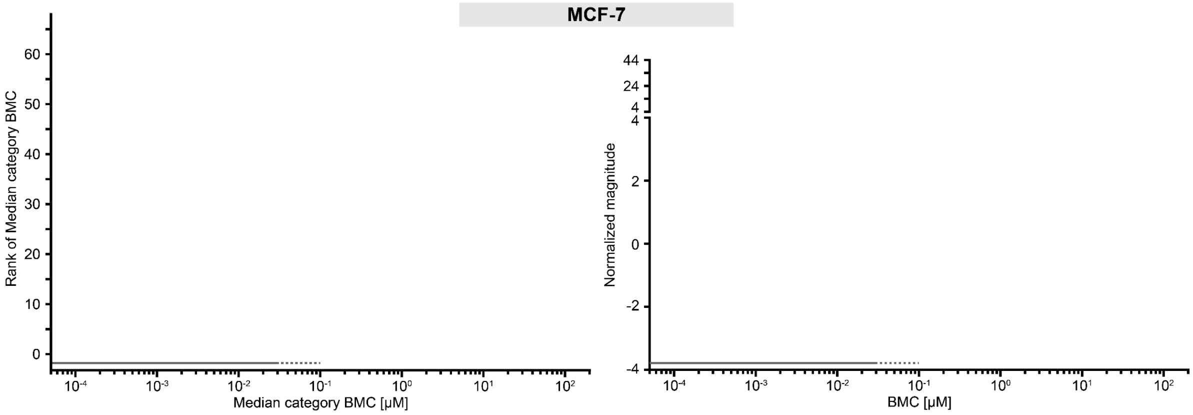

For comparison (from Supplementary Figure 4A):

BMC accumulation and magnitude plots (feature category-level)

Substance:	Staining method:	Image analysis software:
**Brefeldin A**	**Cell Painting Plus**	**Harmony**

Channels: Generic, Actin, RNA, Lyso, DNA, Golgi, Mito, ER

Modules: N Number, M Morphology, I Intensity, S Symmetry, C Compactness, D Distribution, T Texture

Regions: Cell, Nucleus, Nucleoli, Cytoplasm, Membrane, Ring

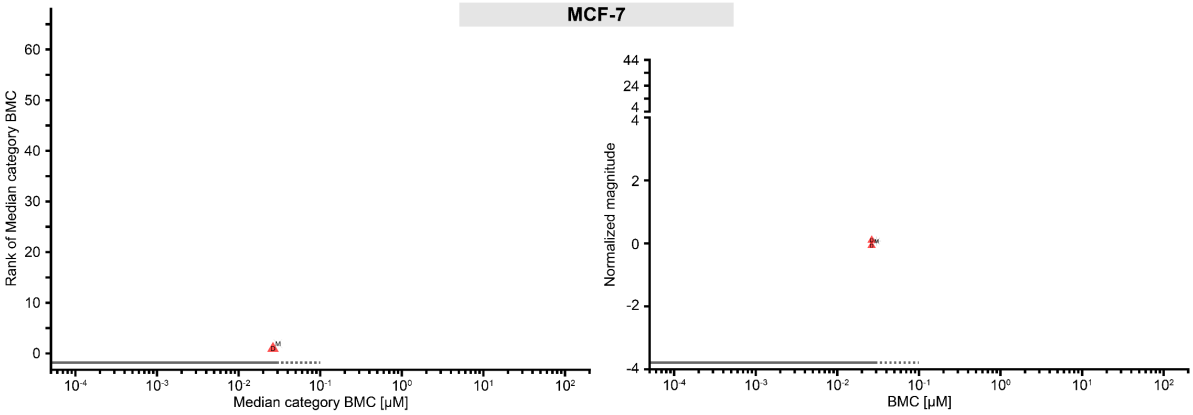

Supplementary Figure 5C

BMC accumulation and magnitude plots (feature category-level)

Substance:	Staining method:	Image analysis software:
**Cytochalasin D**	**Cell Painting**	**Harmony**

Channels: Generic, DNA, RNA/ER, AGP, Mito
Modules: N Number, M Morphology, I Intensity, S Symmetry, C Compactness, D Distribution, T Texture
Regions: Cell, Nucleus, Nucleoli, Cytoplasm, Membrane, Ring

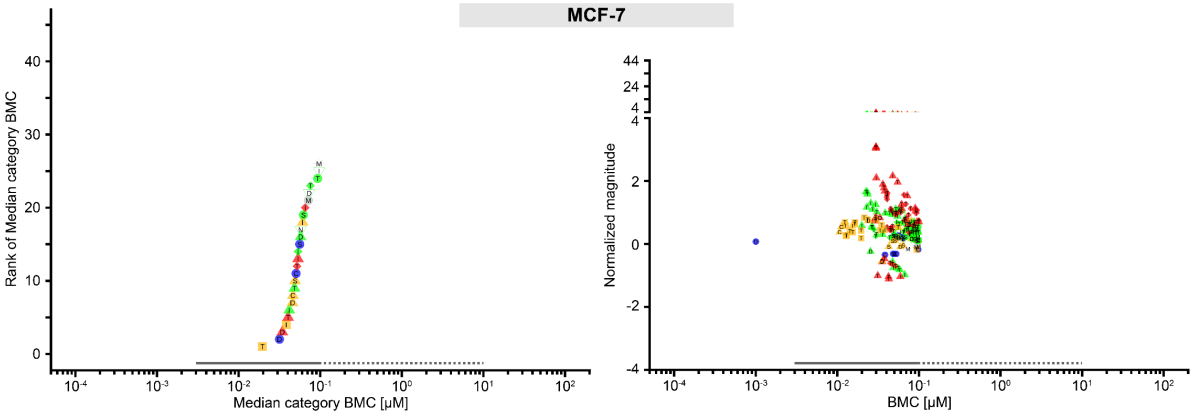

BMC accumulation and magnitude plots (feature category-level)

Substance:	Staining method:	Image analysis software:
**Cytochalasin D**	**Cell Painting Plus**	**Cell Profiler**

Channels: Generic, Actin, RNA, Lyso, DNA, Golgi, Mito, ER
Modules: N Number, M Morphology, I Intensity, G Granularity, D Distribution, T Texture
Regions: Cell, Nucleus, Nucleoli, Cytoplasm, Membrane, Ring

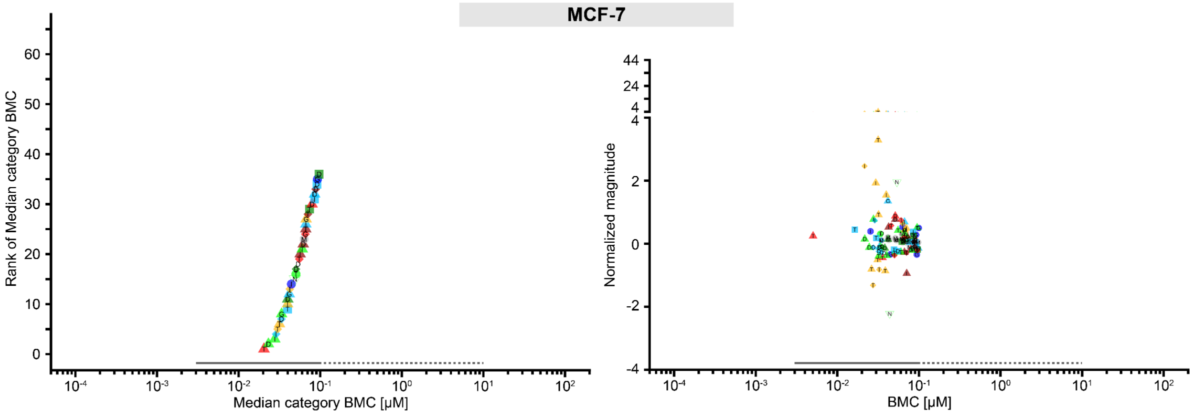

For comparison (from Supplementary Figure 4A):

BMC accumulation and magnitude plots (feature category-level)

Substance:	Staining method:	Image analysis software:
**Cytochalasin D**	**Cell Painting Plus**	**Harmony**

Channels: Generic, Actin, RNA, Lyso, DNA, Golgi, Mito, ER
Modules: N Number, M Morphology, I Intensity, S Symmetry, C Compactness, D Distribution, T Texture
Regions: Cell, Nucleus, Nucleoli, Cytoplasm, Membrane, Ring

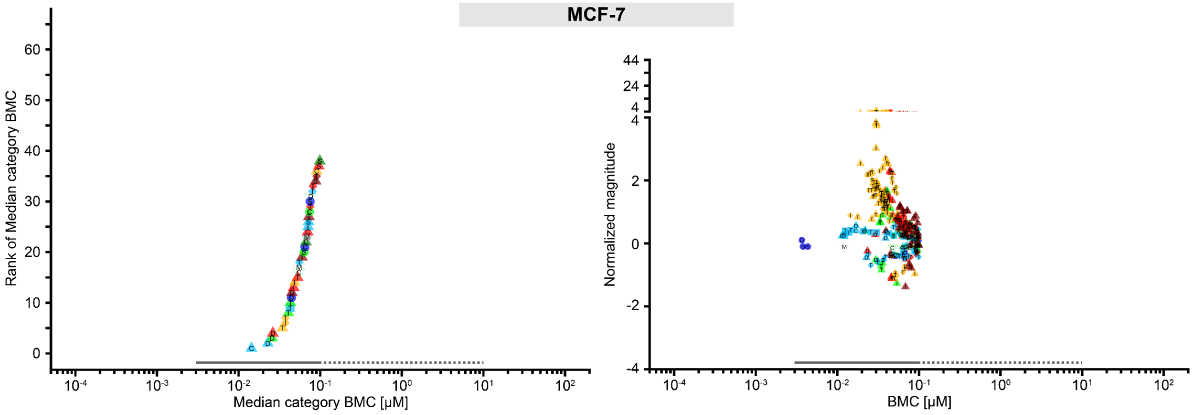

Supplementary Figure 5D

BMC accumulation and magnitude plots (feature category-level)

Substance:	Staining method:	Image analysis software:
**Etoposide**	**Cell Painting**	**Harmony**

Channels: Generic, DNA, RNA/ER, AGP, Mito

Modules: N Number, M Morphology, I Intensity, S Symmetry, C Compactness, D Distribution, T Texture

Regions: Cell, Nucleus, Nucleoli, Cytoplasm, Membrane, Ring

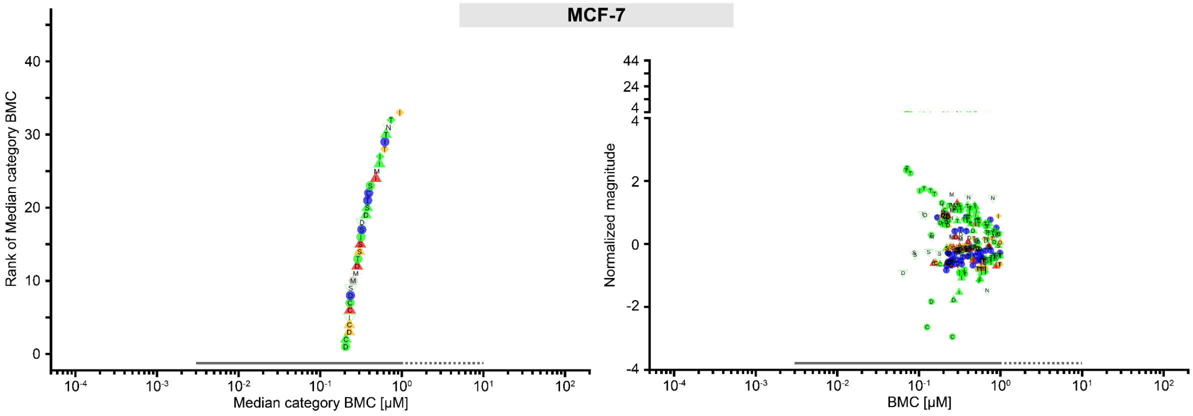

BMC accumulation and magnitude plots (feature category-level)

Substance:	Staining method:	Image analysis software:
**Etoposide**	**Cell Painting Plus**	**Cell Profiler**

Channels: Generic, Actin, RNA, Lyso, DNA, Golgi, Mito, ER

Modules: N Number, M Morphology, I Intensity, G Granularity, D Distribution, T Texture

Regions: Cell, Nucleus, Nucleoli, Cytoplasm, Membrane, Ring

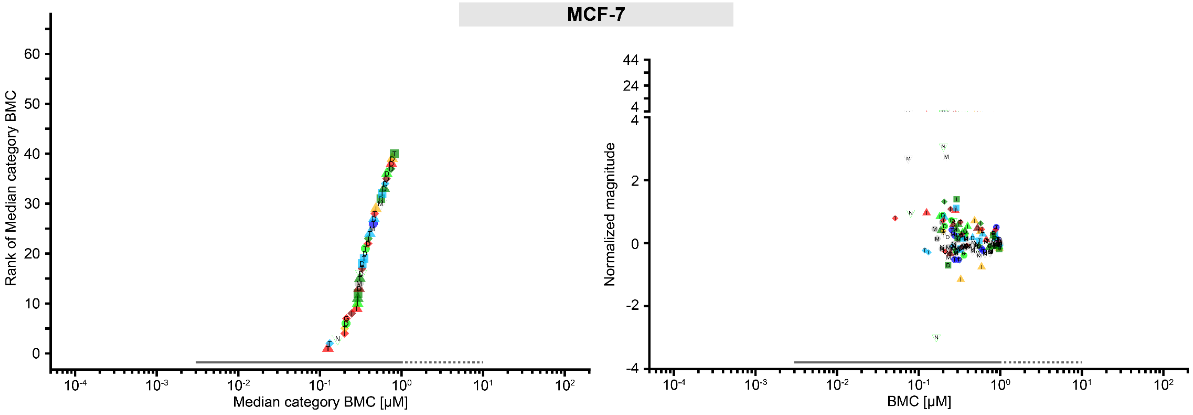

For comparison (from Supplementary Figure 4A):

BMC accumulation and magnitude plots (feature category-level)

Substance:	Staining method:	Image analysis software:
**Etoposide**	**Cell Painting Plus**	**Harmony**

Channels: Generic, Actin, RNA, Lyso, DNA, Golgi, Mito, ER

Modules: N Number, M Morphology, I Intensity, S Symmetry, C Compactness, D Distribution, T Texture

Regions: Cell, Nucleus, Nucleoli, Cytoplasm, Membrane, Ring

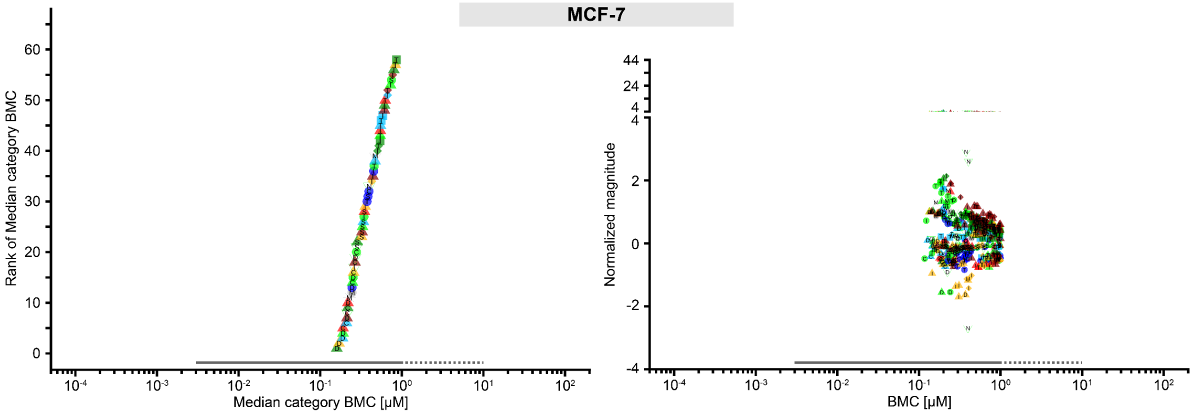

Supplementary Figure 5E

BMC accumulation and magnitude plots (feature category-level)

Substance:	Staining method:	Image analysis software:
**Fluphenazine**	**Cell Painting**	**Harmony**

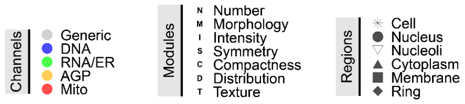

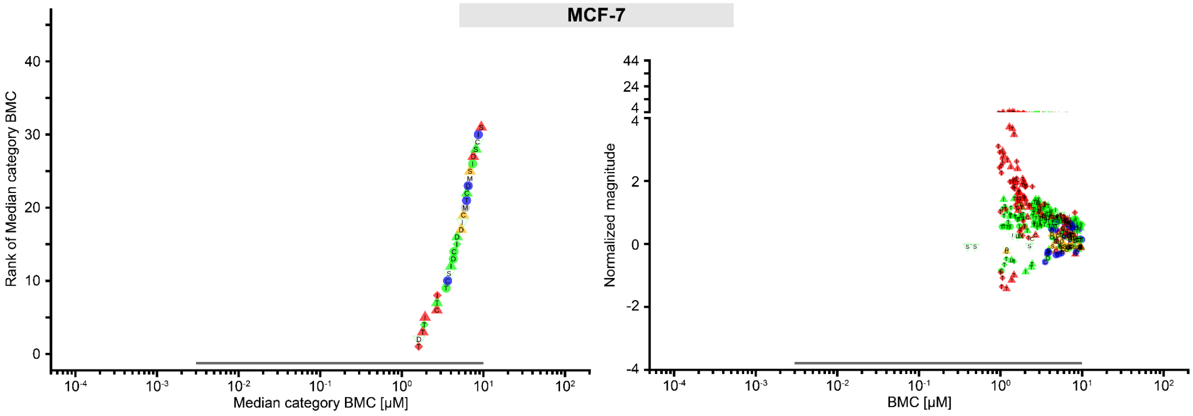

BMC accumulation and magnitude plots (feature category-level)

Substance:	Staining method:	Image analysis software:
**Fluphenazine**	**Cell Painting Plus**	**Cell Profiler**

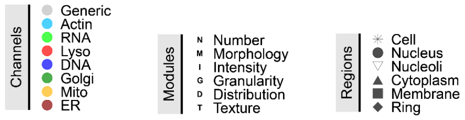

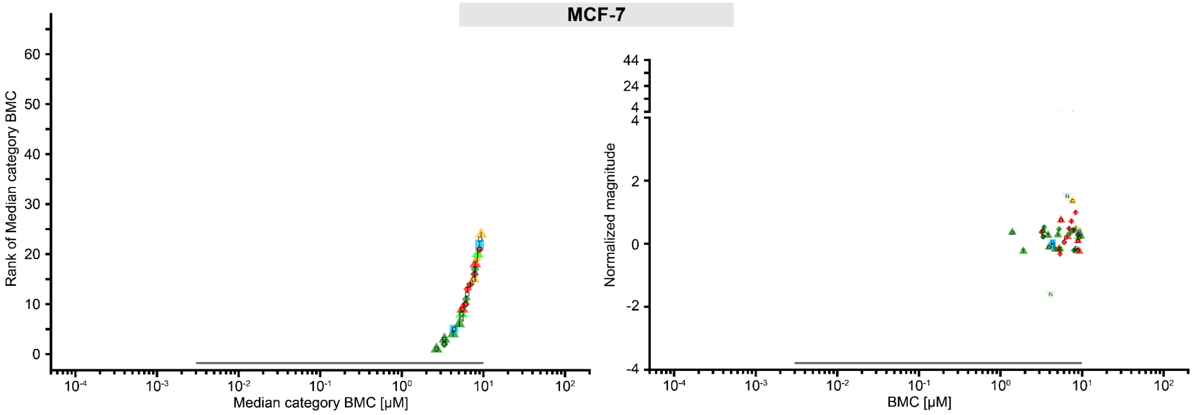

For comparison (from Supplementary Figure 4A):

BMC accumulation and magnitude plots (feature category-level)

Substance:	Staining method:	Image analysis software:
**Fluphenazine**	**Cell Painting Plus**	**Harmony**

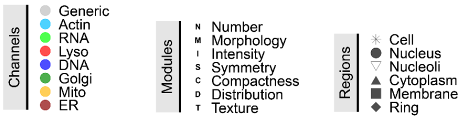

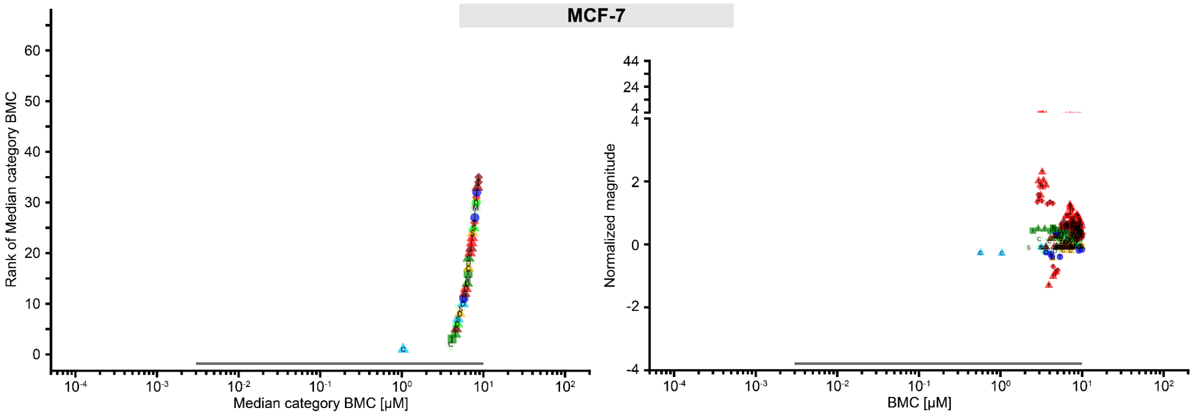

Supplementary Figure 5F

BMC accumulation and magnitude plots (feature category-level)

Substance:	Staining method:	Image analysis software:
**Fulvestrant**	**Cell Painting**	**Harmony**

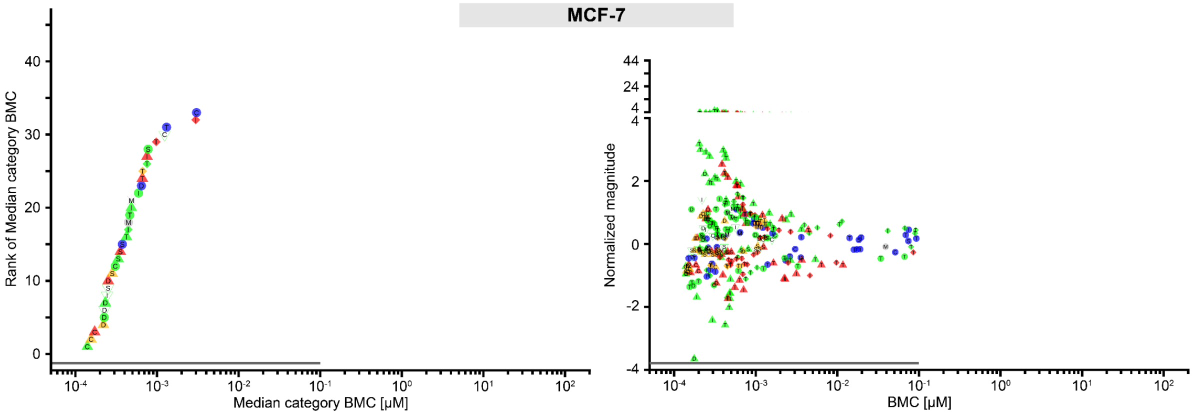

BMC accumulation and magnitude plots (feature category-level)

Substance:	Staining method:	Image analysis software:
**Fulvestrant**	**Cell Painting Plus**	**Cell Profiler**

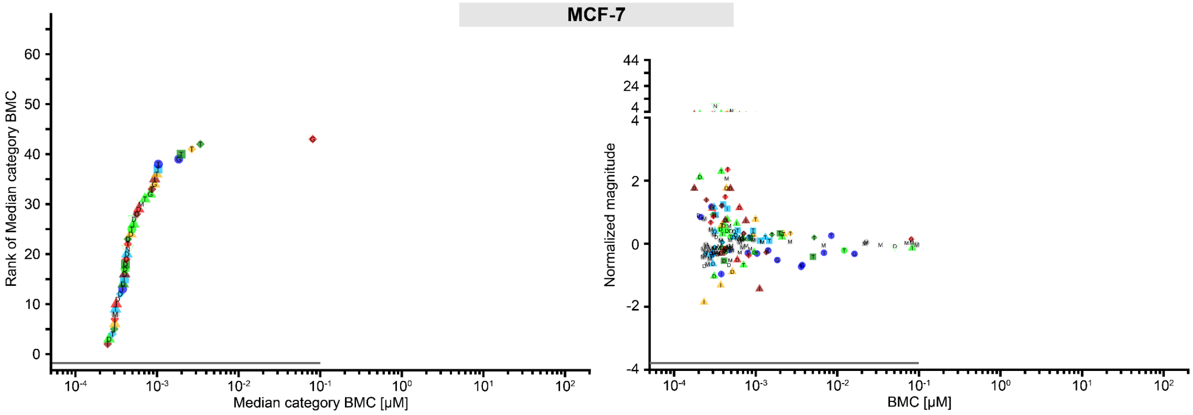

For comparison (from Supplementary Figure 4A):

BMC accumulation and magnitude plots (feature category-level)

Substance:	Staining method:	Image analysis software:
**Fulvestrant**	**Cell Painting Plus**	**Harmony**

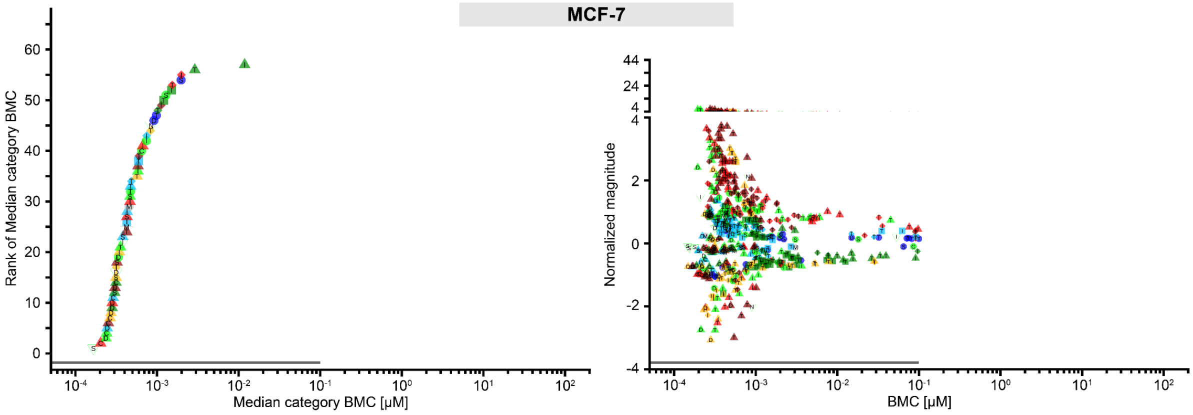

Supplementary Figure 5G

BMC accumulation and magnitude plots (feature category-level)

Substance:	Staining method:	Image analysis software:
**Latrunculin B**	**Cell Painting**	**Harmony**

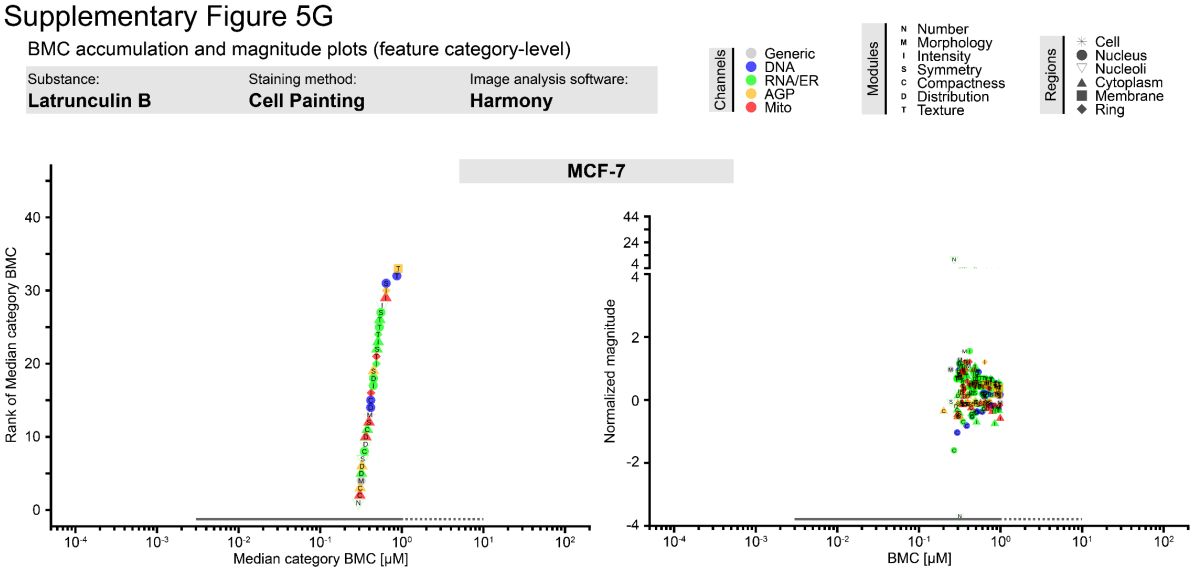

BMC accumulation and magnitude plots (feature category-level)

Substance:	Staining method:	Image analysis software:
**Latrunculin B**	**Cell Painting Plus**	**Cell Profiler**

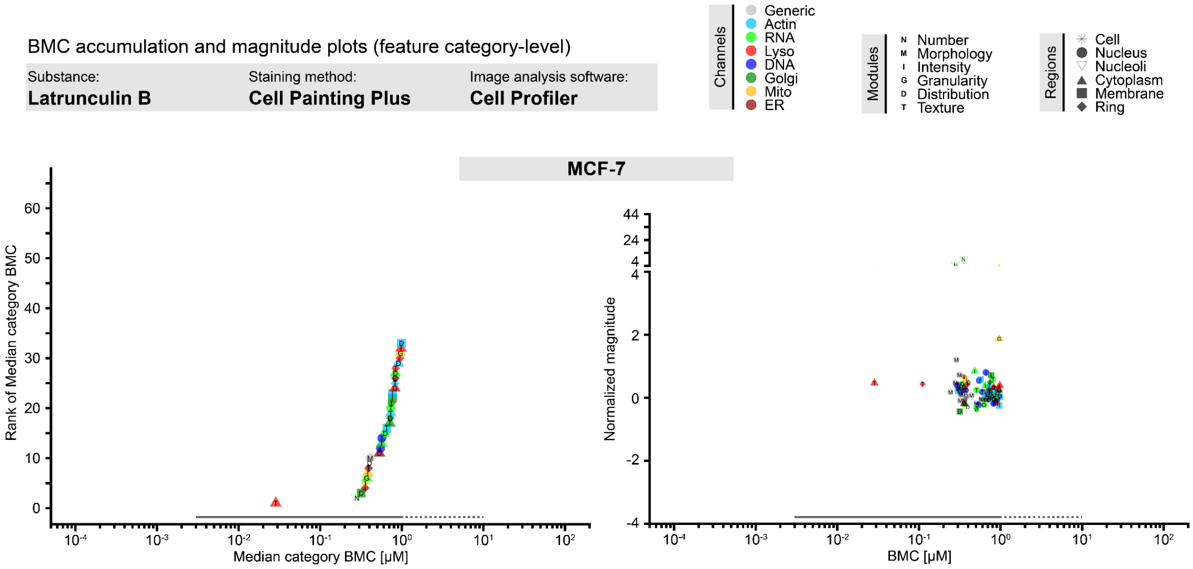

For comparison (from Supplementary Figure 4A):

BMC accumulation and magnitude plots (feature category-level)

Substance:	Staining method:	Image analysis software:
**Latrunculin B**	**Cell Painting Plus**	**Harmony**

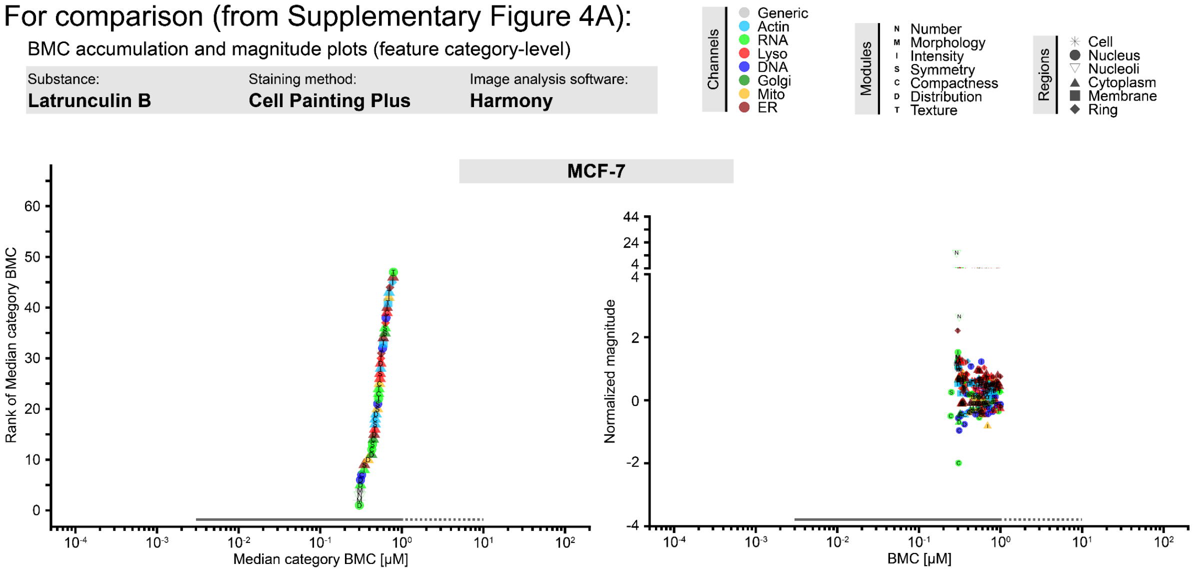

Supplementary Figure 5H

BMC accumulation and magnitude plots (feature category-level)

Substance:	Staining method:	Image analysis software:
**Nocodazole**	**Cell Painting**	**Harmony**

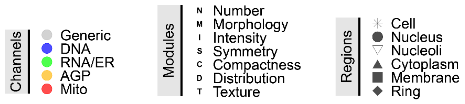

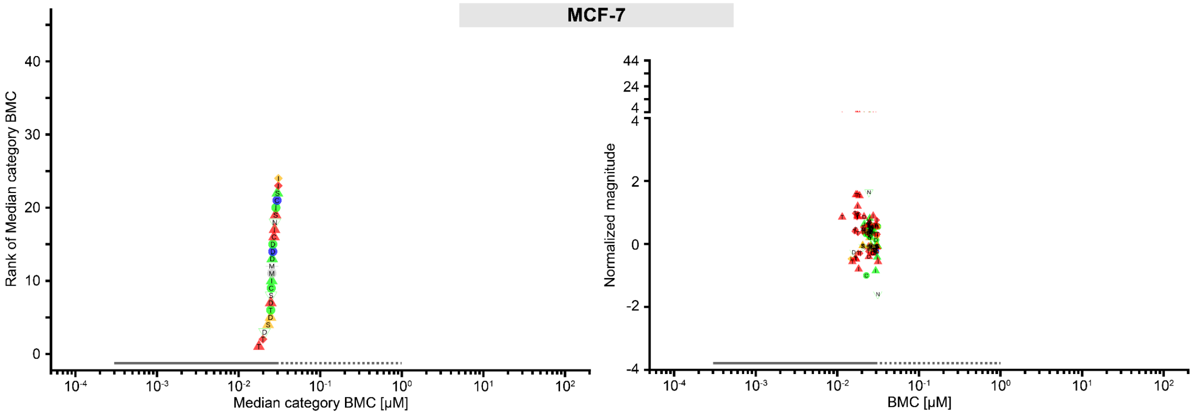

BMC accumulation and magnitude plots (feature category-level)

Substance:	Staining method:	Image analysis software:
**Nocodazole**	**Cell Painting Plus**	**Cell Profiler**

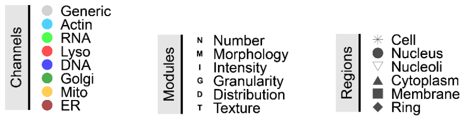

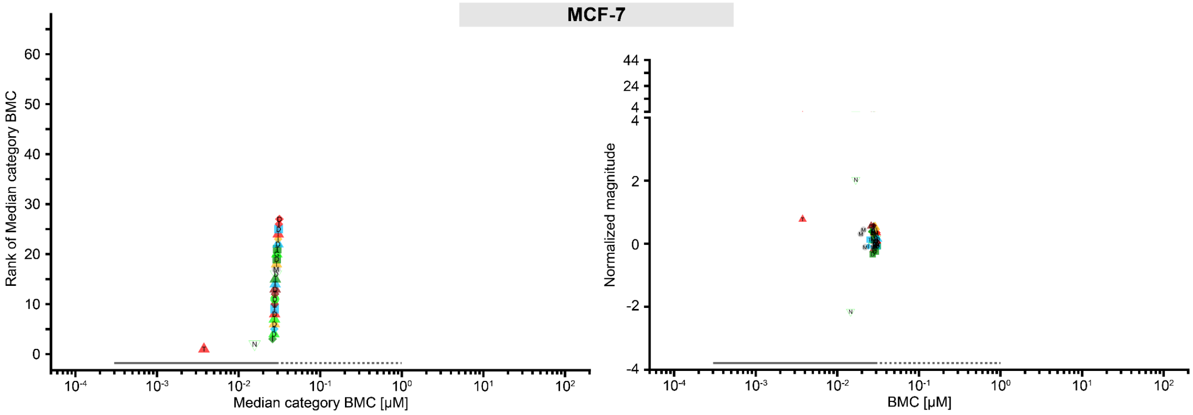

For comparison (from Supplementary Figure 4A):

BMC accumulation and magnitude plots (feature category-level)

Substance:	Staining method:	Image analysis software:
**Nocodazole**	**Cell Painting Plus**	**Harmony**

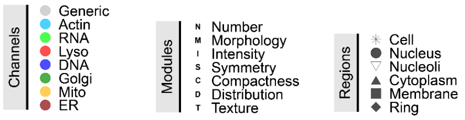

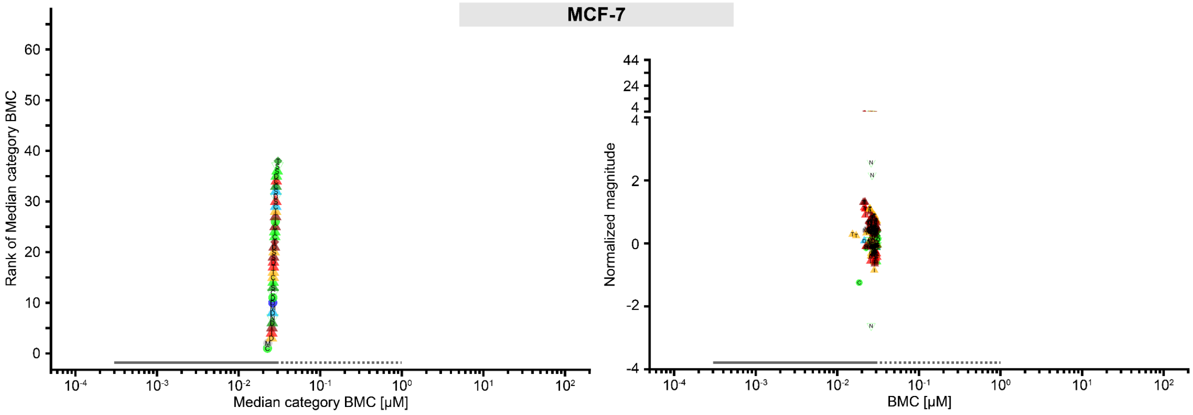

Supplementary Figure 5I

BMC accumulation and magnitude plots (feature category-level)

Substance:	Staining method:	Image analysis software:
**Rapamycin**	**Cell Painting**	**Harmony**

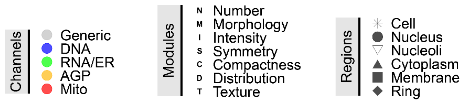

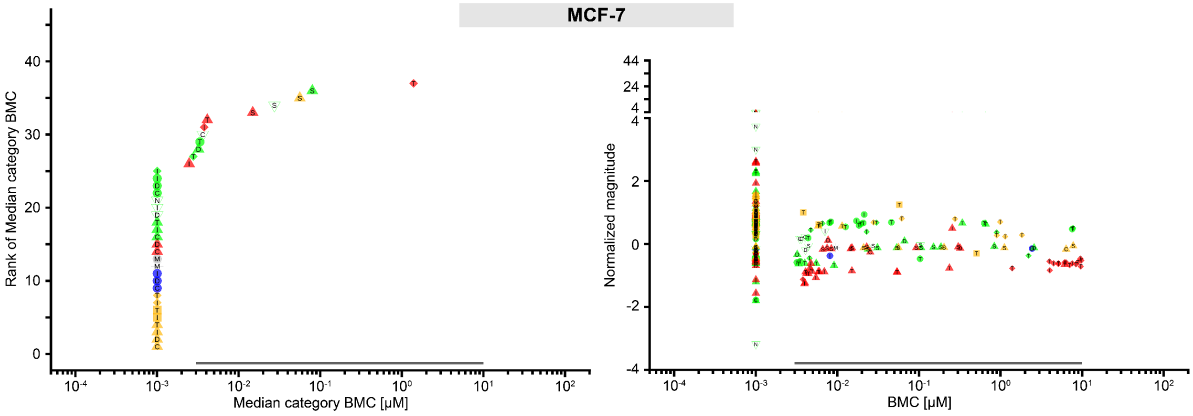

BMC accumulation and magnitude plots (feature category-level)

Substance:	Staining method:	Image analysis software:
**Rapamycin**	**Cell Painting Plus**	**Cell Profiler**

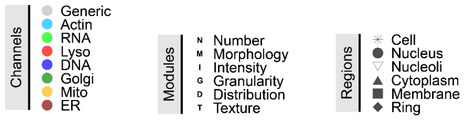

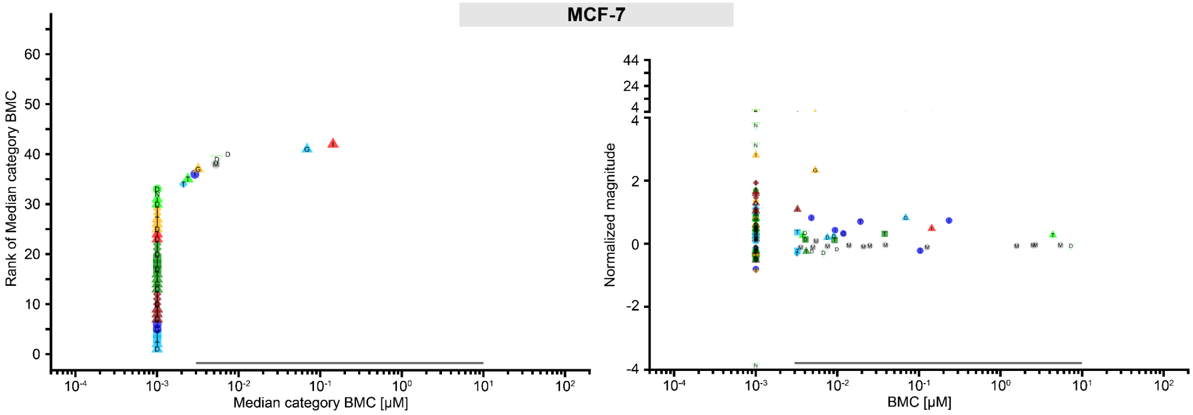

For comparison (from Supplementary Figure 4A):

BMC accumulation and magnitude plots (feature category-level)

Substance:	Staining method:	Image analysis software:
**Rapamycin**	**Cell Painting Plus**	**Harmony**

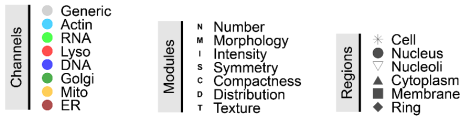

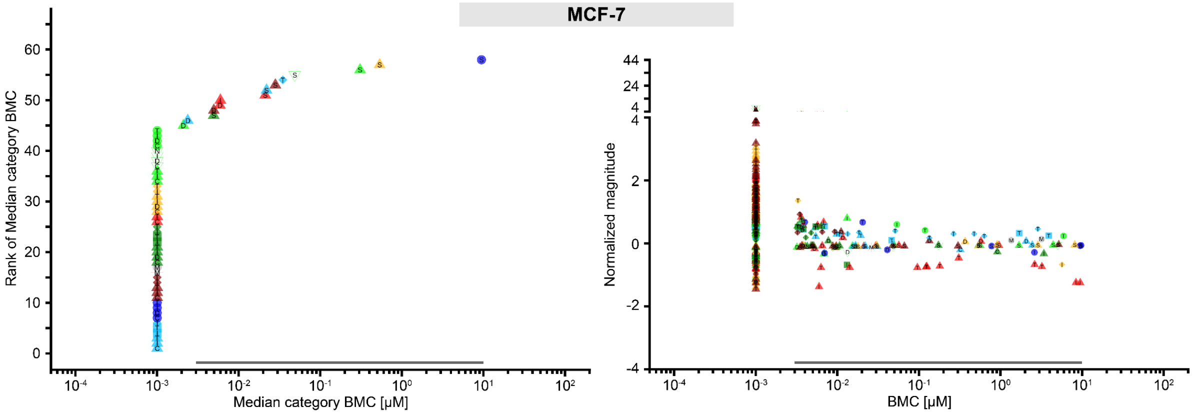

Supplementary Figure 5J

BMC accumulation and magnitude plots (feature category-level)

Substance:	Staining method:	Image analysis software:
**Rotenone**	**Cell Painting**	**Harmony**

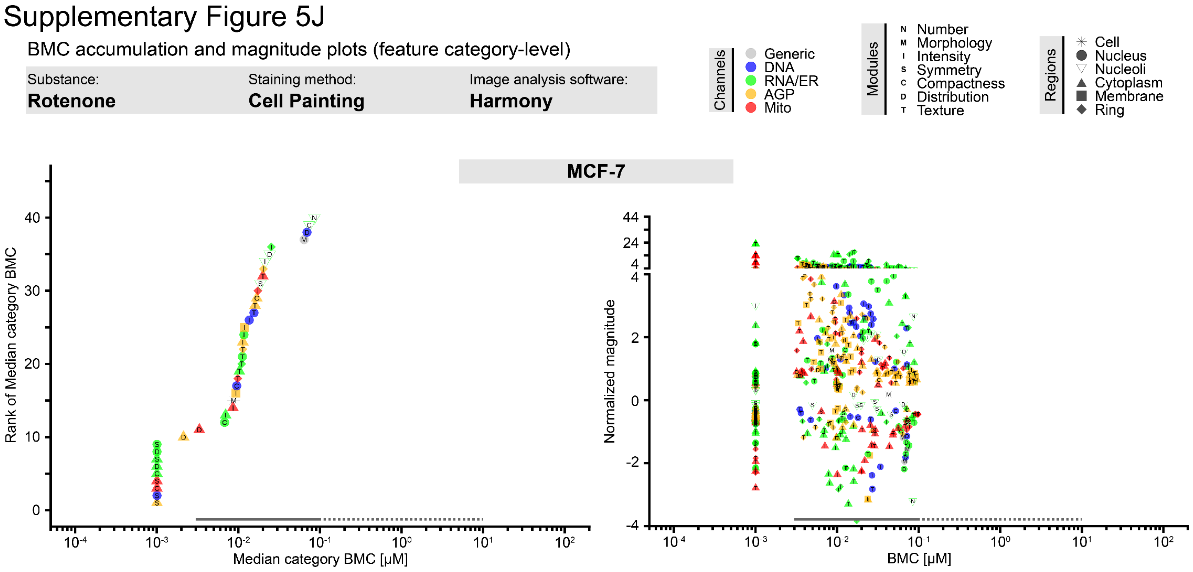

BMC accumulation and magnitude plots (feature category-level)

Substance:	Staining method:	Image analysis software:
**Rotenone**	**Cell Painting Plus**	**Cell Profiler**

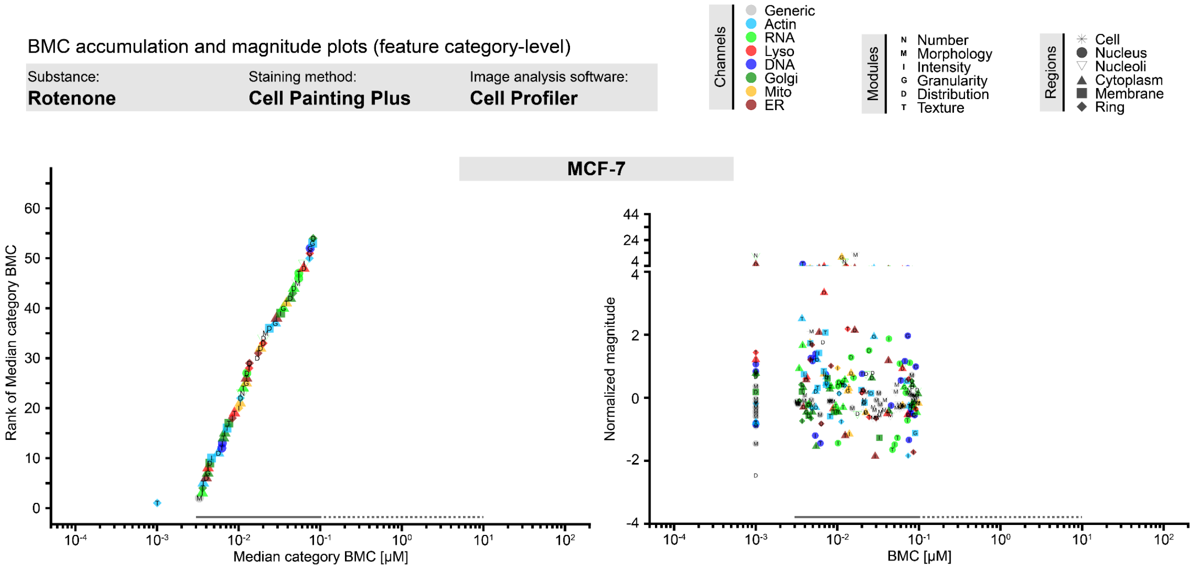

For comparison (from Supplementary Figure 4A):

BMC accumulation and magnitude plots (feature category-level)

Substance:	Staining method:	Image analysis software:
**Rotenone**	**Cell Painting Plus**	**Harmony**

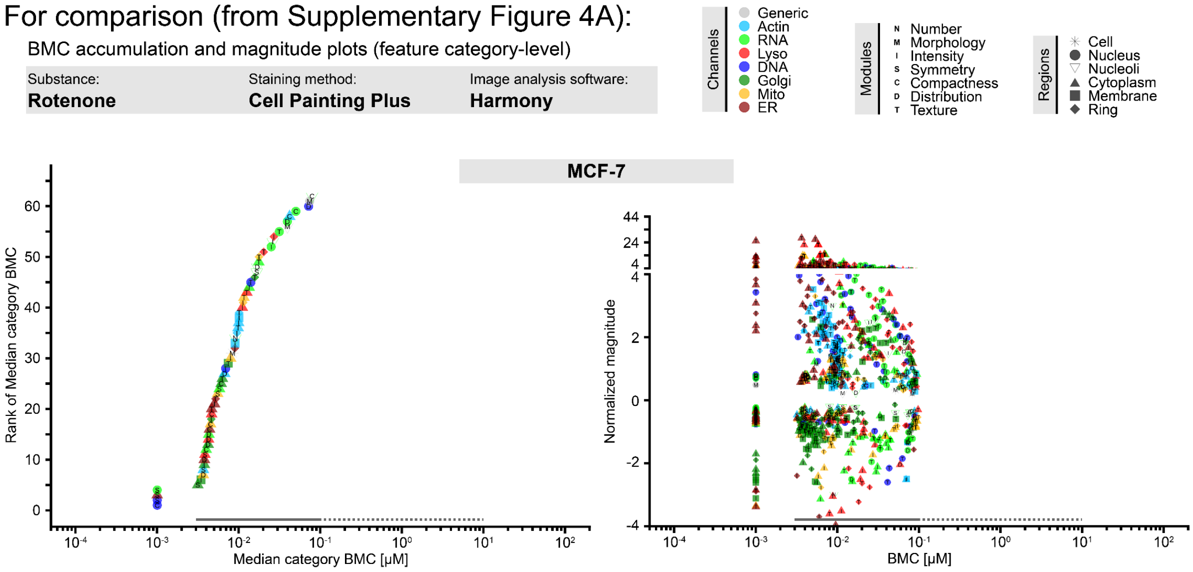

Supplementary Figure 5K

BMC accumulation and magnitude plots (feature category-level)

Substance:	Staining method:	Image analysis software:
**Saccharin**	**Cell Painting**	**Harmony**

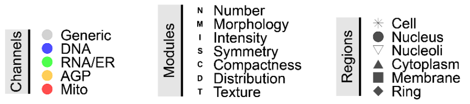

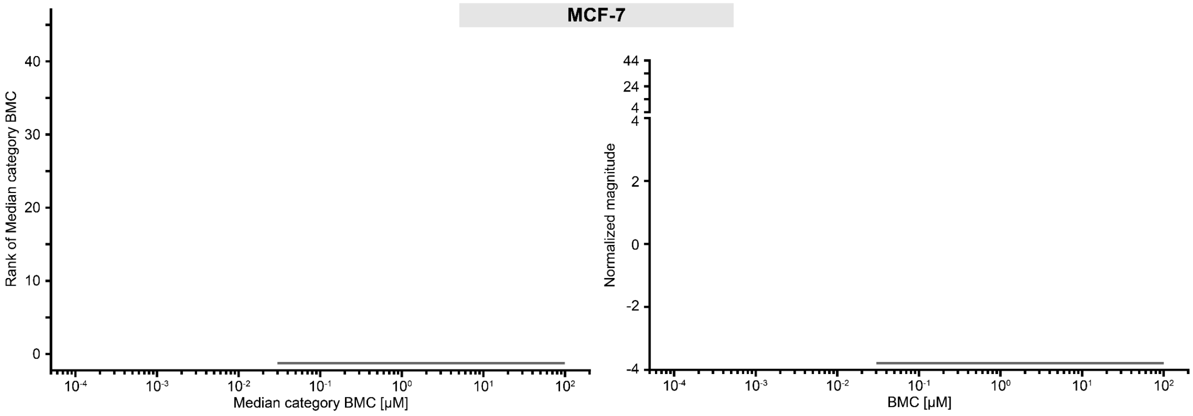

BMC accumulation and magnitude plots (feature category-level)

Substance:	Staining method:	Image analysis software:
**Saccharin**	**Cell Painting Plus**	**Cell Profiler**

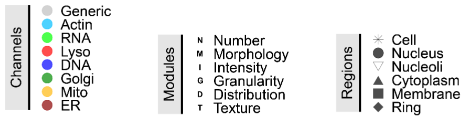

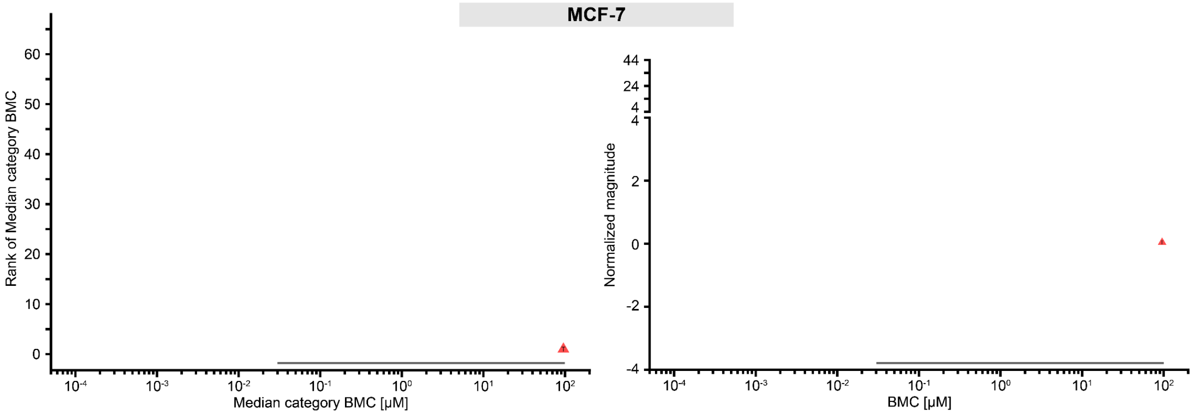

For comparison (from Supplementary Figure 4A):

BMC accumulation and magnitude plots (feature category-level)

Substance:	Staining method:	Image analysis software:
**Saccharin**	**Cell Painting Plus**	**Harmony**

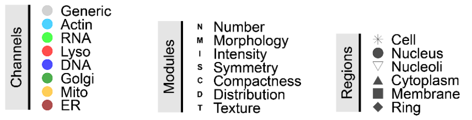

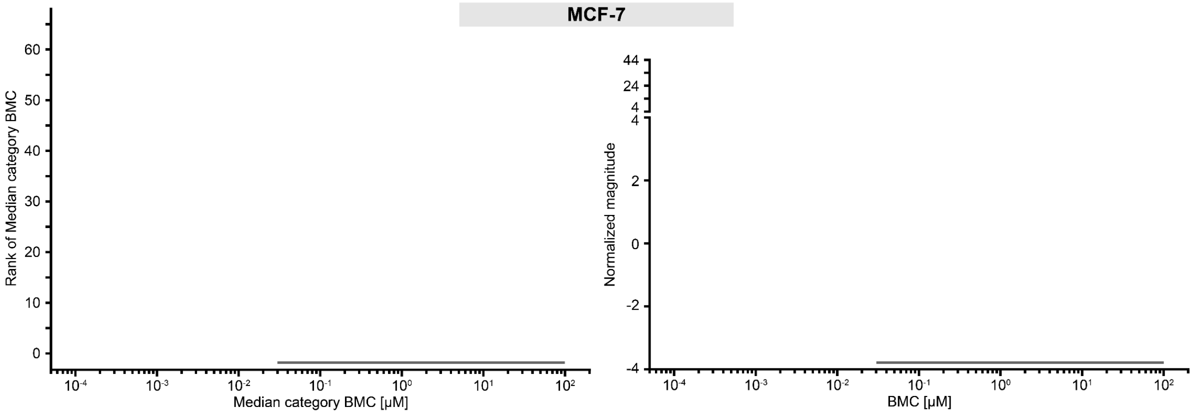

Supplementary Figure 5L

BMC accumulation and magnitude plots (feature category-level)

Substance:	Staining method:	Image analysis software:
**Siramesine**	**Cell Painting**	**Harmony**

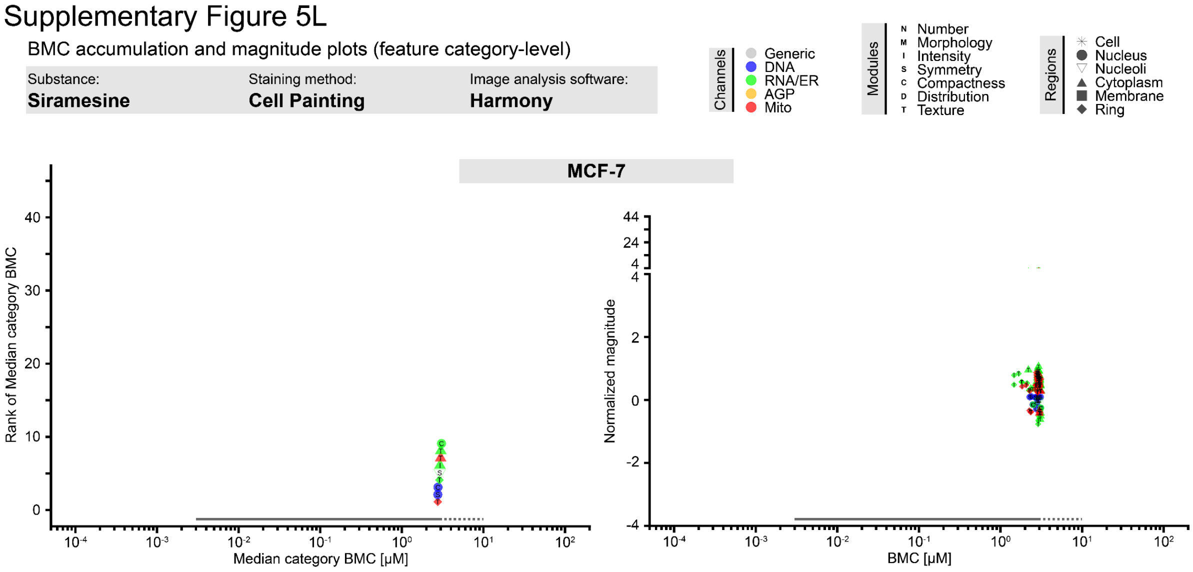

BMC accumulation and magnitude plots (feature category-level)

Substance:	Staining method:	Image analysis software:
**Siramesine**	**Cell Painting Plus**	**Cell Profiler**

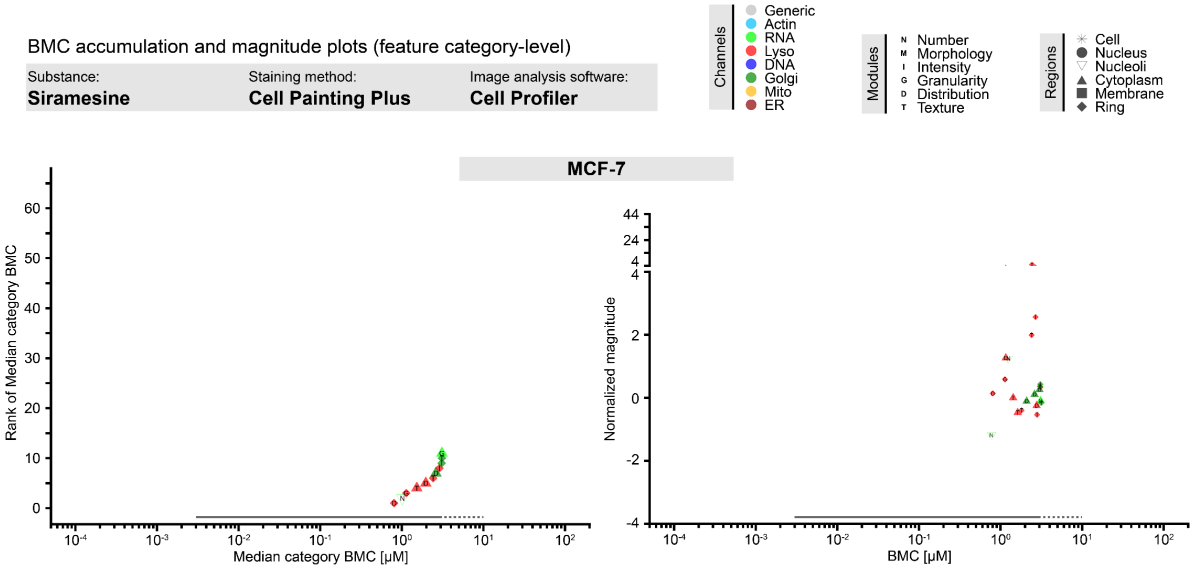

For comparison (from Supplementary Figure 4A):

BMC accumulation and magnitude plots (feature category-level)

Substance:	Staining method:	Image analysis software:
**Siramesine**	**Cell Painting Plus**	**Harmony**

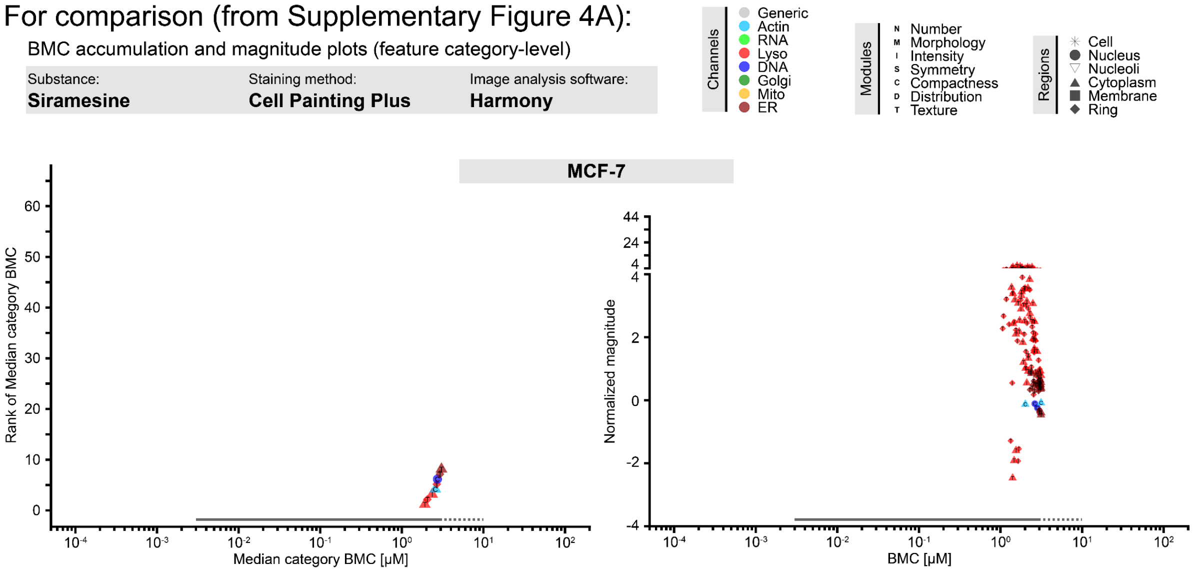

Supplementary Figure 5M

BMC accumulation and magnitude plots (feature category-level)

Substance:	Staining method:	Image analysis software:
**Sorbitol**	**Cell Painting**	**Harmony**

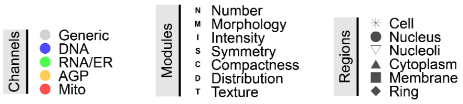

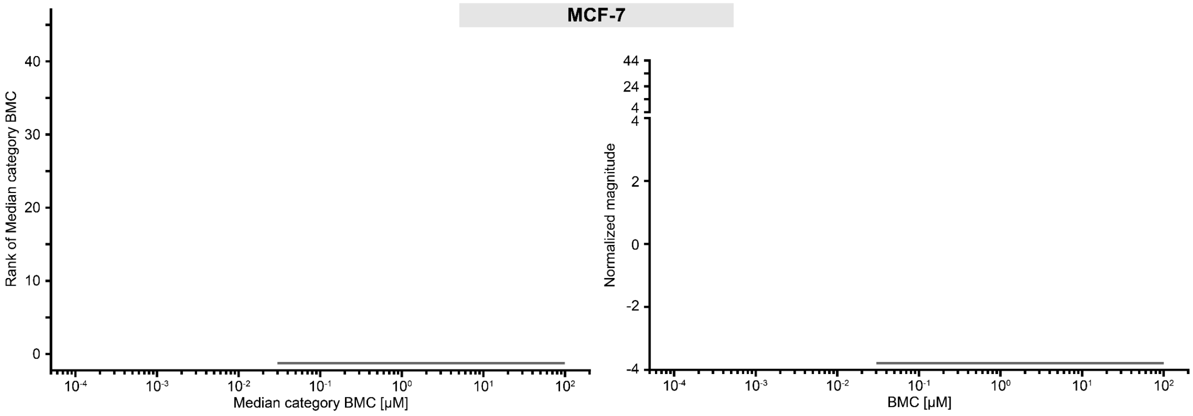

BMC accumulation and magnitude plots (feature category-level)

Substance:	Staining method:	Image analysis software:
**Sorbitol**	**Cell Painting Plus**	**Cell Profiler**

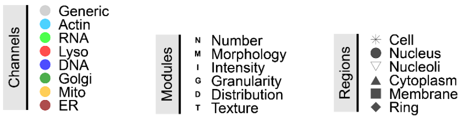

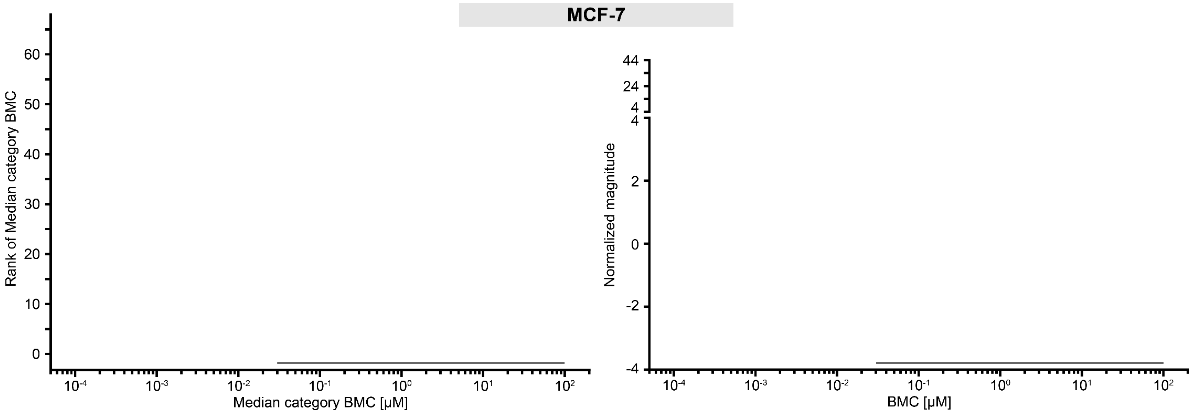

For comparison (from Supplementary Figure 4A):

BMC accumulation and magnitude plots (feature category-level)

Substance:	Staining method:	Image analysis software:
**Sorbitol**	**Cell Painting Plus**	**Harmony**

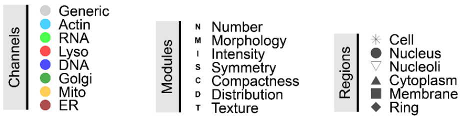

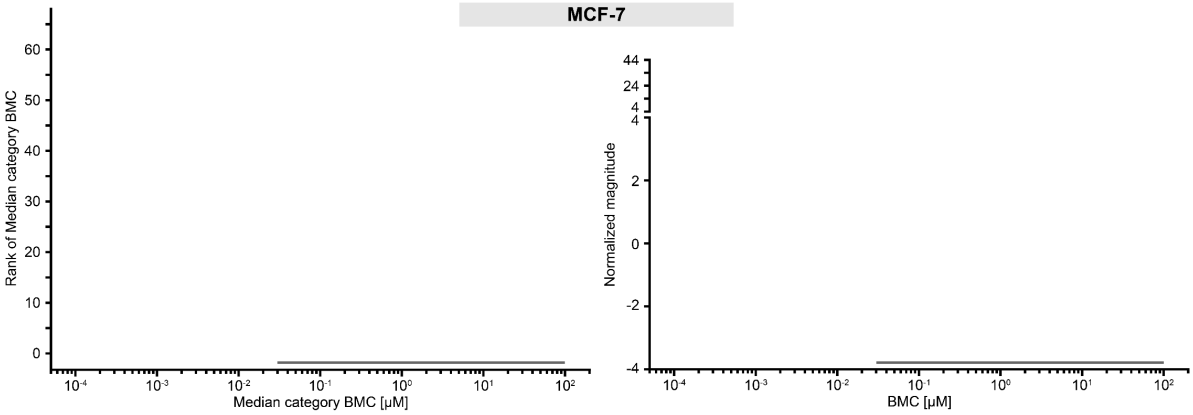

Supplementary Figure 5N

BMC accumulation and magnitude plots (feature category-level)

Substance:	Staining method:	Image analysis software:
**Sunitinib malate**	**Cell Painting**	**Harmony**

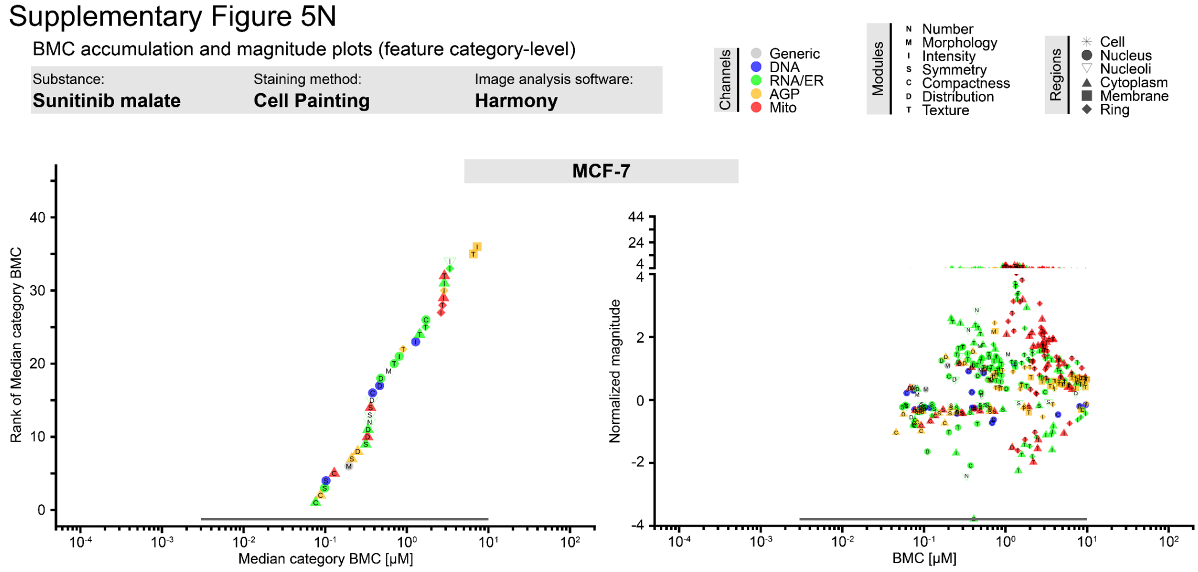

BMC accumulation and magnitude plots (feature category-level)

Substance:	Staining method:	Image analysis software:
**Sunitinib malate**	**Cell Painting Plus**	**Cell Profiler**

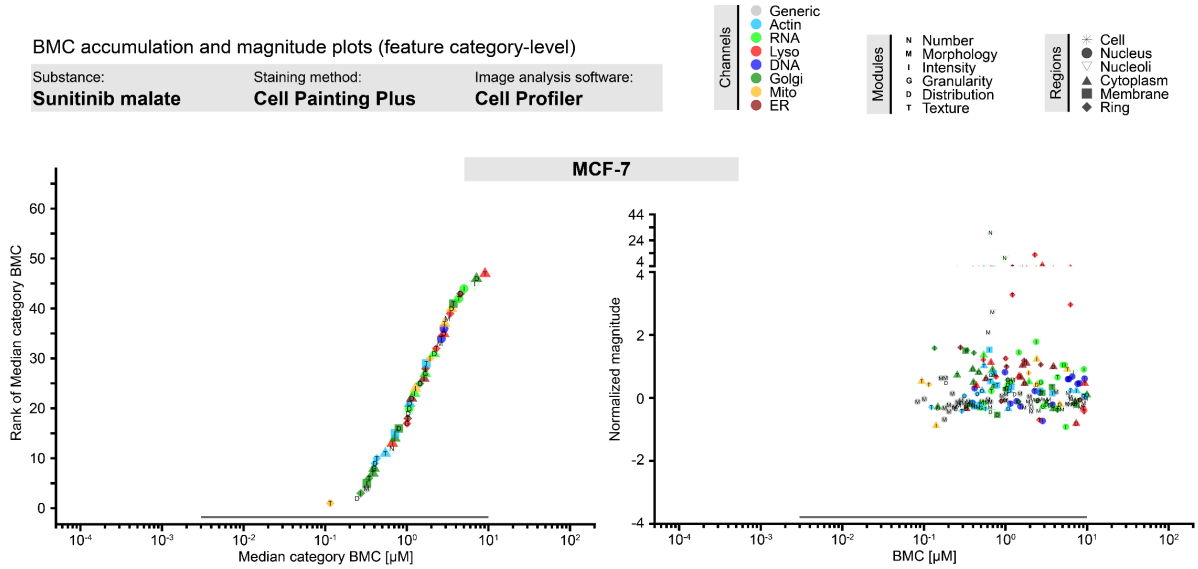

For comparison (from Supplementary Figure 4A):

BMC accumulation and magnitude plots (feature category-level)

Substance:	Staining method:	Image analysis software:
**Sunitinib malate**	**Cell Painting Plus**	**Harmony**

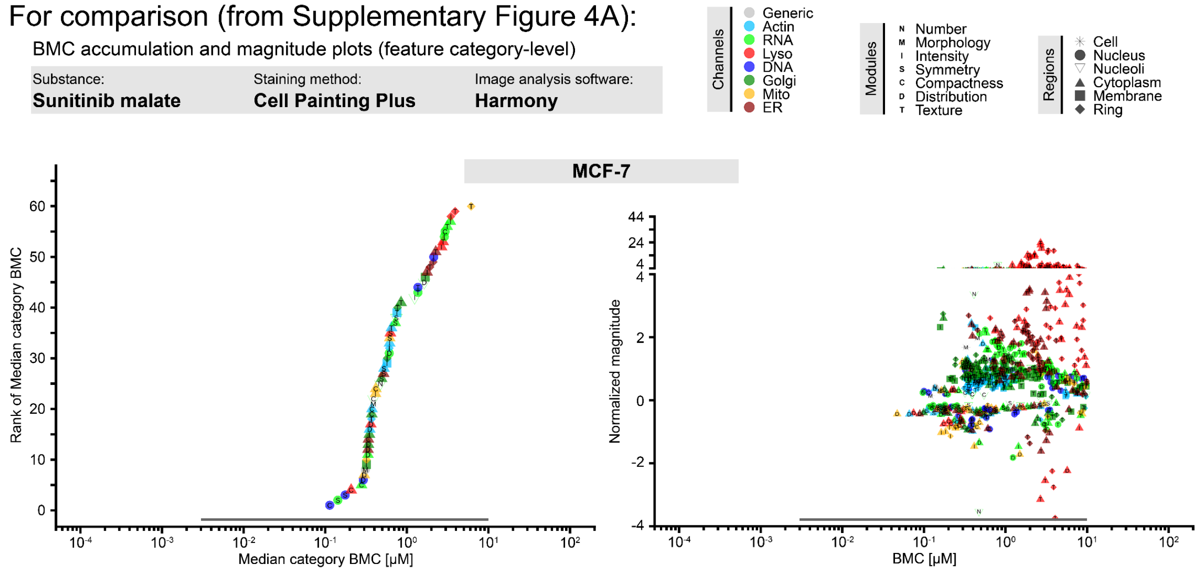

Supplementary Figure 5O

BMC accumulation and magnitude plots (feature category-level)

Substance:	Staining method:	Image analysis software:
**Tetrandrine**	**Cell Painting**	**Harmony**

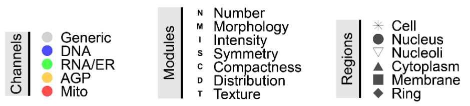

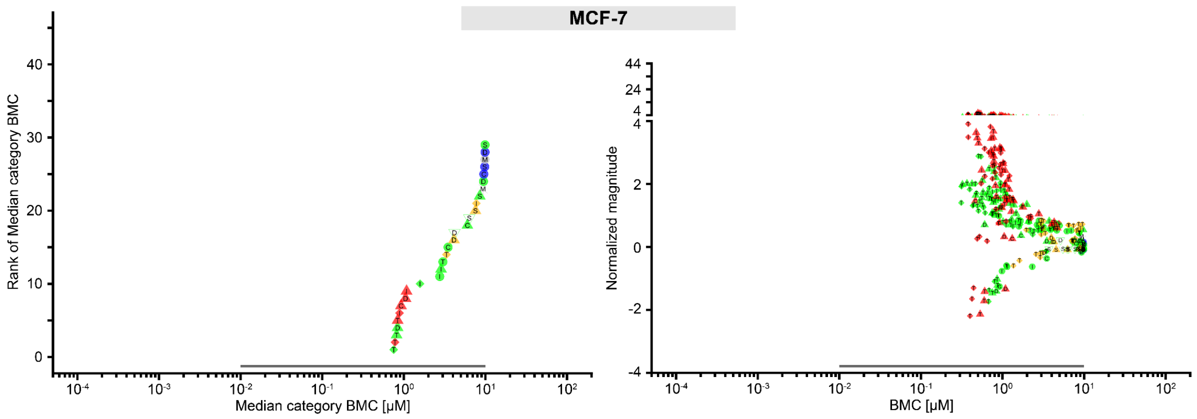

BMC accumulation and magnitude plots (feature category-level)

Substance:	Staining method:	Image analysis software:
**Tetrandrine**	**Cell Painting Plus**	**Cell Profiler**

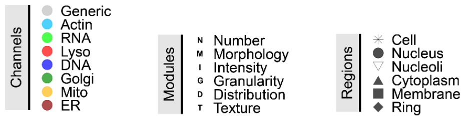

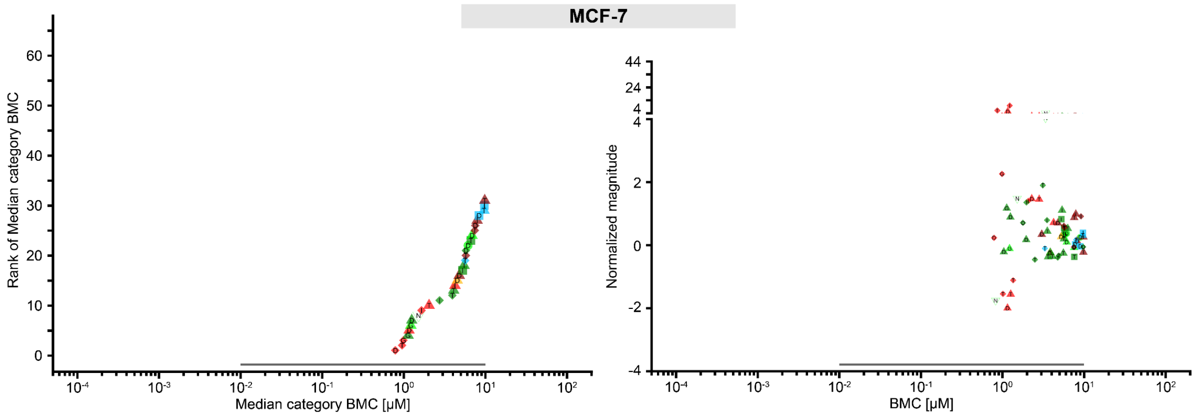

For comparison (from Supplementary Figure 4A):

BMC accumulation and magnitude plots (feature category-level)

Substance:	Staining method:	Image analysis software:
**Tetrandrine**	**Cell Painting Plus**	**Harmony**

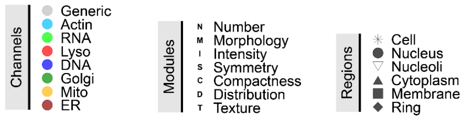

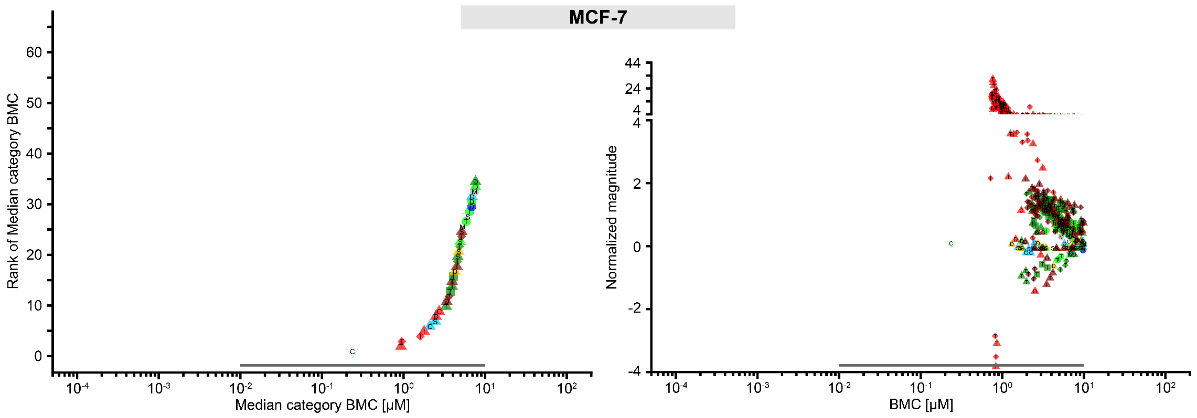

Supplementary Fig. 6. Differentiating between Actin and Golgi responses at different effective compound concentrations using latrunculin B.

(A) Representative images (Actin and Golgi channels) showing activities of latrunculin B on Actin- and Golgi-related features at a non-cytotoxic concentration compared to the DMSO solvent control across four different cell lines. N_{Biol} = 4. Scale bars = 20 µm.

(B) Corresponding BMC accumulation and magnitude plots showing latrunculin B effects on feature categories and single features across four different cell lines as described in Fig. 3D.

(C) Profile similarity plots showing the correlation of the phenotypic profiles (Spearman correlation of robust z-scores at the feature level, excluding Lyso features) of all reference compounds at each highest non-cytotoxic concentration in different cell lines. Compounds are assigned to one of six clusters (grey-shaded boxes) based on hierarchical clustering. Compounds are color-coded (cyan, orange, red) according to their annotated Actin-, Mito-, or Lyso-related MoA (see Fig. 2A). Colored, dashed boxes highlight correlation scores of compounds with the same annotated MoA. Input feature data (median of all N_{Tech} = 3 and N_{Biol} = 4 for each feature) are extracted from CPP images using the Harmony image analysis software. Correlation scores of negative control compounds (i.e., saccharine, sorbitol) are shown slightly transparent.

Source data are provided as a Source Data file.

Supplementary Figure 6

A Representative images of Actin and Golgi phenotypes showing latrunculin B effects in different cell lines

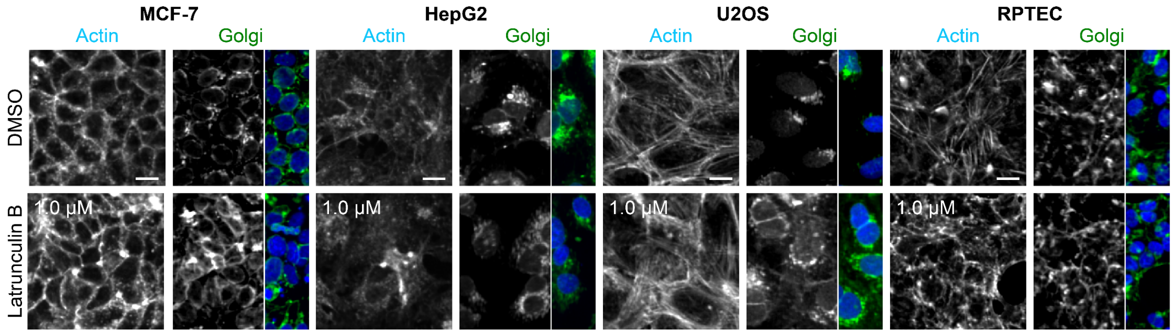

B BMC accumulation and magnitude plots (feature category-level) showing latrunculin B effects in different cell lines

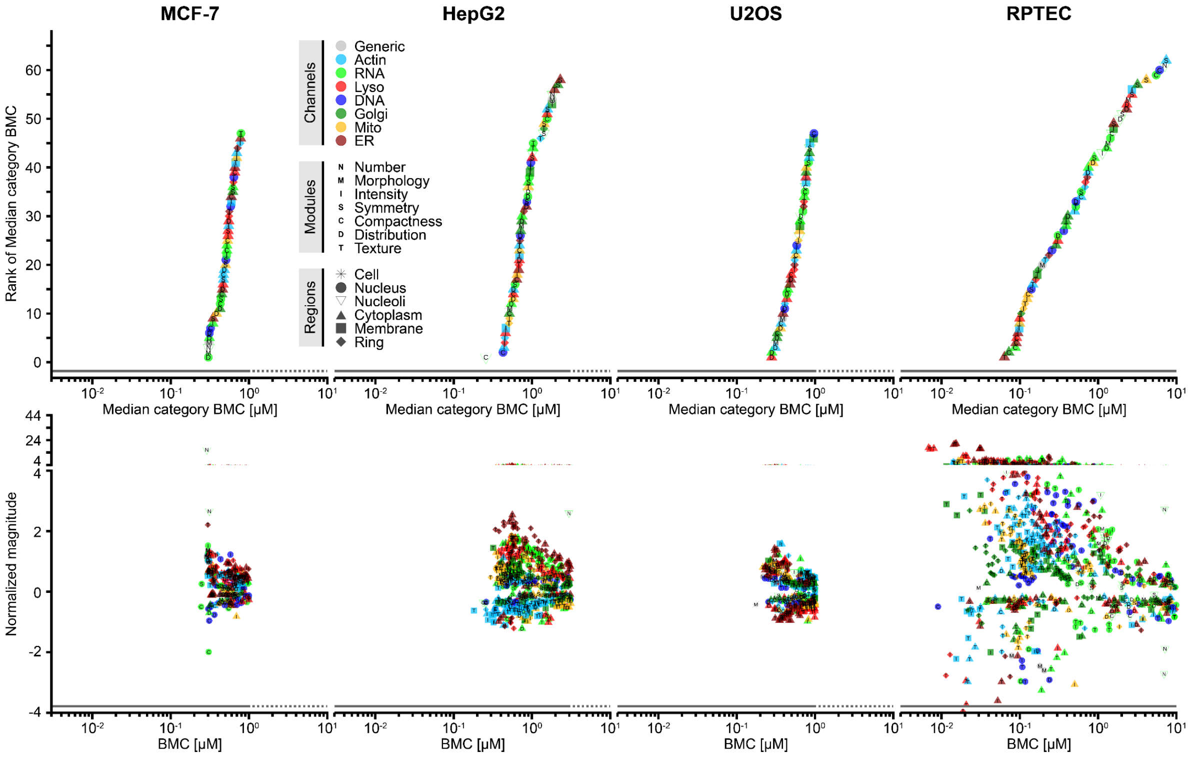

C Profile similarity plots (robust z-score, feature-level, **excluding** Lyso features) of all reference compounds at highest non-cytototoxic concentrations in different cell lines

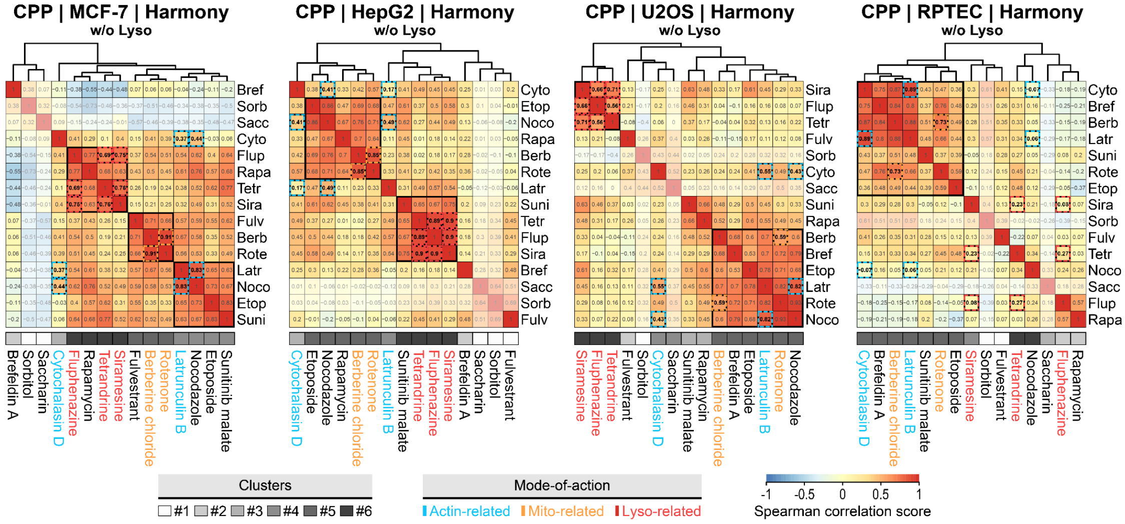

Supplementary Fig. 7. Elucidating compound effects on lysosomes using sunitinib malate.

(A) Representative images (Lyso, Golgi, and ER channels) showing activities of sunitinib malate on Lyso-, Golgi-, and ER-related features at a non-cytotoxic concentration compared to the DMSO solvent control across four different cell lines. N_{Biol} = 4. Scale bars = 20 μm.

(B) Corresponding BMC accumulation and magnitude plots showing sunitinib malate effects on feature categories and single features across four different cell lines as described in Fig. 3D.

(C) Profile similarity plots as described in Supplementary Fig. 6C but including Lyso features.

Source data are provided as a Source Data file.

Supplementary Figure 7

A Representative images of Lyso, Golgi, and ER phenotypes showing sunitinib malate effects in different cell lines

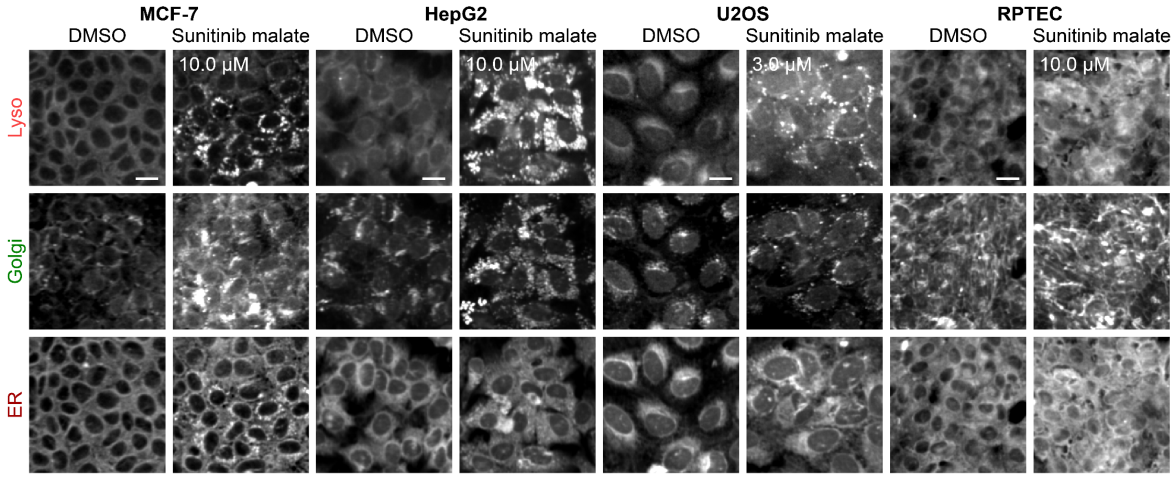

B BMC accumulation and magnitude plots (feature category-level) showing sunitinib malate effects in different cell lines

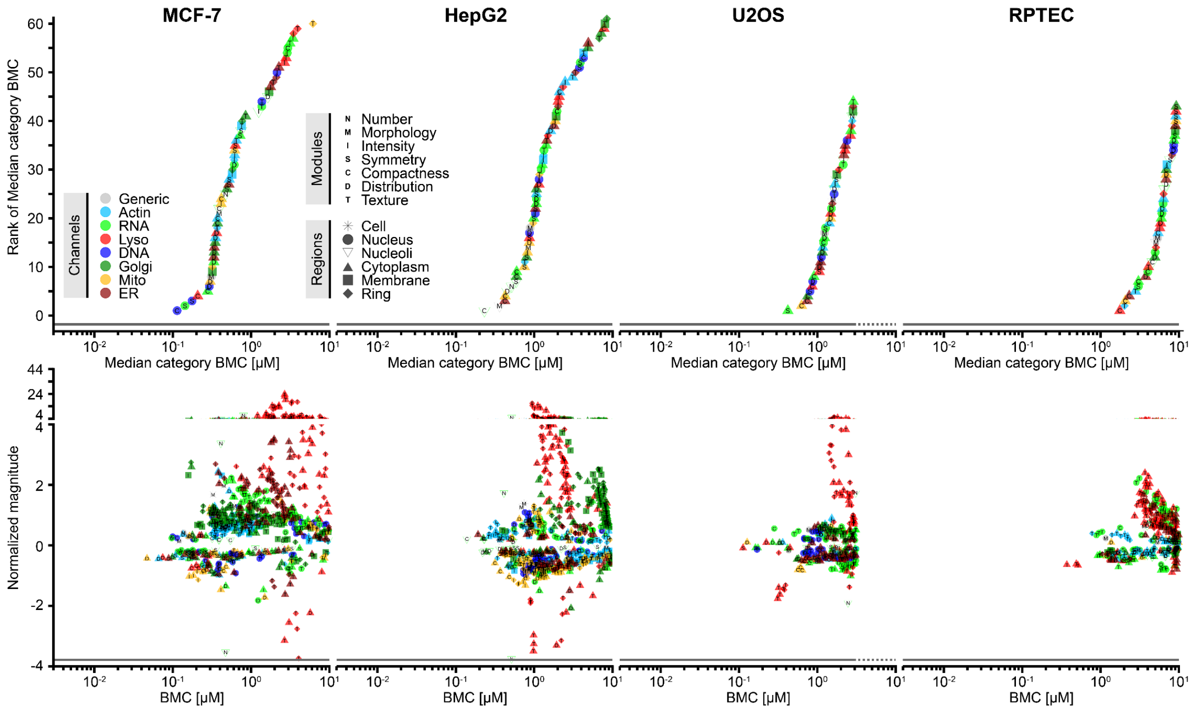

C Profile similarity plots (robust z-score, feature-level, **including** Lyso features) of all reference compounds at highest non-cytotoxic concentrations in different cell lines

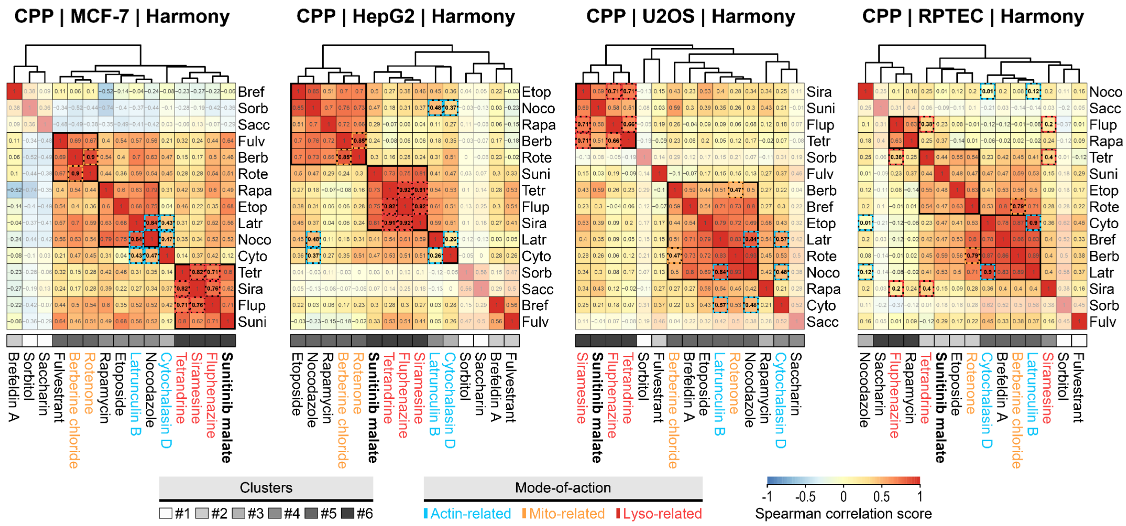

Supplementary Fig. 8. Elucidating compound effects on lysosomes using tetrandrine.

(A) Representative images (Lyso, Golgi, and ER channels) showing activities of tetrandrine on Lyso-, Golgi-, and ER-related features at a non-cytotoxic concentration compared to the DMSO solvent control across four different cell lines. N_{Biol} = 4. Scale bars = 20 µm.

(B) Corresponding BMC accumulation and magnitude plots showing tetrandrine effects on feature categories and single features across four different cell lines as described in Fig. 3D.

(C) Profile similarity plots showing the correlation of the phenotypic profiles (Spearman correlation of robust z-scores at the feature level, ex-/including Lyso features, with/without feature selection performed) of all reference compounds at each highest non-cytotoxic concentration in MCF-7 cells. Compounds are assigned to one of six clusters (grey-shaded boxes) based on hierarchical clustering. Compounds are color-coded (cyan, orange, red) according to their annotated Actin-, Mito-, or Lyso-related MoA (see Fig. 2A). Colored, dashed boxes highlight correlation scores of compounds with the same annotated MoA. Input feature data (median of all N_{Tech} = 3 and N_{Biol} = 4 for each feature) are extracted from CPP images using the Cell Profiler image analysis software. Correlation scores of negative control compounds (i.e., saccharine, sorbitol) are shown slightly transparent.

Source data are provided as a Source Data file.

Supplementary Figure 8

A Representative images of Lyso, Golgi, and ER phenotypes showing tetrandrine effects in different cell lines

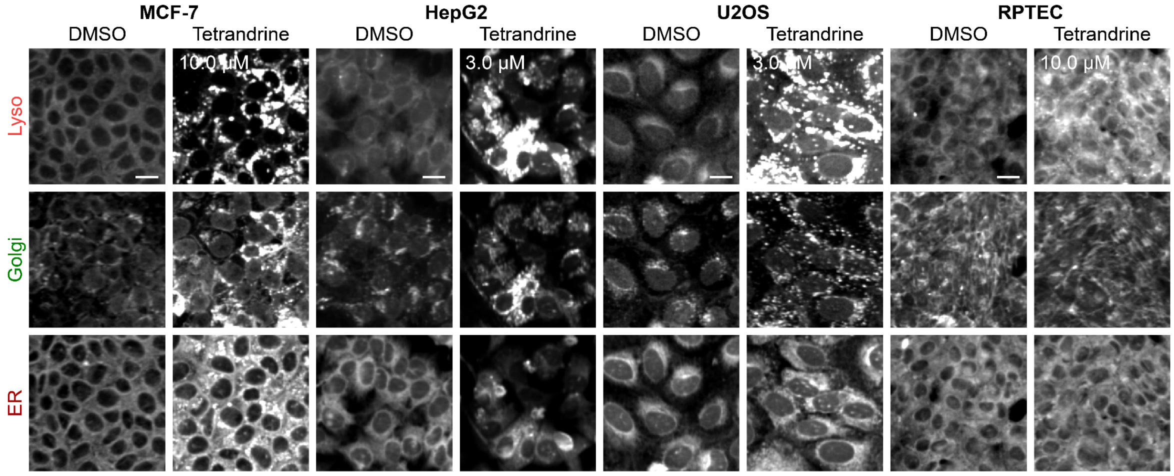

B BMC accumulation and magnitude plots (feature category-level) showing tetrandrine effects in different cell lines

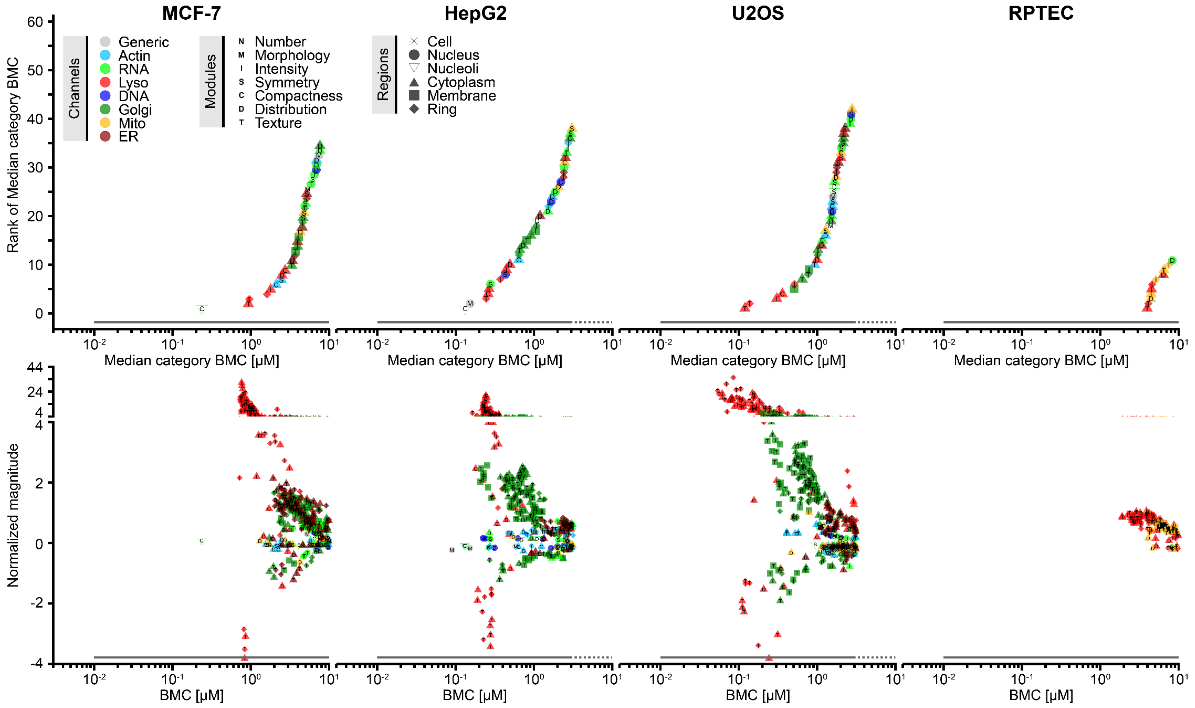

C Profile similarity plots (robust z-score, feature-level, **ex-/including** Lyso features, **with/without** feature selection) of **Cell Profiler**-based analyses of all reference compounds at highest non-cytototoxic concentrations in different cell lines

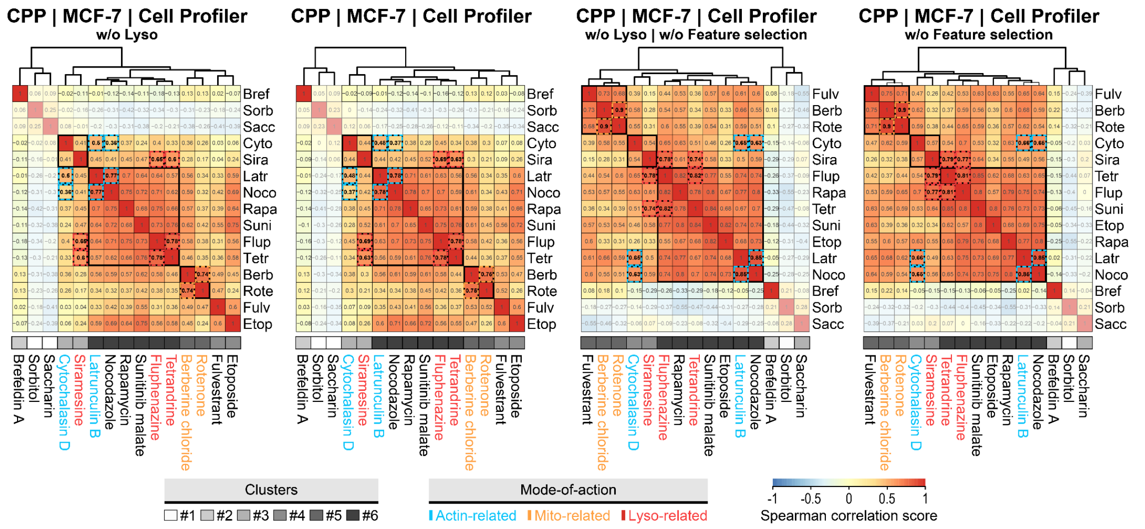

Supplementary Fig. 9. Elucidating compound effects on lysosomes using fluphenazine.

(A) Representative images (Lyso, Golgi, and ER channels) showing activities of fluphenazine on Lyso-, Golgi-, and ER-related features at a non-cytotoxic concentration compared to the DMSO solvent control across four different cell lines. N_{Biol} = 4. Scale bars = 20 µm.

(B) Corresponding BMC accumulation and magnitude plots showing fluphenazine effects on feature categories and single features across four different cell lines as described in Fig. 3D.

Source data are provided as a Source Data file.

Supplementary Figure 9

A Representative images of Lyso, Golgi, and ER phenotypes showing fluphenazine effects in different cell lines

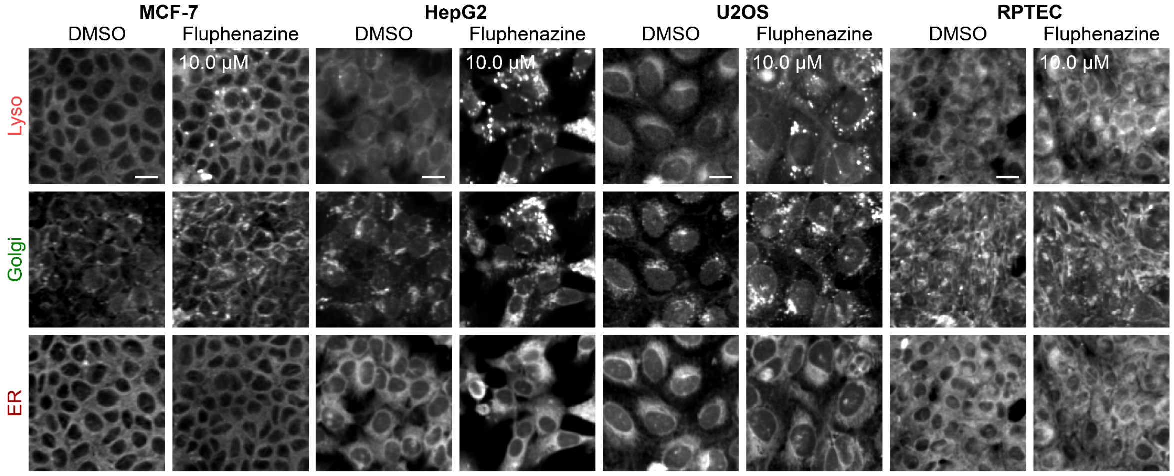

B BMC accumulation and magnitude plots (feature category-level) showing fluphenazine effects in different cell lines

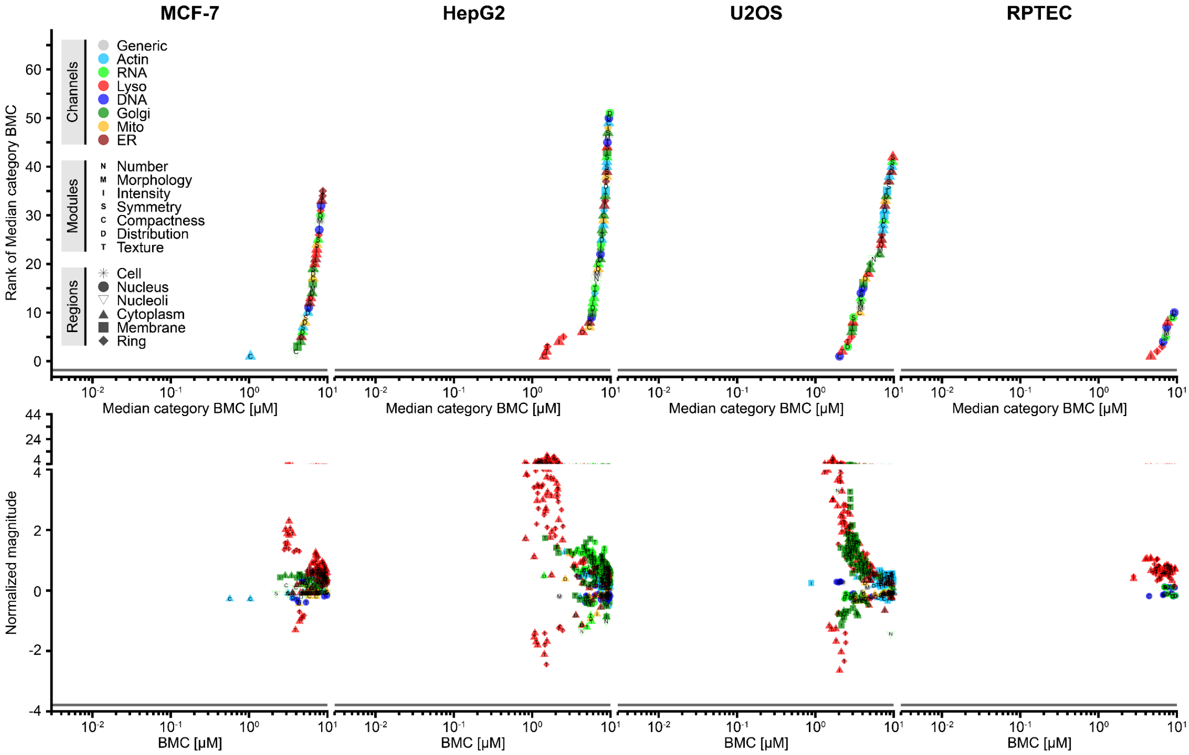

Supplementary Fig. 10. Elucidating compound effects on lysosomes using siramesine.

(A) Representative images (Lyso, Golgi, and ER channels) showing activities of siramesine on Lyso-, Golgi-, and ER-related features at a non-cytotoxic concentration compared to the DMSO solvent control across four different cell lines. N_{Biol} = 4. Scale bars = 20 µm.

(B) Corresponding BMC accumulation and magnitude plots showing siramesine effects on feature categories and single features across four different cell lines as described in Fig. 3D.

Source data are provided as a Source Data file.

Supplementary Figure 10

A Representative images of Lyso, Golgi, and ER phenotypes showing siramesine effects in different cell lines

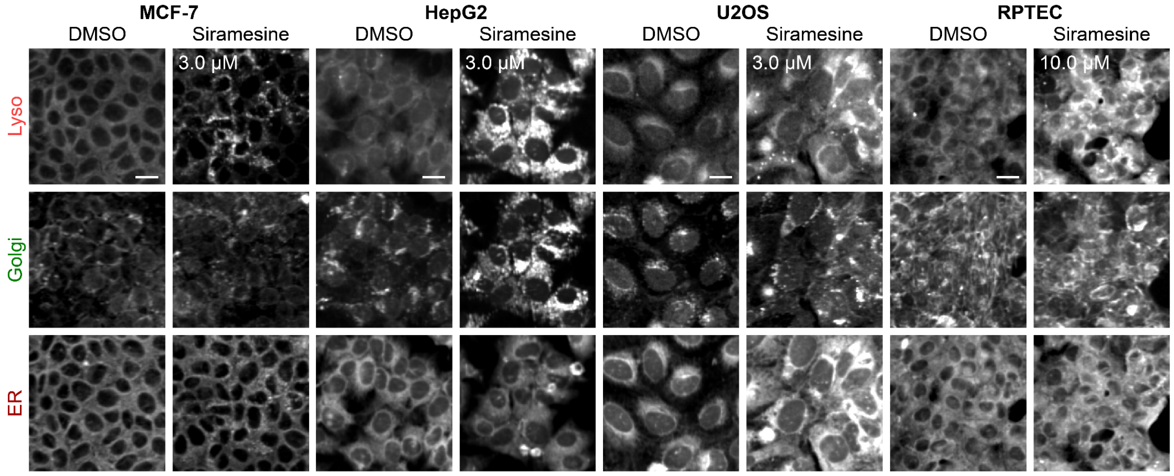

B BMC accumulation and magnitude plots (feature category-level) showing siramesine effects in different cell lines

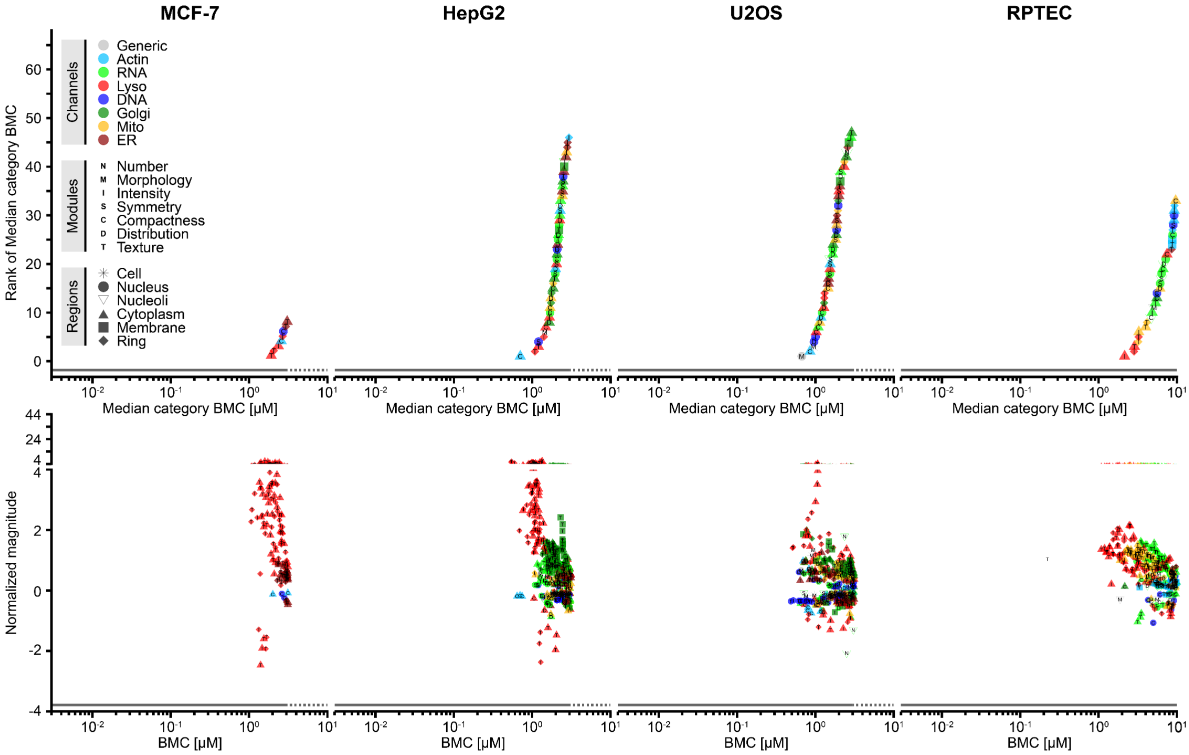

REFERENCES

1 Bray, M. A. *et al.* Cell Painting, a high-content image-based assay for morphological profiling using multiplexed fluorescent dyes. *Nat Protoc* **11**, 1757-1774 (2016). https://doi.org/10.1038/nprot.2016.105

2 Cimini, B. A. *et al.* Optimizing the Cell Painting assay for image-based profiling. *Nat Protoc* (2023). https://doi.org/10.1038/s41596-023-00840-9
