# Supplementary material for: Cell Painting PLUS: expanding the multiplexing capacity of Cell Painting-based phenotypic profiling using iterative staining-elution cycles
Source: Nat Commun. 2025 Apr 24;16:3857. doi: 10.1038/s41467-025-58765-8 (PMC12022024; doi:10.1038/s41467-025-58765-8)
Supplement: Supplementary file 2 — Reporting Summary [file 41467_2025_58765_MOESM2_ESM.pdf]

nature portfolio

Corresponding author(s): Sebastian Dunst & Shu Liu

Last updated by author(s): Mar 5, 2025

# Reporting Summary

Nature Portfolio wishes to improve the reproducibility of the work that we publish. This form provides structure for consistency and transparency in reporting. For further information on Nature Portfolio policies, see our Editorial Policies and the Editorial Policy Checklist.

## Statistics

For all statistical analyses, confirm that the following items are present in the figure legend, table legend, main text, or Methods section.

| n/a | Confirmed | |
|---|---|---|
| | x | The exact sample size (*n*) for each experimental group/condition, given as a discrete number and unit of measurement |
| | x | A statement on whether measurements were taken from distinct samples or whether the same sample was measured repeatedly |
| | x | The statistical test(s) used AND whether they are one- or two-sided<br>*Only common tests should be described solely by name; describe more complex techniques in the Methods section.* |
| x | | A description of all covariates tested |
| x | | A description of any assumptions or corrections, such as tests of normality and adjustment for multiple comparisons |
| | x | A full description of the statistical parameters including central tendency (e.g. means) or other basic estimates (e.g. regression coefficient) AND variation (e.g. standard deviation) or associated estimates of uncertainty (e.g. confidence intervals) |
| | x | For null hypothesis testing, the test statistic (e.g. *F*, *t*, *r*) with confidence intervals, effect sizes, degrees of freedom and *P* value noted<br>*Give P values as exact values whenever suitable.* |
| x | | For Bayesian analysis, information on the choice of priors and Markov chain Monte Carlo settings |
| x | | For hierarchical and complex designs, identification of the appropriate level for tests and full reporting of outcomes |
| | x | Estimates of effect sizes (e.g. Cohen's *d*, Pearson's *r*), indicating how they were calculated |

*Our web collection on statistics for biologists contains articles on many of the points above.*

## Software and code

Policy information about availability of computer code

| | |
|---|---|
| Data collection | As described in the methods section, all images were recorded at an Opera Phenix High-Content Screening System (Revvity Inc.) using the integrated Harmony software [v4.8]. Image analysis was performed using the commercial Harmony software [v4.8] (Revvity Inc.) or the open-source Cell Profiler [v4.2.4] software. When using the Harmony software for image analysis, an integrated application-specific building block was used for image registration. When using the Cell Profiler software for image analysis, image registration was performed using a customized Python software (4i stitcher). All customized image analysis pipelines and tools are provided in the Supplementary Data file and are publicly available in the Zenodo repository under accession code 14982928 [https://doi.org/10.5281/zenodo.14982928]. |
| Data analysis | As described in the methods section, data analysis and visualization was performed using the open-source process automation software KNIME [v4.7.1] and the R software [v4.2.2] . All customized data analysis pipelines are provided in the Supplementary Data file and are publicly available in the Zenodo repository under accession code 14982928 [https://doi.org/10.5281/zenodo.14982928]. Statistical analysis (Student's t-test/Pearson/Spearman correlation) and hierarchical clustering of robust z-score and BMC (log10 BMC) data were performed using the R software. |

For manuscripts utilizing custom algorithms or software that are central to the research but not yet described in published literature, software must be made available to editors and reviewers. We strongly encourage code deposition in a community repository (e.g. GitHub). See the Nature Portfolio guidelines for submitting code & software for further information.

# Data

Policy information about availability of data

All manuscripts must include a data availability statement. This statement should provide the following information, where applicable:

- Accession codes, unique identifiers, or web links for publicly available datasets
- A description of any restrictions on data availability
- For clinical datasets or third party data, please ensure that the statement adheres to our policy

The processed data (aggregated and processed profiles and BMC data) generated in this study are provided in the Supplementary Data/Source Data files and are publicly available in the Zenodo repository under accession code 14982928 [https://doi.org/10.5281/zenodo.14982928]. Due to their large size (25-50 GB per plate), the raw data (images and unprocessed profiles at the single-cell level) are available upon reasonable request directed to the corresponding authors.

## Research involving human participants, their data, or biological material

Policy information about studies with human participants or human data. See also policy information about sex, gender (identity/presentation), and sexual orientation and race, ethnicity and racism.

| | |
|---|---|
| Reporting on sex and gender | *Use the terms sex (biological attribute) and gender (shaped by social and cultural circumstances) carefully in order to avoid confusing both terms. Indicate if findings apply to only one sex or gender; describe whether sex and gender were considered in study design; whether sex and/or gender was determined based on self-reporting or assigned and methods used.*<br>*Provide in the source data disaggregated sex and gender data, where this information has been collected, and if consent has been obtained for sharing of individual-level data; provide overall numbers in this Reporting Summary. Please state if this information has not been collected.*<br>*Report sex- and gender-based analyses where performed, justify reasons for lack of sex- and gender-based analysis.* |
| Reporting on race, ethnicity, or other socially relevant groupings | *Please specify the socially constructed or socially relevant categorization variable(s) used in your manuscript and explain why they were used. Please note that such variables should not be used as proxies for other socially constructed/relevant variables (for example, race or ethnicity should not be used as a proxy for socioeconomic status).*<br>*Provide clear definitions of the relevant terms used, how they were provided (by the participants/respondents, the researchers, or third parties), and the method(s) used to classify people into the different categories (e.g. self-report, census or administrative data, social media data, etc.)*<br>*Please provide details about how you controlled for confounding variables in your analyses.* |
| Population characteristics | *Describe the covariate-relevant population characteristics of the human research participants (e.g. age, genotypic information, past and current diagnosis and treatment categories). If you filled out the behavioural & social sciences study design questions and have nothing to add here, write "See above."* |
| Recruitment | *Describe how participants were recruited. Outline any potential self-selection bias or other biases that may be present and how these are likely to impact results.* |
| Ethics oversight | *Identify the organization(s) that approved the study protocol.* |

Note that full information on the approval of the study protocol must also be provided in the manuscript.

# Field-specific reporting

Please select the one below that is the best fit for your research. If you are not sure, read the appropriate sections before making your selection.

[x] Life sciences   [ ] Behavioural & social sciences   [ ] Ecological, evolutionary & environmental sciences

For a reference copy of the document with all sections, see nature.com/documents/nr-reporting-summary-flat.pdf

# Life sciences study design

All studies must disclose on these points even when the disclosure is negative.

| | |
|---|---|
| Sample size | For all quantitative in vitro data described in the article, the sample size (N) was reported as an exact number in the corresponding figure legends. |
| Data exclusions | Border cells touching the edge of the image were excluded from further analysis as the provide only incomplete phenotypic profiles. During image registration using the 4i stitcher Python software, registrations with a cross-correlation coefficient below 0.8 underwent manual verification for accuracy, and those below 0.5 were excluded. Compound solubility issues after cell exposure were observed for the highest concentration of berberine chloride. The corresponding wells were therefore excluded from all analyses. The weaker activity profile of tetrandrine at the highest tested concentration across all cell lines also indicated potential compound solubility issues. The resulting lower exposure levels were also reflected in the corresponding relative cell number plots, consequently leading to the exclusion of this concentration from subsequent analysis. BMCs were only determined from at least four non-cytotoxic concentrations of at least three biological replicates to ensure high quality BMC curve fitting in accordance with published guidance on benchmark dose modeling in toxicology. Feature categories for which less than 30% of the included features showed a response were excluded from the BMC accumulation and magnitude plots to maintain most relevant feature groups. For generation of Proportion BMC profiles, features for which |

| | |
|---|---|
| | no BMC could be determined were adjusted to a modified BMC corresponding to the maximal tested compound concentration (100 µM) to enable comparison between all compounds. |
| Replication | This phenotypic screening study was performed by running the CPP assay on the reference compound plate in three technical and four biological replicates for each compound and concentration tested. |
| Randomization | As described in the article and its Supplementary Information, reference compounds were distributed across the reference compound plate in three blocks of technical replicates and alternating order to evaluate potential plate position effects. |
| Blinding | All quantitative in vitro data were collected and analyzed using automated workflows. |

# Reporting for specific materials, systems and methods

We require information from authors about some types of materials, experimental systems and methods used in many studies. Here, indicate whether each material, system or method listed is relevant to your study. If you are not sure if a list item applies to your research, read the appropriate section before selecting a response.

## Materials & experimental systems

| n/a | Involved in the study | |
|---|---|---|
| ☐ | ☒ | Antibodies |
| ☐ | ☒ | Eukaryotic cell lines |
| ☒ | ☐ | Palaeontology and archaeology |
| ☒ | ☐ | Animals and other organisms |
| ☒ | ☐ | Clinical data |
| ☒ | ☐ | Dual use research of concern |
| ☒ | ☐ | Plants |

## Methods

| n/a | Involved in the study | |
|---|---|---|
| ☒ | ☐ | ChIP-seq |
| ☒ | ☐ | Flow cytometry |
| ☒ | ☐ | MRI-based neuroimaging |

## Antibodies

Antibodies used

Antibodies:
Rabbit anti-Actin directly-conjugated to Alexa Fluor 555, Abcam, Cambridge, UK, No.ab208080, Clone EPR16769

Dyes:
Concanavalin A Alexa Fluor 488 Conjugate, Invitrogen, No.C11252
Concanavalin A Alexa Fluor 647 Conjugate, Invitrogen, No.C21421
Hoechst33342, Invitrogen, No.H3570
LysoTracker: Cell Navigator Lysosome Staining Kit *NIR Fluorescence*, AAT Bioquest, No.22652
MitoTracker Orange CMTMROS, Invitrogen, No.M7510
MitoTracker Deep Red FM - Special Packaging, Invitrogen, No.M22426
Phalloidin Alexa Fluor 568, Invitrogen, No.A12380
Phalloidin Alexa Fluor 647, Invitrogen, No.A22287
Phalloidin Alexa Fluor Plus 405, Invitrogen, No.A30104
Phalloidin FITC, Sigma-Aldrich, No.P5282
Phalloidin-iFluor647 conjugate, Abcam, No.ab176759
SYTO 14 Green Fluorescent Nucleic Acid Stain, Invitrogen, No.S7576
Wheat Germ Agglutinin (WGA), Alexa Fluor 488 Conjugate, Invitrogen, No.W11261
Wheat Germ Agglutinin Alexa Fluor 555 Conjugate, Invitrogen, No.W32464

Validation

Antibodies:
Rabbit anti-Actin directly-conjugated to Alexa Fluor 555 (No.ab208080, Clone EPR16769) is a tested rabbit recombinant monoclonal antibody directed against human alpha skeletal muscle Actin.

https://www.abcam.com/en-us/products/primary-antibodies/actin-antibody-epr16769-ab179467

--> The manufacturer website provides the following information:
Anti-Actin antibody [EPR16769] ab179467 is a rabbit monoclonal antibody that is used in Actin western blotting, IHC, immunofluorescence and flow cytometry. Suitable for human, mouse and rat samples.
- Recombinant format for unrivaled batch-batch consistency: no need for same-lot requests
- Specificity and sensitivity confirmed in IHC with multi-tissue microarray (TMA) validation
- Antibody clone EPR16769 is cited in over 520 publications
- One antibody for all your Actin staining

--> According to the manufacturer website, the antibody has been used in > 400 publications

## Eukaryotic cell lines

Policy information about cell lines and Sex and Gender in Research

| | |
|---|---|
| Cell line source(s) | The MCF-7/vBOS cell line (female) was obtained from own lab and is described in https://doi.org/10.1016/j.isci.2020.101683 and https://doi.org/10.1016/j.envint.2021.106411.<br>The U2OS (HTB-96) cell line (female) was obtained from ATCC.<br>The HepG2 (ACC 180) cell line (male) was obtained from DMSZ.<br>The RPTEC-TERT1 cell line (male) was obtained from Bob van de Water (Leiden University, The Netherlands) and is described in https://doi.org/10.1152/ajprenal.90405.2008. |
| Authentication | The identities of all cell lines were verified using the Eurofins Genomics Cell Line Authentication service (Eurofins Genomics, Ebersberg, Germany). |
| Mycoplasma contamination | The cell lines were regularly tested using the Eurofins Genomics mycoplasma test service (Eurofins Genomics, Ebersberg, Germany). All cell lines used did not show mycoplamsa contamination. |
| Commonly misidentified lines (See ICLAC register) | No commonly misidentified cell lines were used. |

## Plants

| | |
|---|---|
| Seed stocks | *Report on the source of all seed stocks or other plant material used. If applicable, state the seed stock centre and catalogue number. If plant specimens were collected from the field, describe the collection location, date and sampling procedures.* |
| Novel plant genotypes | *Describe the methods by which all novel plant genotypes were produced. This includes those generated by transgenic approaches, gene editing, chemical/radiation-based mutagenesis and hybridization. For transgenic lines, describe the transformation method, the number of independent lines analyzed and the generation upon which experiments were performed. For gene-edited lines, describe the editor used, the endogenous sequence targeted for editing, the targeting guide RNA sequence (if applicable) and how the editor was applied.* |
| Authentication | *Describe any authentication procedures for each seed stock used or novel genotype generated. Describe any experiments used to assess the effect of a mutation and, where applicable, how potential secondary effects (e.g. second site T-DNA insertions, mosiacism, off-target gene editing) were examined.* |
