# Supplementary material for: Cell Painting PLUS: expanding the multiplexing capacity of Cell Painting-based phenotypic profiling using iterative staining-elution cycles
Source: Nat Commun. 2025 Apr 24;16:3857. doi: 10.1038/s41467-025-58765-8 (PMC12022024; doi:10.1038/s41467-025-58765-8)
Supplement: Supplementary file 3 — Supplementary Data [file 41467_2025_58765_MOESM3_ESM.zip › Supplementary Data/Supplementary Data_23_FigS5_BMCBarPlots_CPP_CP_ActinGolgi_CytoplasmRingMembrane.pdf]

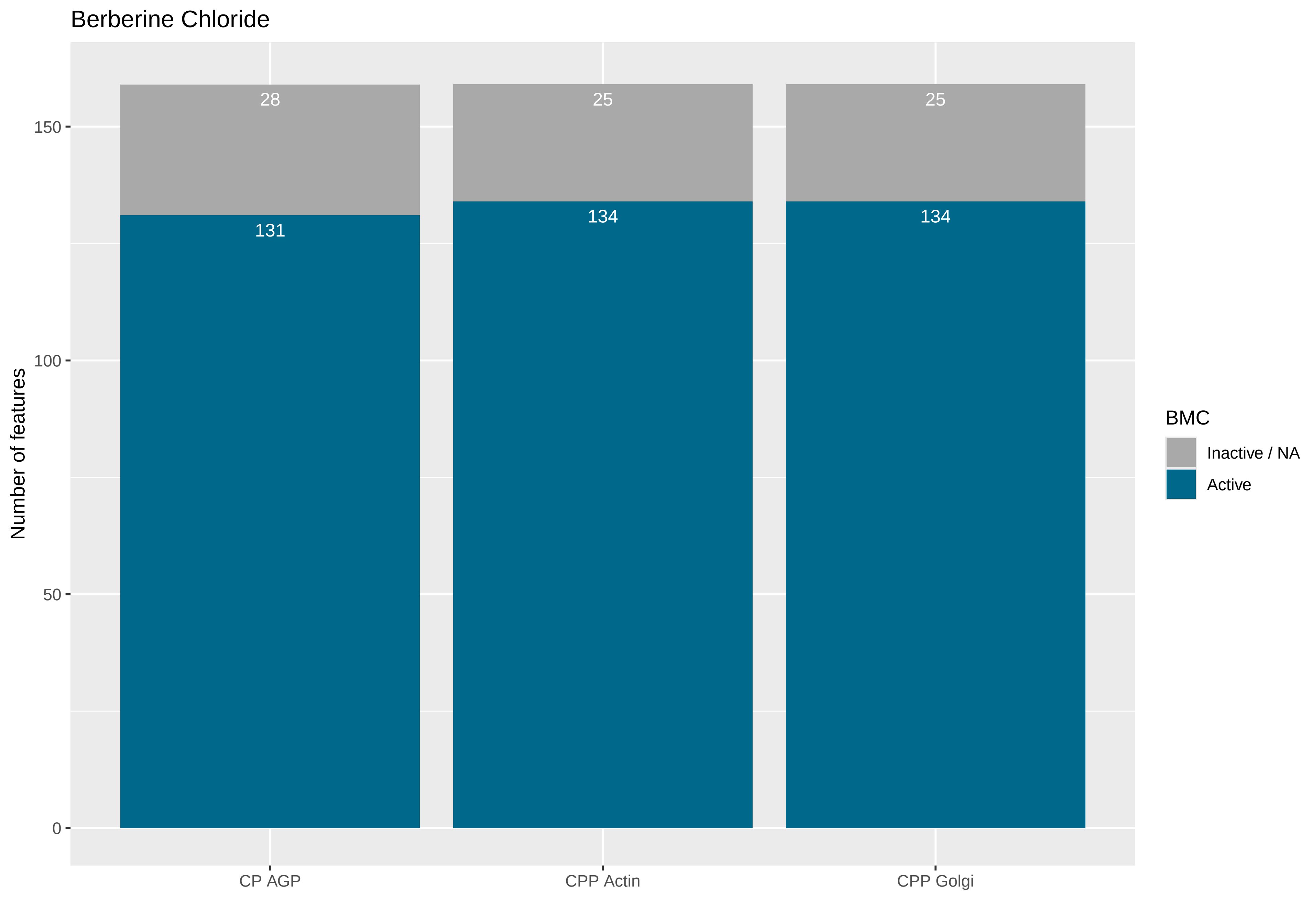

Berberine Chloride
Number of features
28
25
25
131
134
134
0
50
100
150
CP AGP
CPP Actin
CPP Golgi
BMC
Inactive / NA
Active

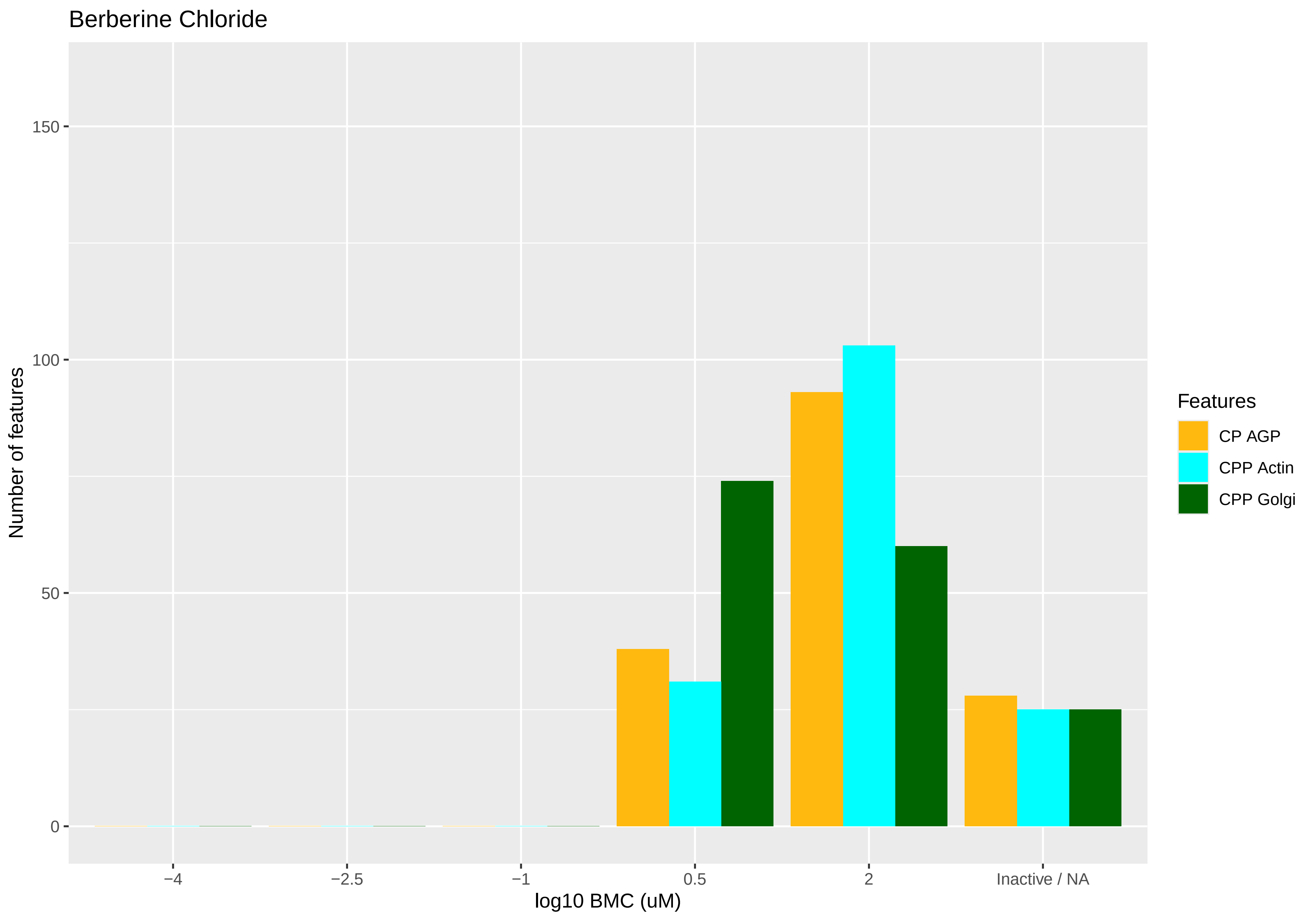

Berberine Chloride
Number of features
0
50
100
150
−4
−2.5
−1
0.5
2
Inactive / NA
log10 BMC (uM)
Features
CP AGP
CPP Actin
CPP Golgi

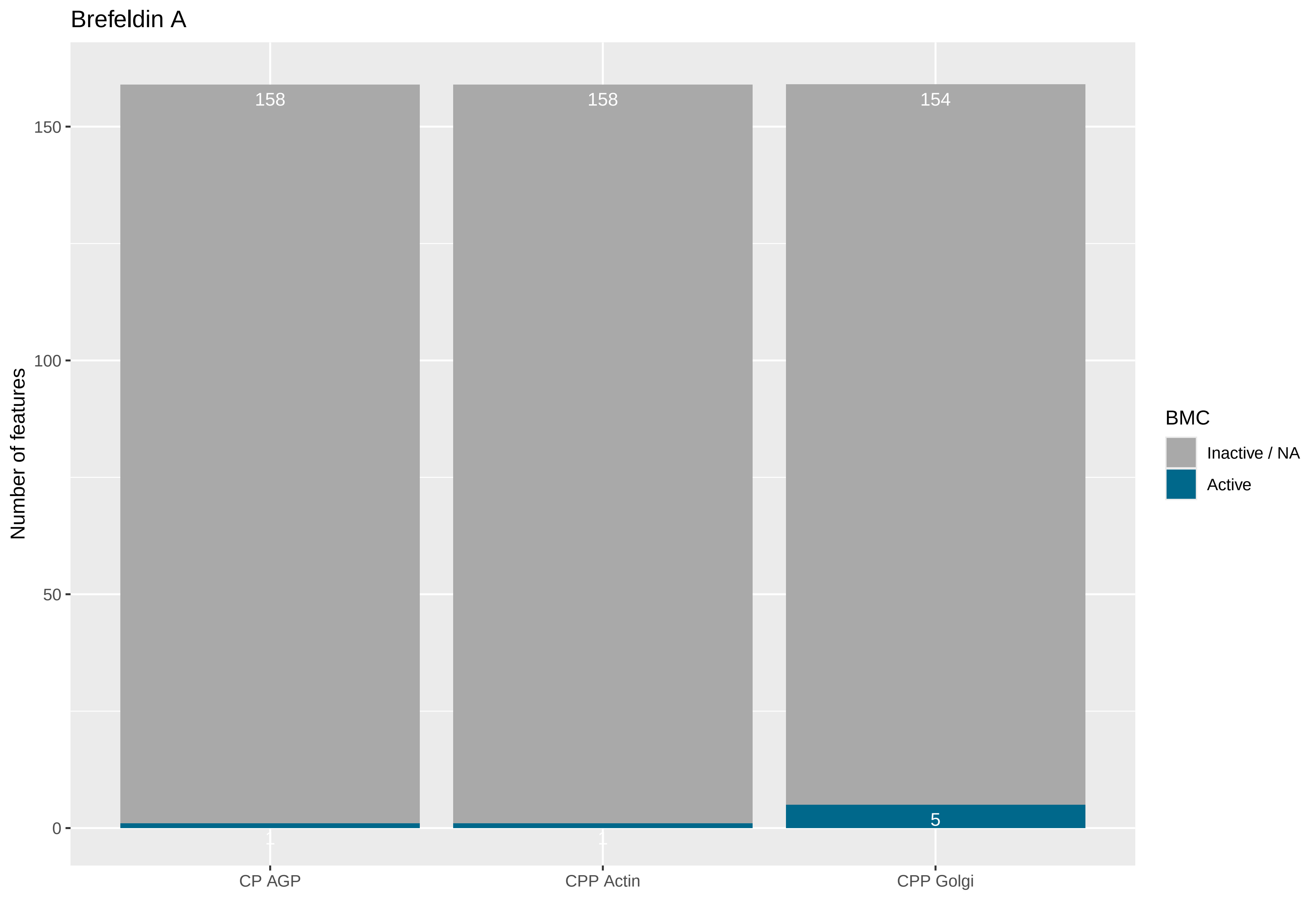

Brefeldin A
Number of features
158
158
154
1
1
5
0
50
100
150
CP AGP
CPP Actin
CPP Golgi
BMC
Inactive / NA
Active

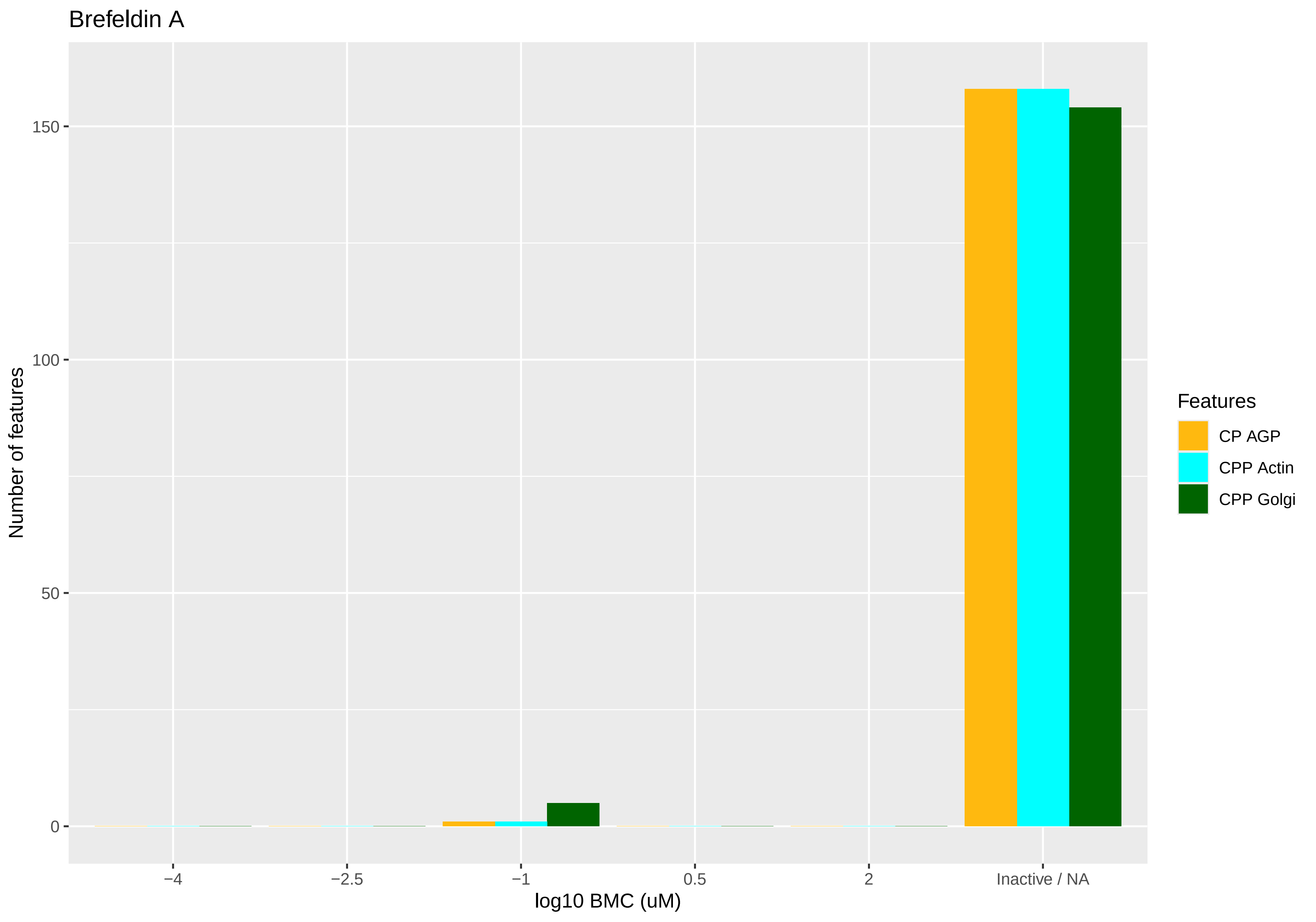

Brefeldin A
Number of features
0
50
100
150
−4
−2.5
−1
0.5
2
Inactive / NA
log10 BMC (uM)
Features
CP AGP
CPP Actin
CPP Golgi

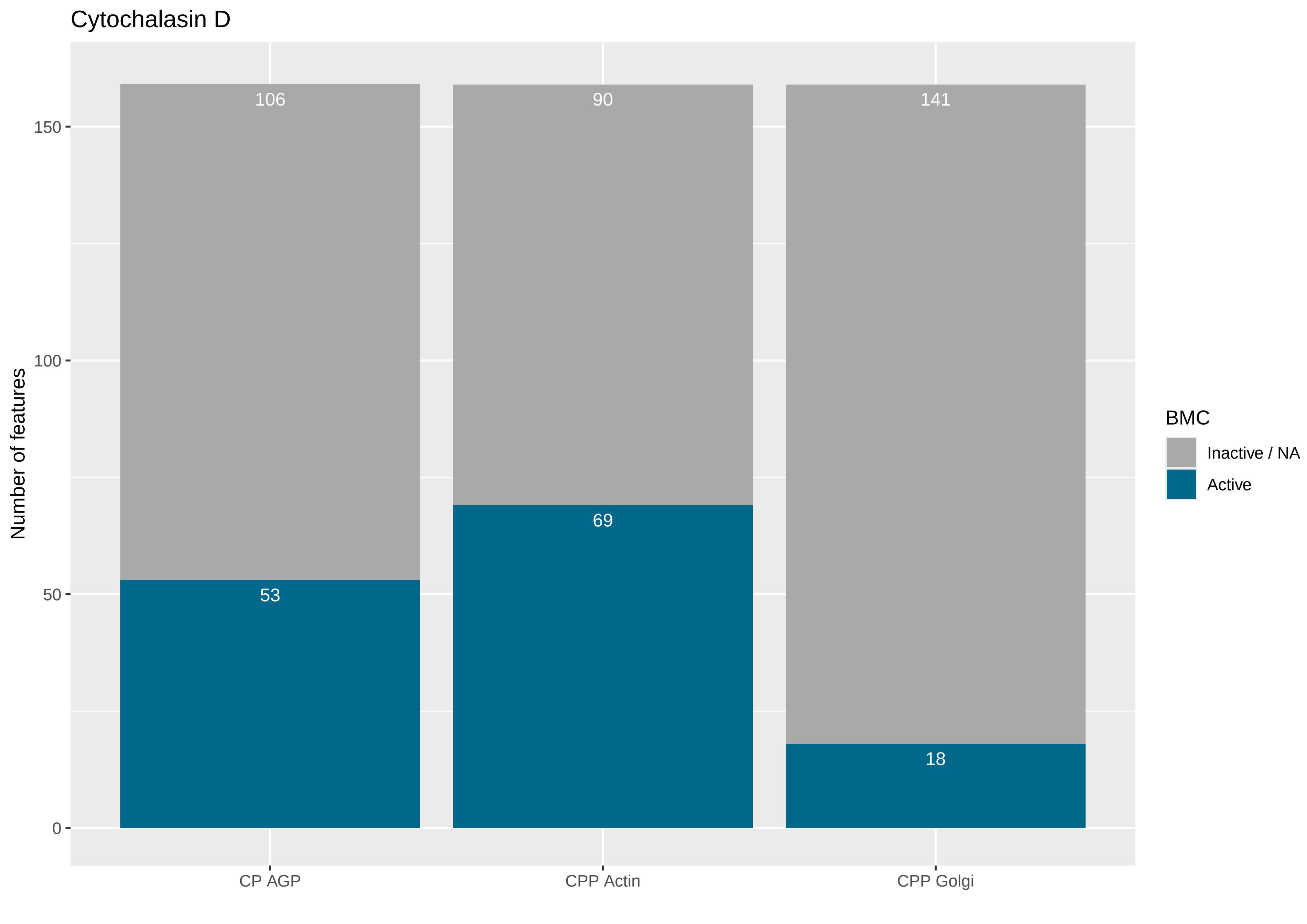
Cytochalasin D
Number of features
150
100
50
0
106
90
141
53
69
18
CP AGP
CPP Actin
CPP Golgi
BMC
Inactive / NA
Active

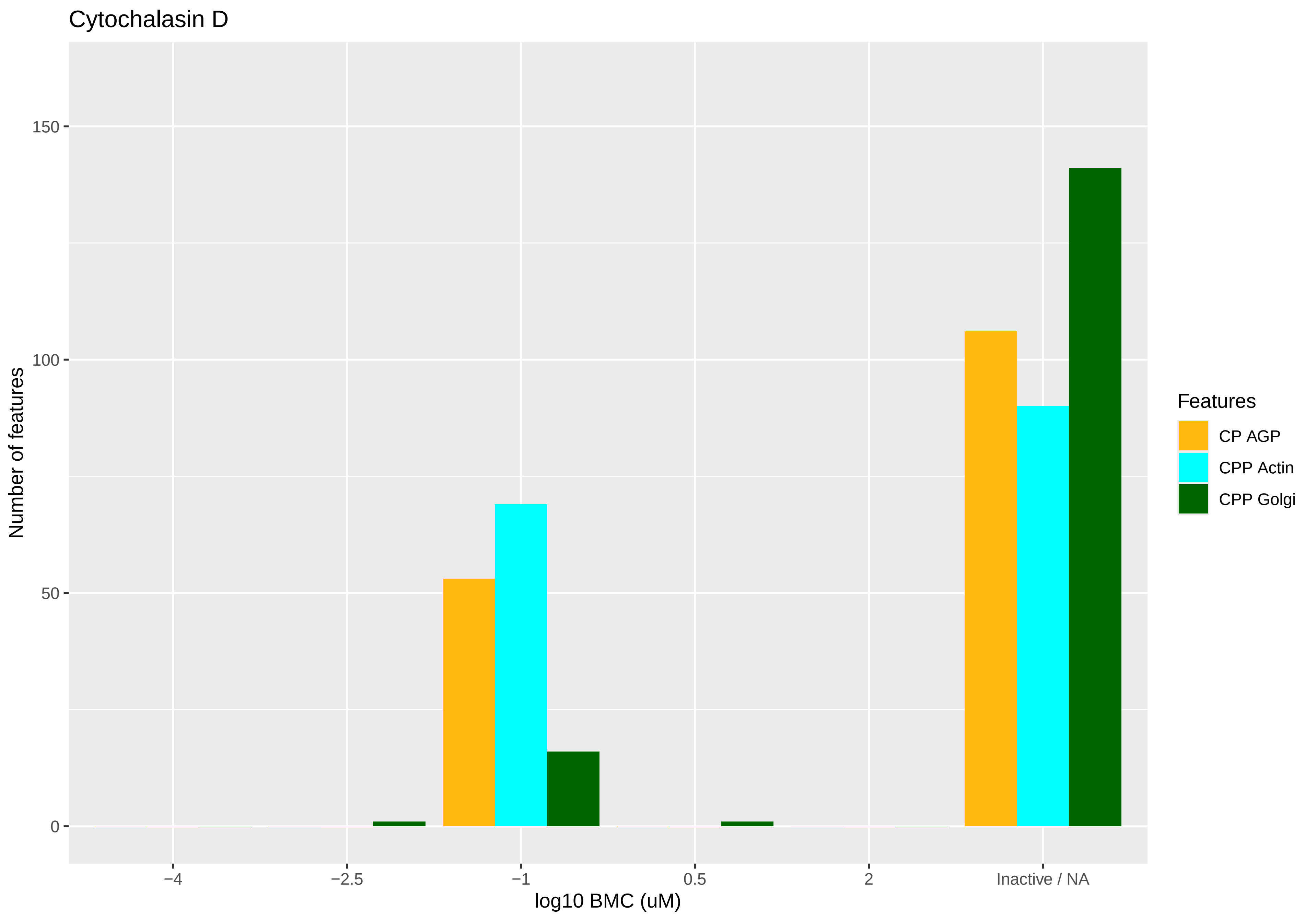
Cytochalasin D
Number of features
150
100
50
0
−4
−2.5
−1
0.5
2
Inactive / NA
log10 BMC (uM)
Features
CP AGP
CPP Actin
CPP Golgi

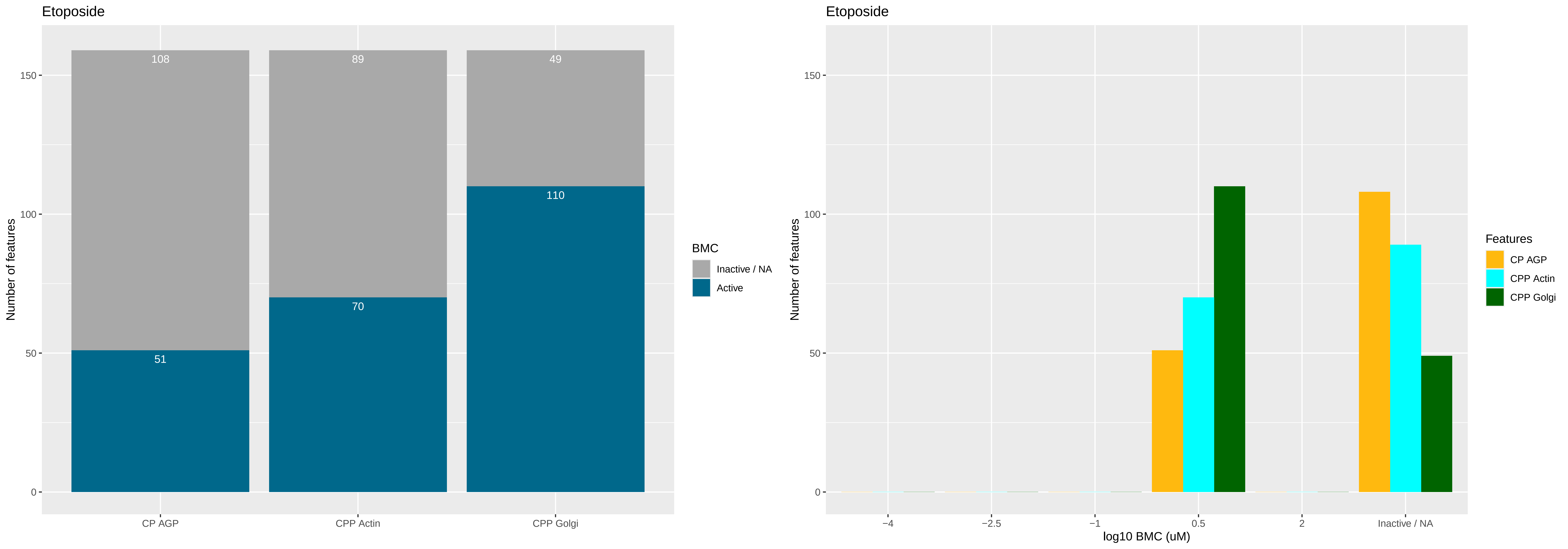
Etoposide
108
89
49
110
70
51
Number of features
150
100
50
0
CP AGP
CPP Actin
CPP Golgi
BMC
Inactive / NA
Active
Etoposide
Number of features
150
100
50
0
−4
−2.5
−1
0.5
2
Inactive / NA
log10 BMC (uM)
Features
CP AGP
CPP Actin
CPP Golgi

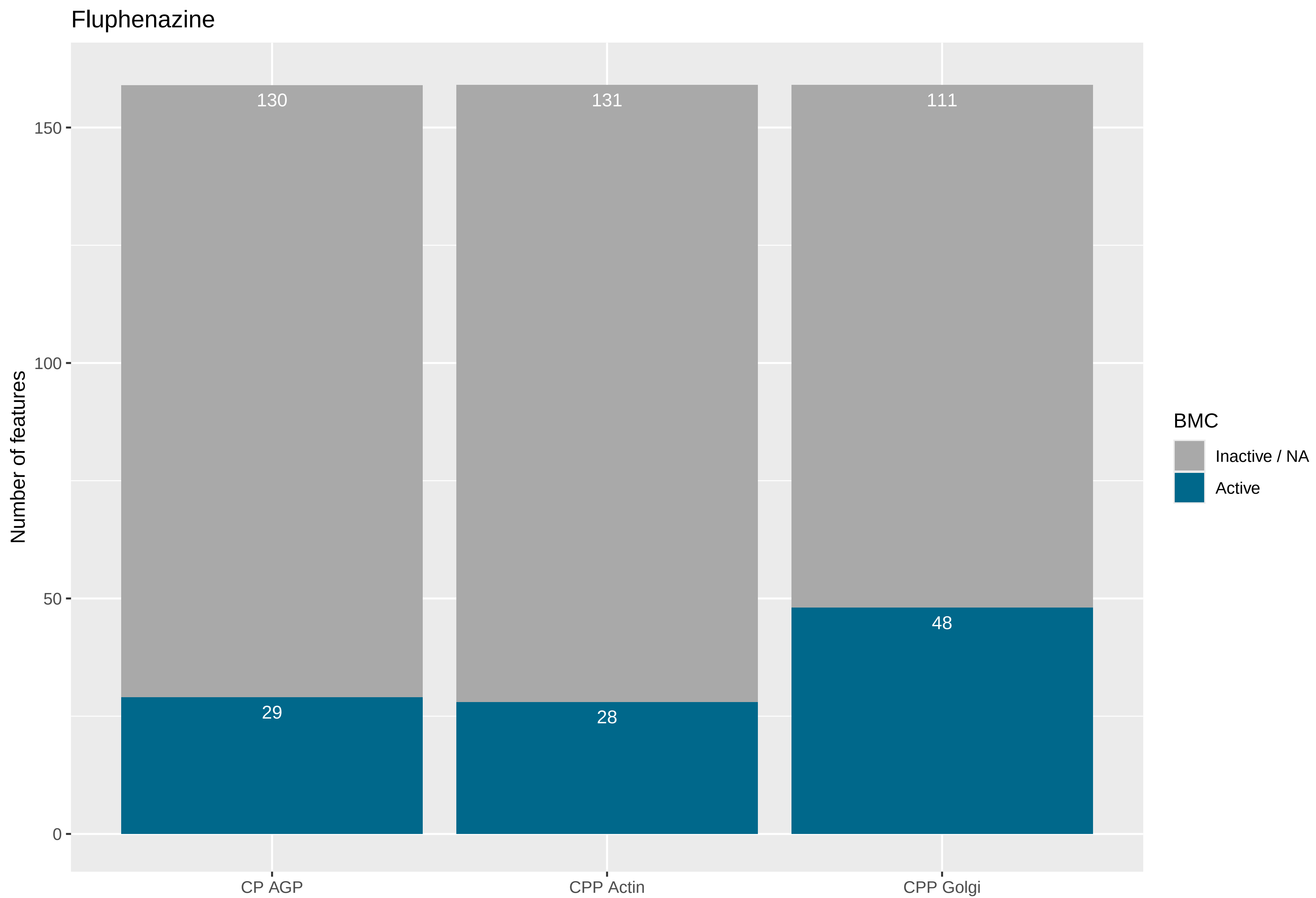

Fluphenazine
130
131
111
29
28
48
Number of features
150
100
50
0
CP AGP
CPP Actin
CPP Golgi
BMC
Inactive / NA
Active

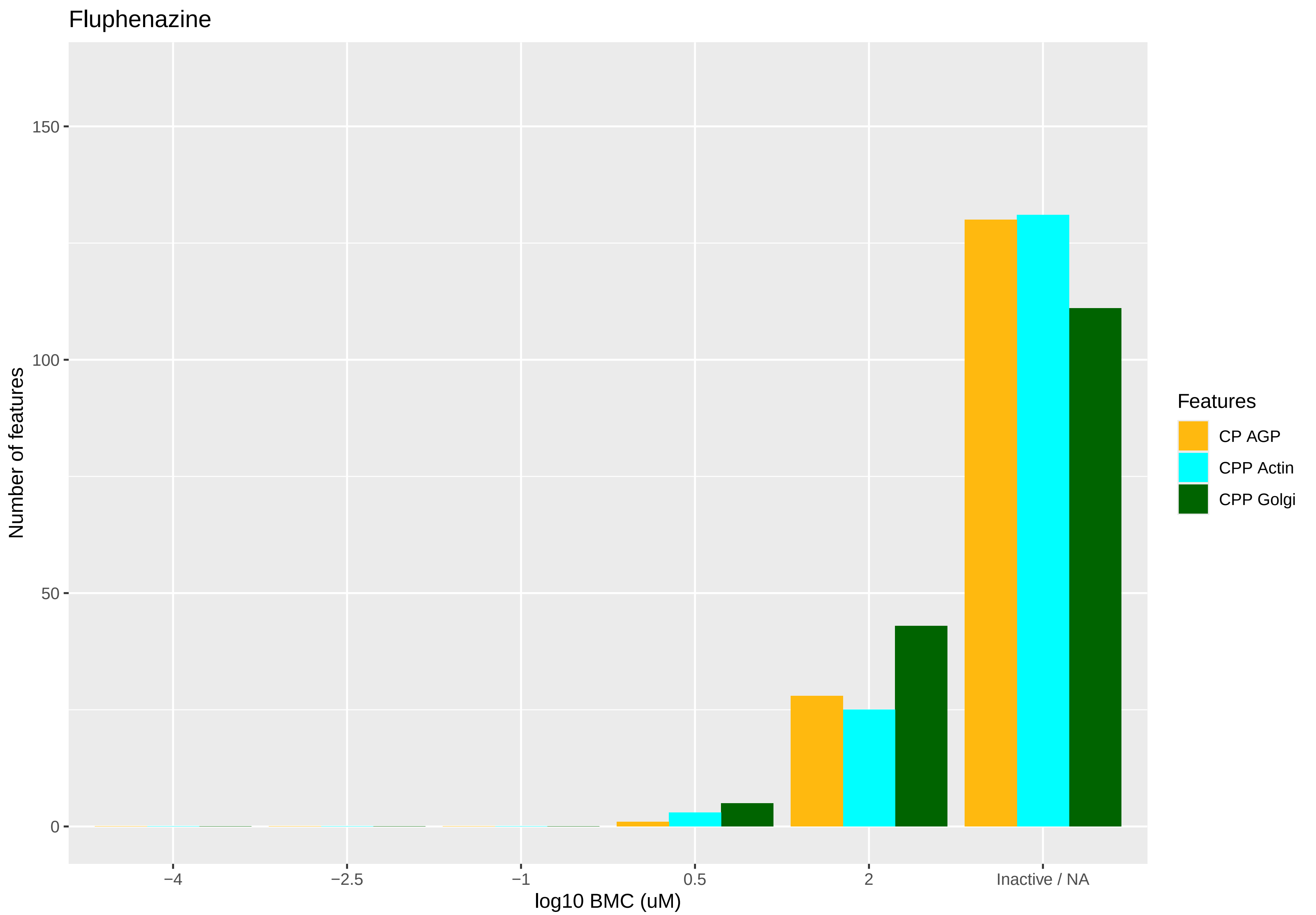

Fluphenazine
Number of features
150
100
50
0
−4
−2.5
−1
0.5
2
Inactive / NA
log10 BMC (uM)
Features
CP AGP
CPP Actin
CPP Golgi

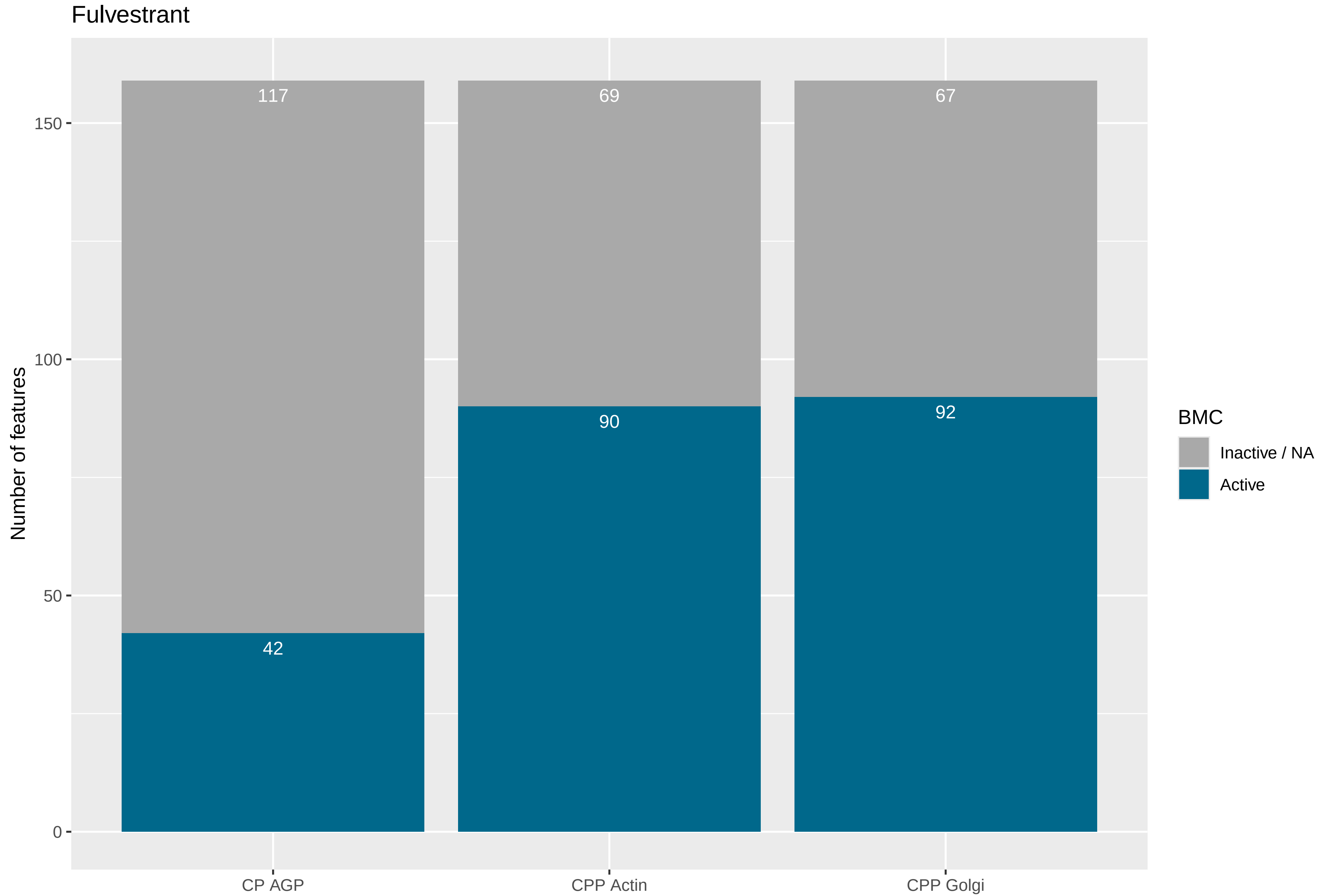
Fulvestrant
Number of features
117
69
67
90
92
42
150
100
50
0
CP AGP
CPP Actin
CPP Golgi
BMC
Inactive / NA
Active

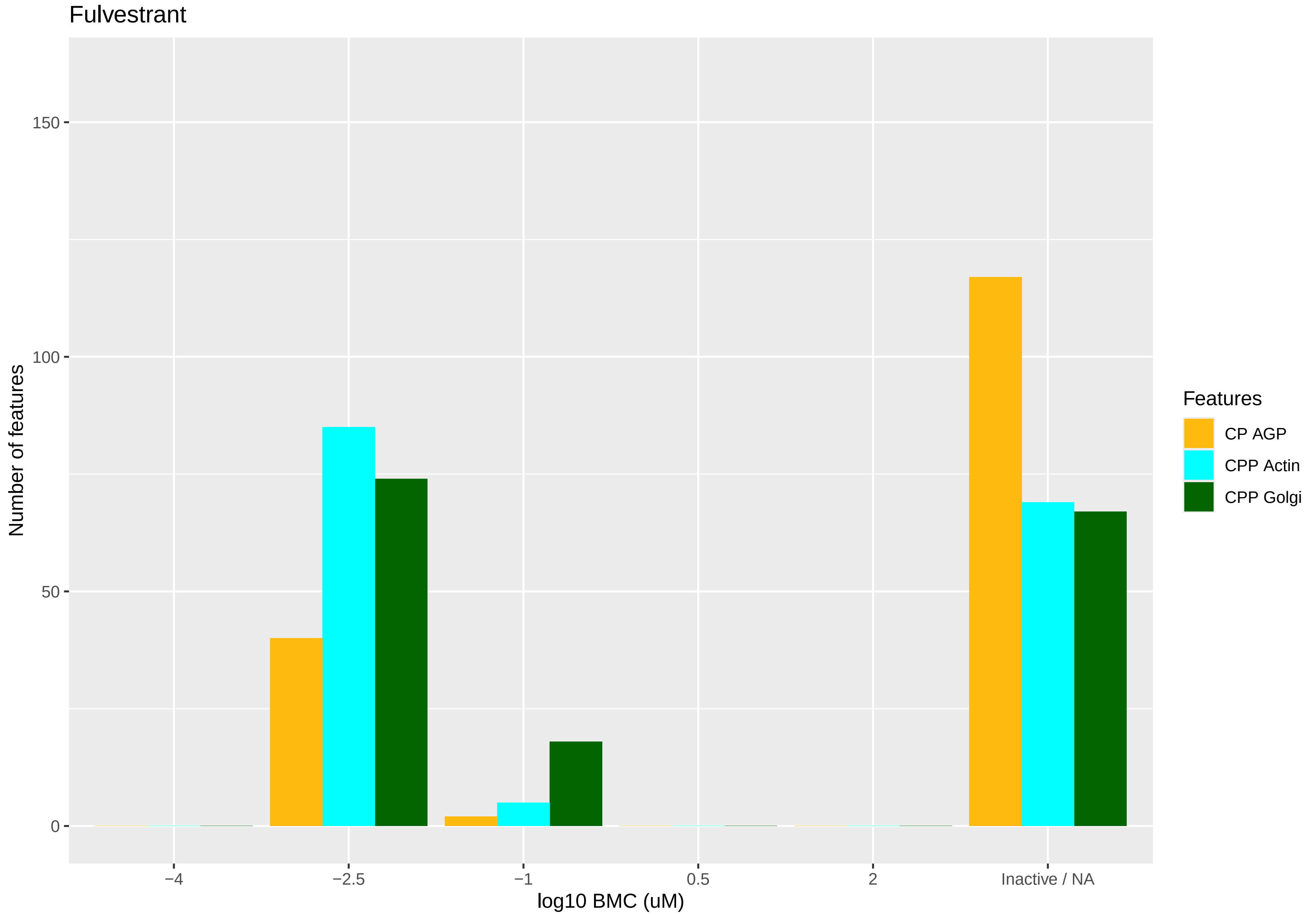
Fulvestrant
Number of features
150
100
50
0
−4
−2.5
−1
0.5
2
Inactive / NA
log10 BMC (uM)
Features
CP AGP
CPP Actin
CPP Golgi

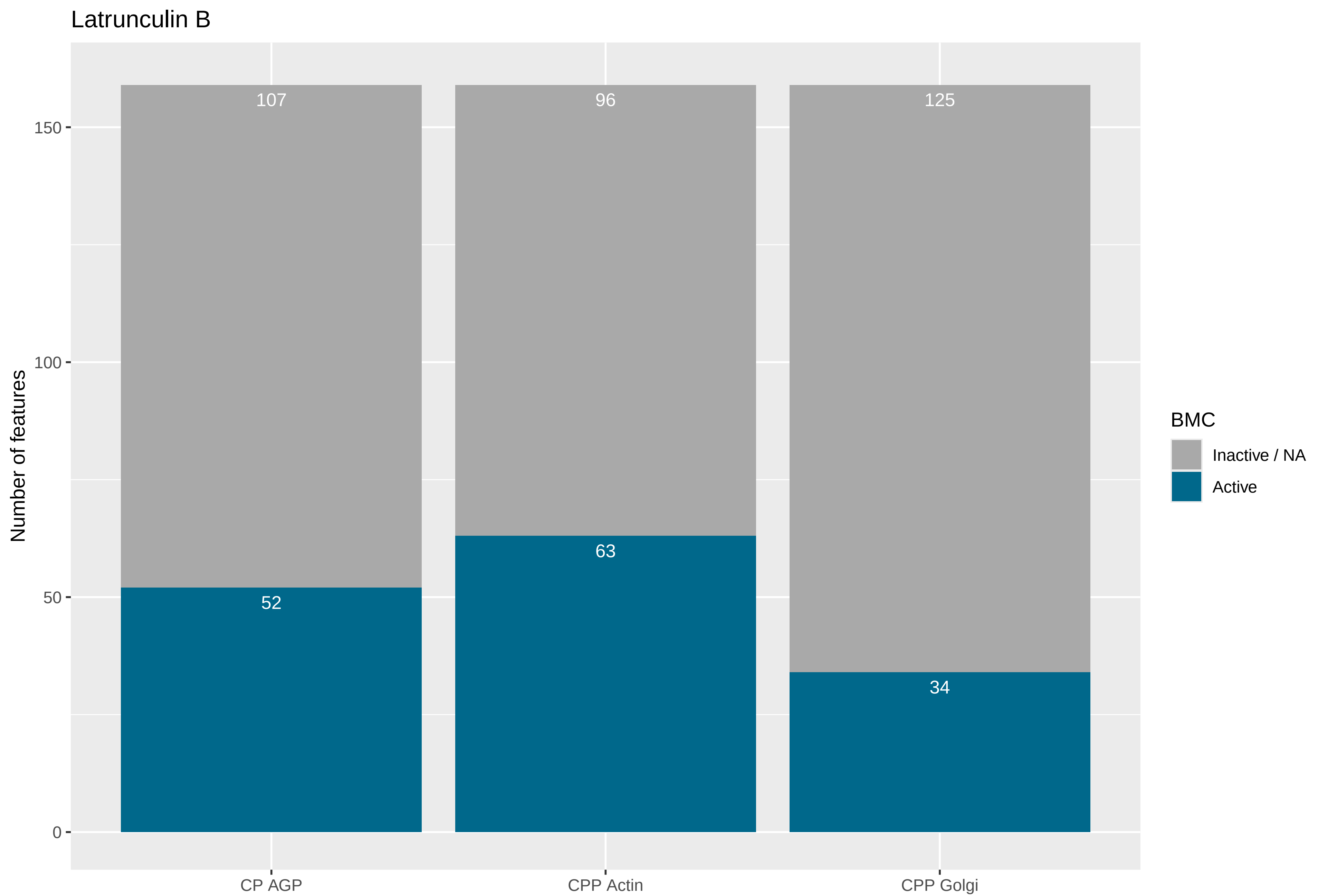

Latrunculin B
Number of features
BMC
Inactive / NA
Active
107
96
125
52
63
34
0
50
100
150
CP AGP
CPP Actin
CPP Golgi

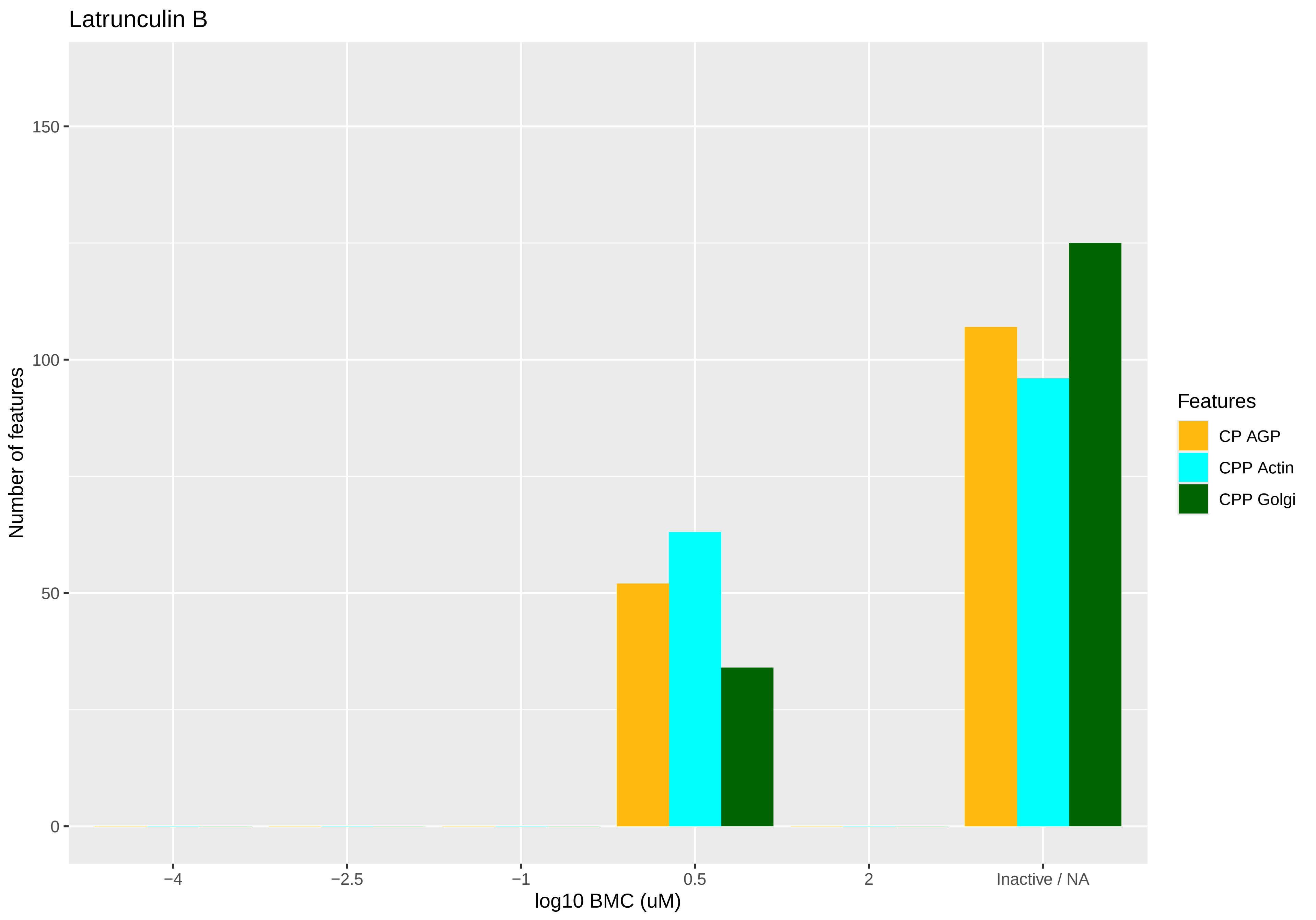

Latrunculin B
Number of features
log10 BMC (uM)
Features
CP AGP
CPP Actin
CPP Golgi
0
50
100
150
−4
−2.5
−1
0.5
2
Inactive / NA

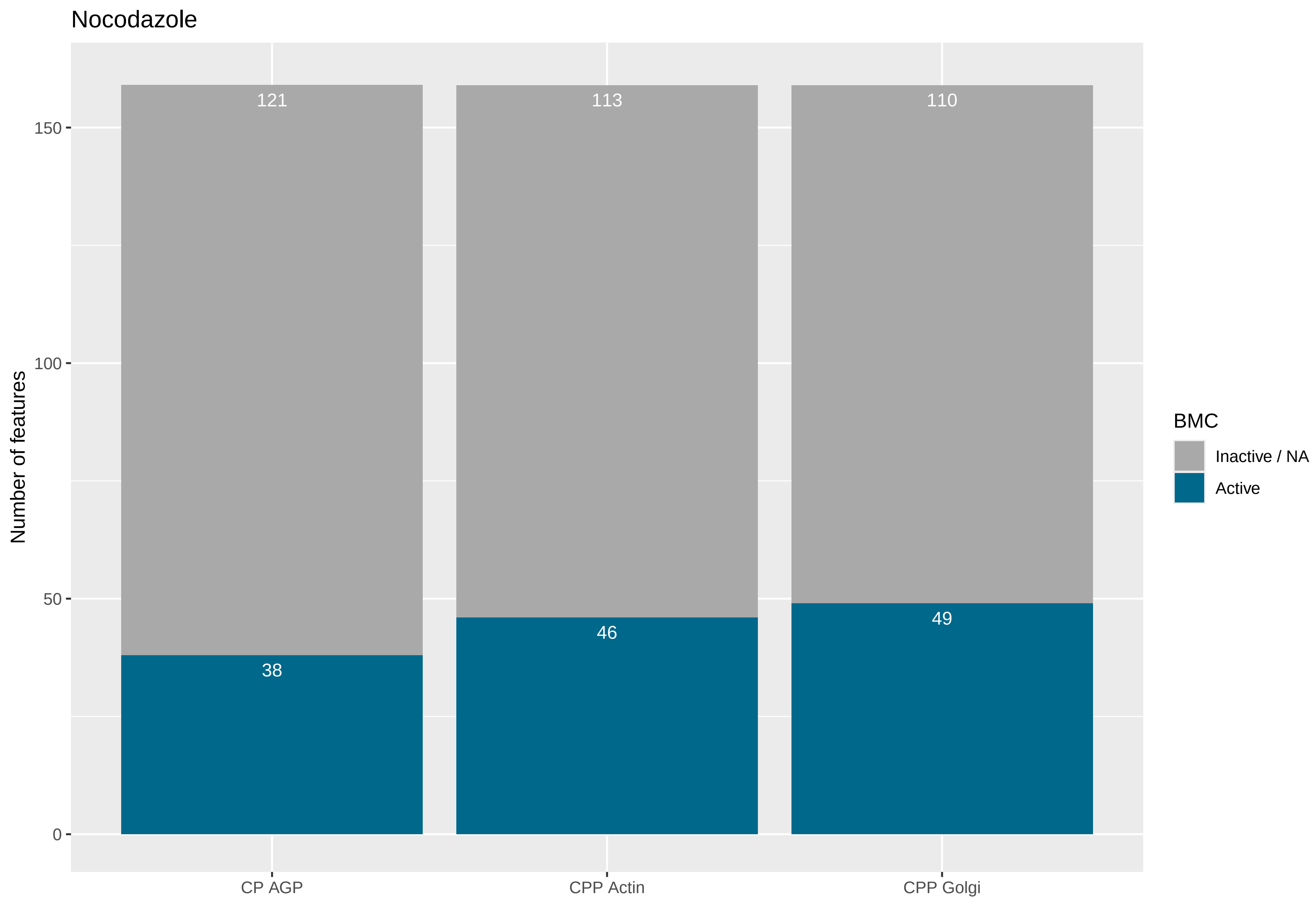
Nocodazole
Number of features
150
100
50
0
121
113
110
46
49
38
CP AGP
CPP Actin
CPP Golgi
BMC
Inactive / NA
Active

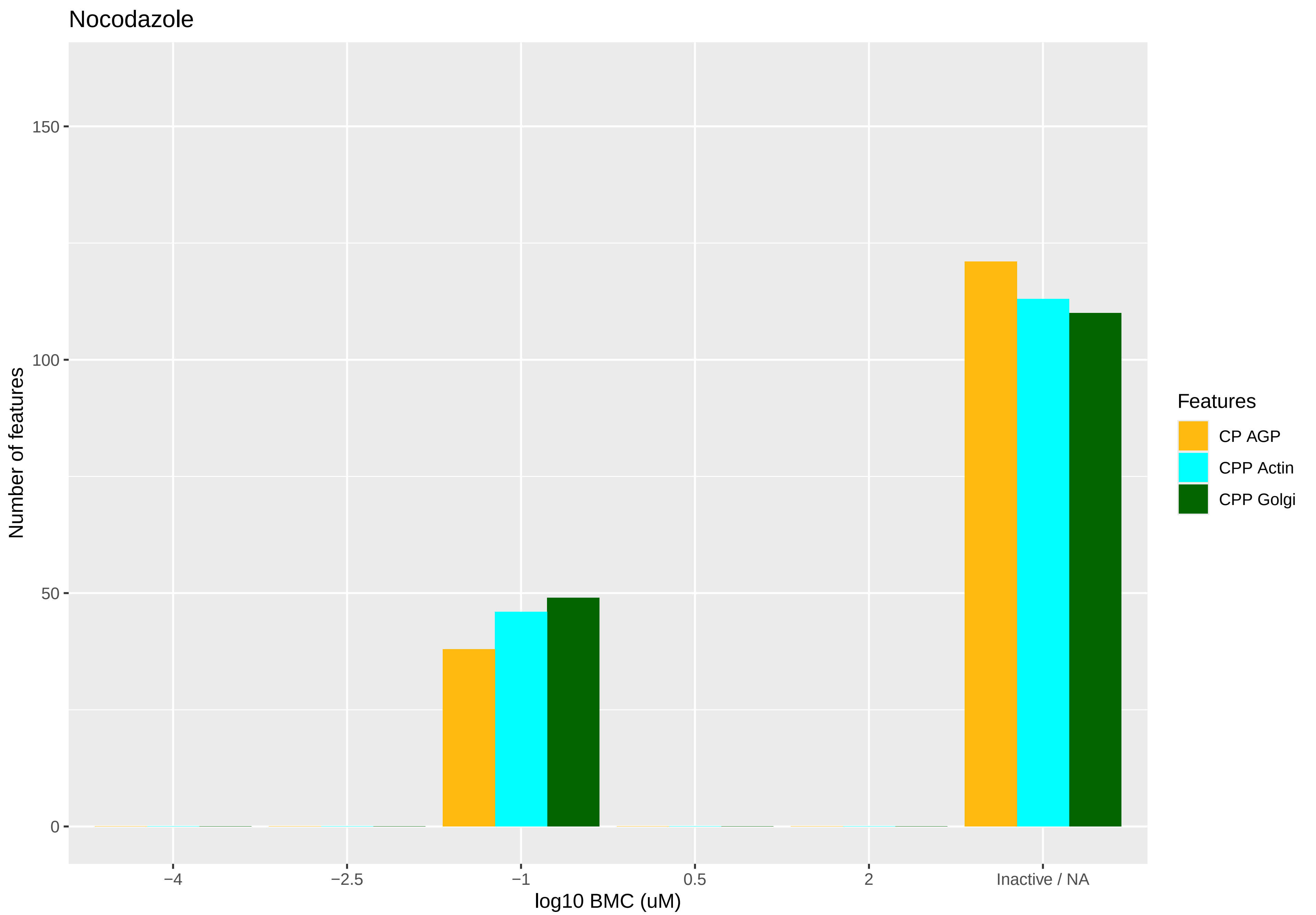
Nocodazole
Number of features
150
100
50
0
−4
−2.5
−1
0.5
2
Inactive / NA
log10 BMC (uM)
Features
CP AGP
CPP Actin
CPP Golgi

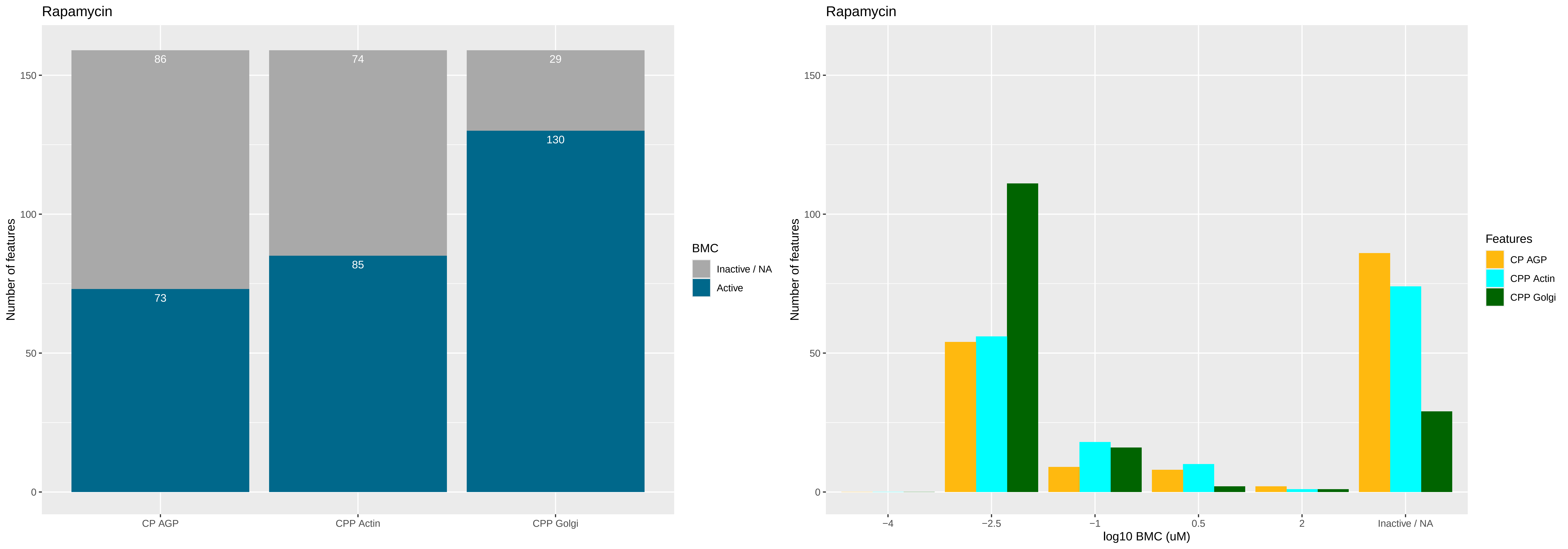

Rapamycin
86
74
29
130
85
73
150
100
50
0
Number of features
CP AGP
CPP Actin
CPP Golgi
BMC
Inactive / NA
Active
Rapamycin
150
100
50
0
Number of features
−4
−2.5
−1
0.5
2
Inactive / NA
log10 BMC (uM)
Features
CP AGP
CPP Actin
CPP Golgi

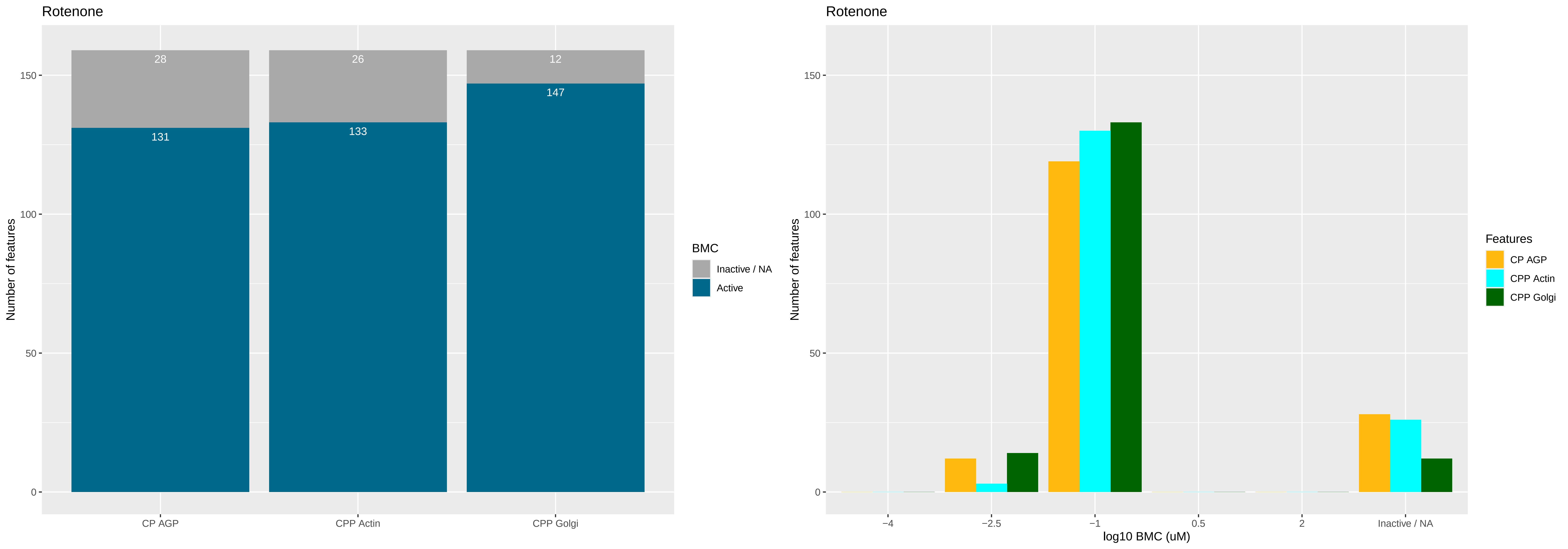
Rotenone
28
26
12
131
133
147
Number of features
150
100
50
0
CP AGP
CPP Actin
CPP Golgi
BMC
Inactive / NA
Active
Rotenone
Number of features
150
100
50
0
−4
−2.5
−1
0.5
2
Inactive / NA
log10 BMC (uM)
Features
CP AGP
CPP Actin
CPP Golgi

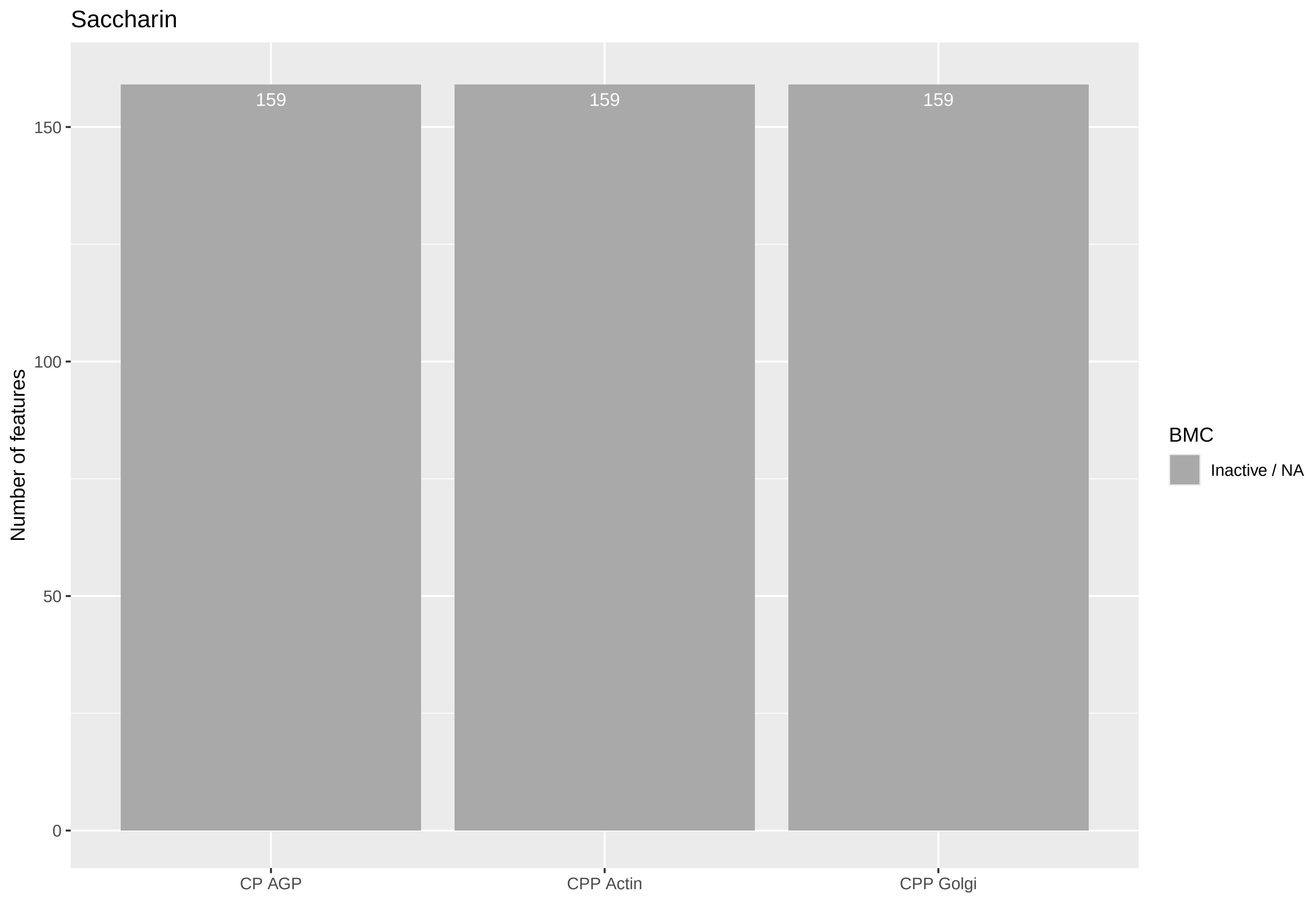
Saccharin
Number of features
159
159
159
150
100
50
0
CP AGP
CPP Actin
CPP Golgi
BMC
Inactive / NA

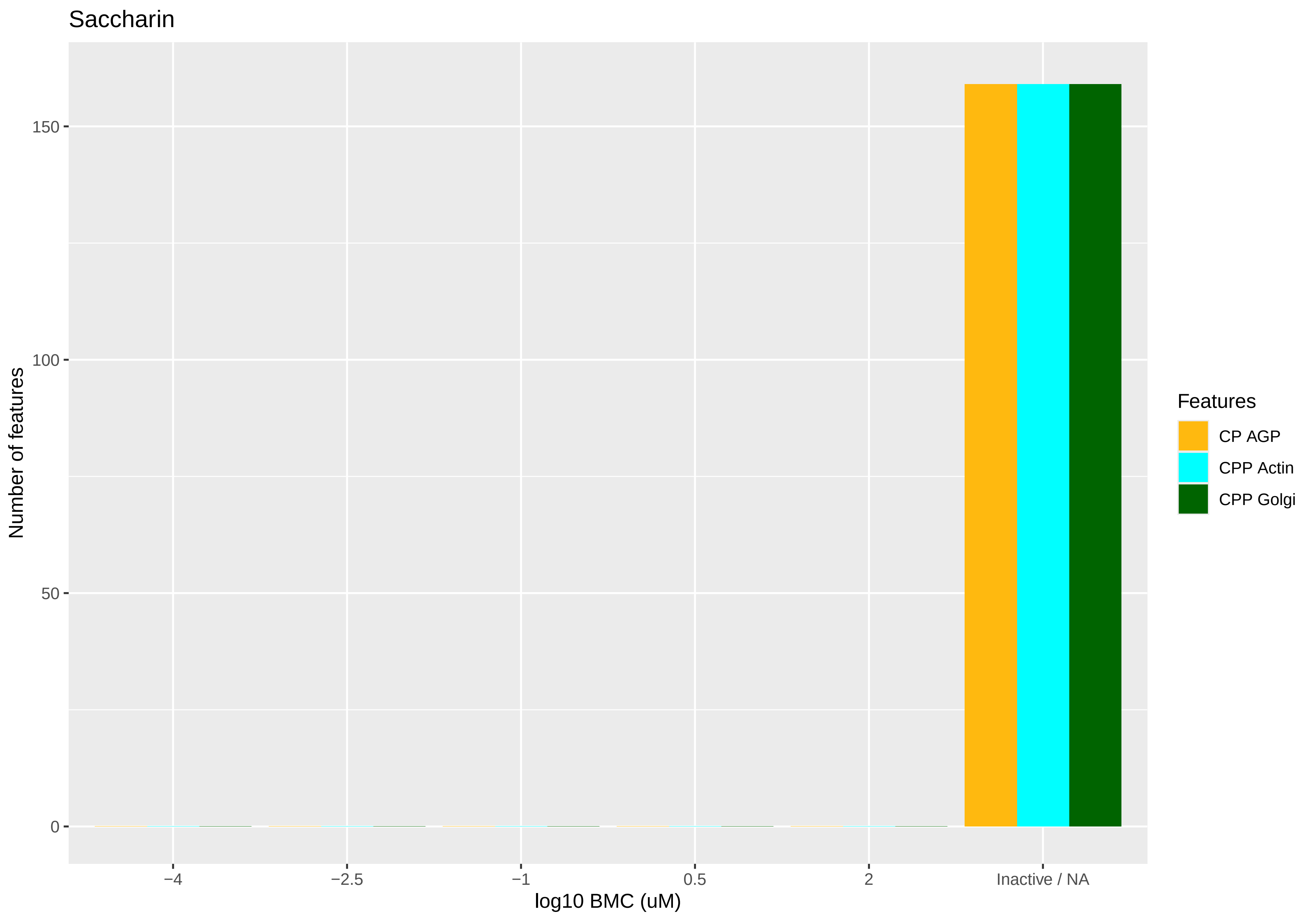
Saccharin
Number of features
150
100
50
0
−4
−2.5
−1
0.5
2
Inactive / NA
log10 BMC (uM)
Features
CP AGP
CPP Actin
CPP Golgi

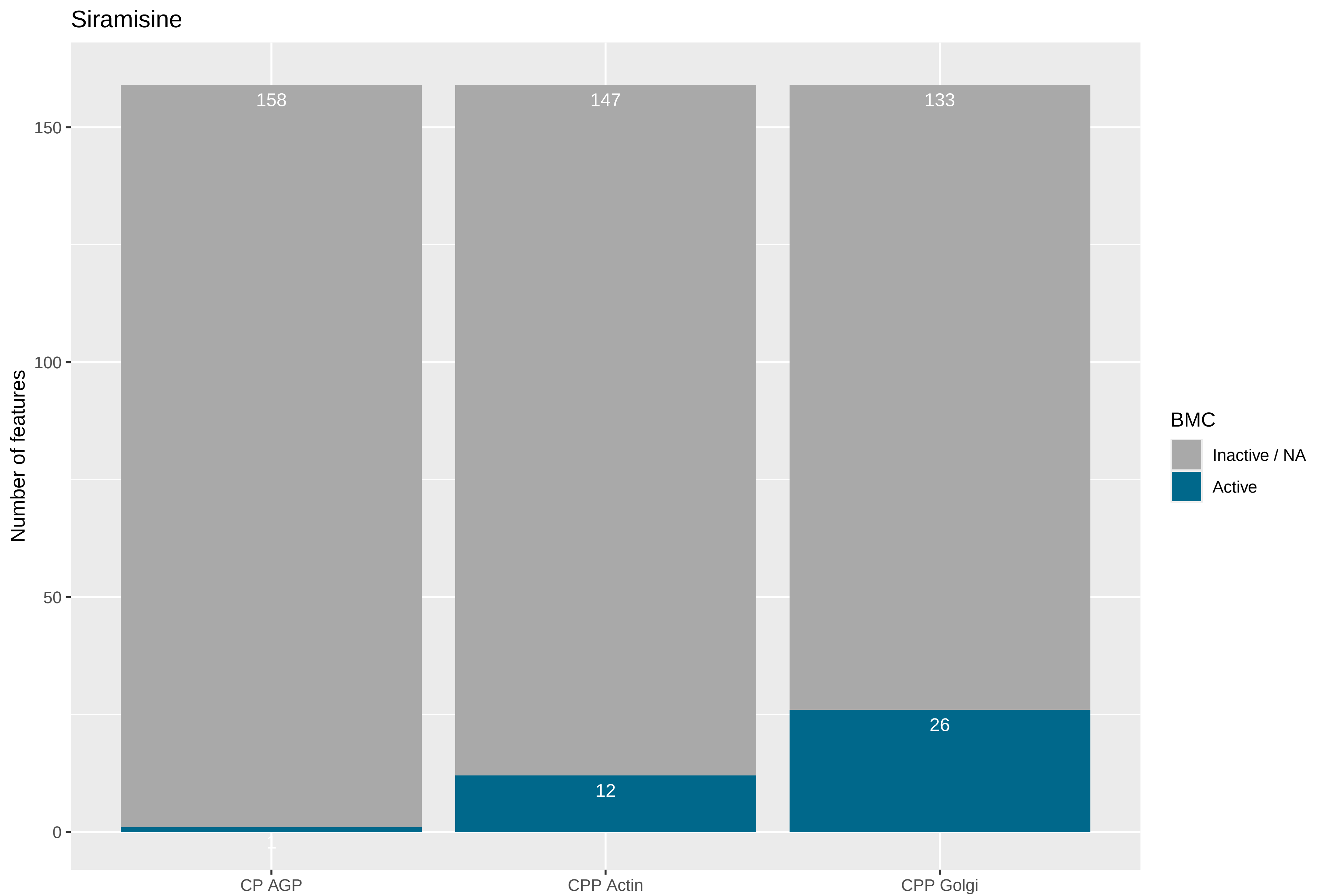
Siramisine
Number of features
158
147
133
12
26
1
150
100
50
0
CP AGP
CPP Actin
CPP Golgi
BMC
Inactive / NA
Active

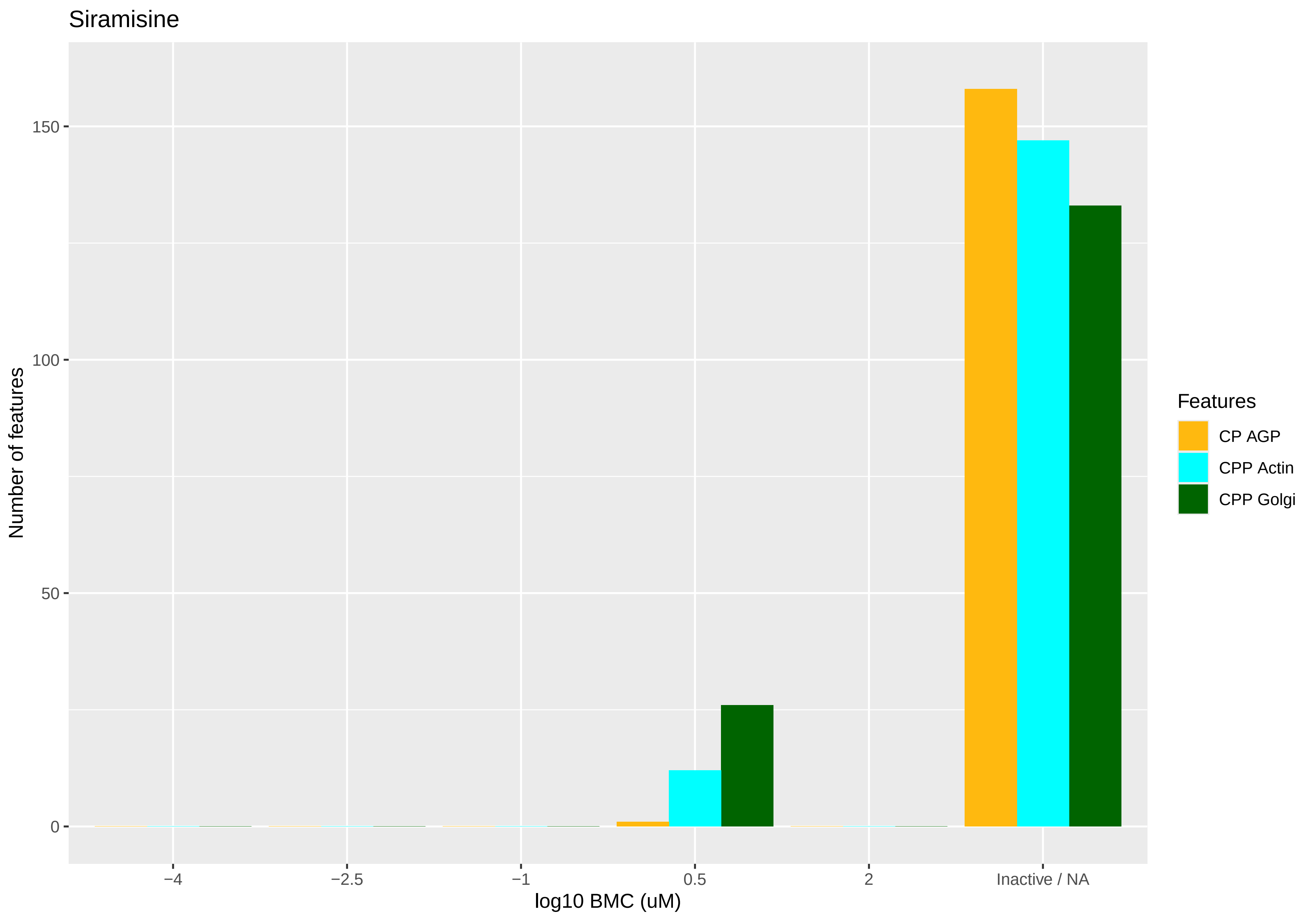
Siramisine
Number of features
150
100
50
0
−4
−2.5
−1
0.5
2
Inactive / NA
log10 BMC (uM)
Features
CP AGP
CPP Actin
CPP Golgi

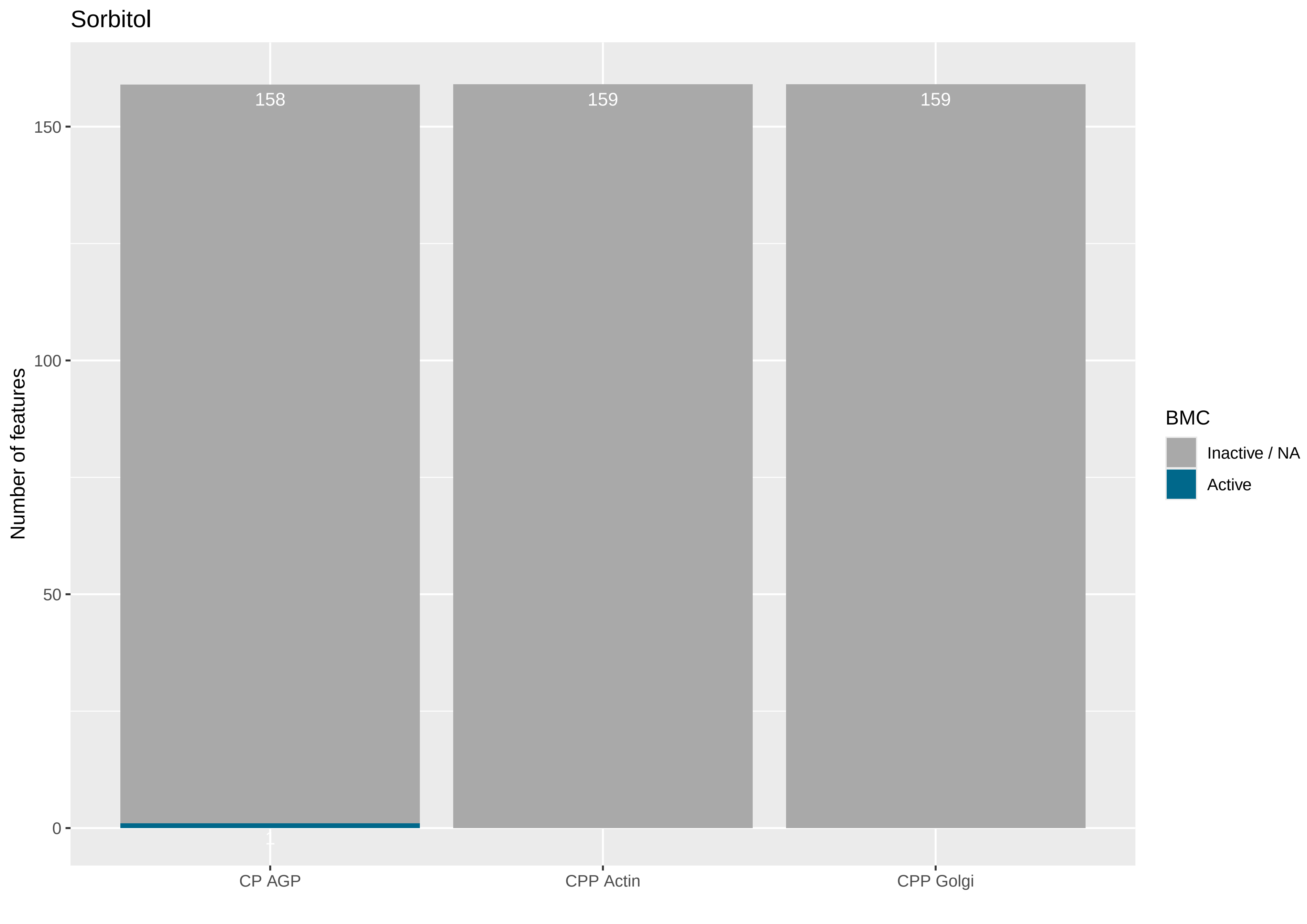
Sorbitol
Number of features
158
159
159
1
0
50
100
150
CP AGP
CPP Actin
CPP Golgi
BMC
Inactive / NA
Active

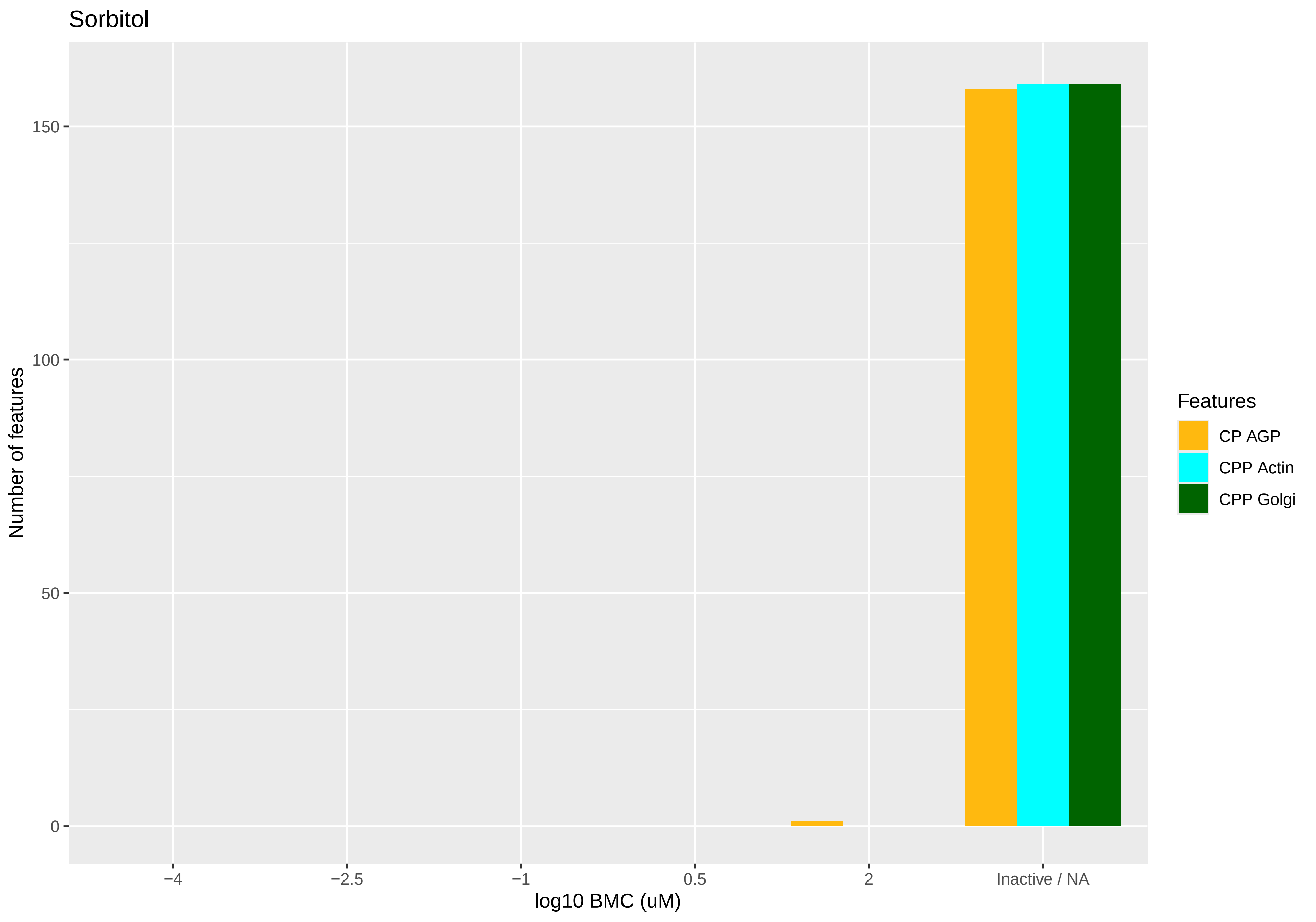
Sorbitol
Number of features
0
50
100
150
−4
−2.5
−1
0.5
2
Inactive / NA
log10 BMC (uM)
Features
CP AGP
CPP Actin
CPP Golgi

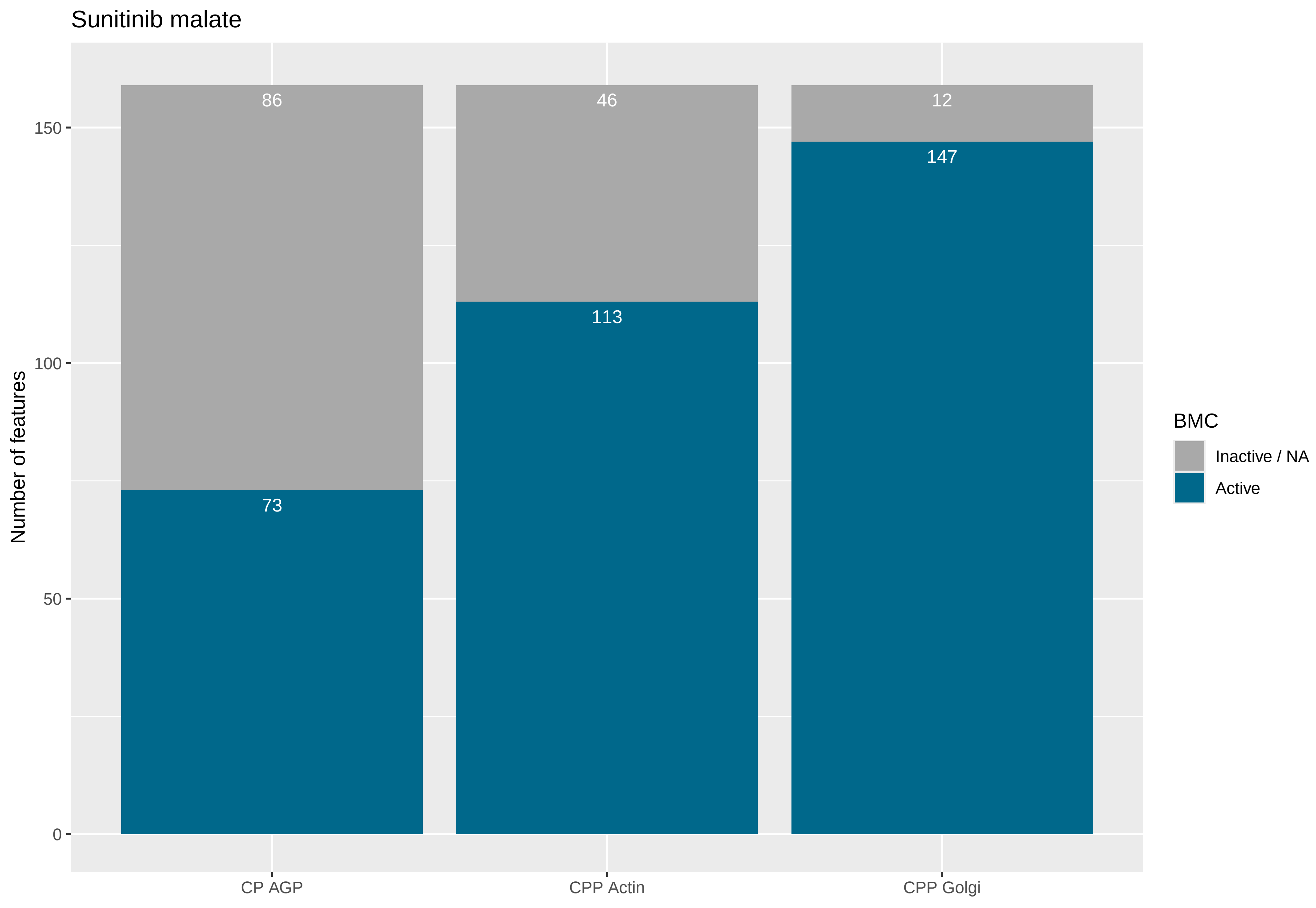

Sunitinib malate
86
46
12
147
113
73
150
100
50
0
Number of features
CP AGP
CPP Actin
CPP Golgi
BMC
Inactive / NA
Active

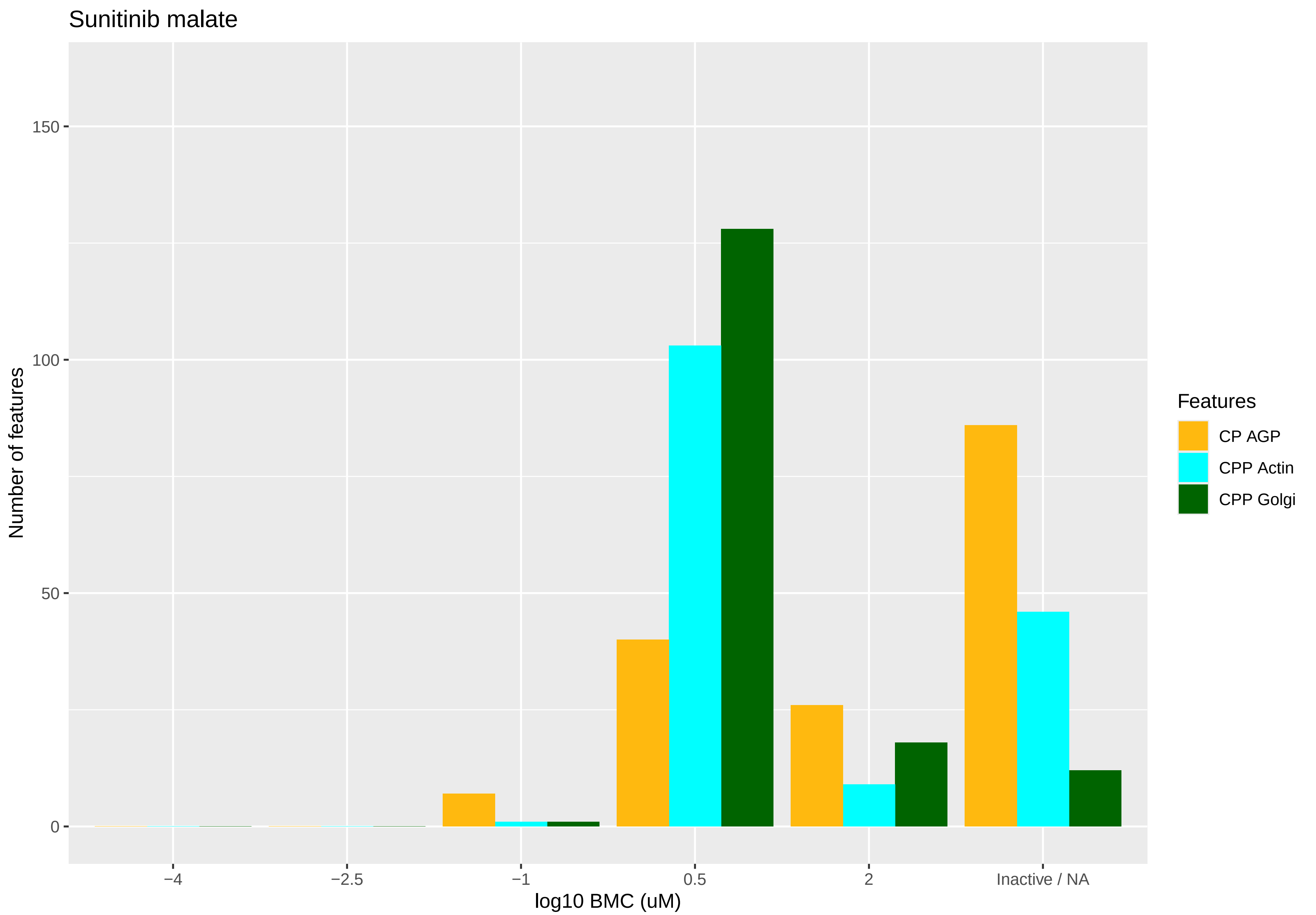

Sunitinib malate
150
100
50
0
Number of features
−4
−2.5
−1
0.5
2
Inactive / NA
log10 BMC (uM)
Features
CP AGP
CPP Actin
CPP Golgi

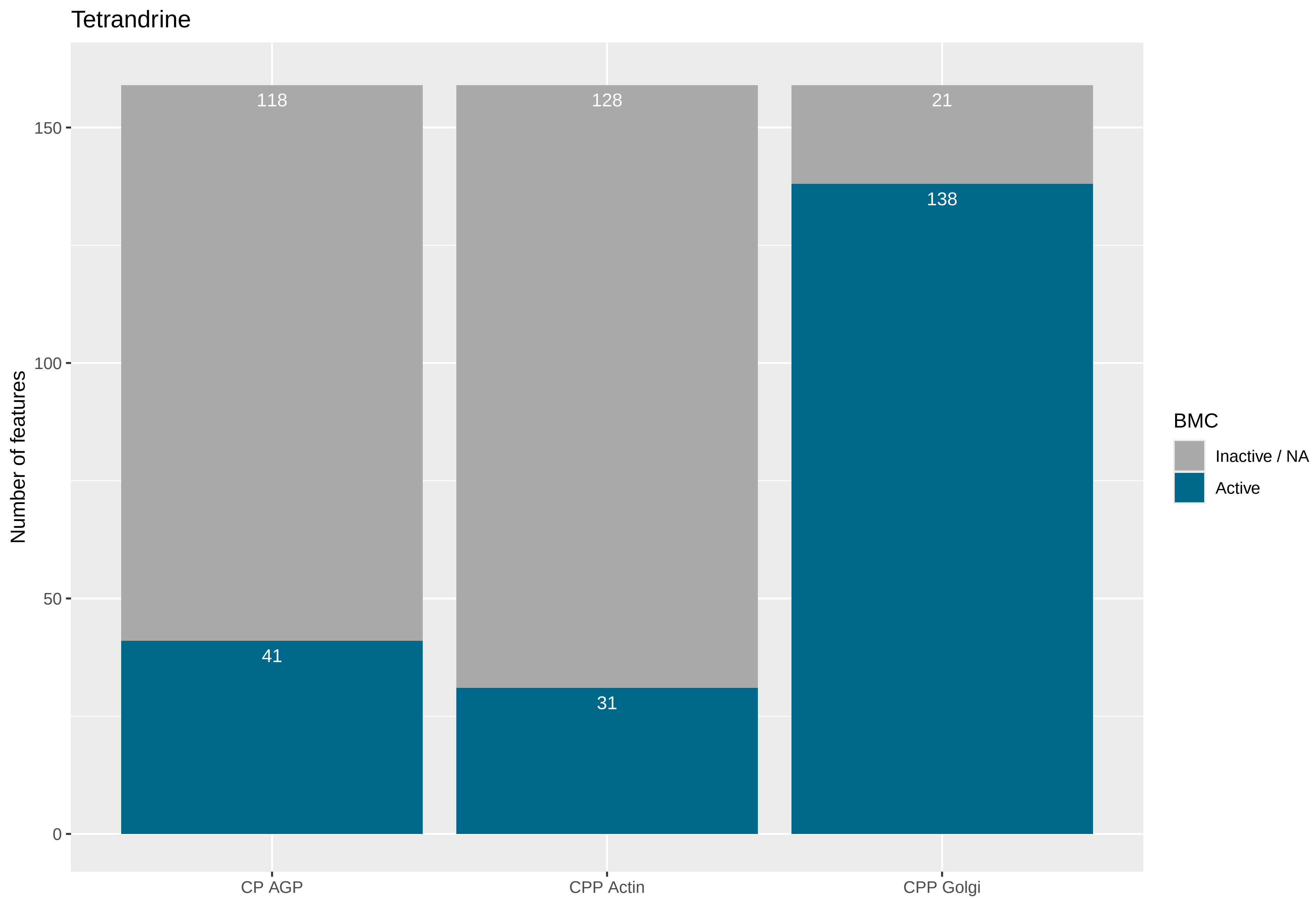
Tetrandrine
Number of features
150
100
50
0
118
128
21
138
41
31
CP AGP
CPP Actin
CPP Golgi
BMC
Inactive / NA
Active

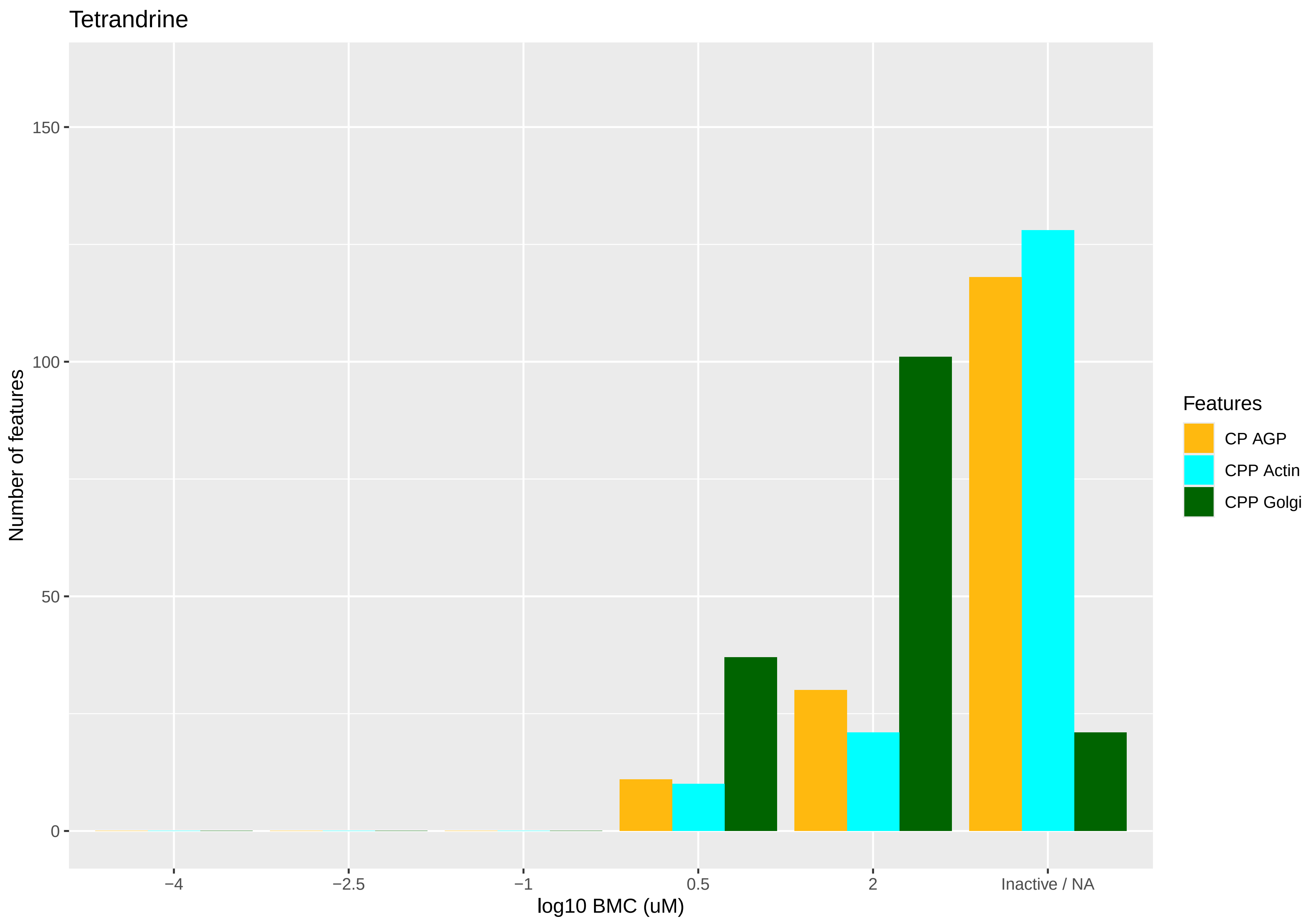
Tetrandrine
Number of features
150
100
50
0
−4
−2.5
−1
0.5
2
Inactive / NA
log10 BMC (uM)
Features
CP AGP
CPP Actin
CPP Golgi
