# Supplementary material for: Cell Painting PLUS: expanding the multiplexing capacity of Cell Painting-based phenotypic profiling using iterative staining-elution cycles
Source: Nat Commun. 2025 Apr 24;16:3857. doi: 10.1038/s41467-025-58765-8 (PMC12022024; doi:10.1038/s41467-025-58765-8)
Supplement: Supplementary file 3 — Supplementary Data [file 41467_2025_58765_MOESM3_ESM.zip › Supplementary Data/Supplementary Data_24_FigS5_BMCBarPlots_CPP_CP_RNAER_Cytoplasm.pdf]

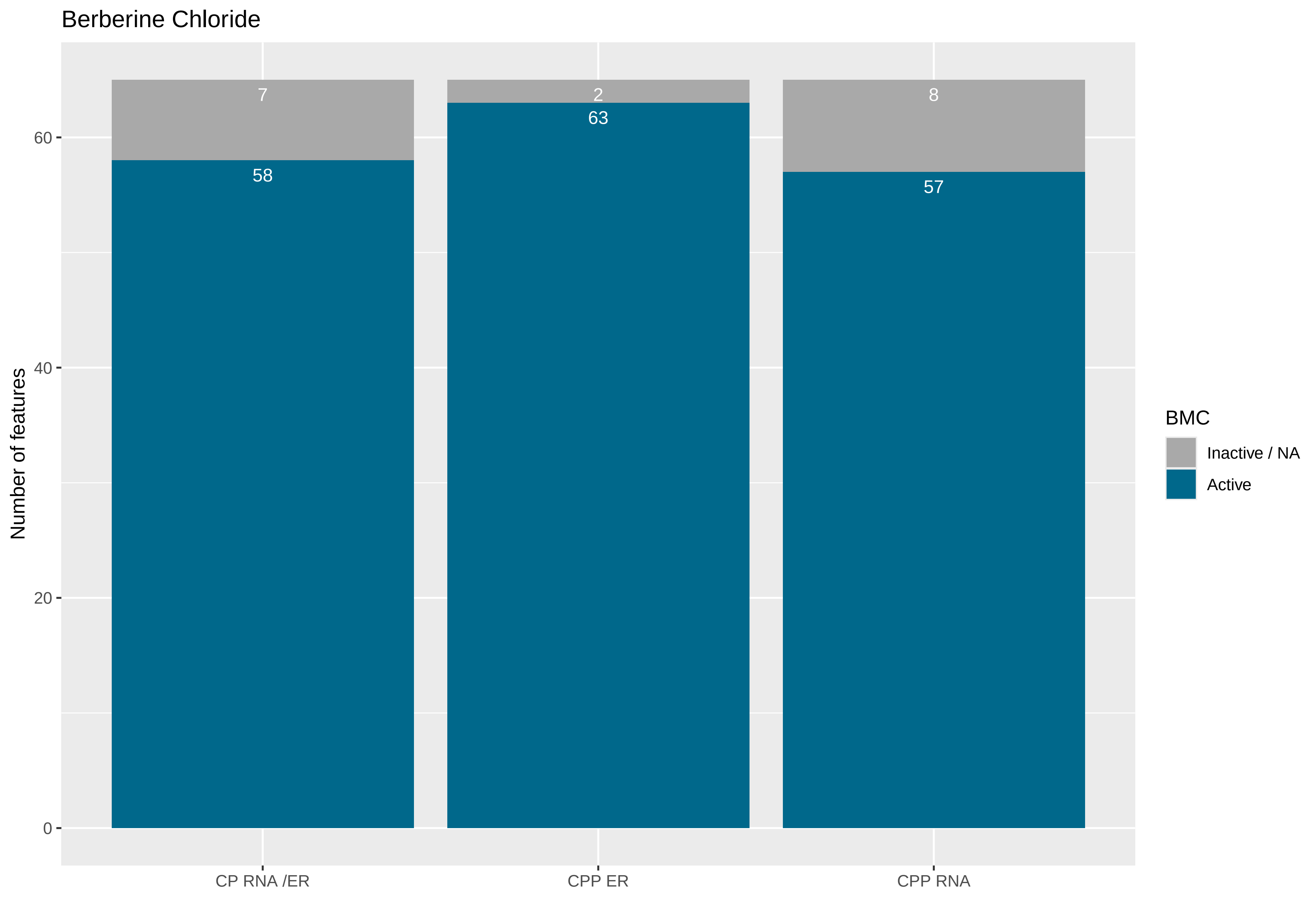

Berberine Chloride
Number of features
0
20
40
60
7
58
2
63
8
57
CP RNA /ER
CPP ER
CPP RNA
BMC
Inactive / NA
Active

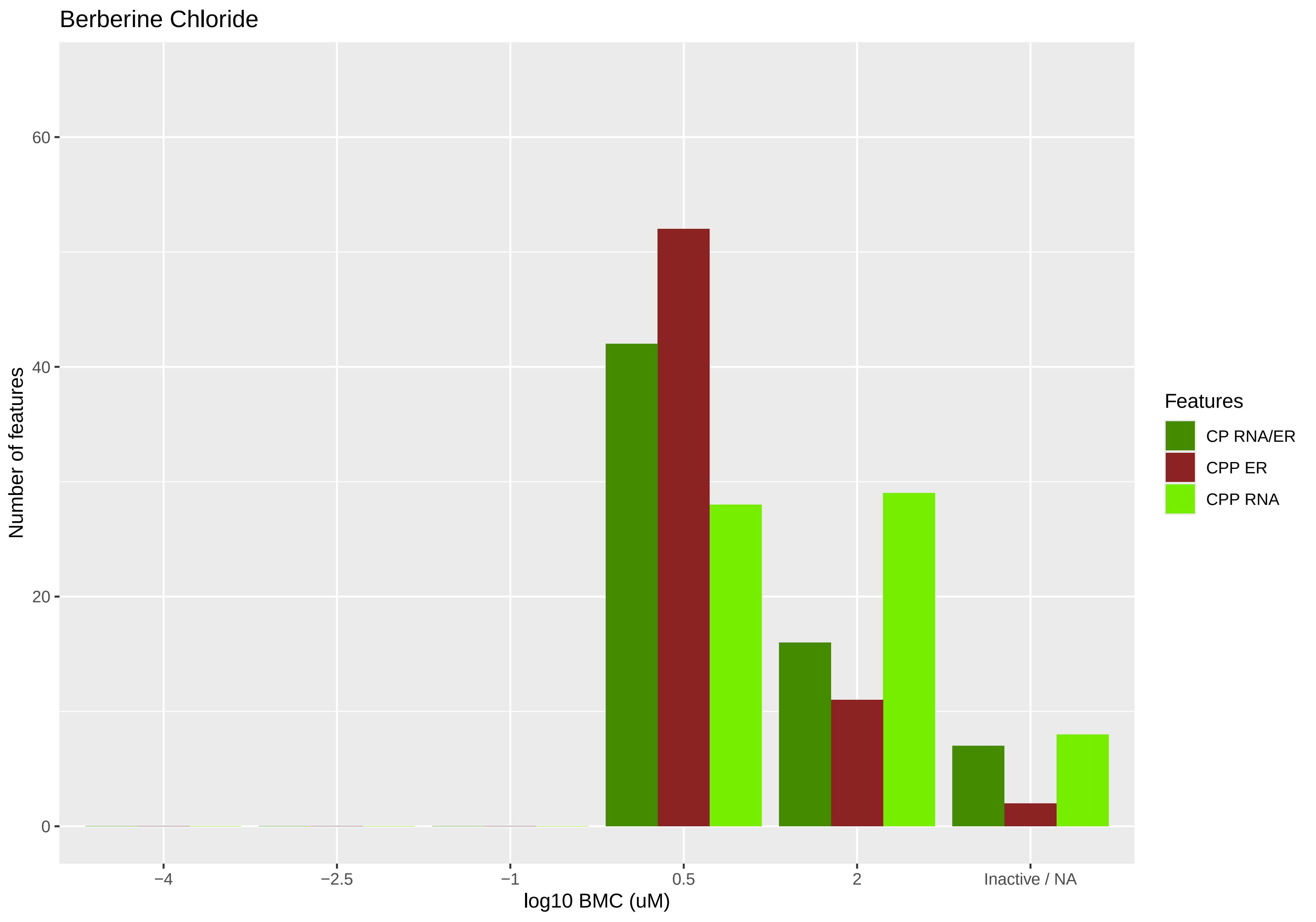

Berberine Chloride
Number of features
0
20
40
60
−4
−2.5
−1
0.5
2
Inactive / NA
log10 BMC (uM)
Features
CP RNA/ER
CPP ER
CPP RNA

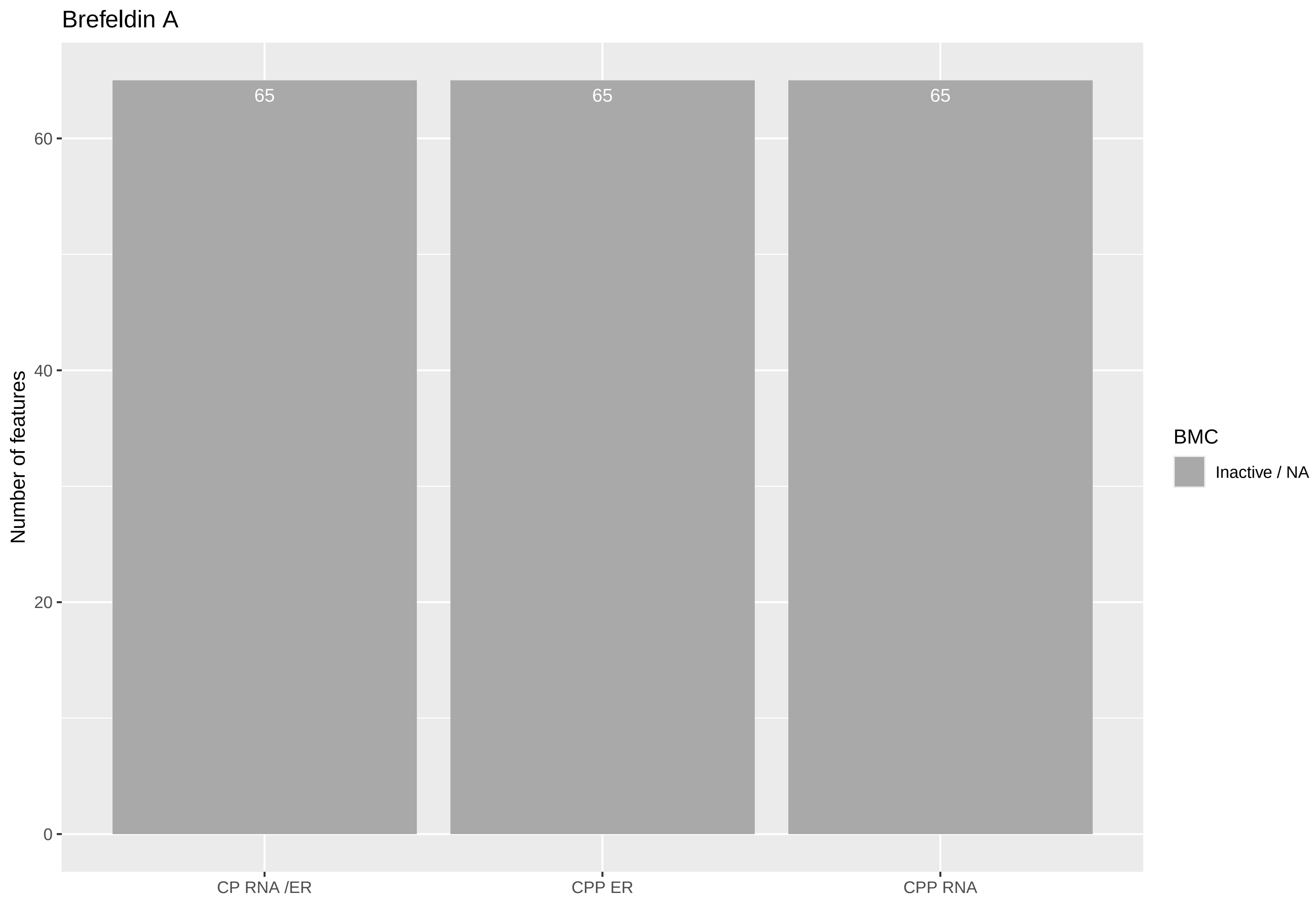

Brefeldin A
Number of features
0
20
40
60
65
65
65
CP RNA /ER
CPP ER
CPP RNA
BMC
Inactive / NA

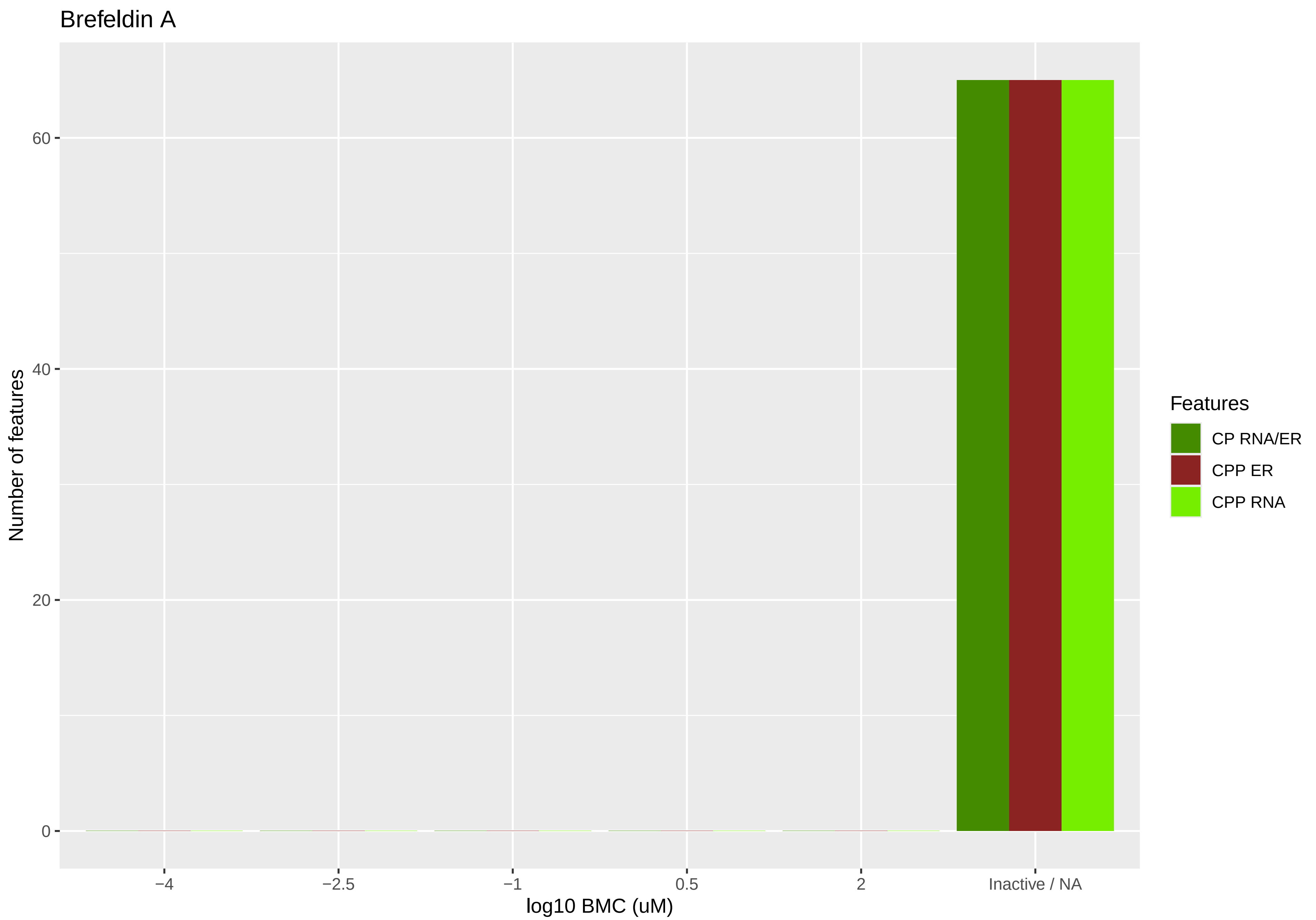

Brefeldin A
Number of features
0
20
40
60
−4
−2.5
−1
0.5
2
Inactive / NA
log10 BMC (uM)
Features
CP RNA/ER
CPP ER
CPP RNA

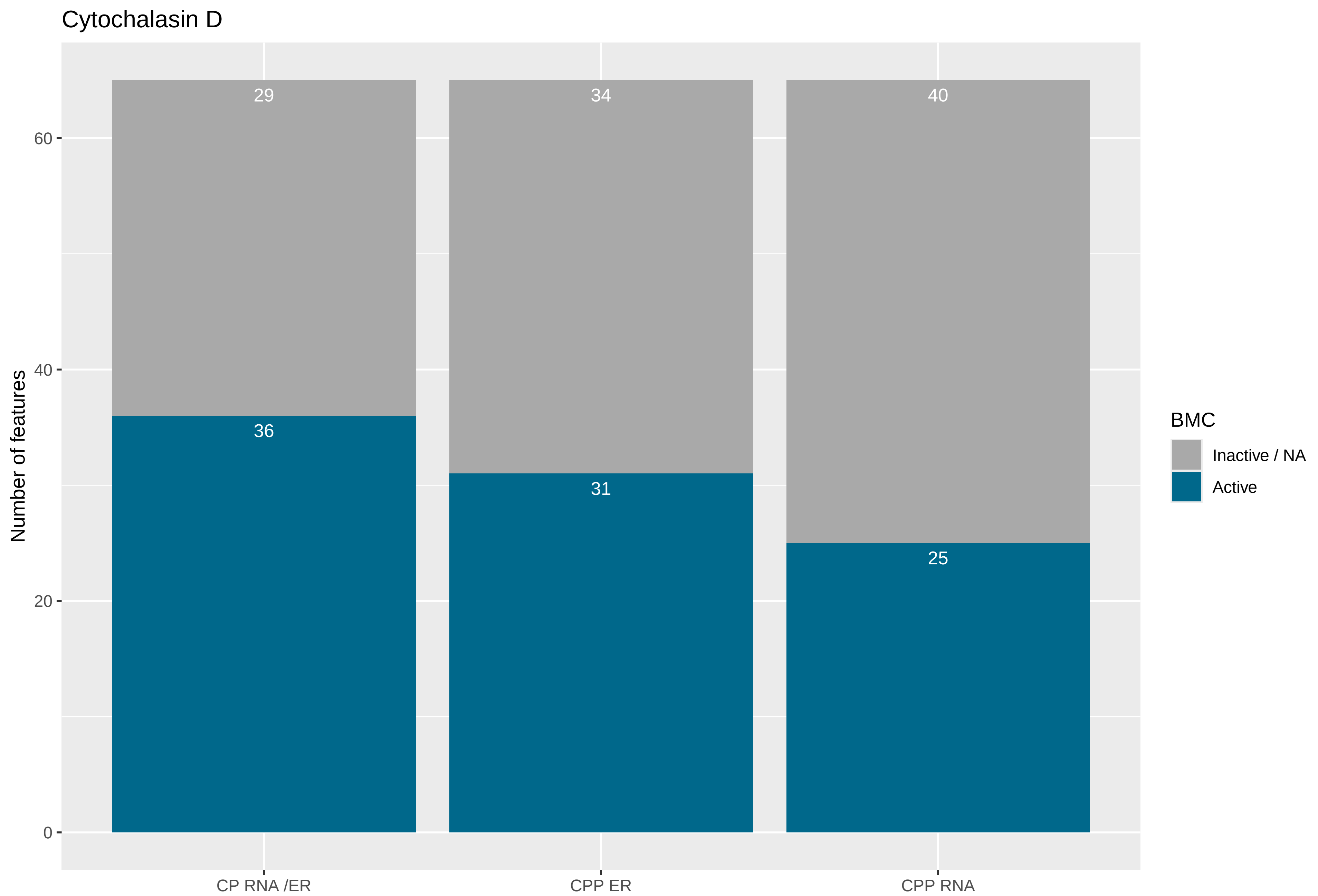

Cytochalasin D
Number of features
0
20
40
60
29
34
40
36
31
25
CP RNA /ER
CPP ER
CPP RNA
BMC
Inactive / NA
Active

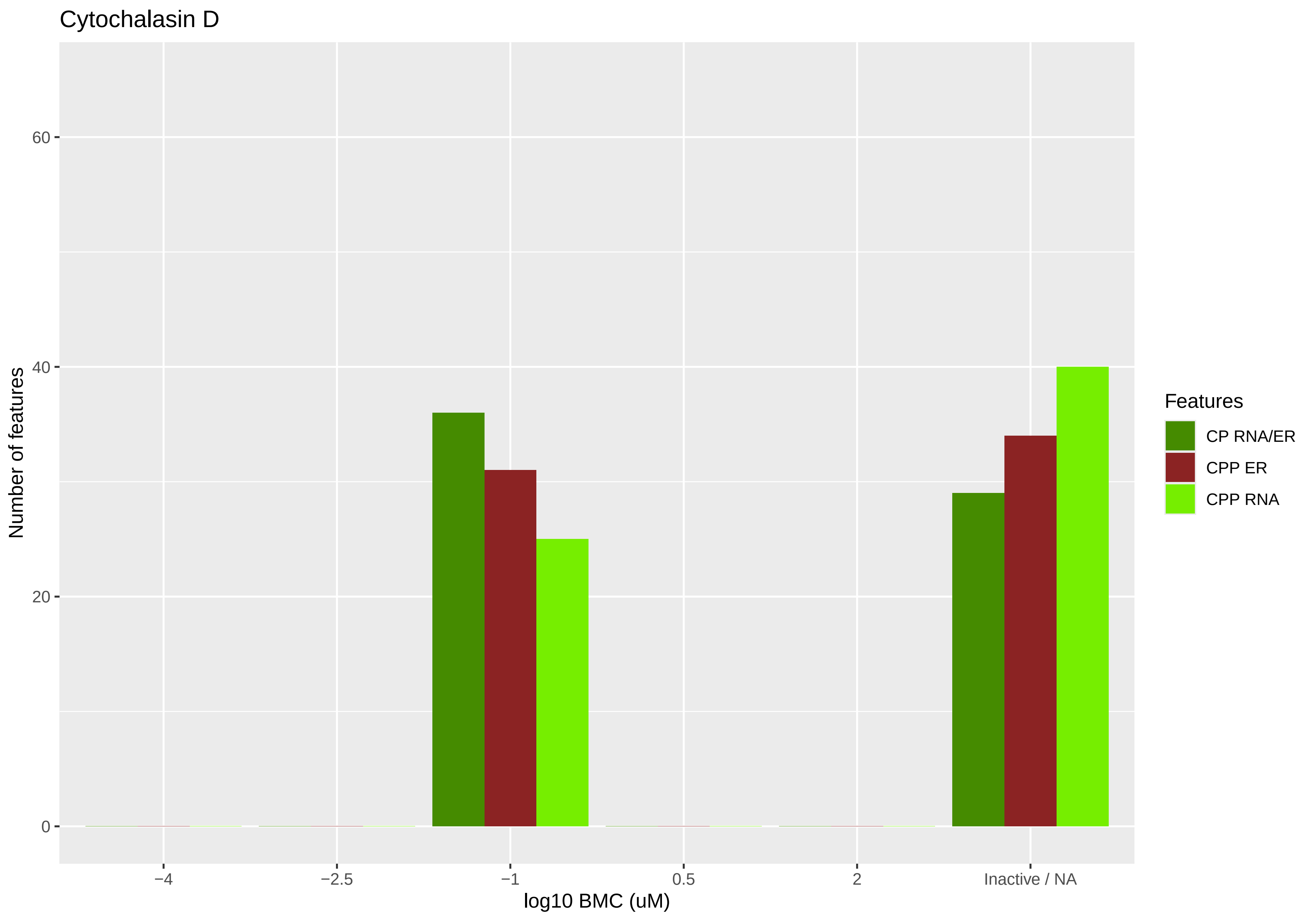

Cytochalasin D
Number of features
0
20
40
60
−4
−2.5
−1
0.5
2
Inactive / NA
log10 BMC (uM)
Features
CP RNA/ER
CPP ER
CPP RNA

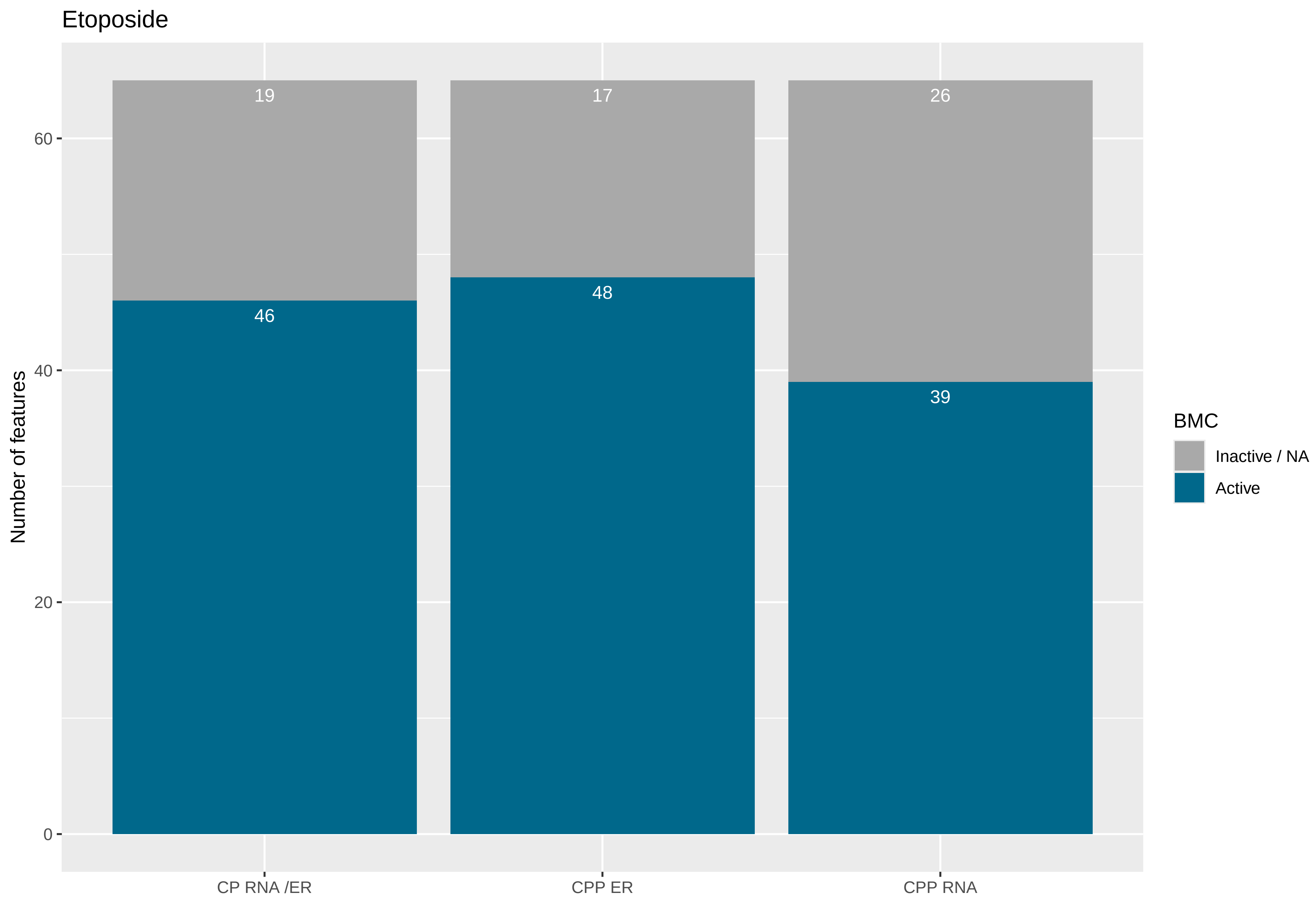
Etoposide
Number of features
19
17
26
46
48
39
CP RNA /ER
CPP ER
CPP RNA
BMC
Inactive / NA
Active
0
20
40
60

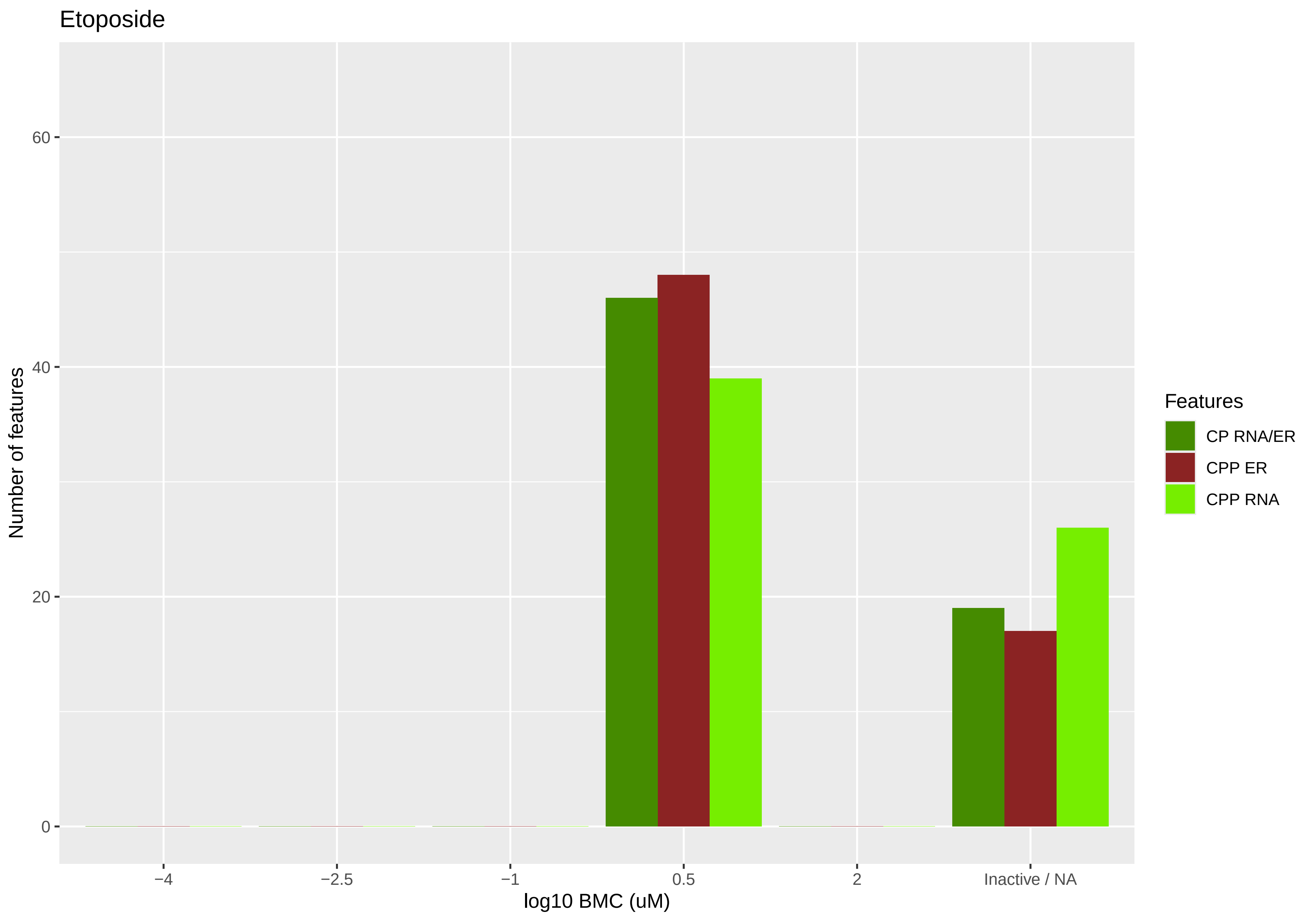
Etoposide
Number of features
log10 BMC (uM)
−4
−2.5
−1
0.5
2
Inactive / NA
Features
CP RNA/ER
CPP ER
CPP RNA
0
20
40
60

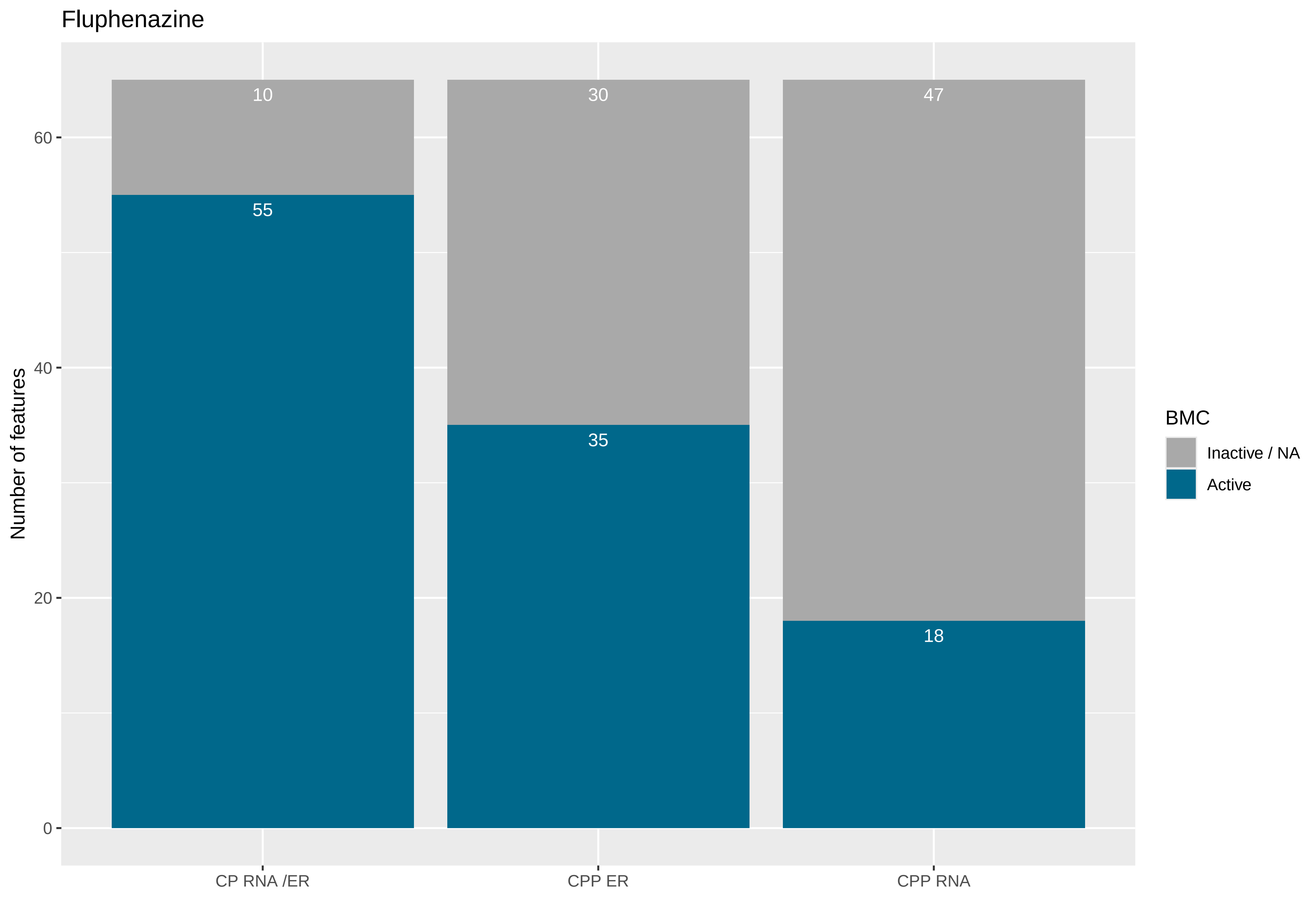
Fluphenazine
Number of features
BMC
Inactive / NA
Active
10
30
47
55
35
18
0
20
40
60
CP RNA /ER
CPP ER
CPP RNA
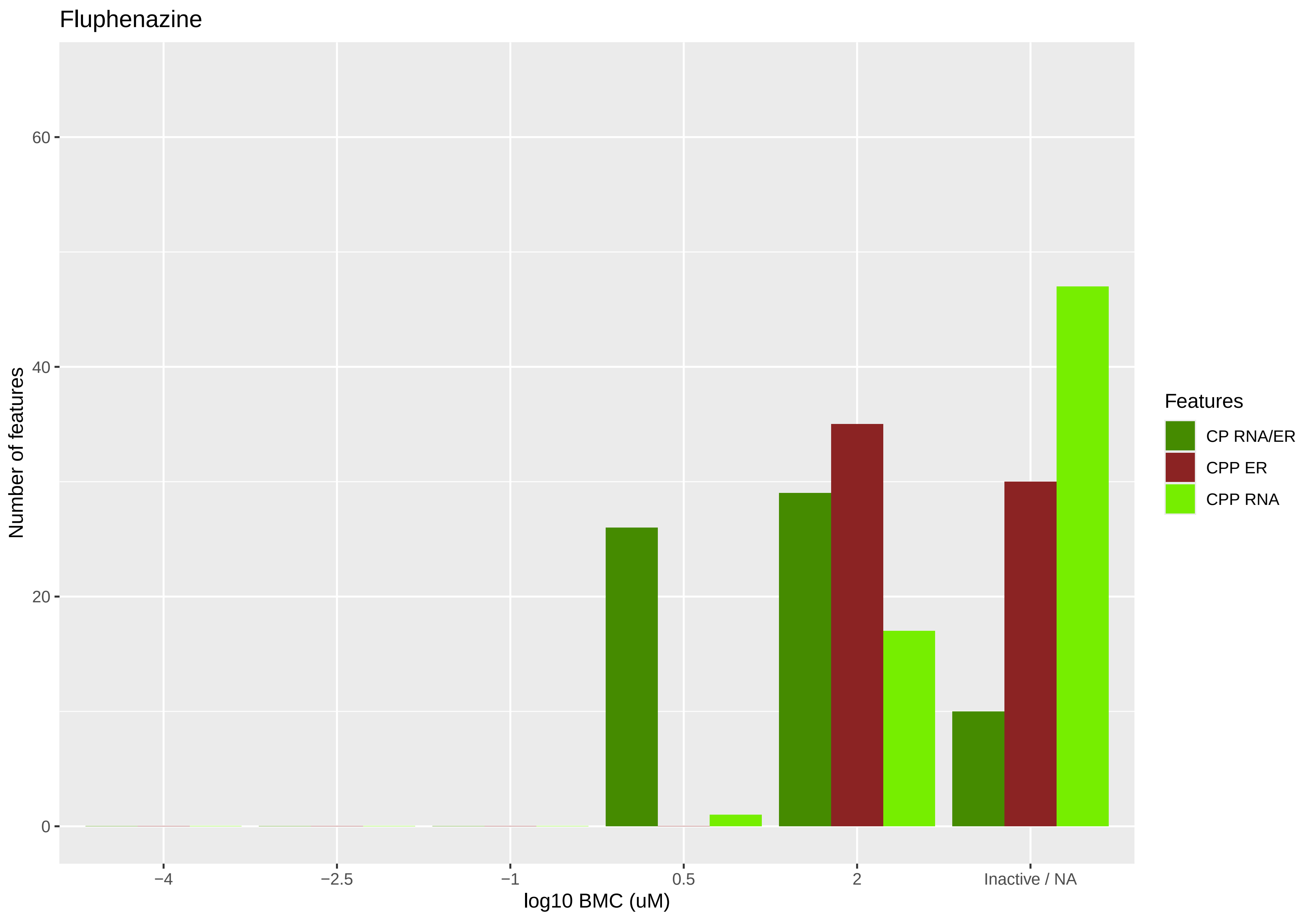
Fluphenazine
Number of features
log10 BMC (uM)
Features
CP RNA/ER
CPP ER
CPP RNA
0
20
40
60
−4
−2.5
−1
0.5
2
Inactive / NA

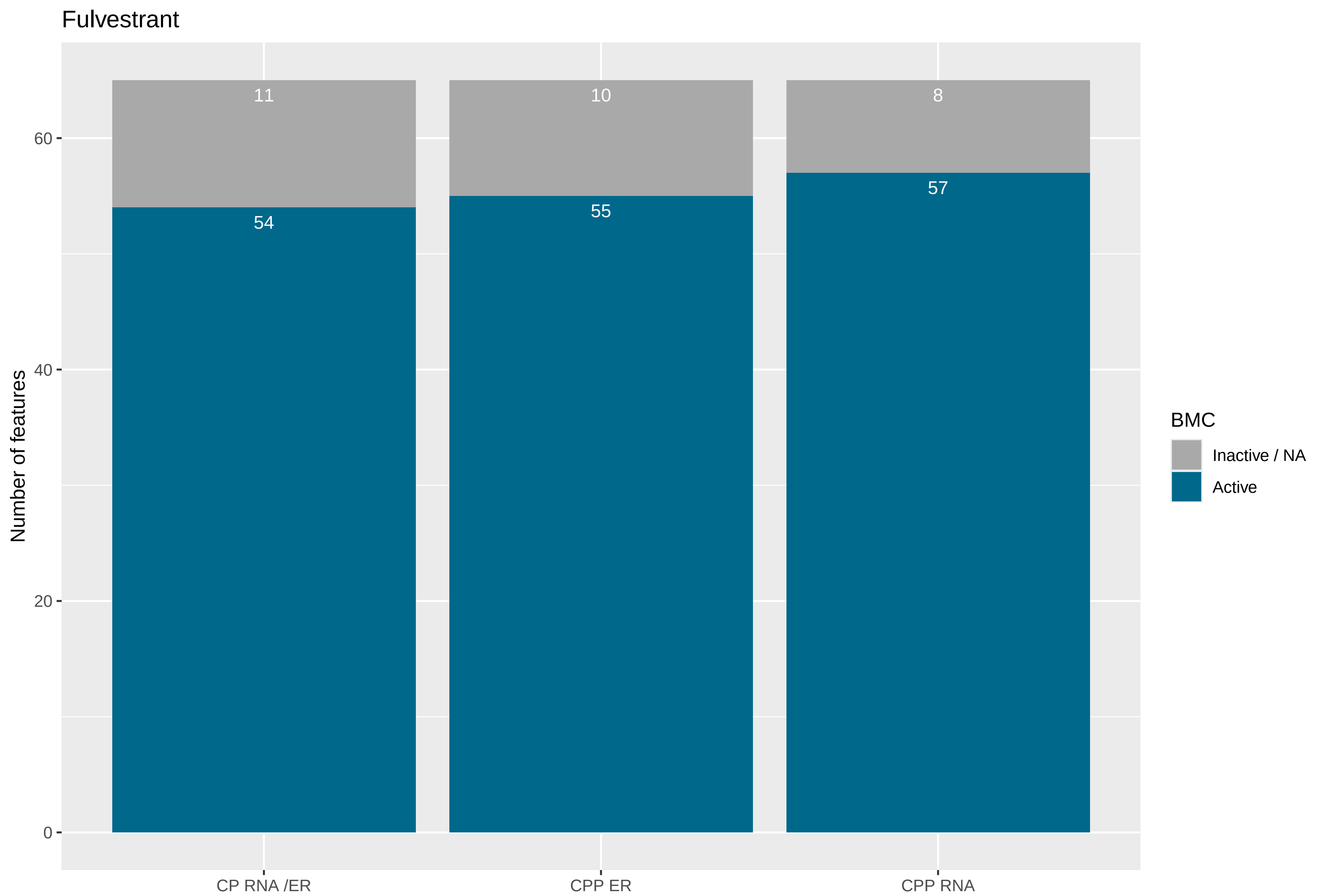

Fulvestrant
Number of features
60
40
20
0
11
10
8
54
55
57
CP RNA /ER
CPP ER
CPP RNA
BMC
Inactive / NA
Active

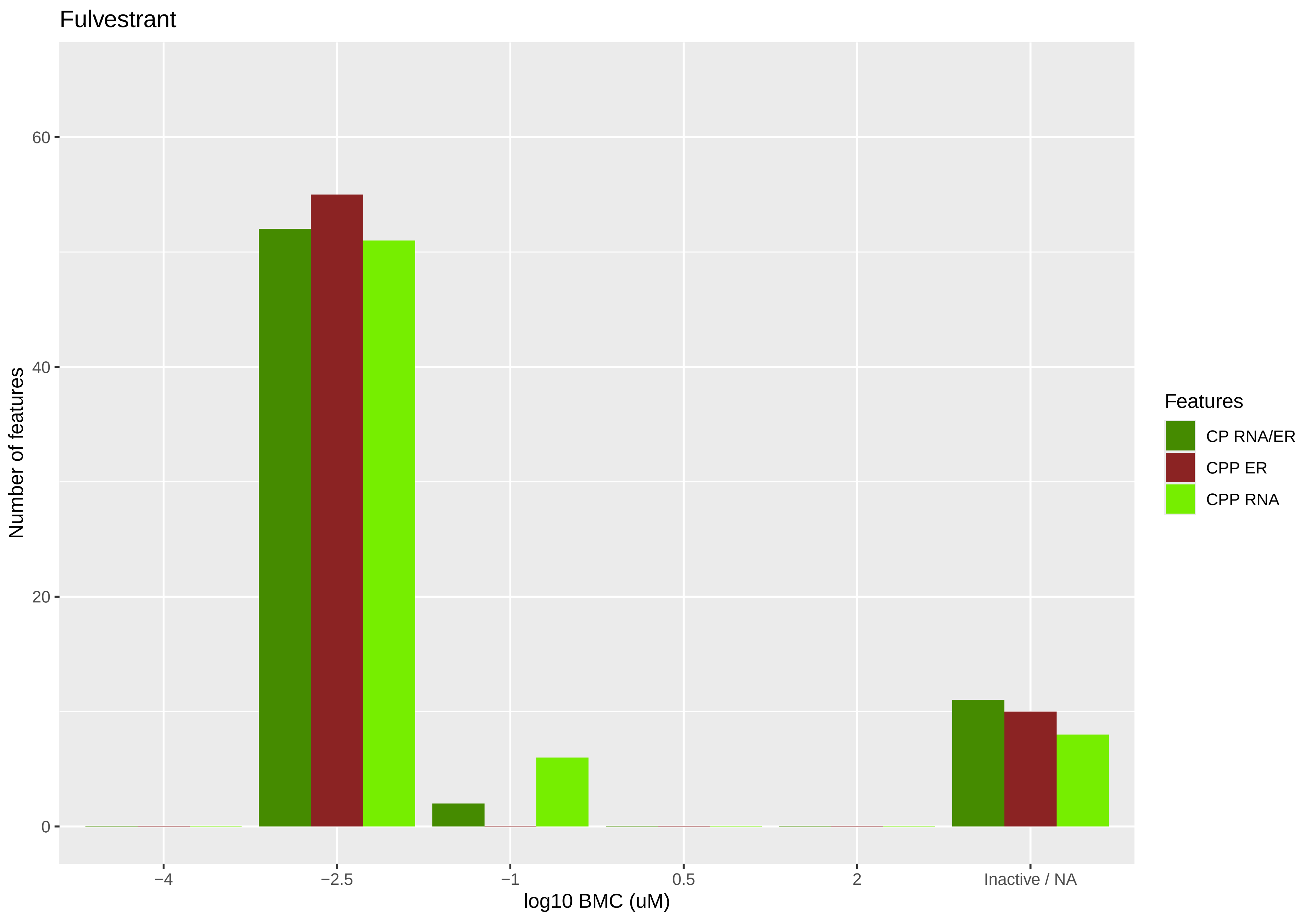

Fulvestrant
Number of features
60
40
20
0
−4
−2.5
−1
0.5
2
Inactive / NA
log10 BMC (uM)
Features
CP RNA/ER
CPP ER
CPP RNA

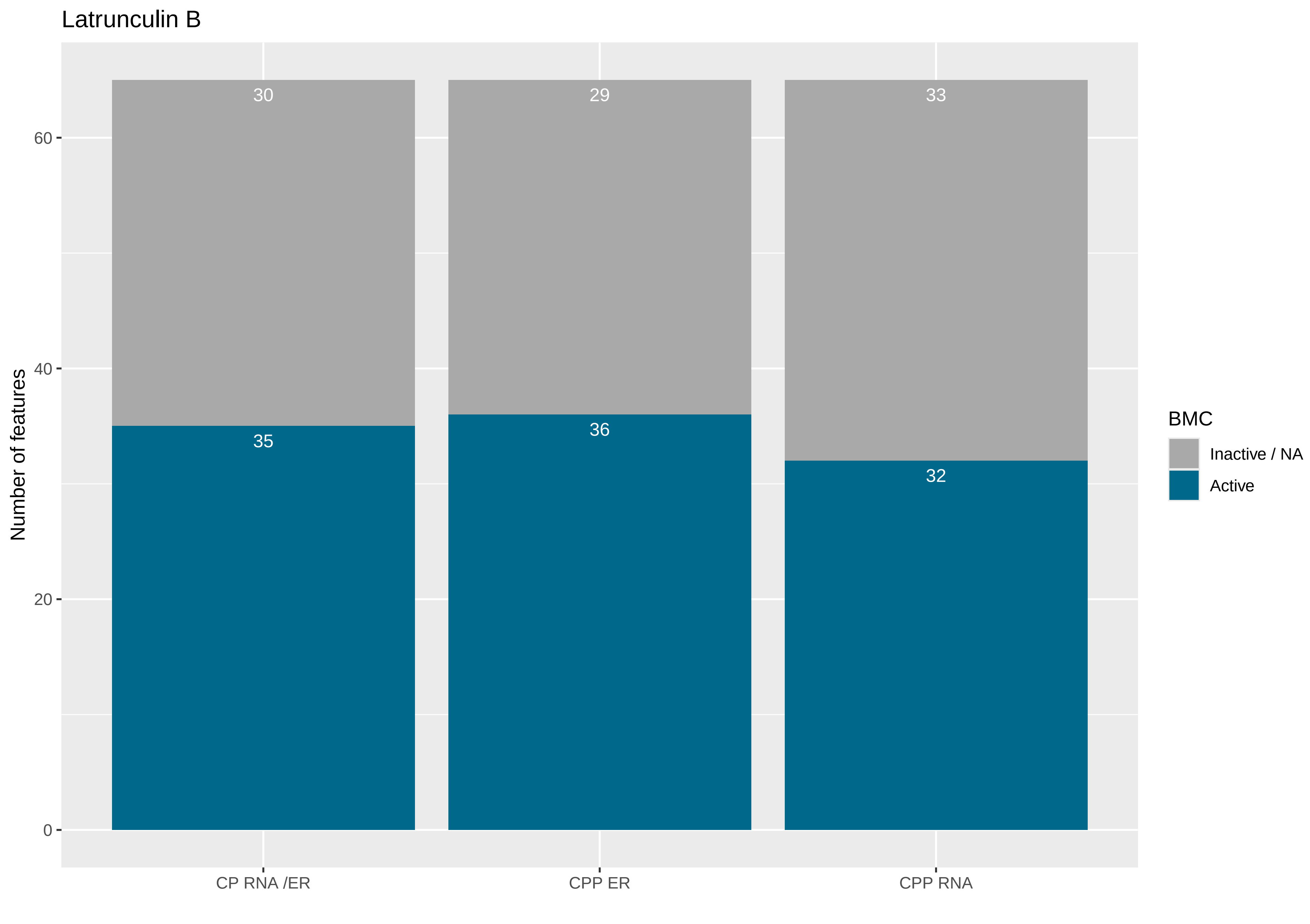

Latrunculin B
Number of features
30
29
33
35
36
32
60
40
20
0
CP RNA /ER
CPP ER
CPP RNA
BMC
Inactive / NA
Active

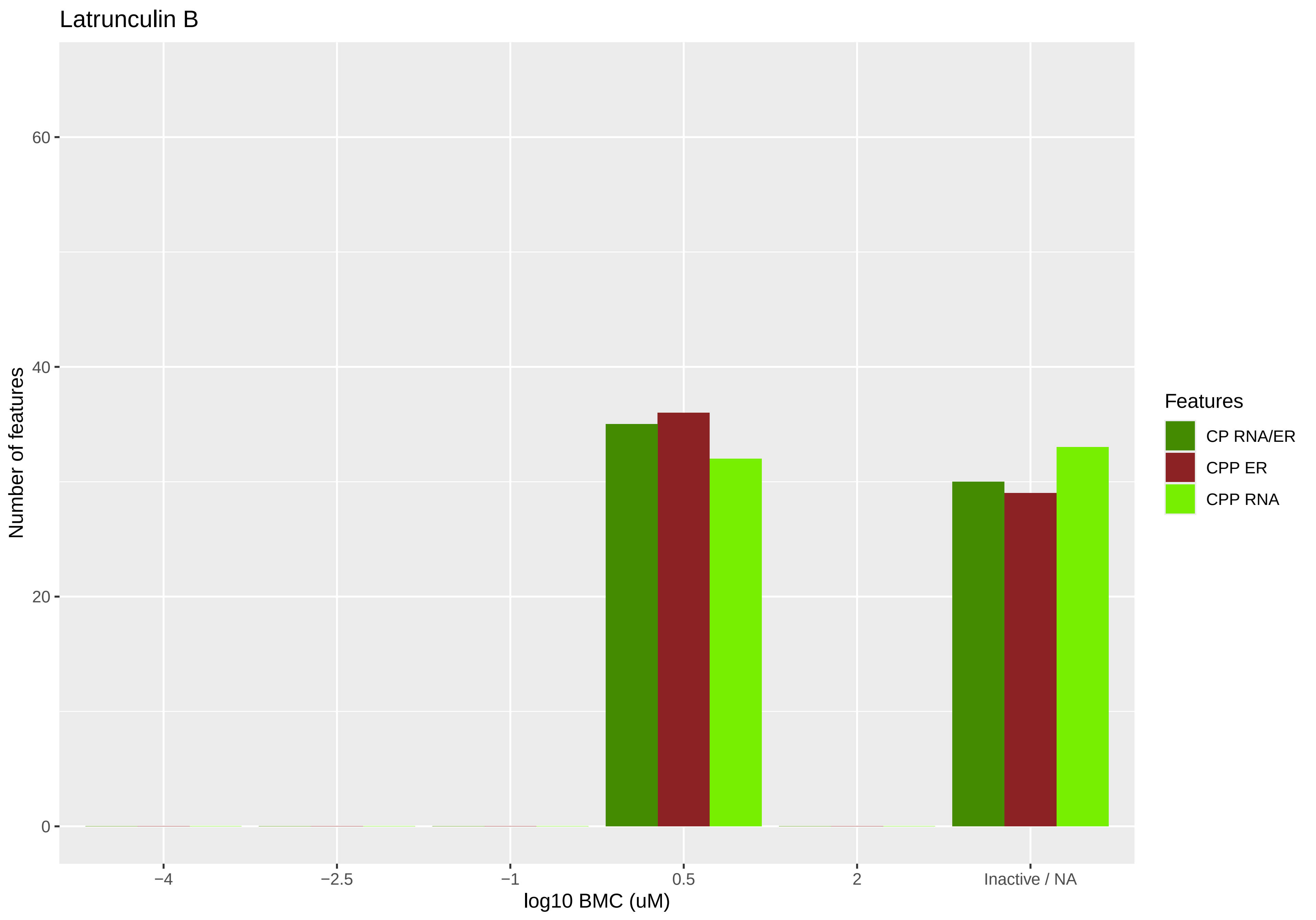

Latrunculin B
Number of features
60
40
20
0
−4
−2.5
−1
0.5
2
Inactive / NA
log10 BMC (uM)
Features
CP RNA/ER
CPP ER
CPP RNA

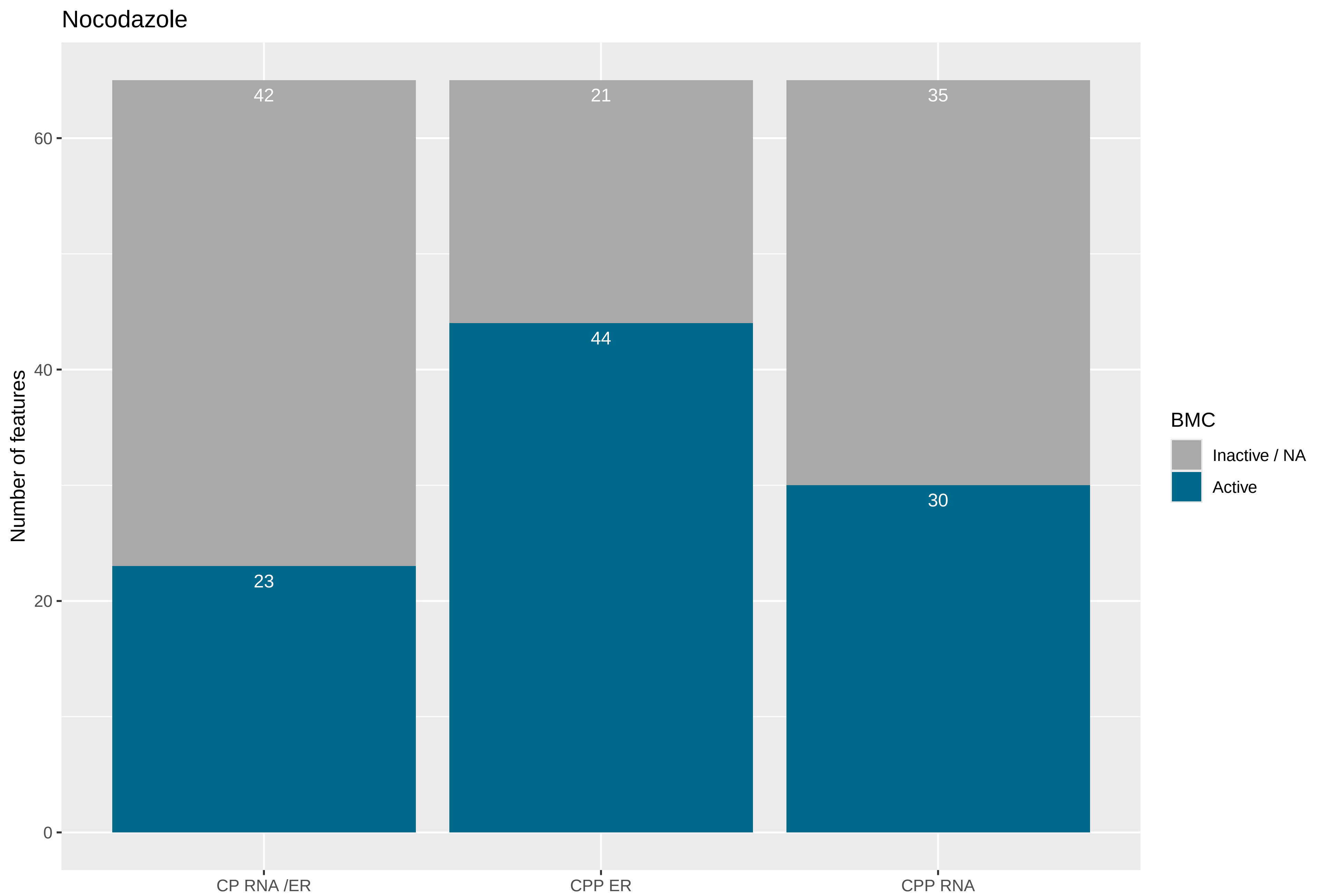
Nocodazole
42
21
35
44
30
23
60
40
20
0
Number of features
CP RNA /ER
CPP ER
CPP RNA
BMC
Inactive / NA
Active

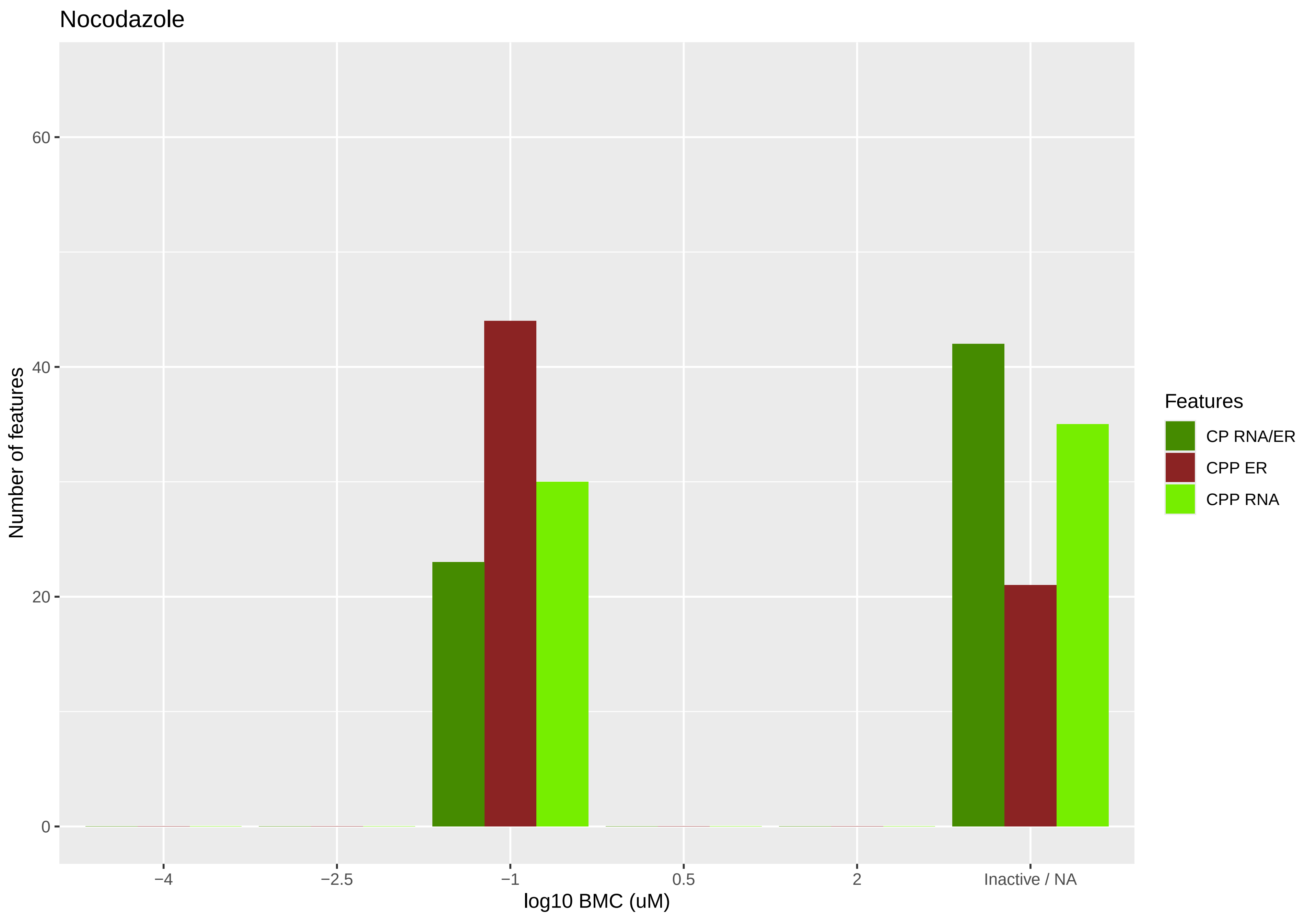
Nocodazole
60
40
20
0
Number of features
−4
−2.5
−1
0.5
2
Inactive / NA
log10 BMC (uM)
Features
CP RNA/ER
CPP ER
CPP RNA

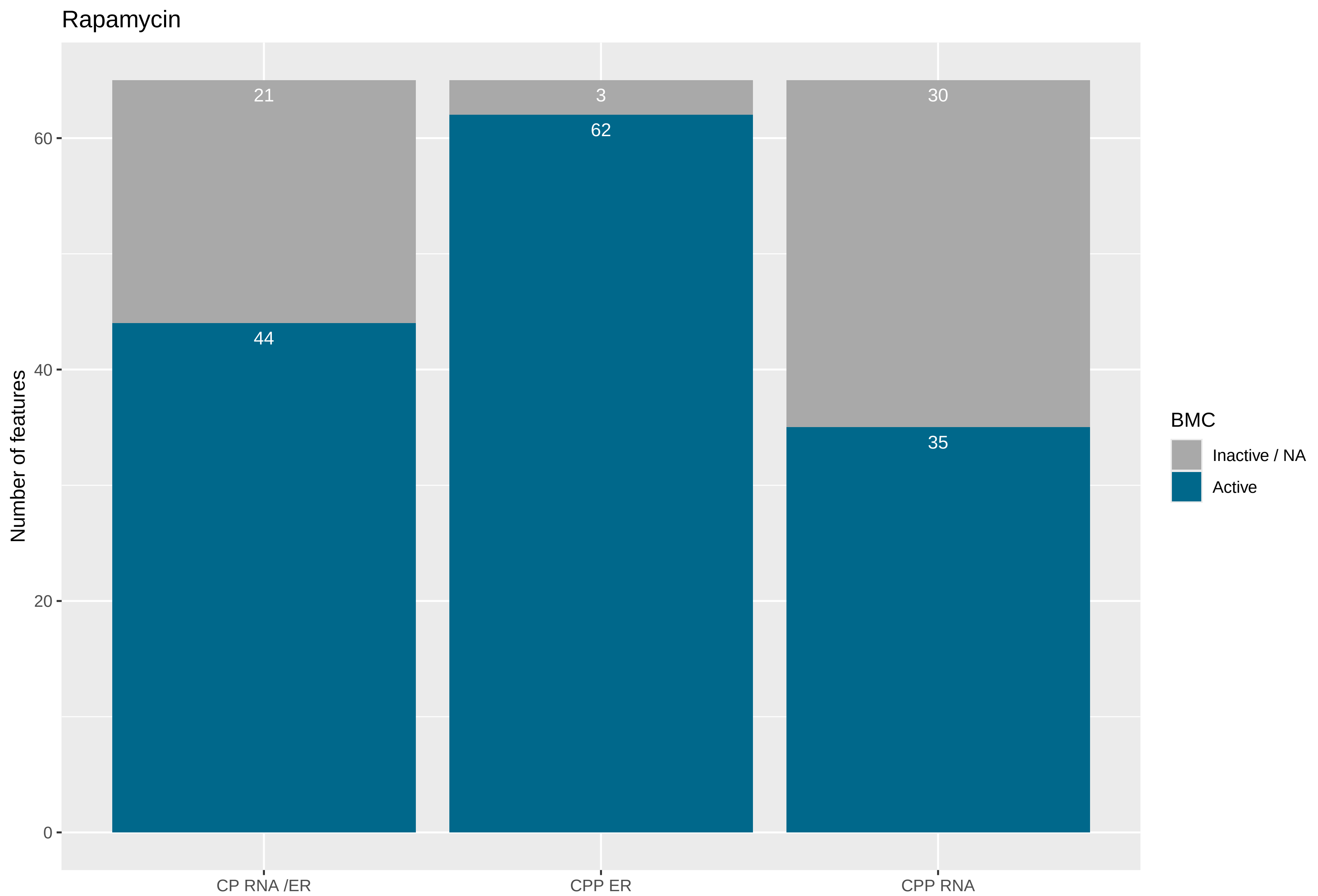

Rapamycin
Number of features
21
44
3
62
30
35
0
20
40
60
CP RNA /ER
CPP ER
CPP RNA
BMC
Inactive / NA
Active

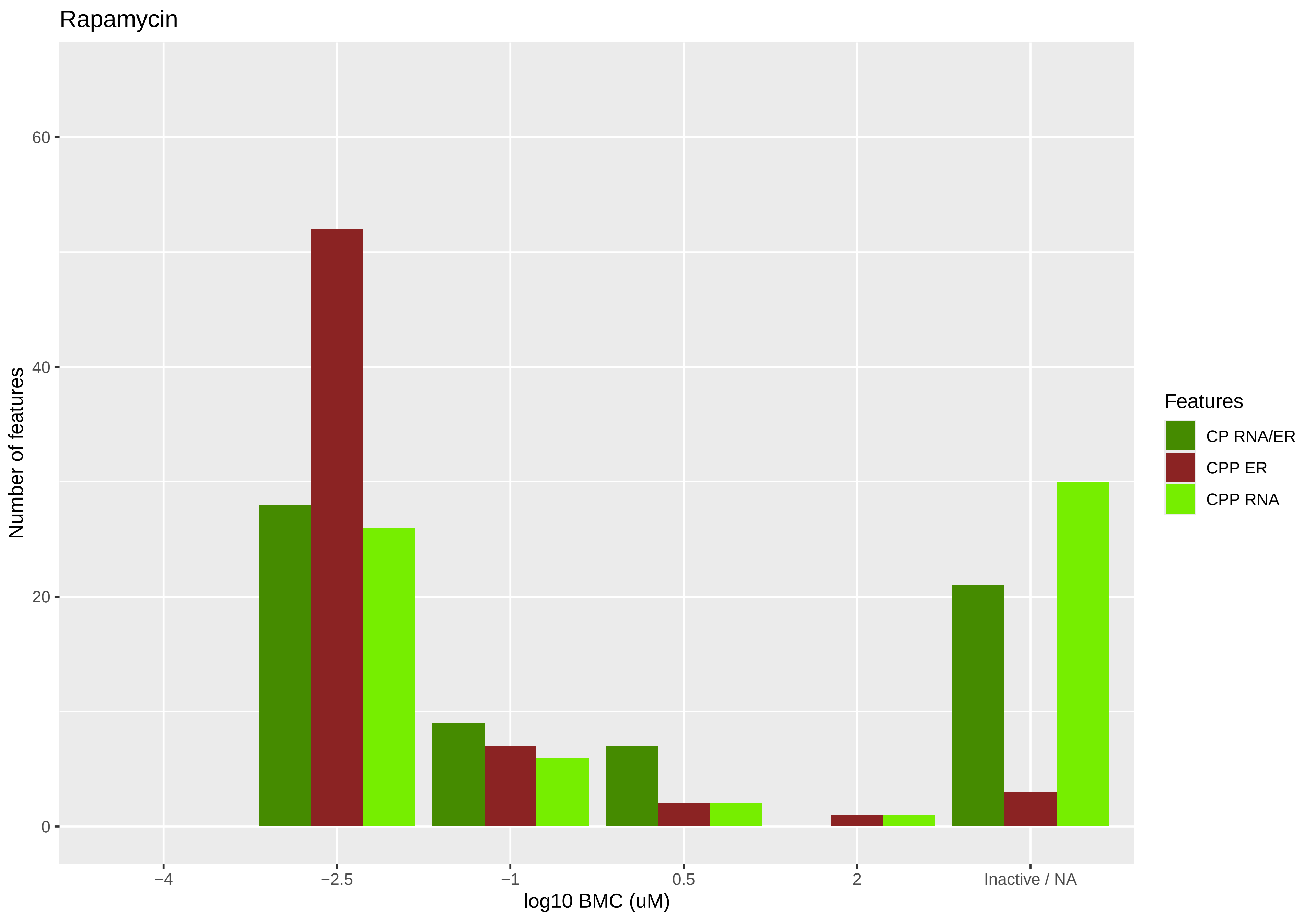

Rapamycin
Number of features
0
20
40
60
−4
−2.5
−1
0.5
2
Inactive / NA
log10 BMC (uM)
Features
CP RNA/ER
CPP ER
CPP RNA

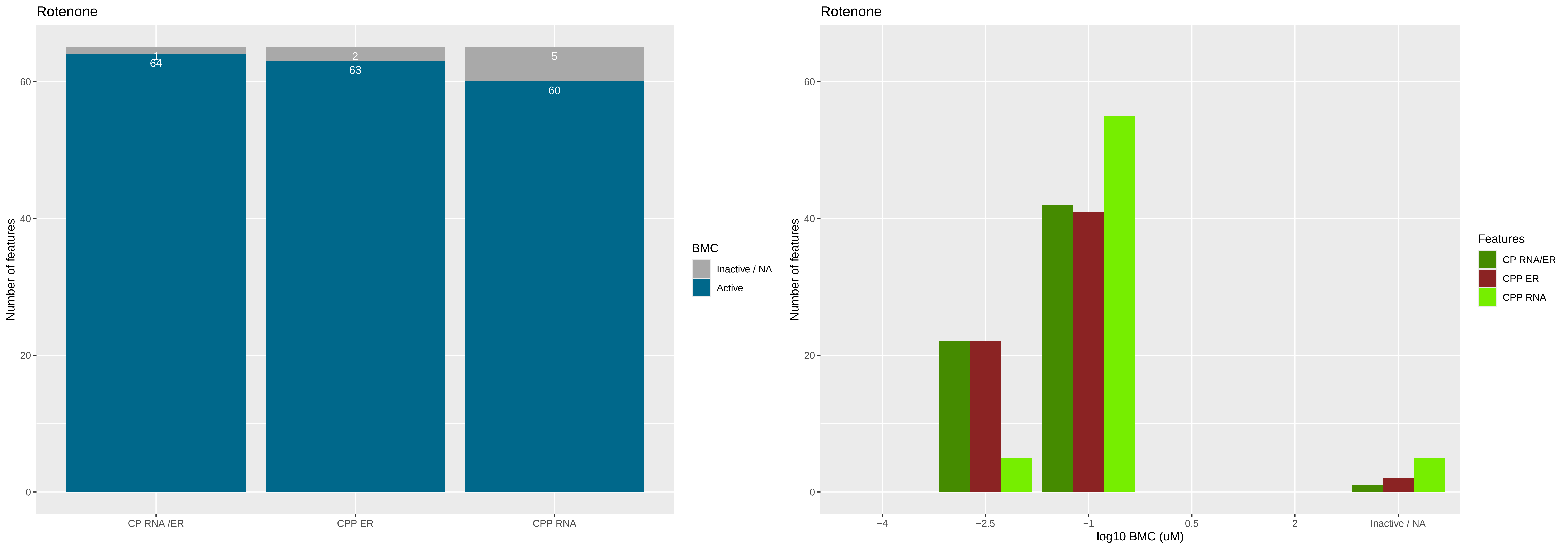
Rotenone
Number of features
1
64
2
63
5
60
CP RNA /ER
CPP ER
CPP RNA
BMC
Inactive / NA
Active
Rotenone
Number of features
log10 BMC (uM)
−4
−2.5
−1
0.5
2
Inactive / NA
Features
CP RNA/ER
CPP ER
CPP RNA

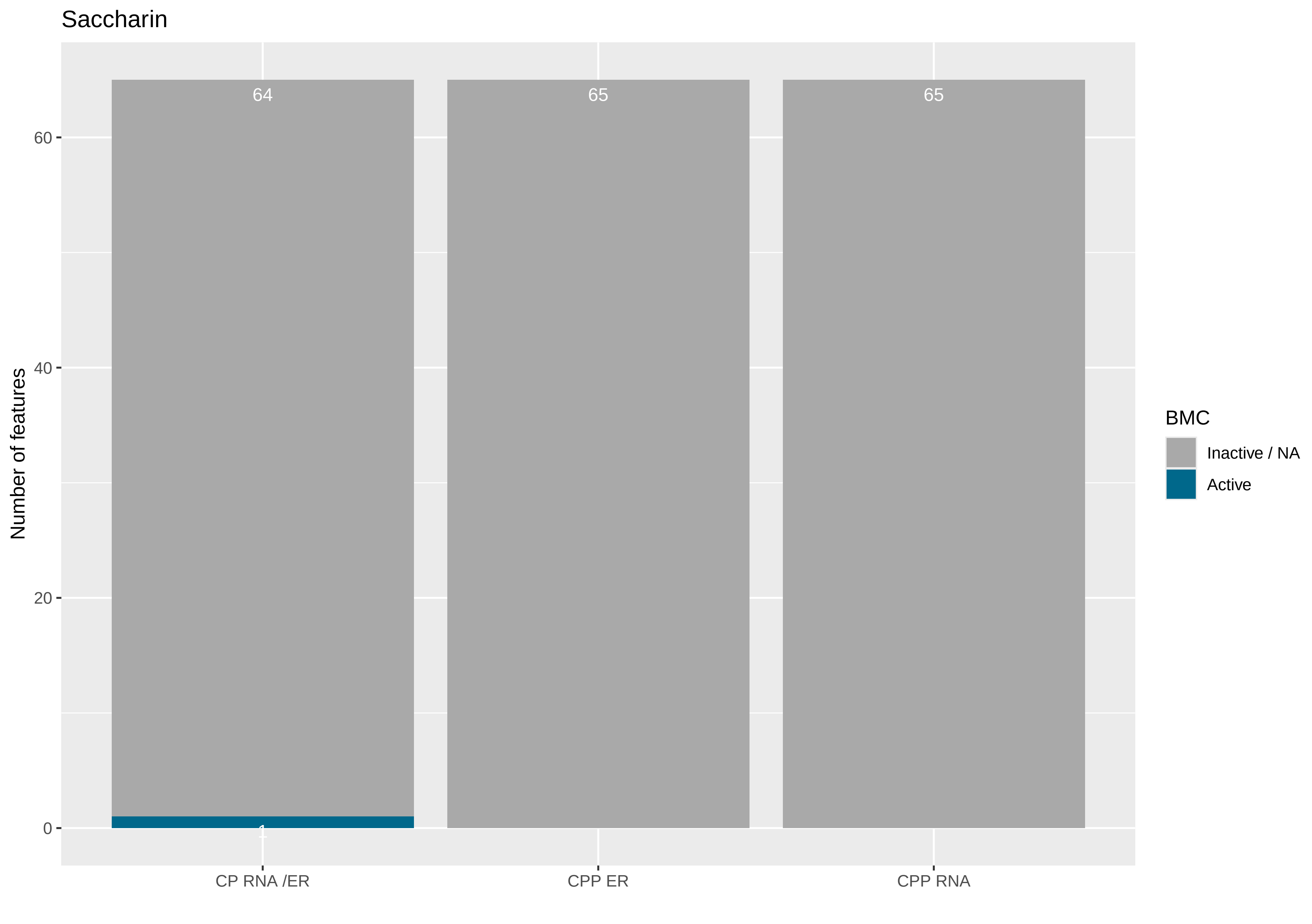
Saccharin
64
65
65
1
Number of features
0
20
40
60
CP RNA /ER
CPP ER
CPP RNA
BMC
Inactive / NA
Active

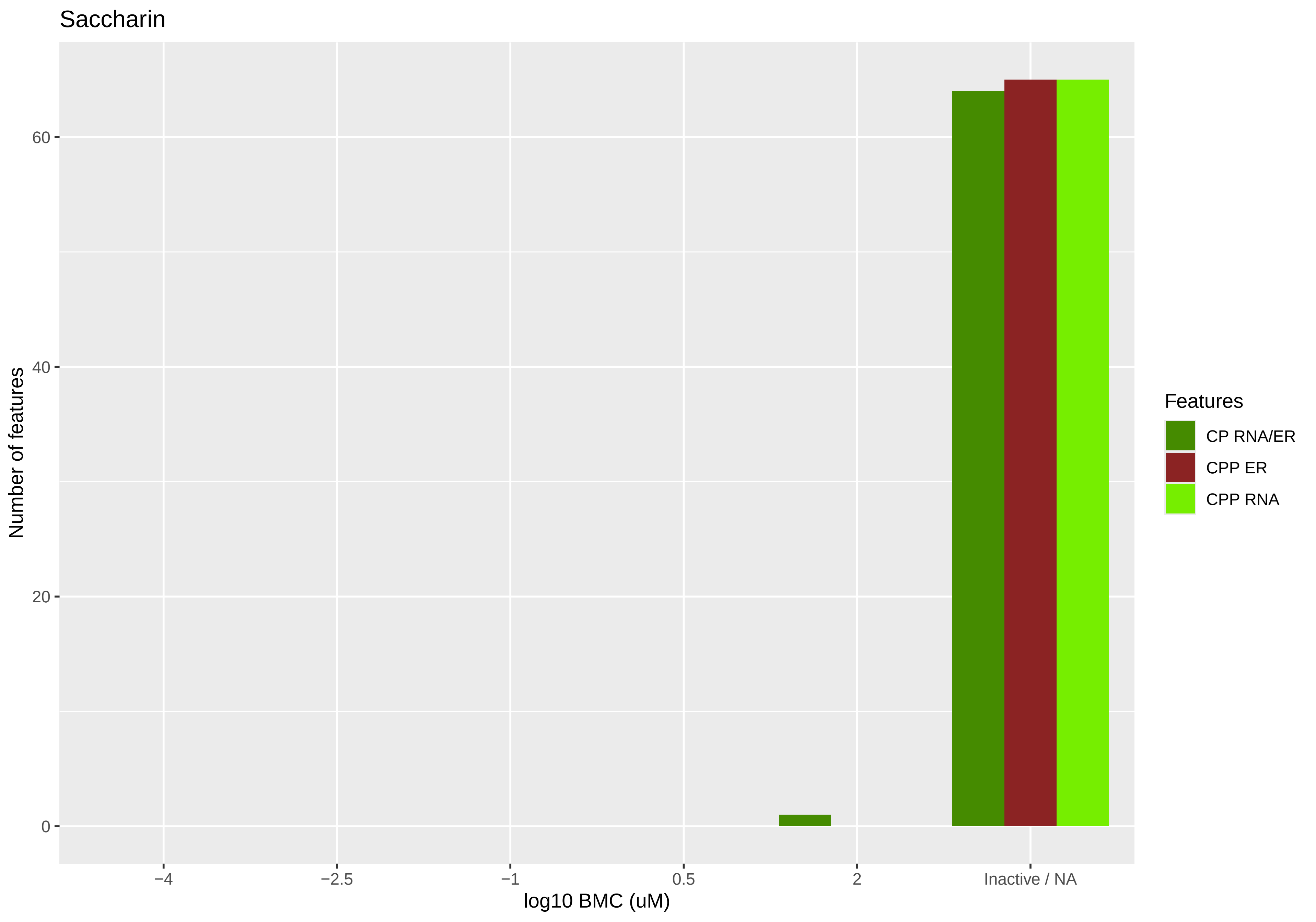
Saccharin
Number of features
0
20
40
60
−4
−2.5
−1
0.5
2
Inactive / NA
log10 BMC (uM)
Features
CP RNA/ER
CPP ER
CPP RNA

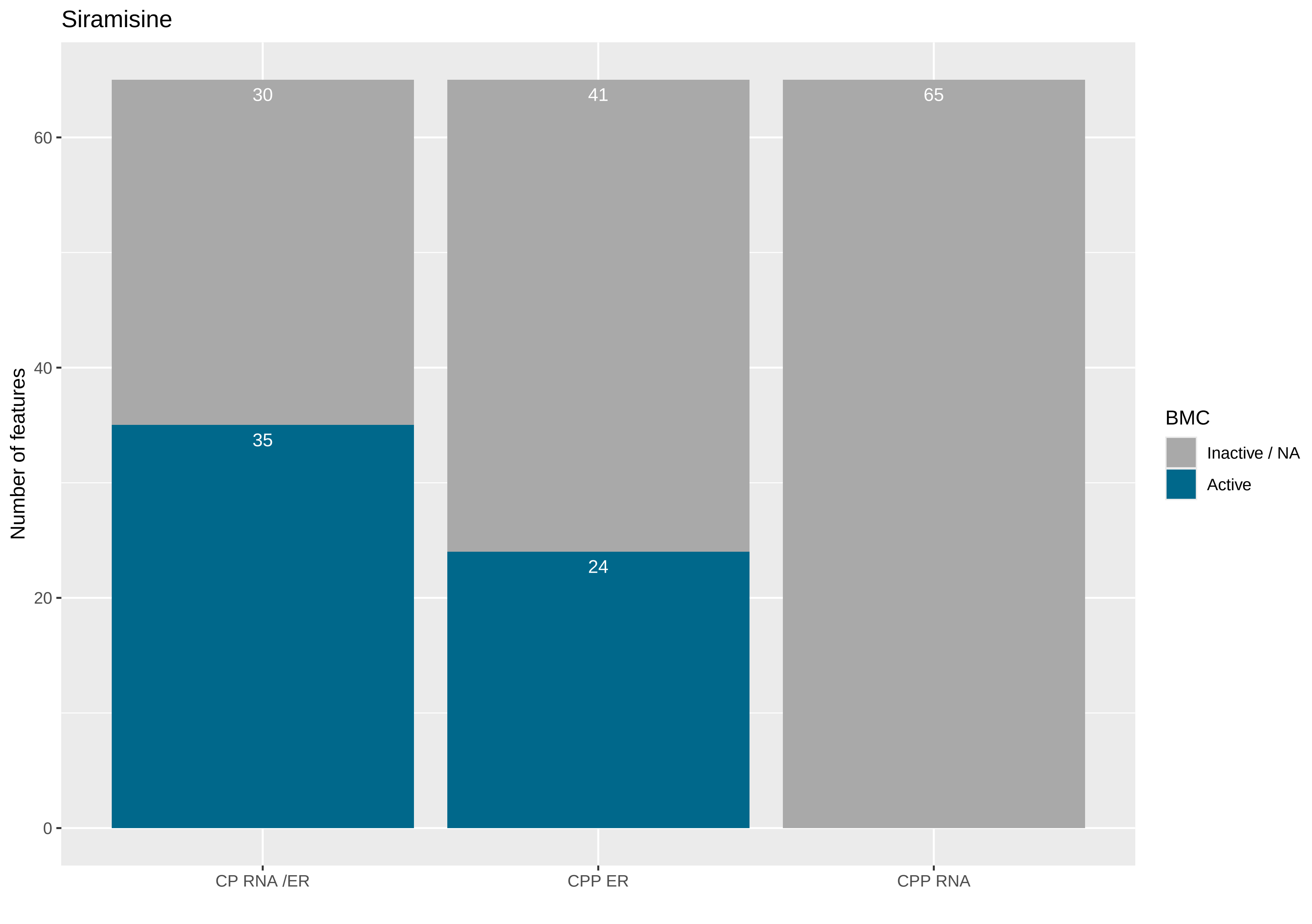

Siramisine
30
41
65
35
24
60
40
20
0
Number of features
CP RNA /ER
CPP ER
CPP RNA
BMC
Inactive / NA
Active

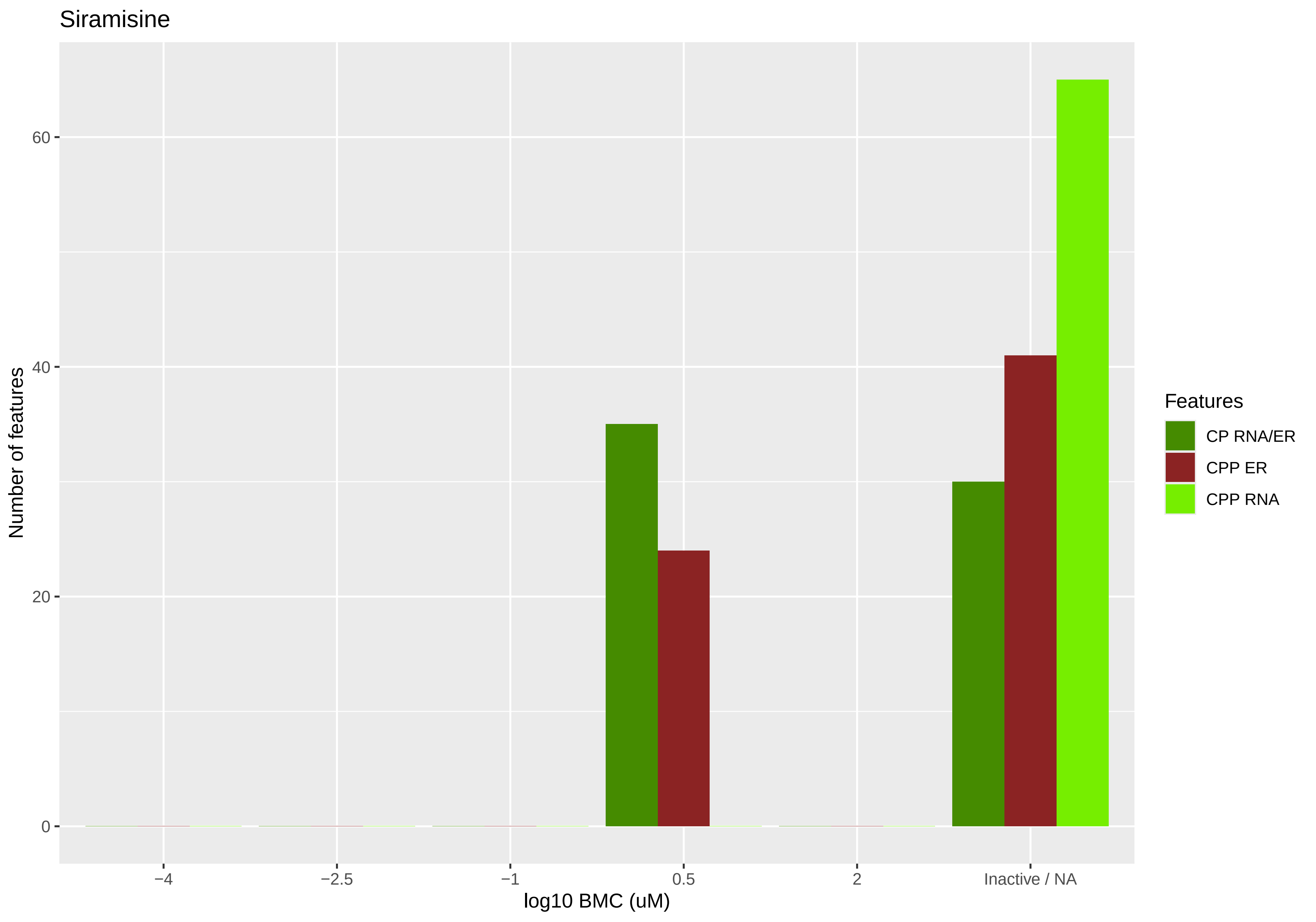

Siramisine
60
40
20
0
Number of features
−4
−2.5
−1
0.5
2
Inactive / NA
log10 BMC (uM)
Features
CP RNA/ER
CPP ER
CPP RNA

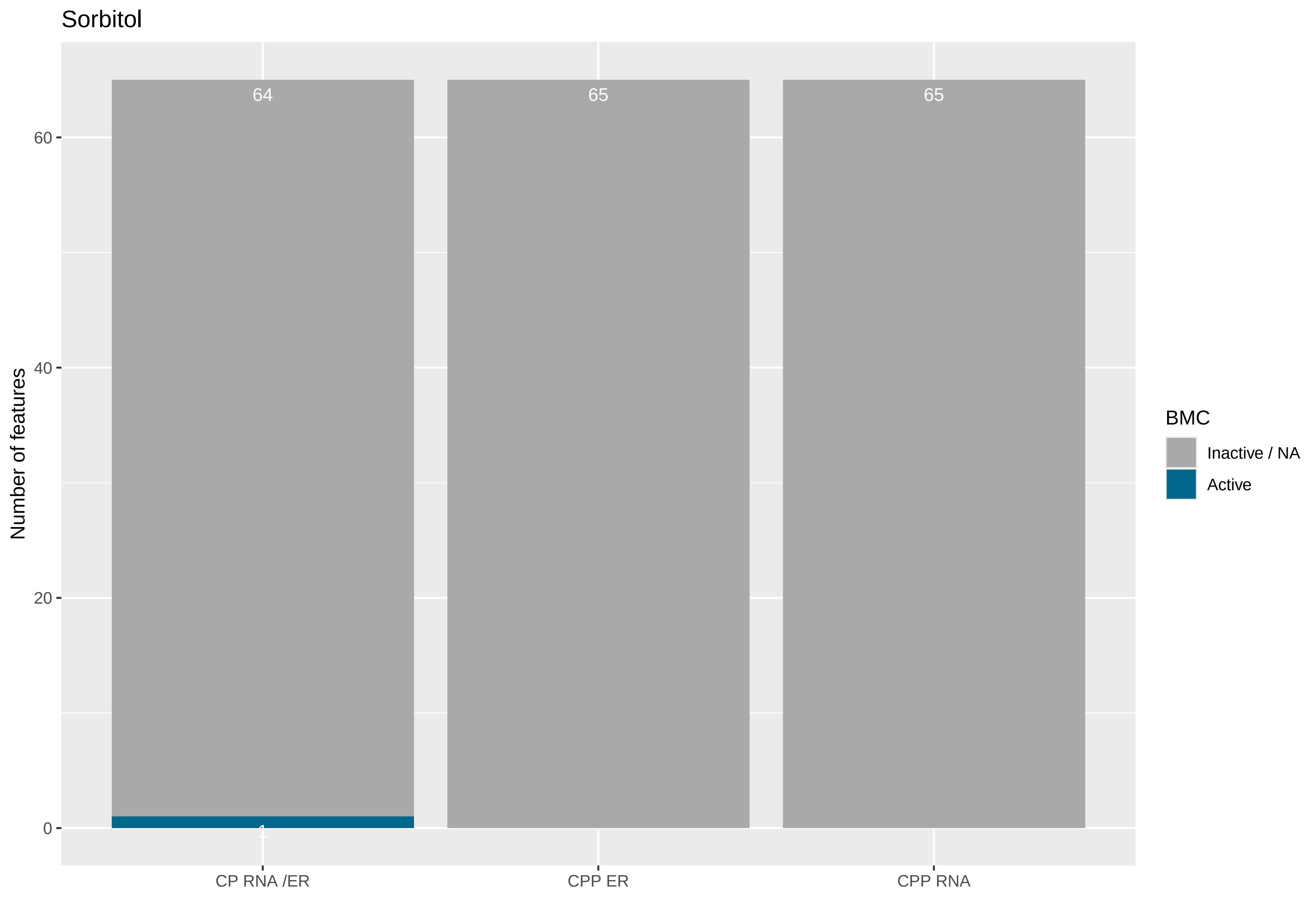

Sorbitol
Number of features
64
65
65
1
0
20
40
60
CP RNA /ER
CPP ER
CPP RNA
BMC
Inactive / NA
Active

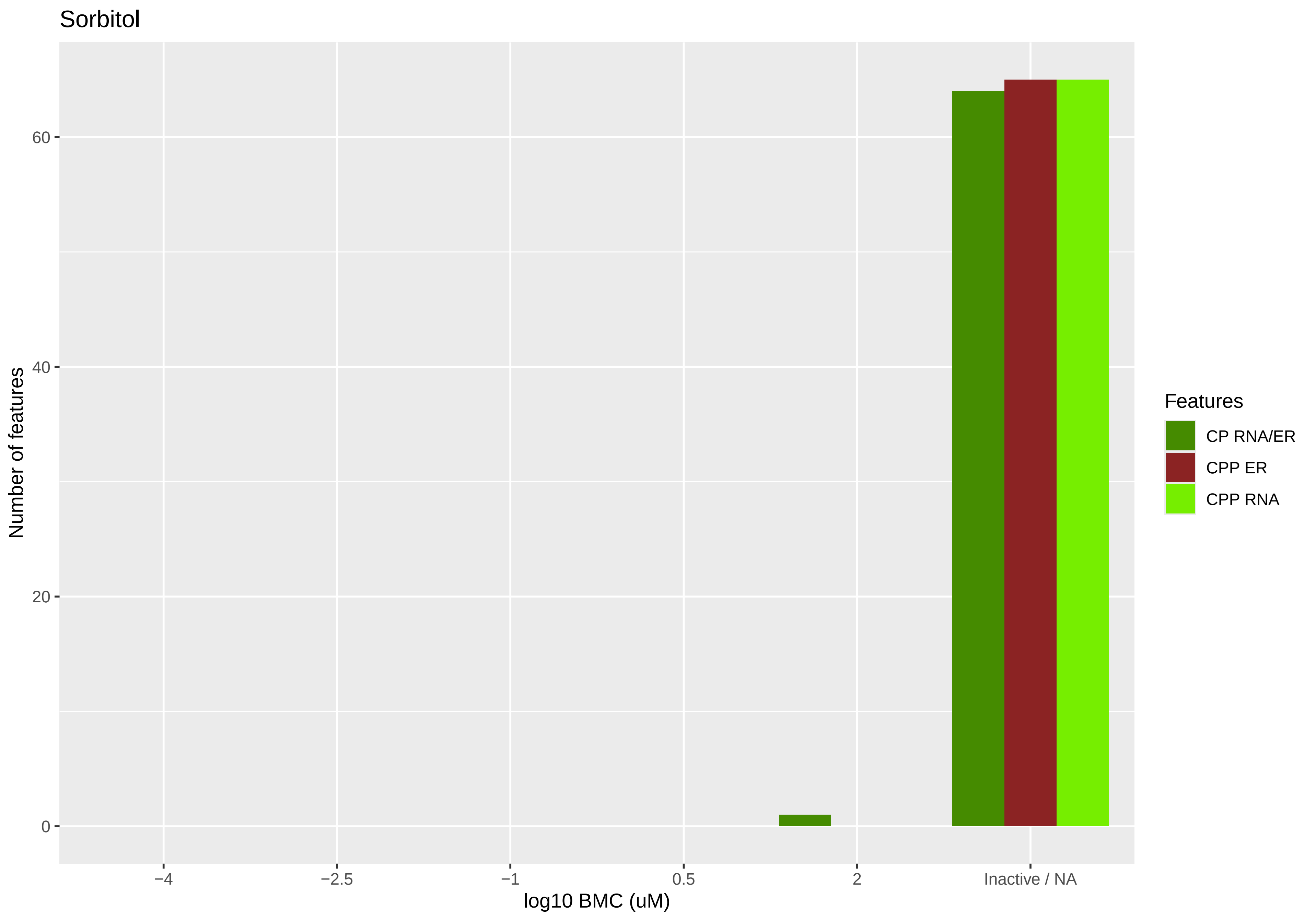

Sorbitol
Number of features
0
20
40
60
−4
−2.5
−1
0.5
2
Inactive / NA
log10 BMC (uM)
Features
CP RNA/ER
CPP ER
CPP RNA

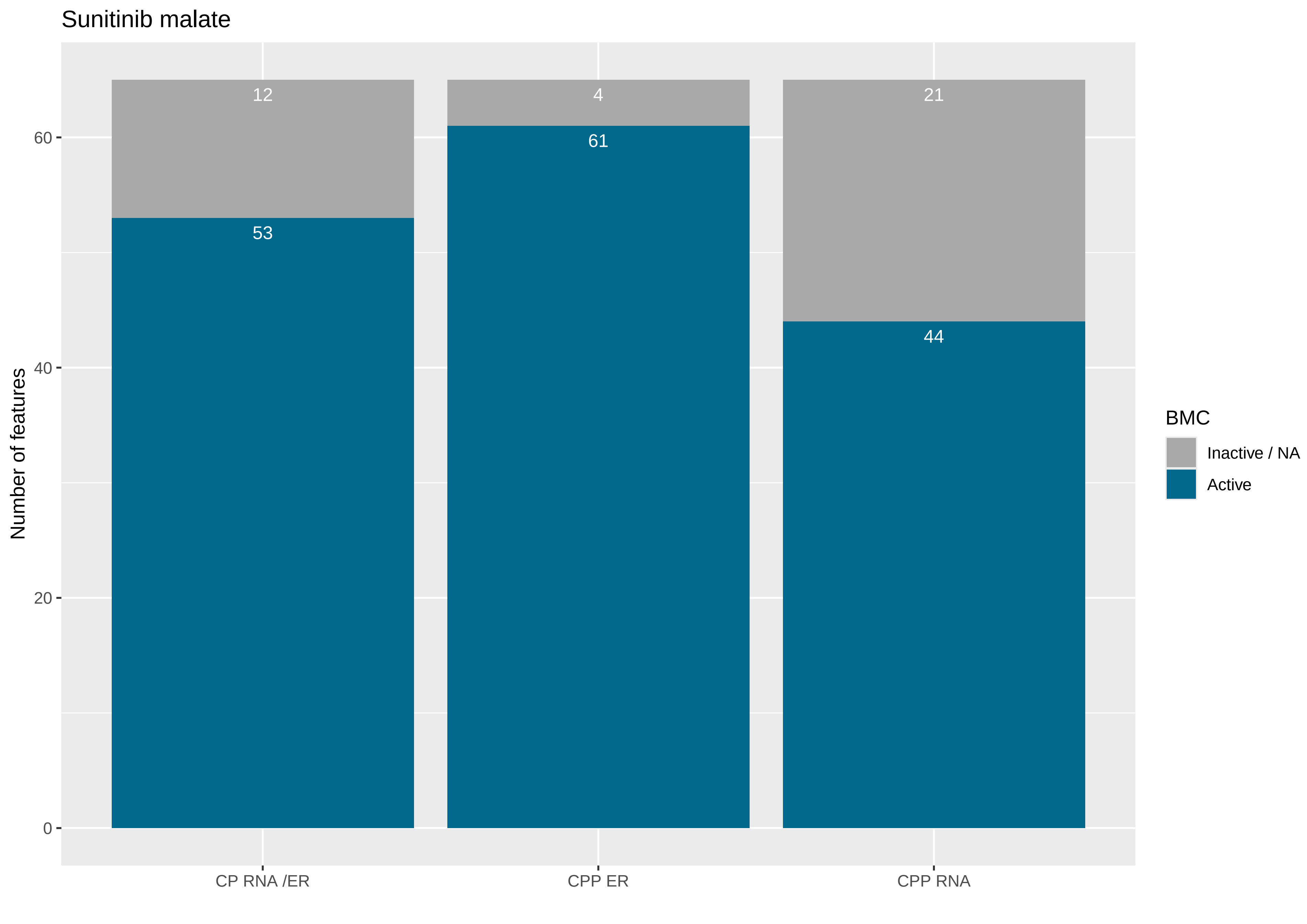
Sunitinib malate
Number of features
BMC
Inactive / NA
Active
12
4
21
53
61
44
0
20
40
60
CP RNA /ER
CPP ER
CPP RNA

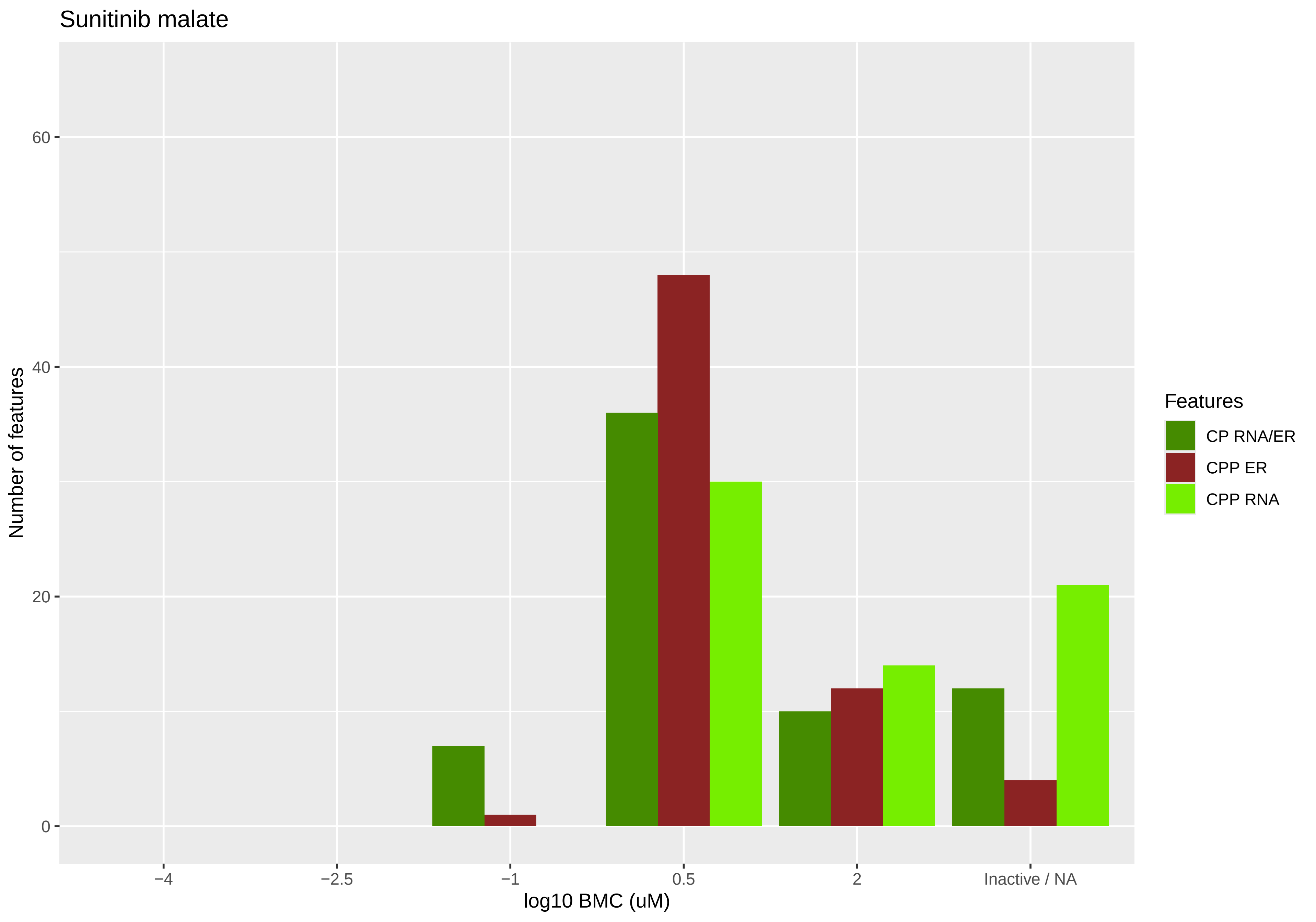
Sunitinib malate
Number of features
log10 BMC (uM)
Features
CP RNA/ER
CPP ER
CPP RNA
0
20
40
60
−4
−2.5
−1
0.5
2
Inactive / NA

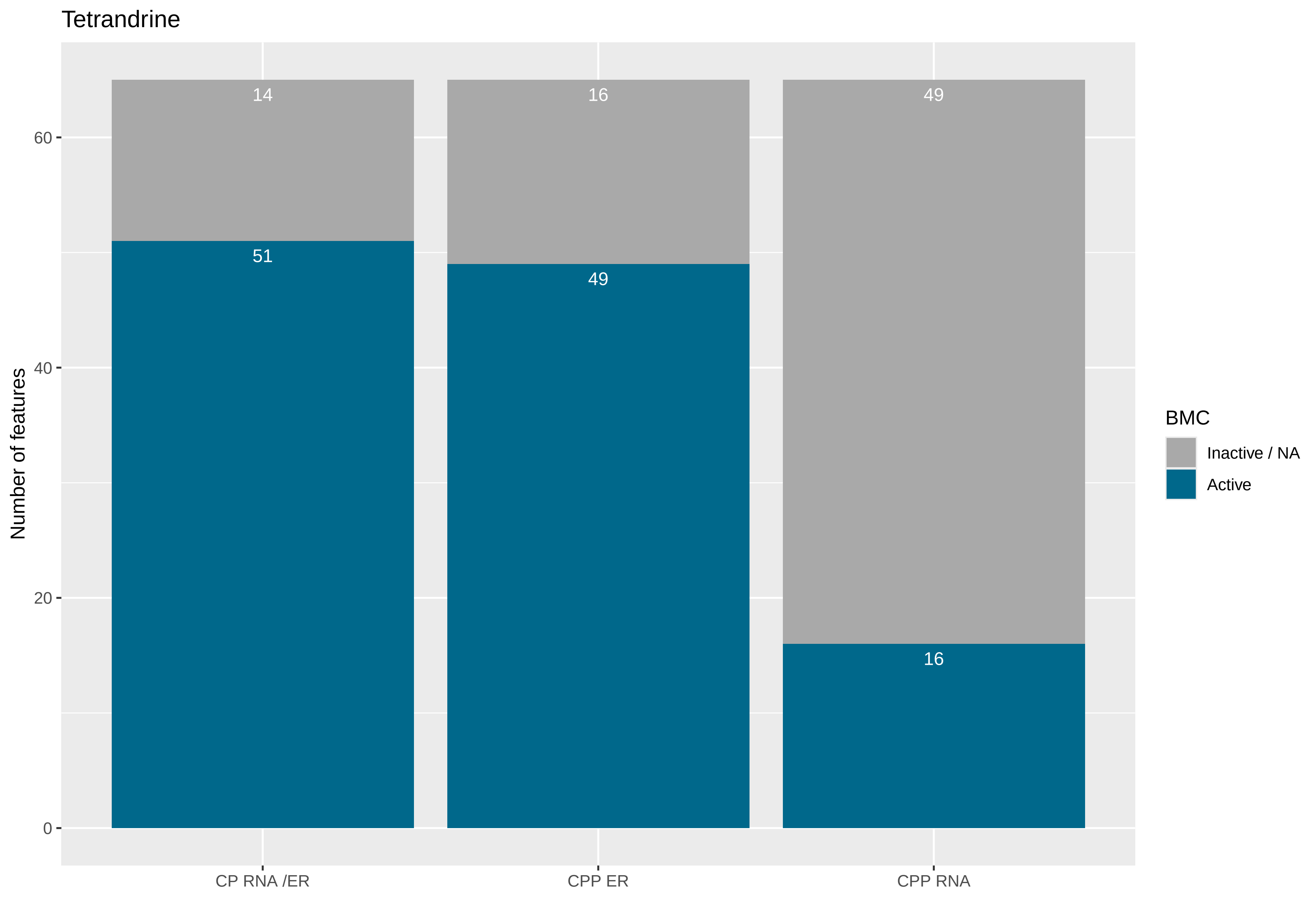

Tetrandrine
Number of features
0
20
40
60
14
51
16
49
49
16
CP RNA /ER
CPP ER
CPP RNA
BMC
Inactive / NA
Active

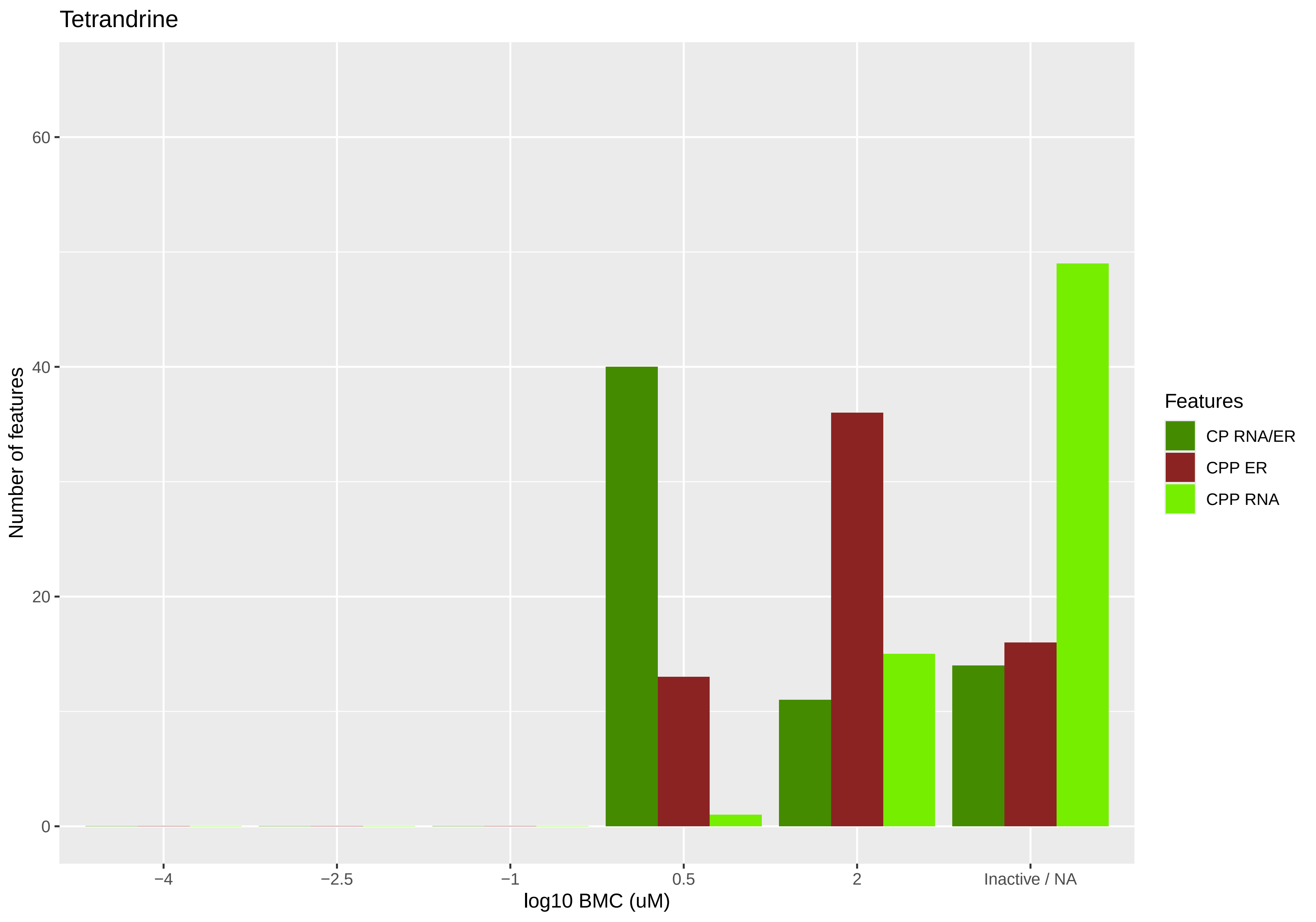

Tetrandrine
Number of features
0
20
40
60
−4
−2.5
−1
0.5
2
Inactive / NA
log10 BMC (uM)
Features
CP RNA/ER
CPP ER
CPP RNA
