# Supplementary material for: Cell Painting PLUS: expanding the multiplexing capacity of Cell Painting-based phenotypic profiling using iterative staining-elution cycles
Source: Nat Commun. 2025 Apr 24;16:3857. doi: 10.1038/s41467-025-58765-8 (PMC12022024; doi:10.1038/s41467-025-58765-8)
Supplement: Supplementary file 3 — Supplementary Data [file 41467_2025_58765_MOESM3_ESM.zip › Supplementary Data/Supplementary Data_9_Fig2C_RelCellNumber_HepG2.pdf]

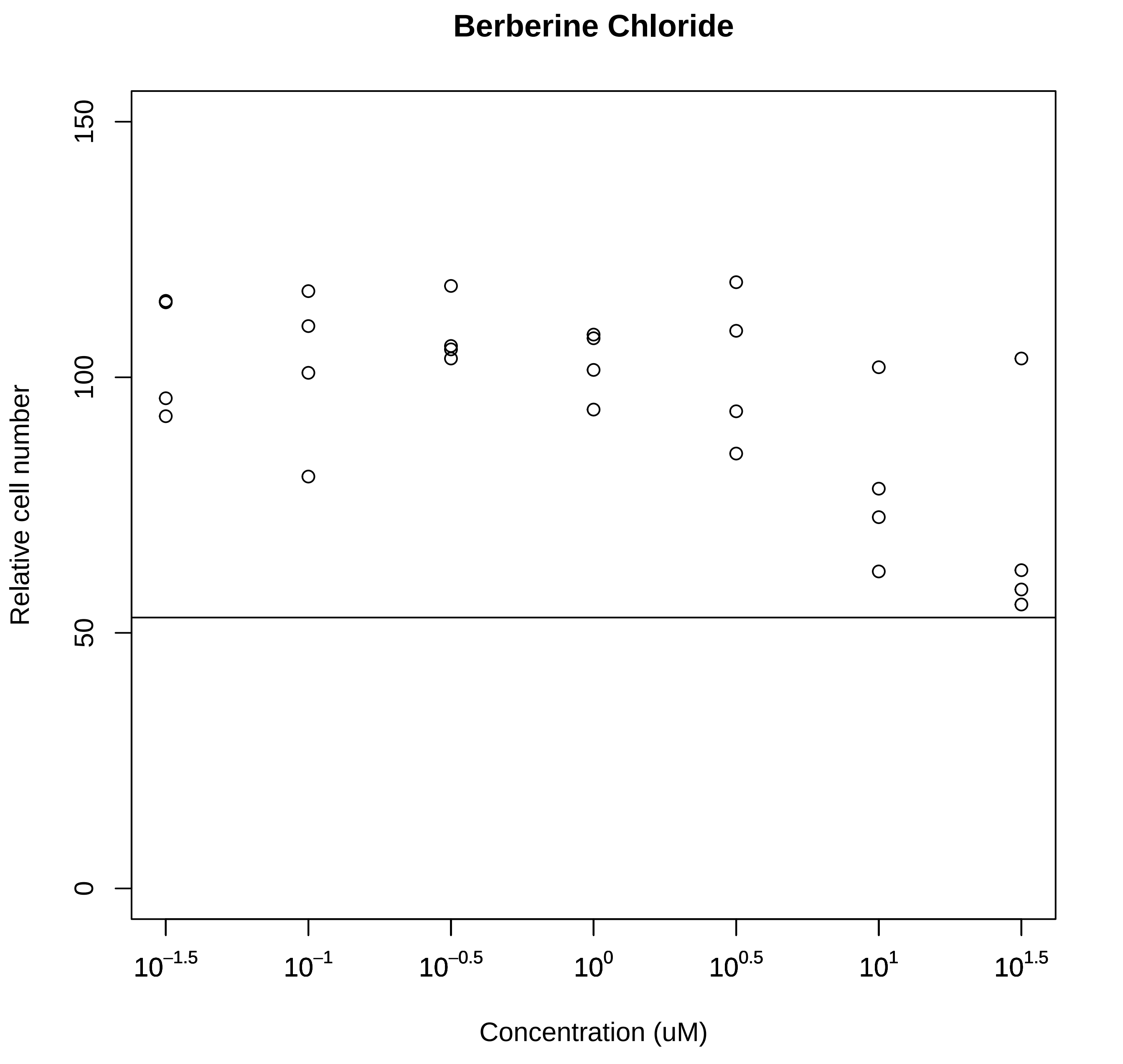
Berberine Chloride
Relative cell number
150
100
50
0
$10^{-1.5}$
10^{-1}
$10^{-0.5}$
10^{0}
$10^{0.5}$
10^{1}
$10^{1.5}$
Concentration (uM)

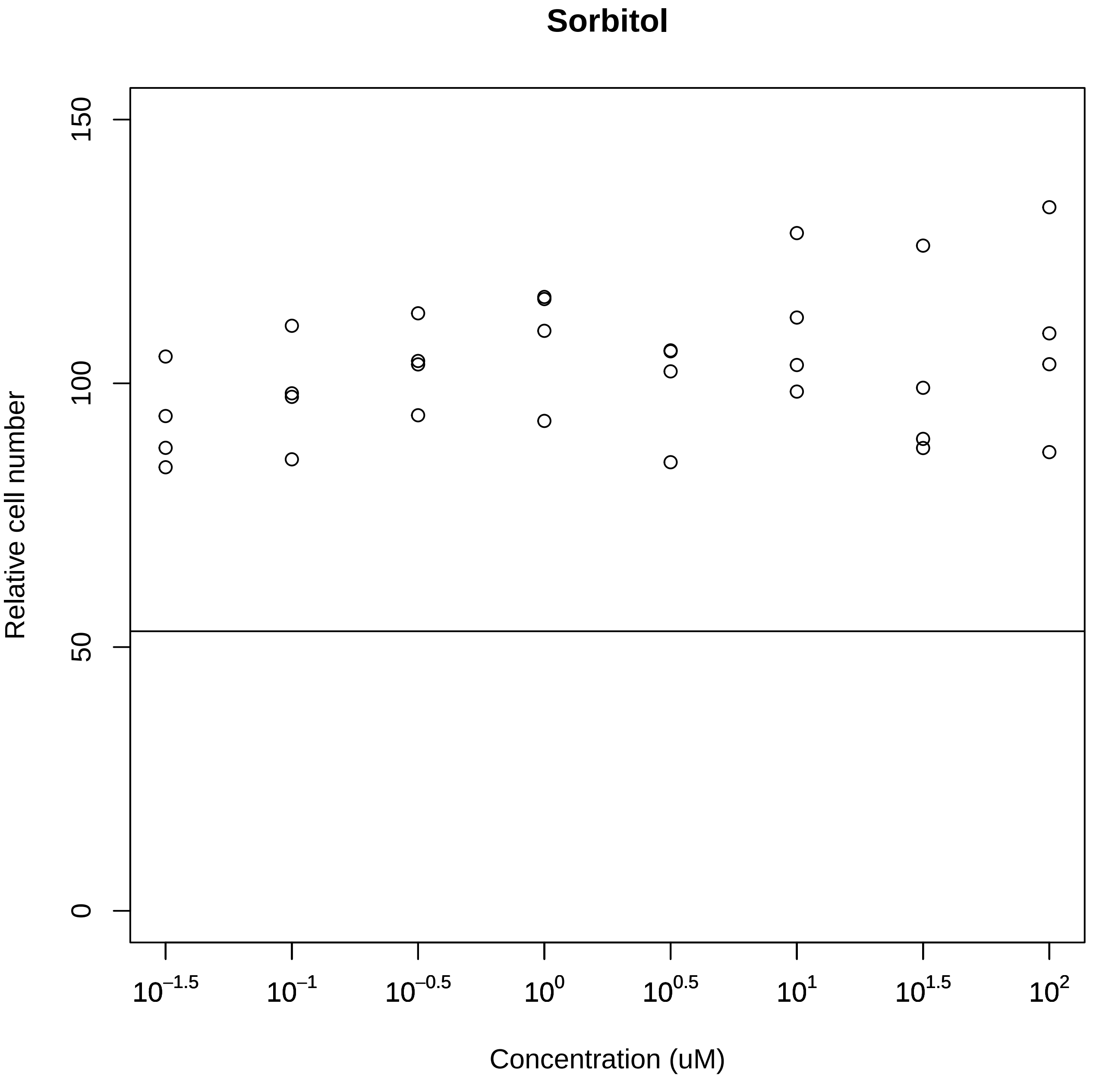

Sorbitol
Relative cell number
0
50
100
150
$10^{-1.5}$
10^{-1}
$10^{-0.5}$
10^{0}
$10^{0.5}$
10^{1}
$10^{1.5}$
10^{2}
Concentration (uM)

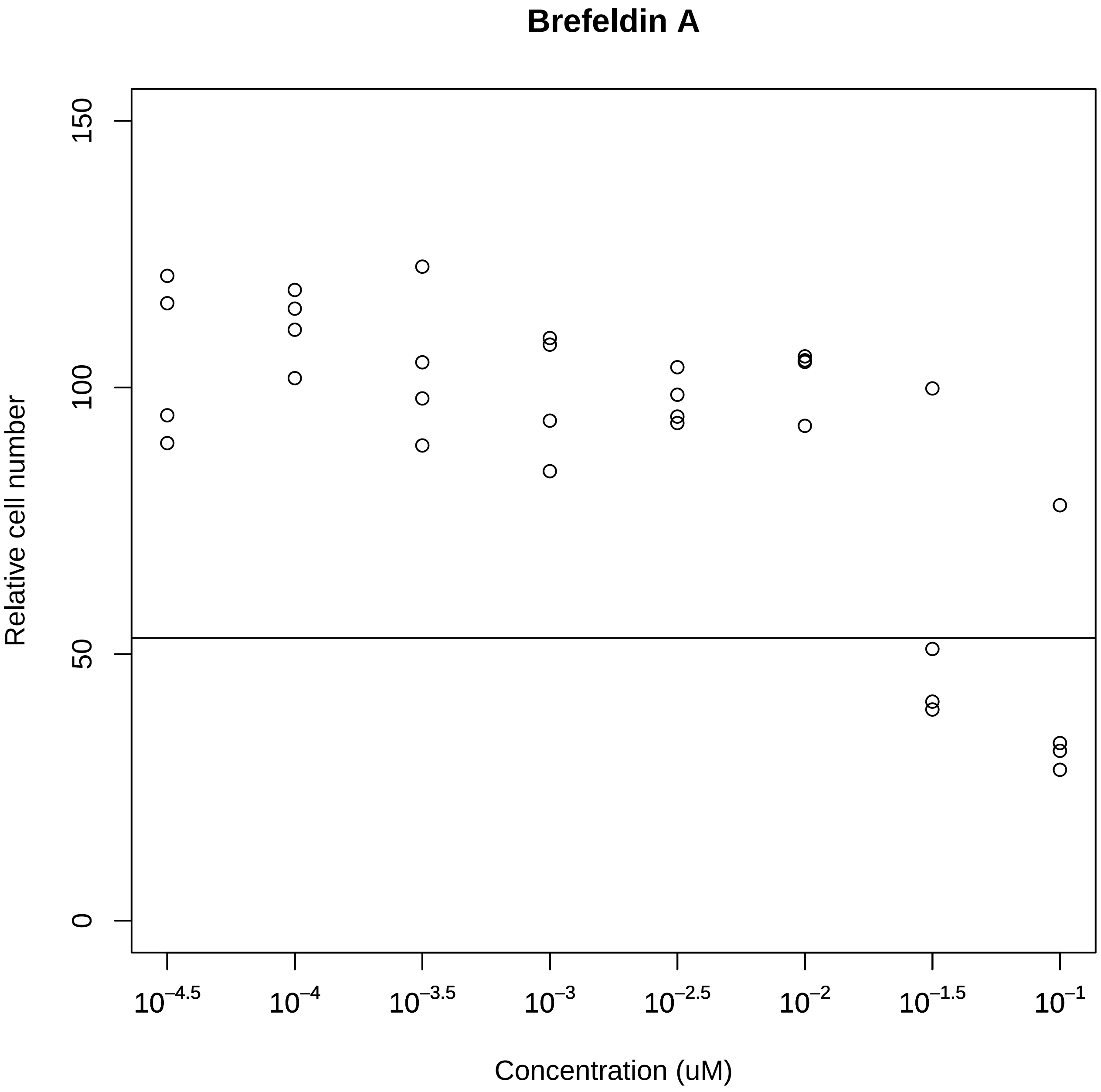

Brefeldin A
Relative cell number
0
50
100
150
$10^{-4.5}$
10^{-4}
$10^{-3.5}$
10^{-3}
$10^{-2.5}$
10^{-2}
$10^{-1.5}$
10^{-1}
Concentration (uM)

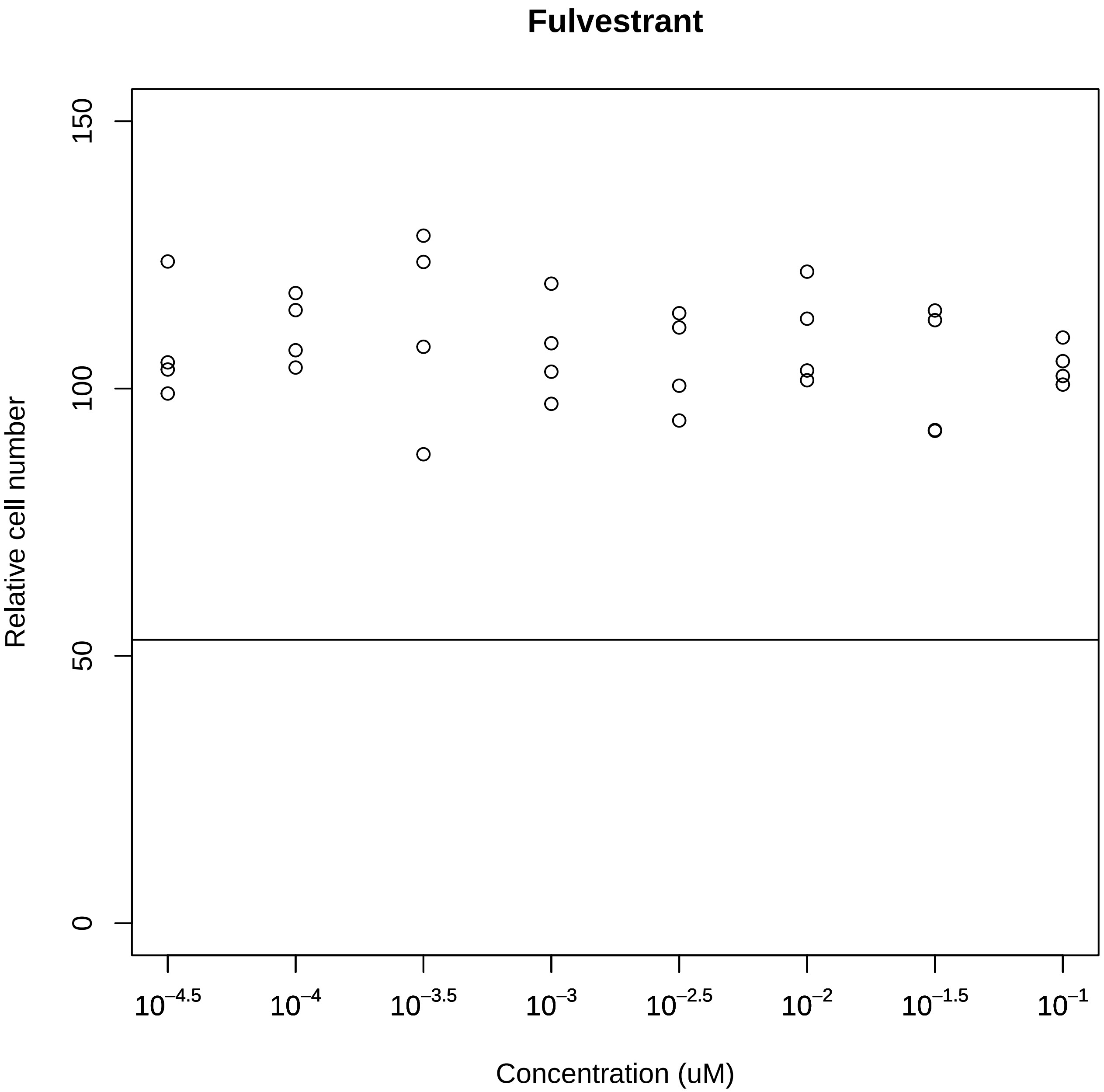
Fulvestrant
Relative cell number
0
50
100
150
$10^{-4.5}$
10^{-4}
$10^{-3.5}$
10^{-3}
$10^{-2.5}$
10^{-2}
$10^{-1.5}$
10^{-1}
Concentration (uM)

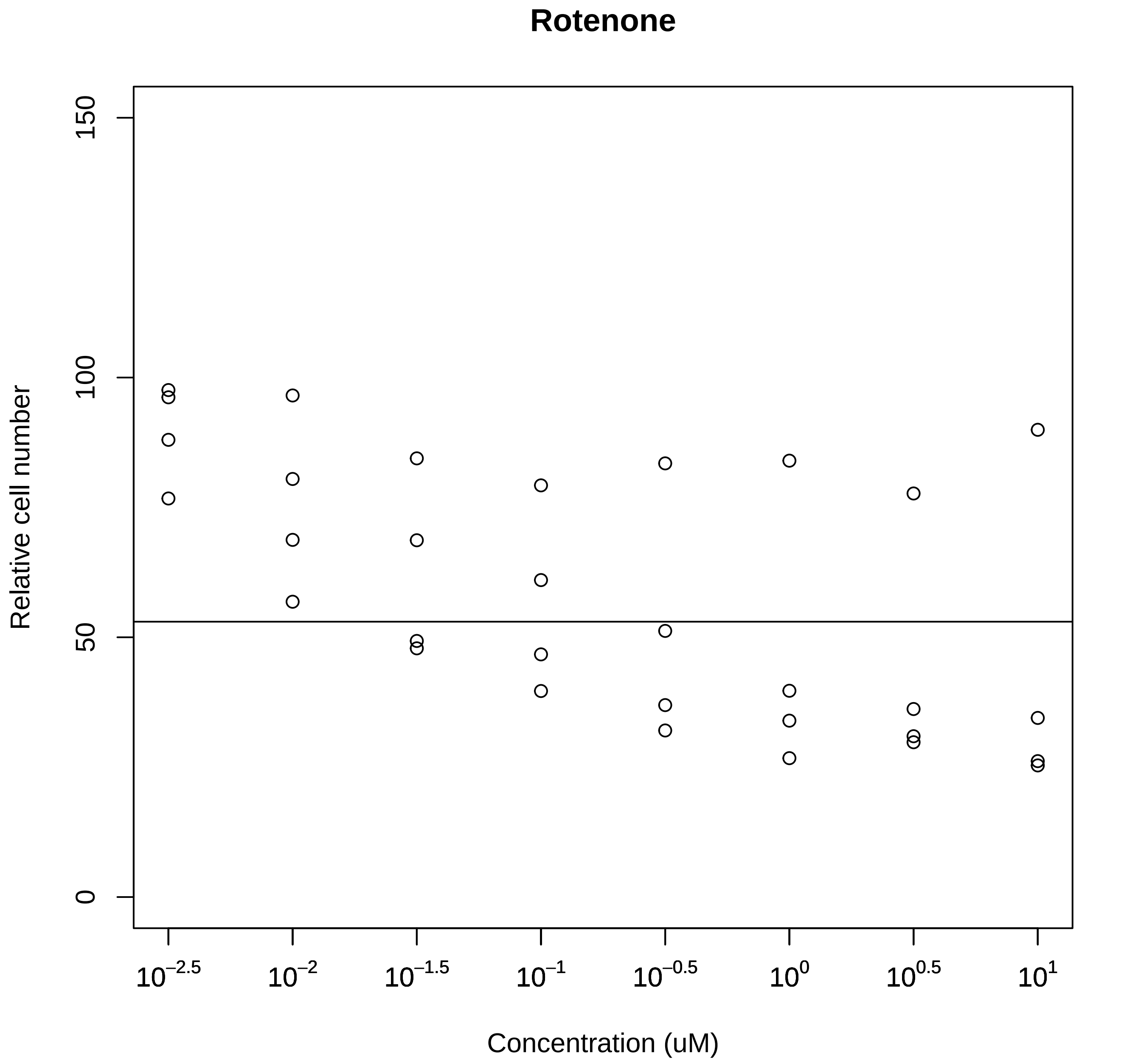

Rotenone
Relative cell number
0
50
100
150
$10^{-2.5}$
10^{-2}
$10^{-1.5}$
10^{-1}
$10^{-0.5}$
10^{0}
$10^{0.5}$
10^{1}
Concentration (uM)

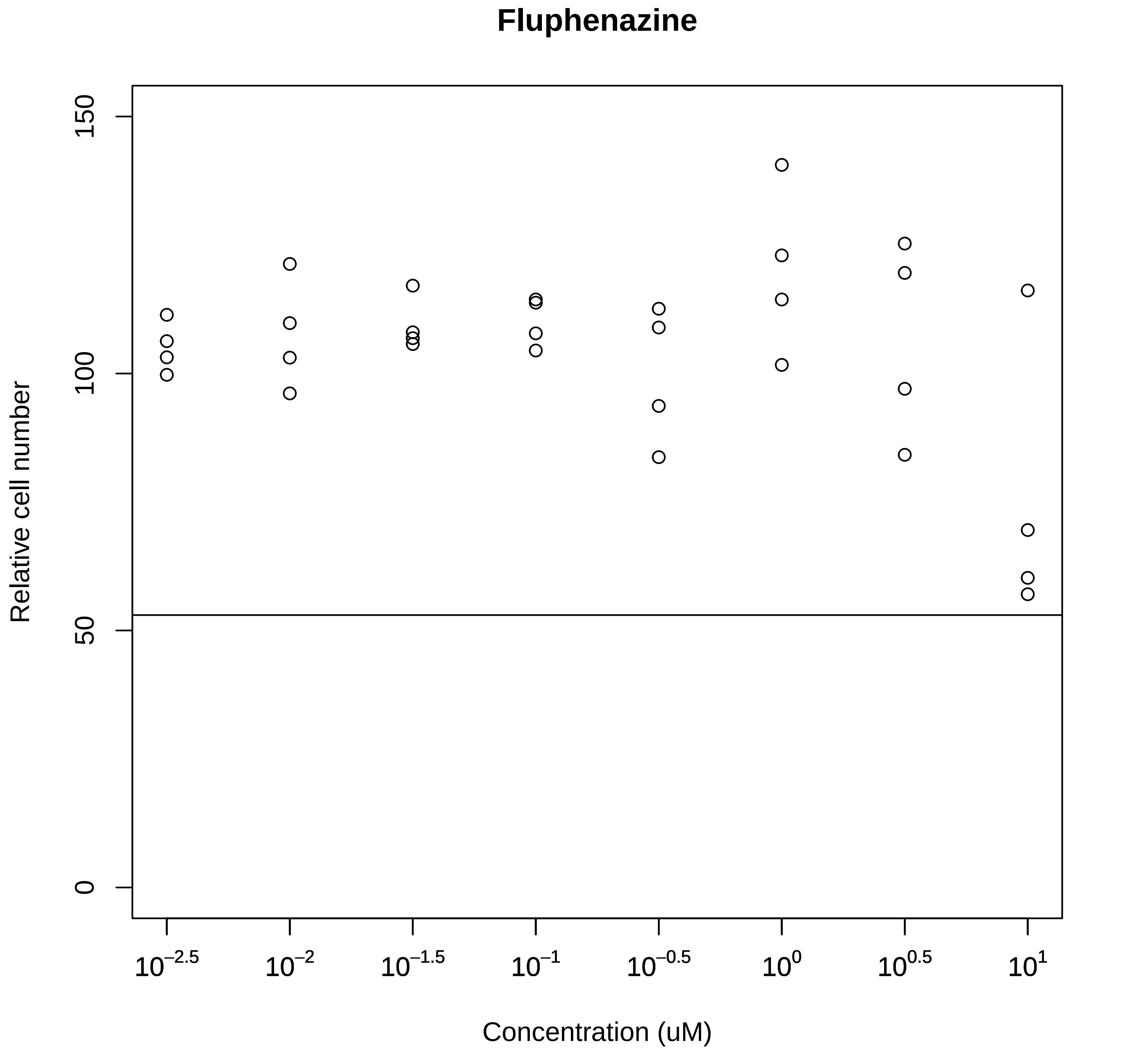

Fluphenazine
Relative cell number
150
100
50
0
$10^{-2.5}$
10^{-2}
$10^{-1.5}$
10^{-1}
$10^{-0.5}$
10^{0}
$10^{0.5}$
10^{1}
Concentration (uM)

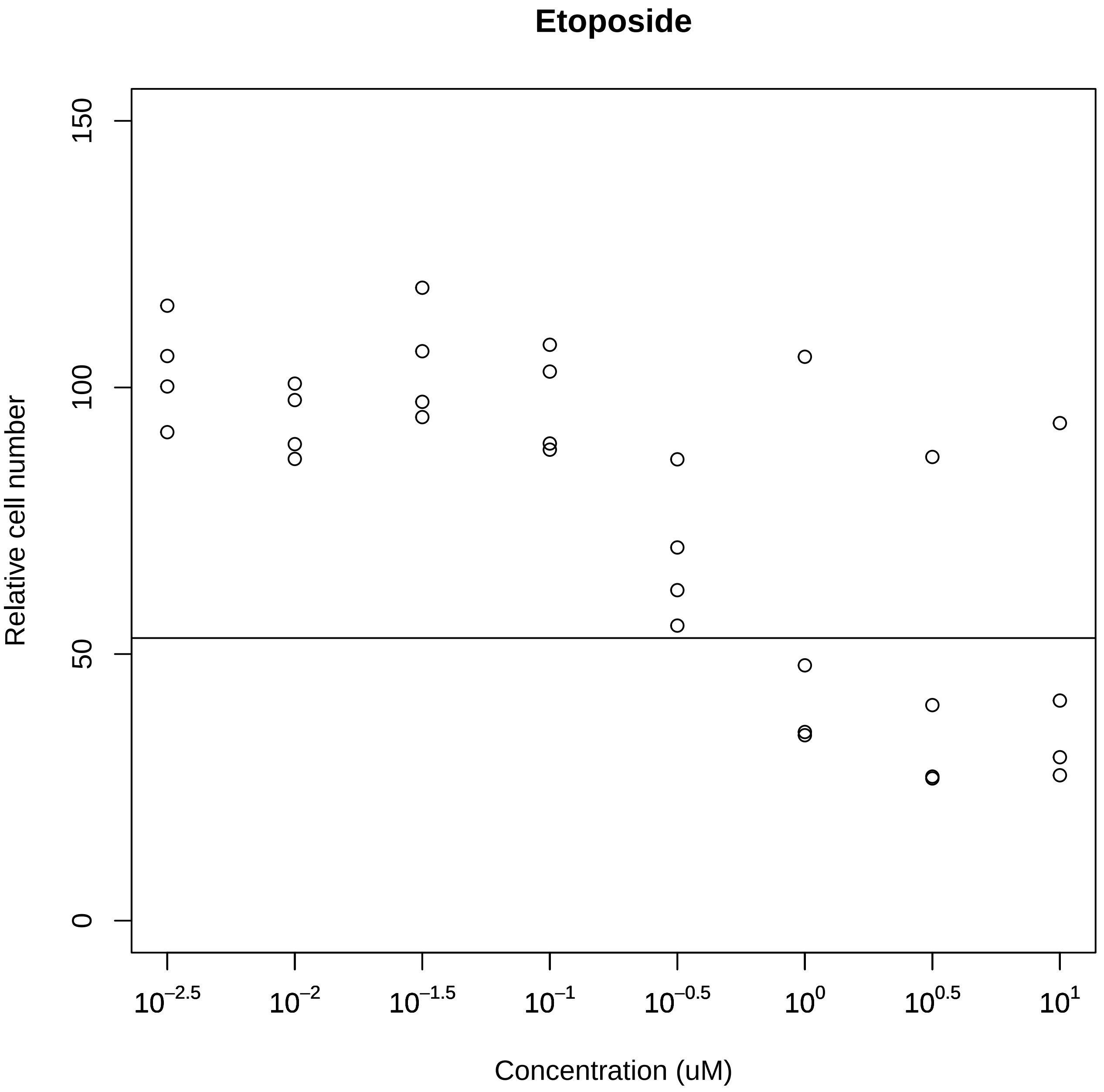

Etoposide
150
100
50
0
Relative cell number
$10^{-2.5}$
10^{-2}
$10^{-1.5}$
10^{-1}
$10^{-0.5}$
10^{0}
$10^{0.5}$
10^{1}
Concentration (uM)

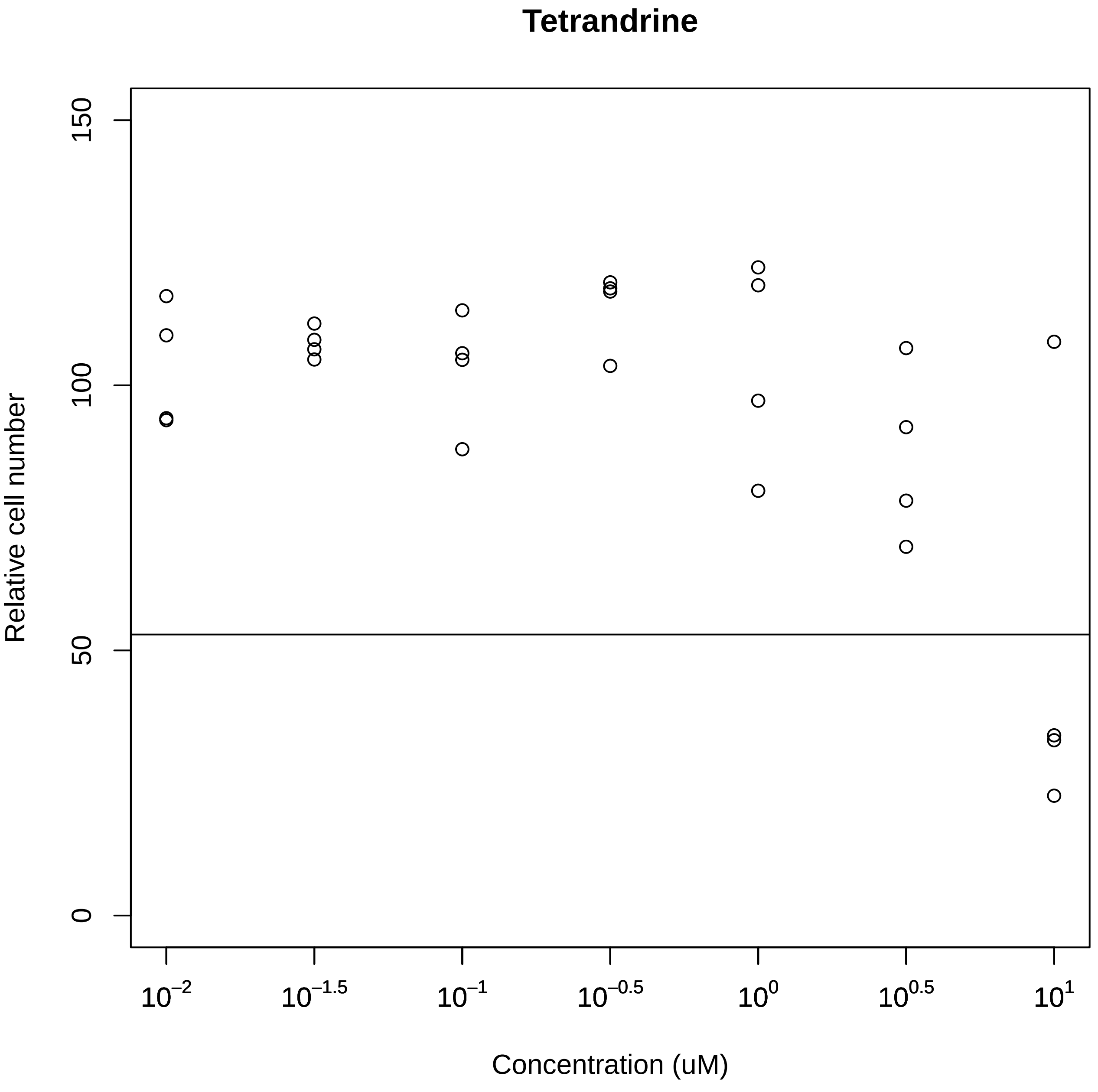

Tetrandrine
Relative cell number
150
100
50
0
10^{-2}
$10^{-1.5}$
10^{-1}
$10^{-0.5}$
10^{0}
$10^{0.5}$
10^{1}
Concentration (uM)

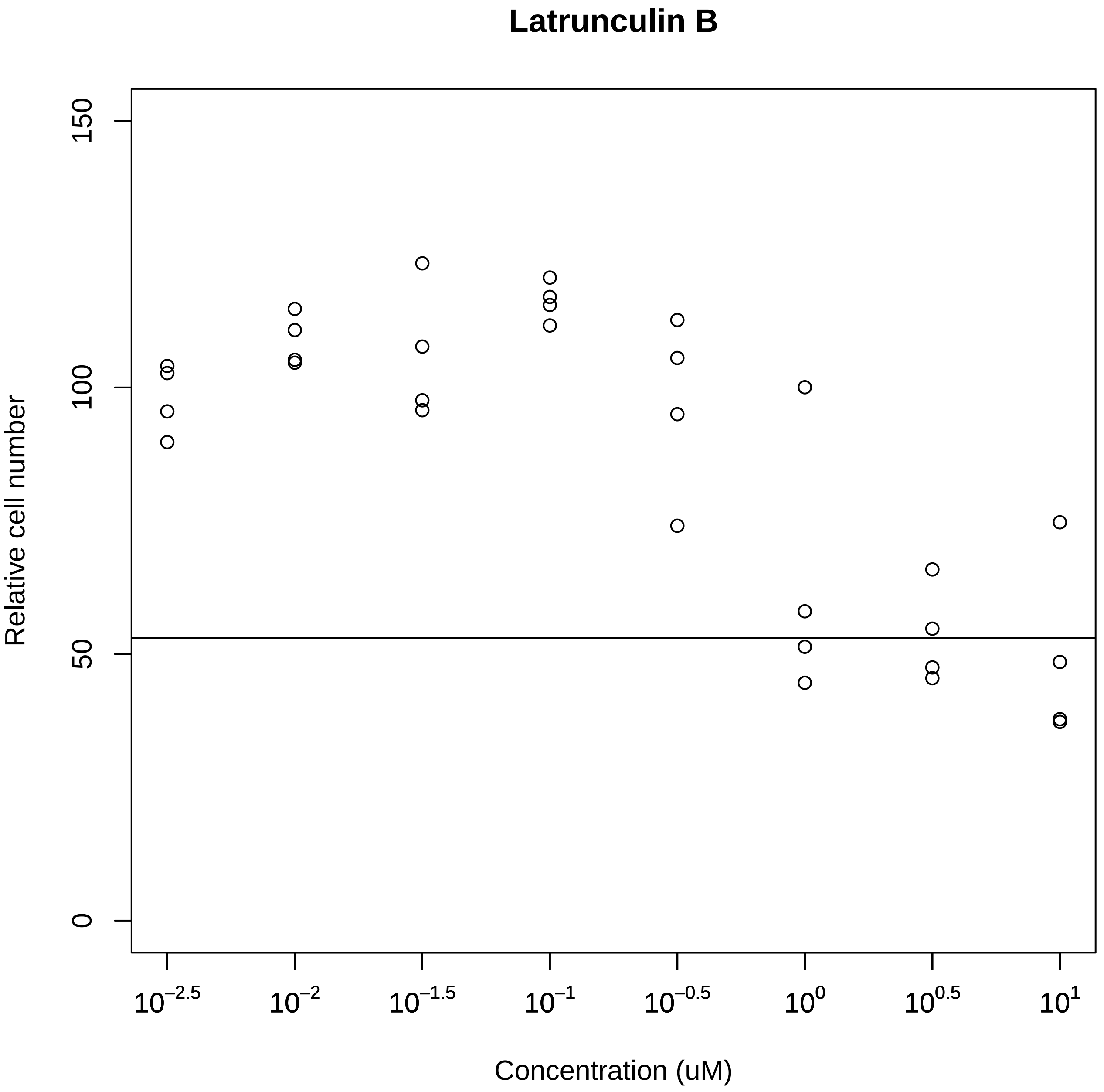

Latrunculin B
Relative cell number
150
100
50
0
$10^{-2.5}$
10^{-2}
$10^{-1.5}$
10^{-1}
$10^{-0.5}$
10^{0}
$10^{0.5}$
10^{1}
Concentration (uM)

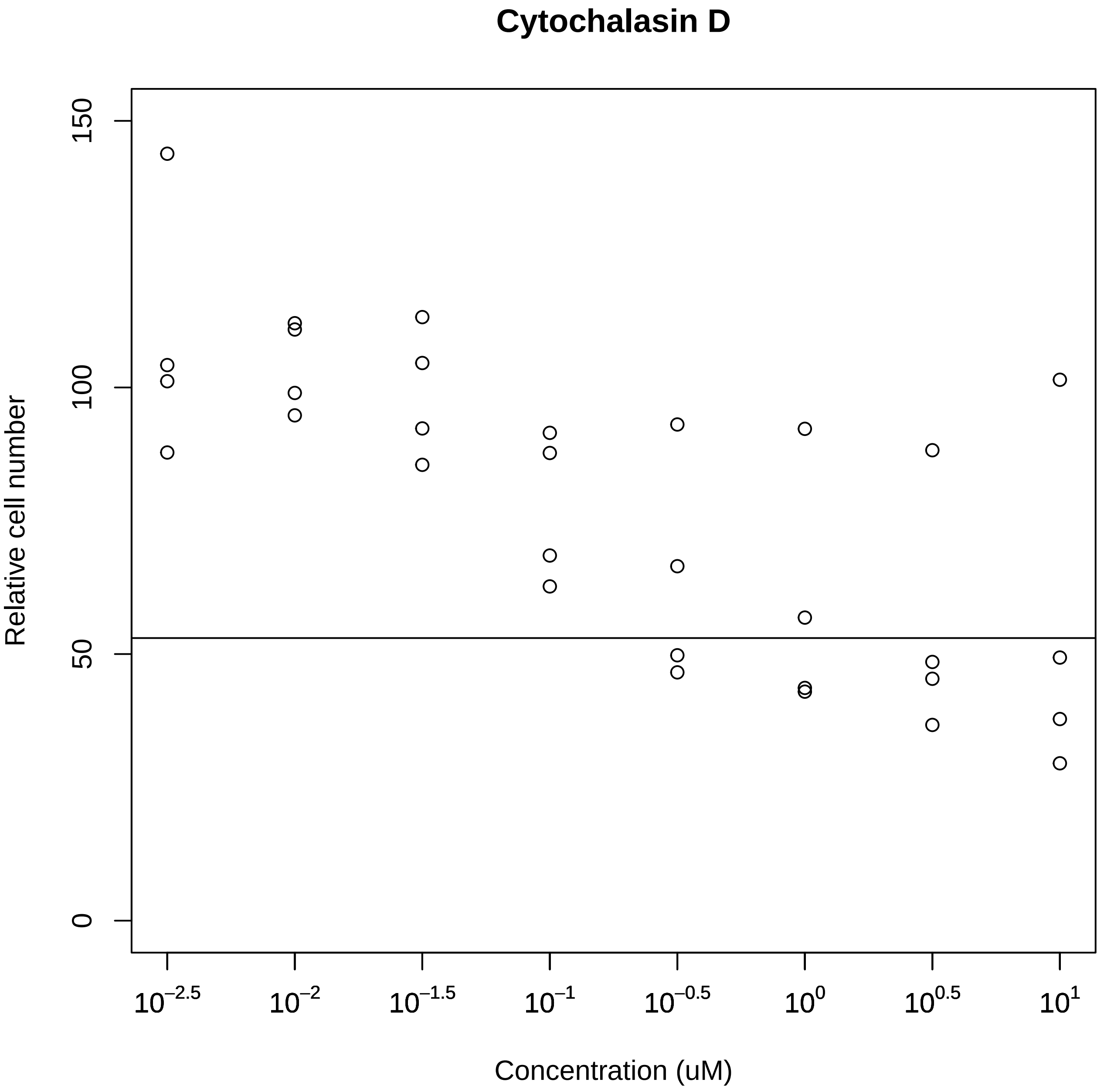

Cytochalasin D
Relative cell number
150
100
50
0
$10^{-2.5}$
10^{-2}
$10^{-1.5}$
10^{-1}
$10^{-0.5}$
10^{0}
$10^{0.5}$
10^{1}
Concentration (uM)

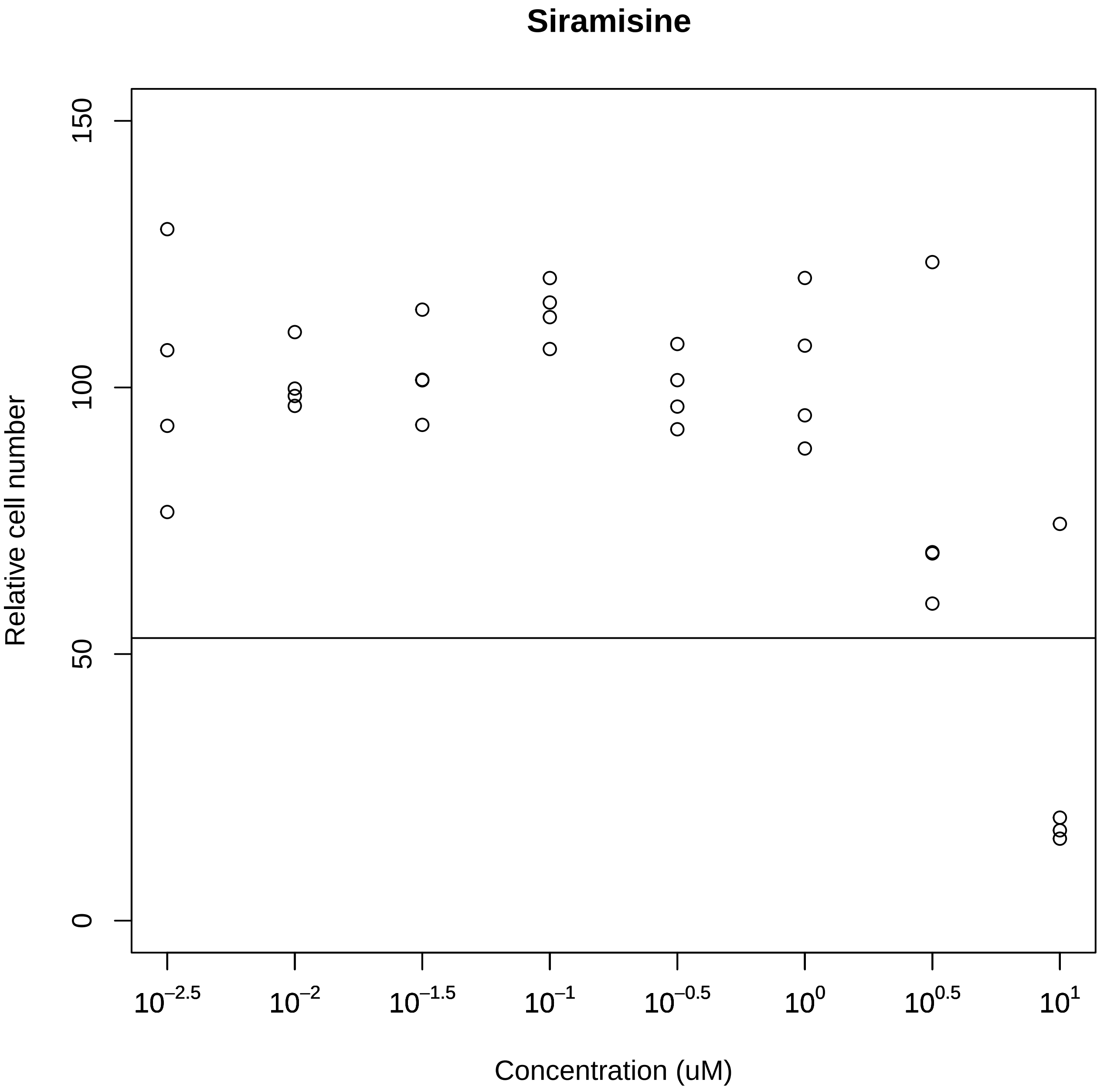

Siramisine
Relative cell number
150
100
50
0
$10^{-2.5}$
10^{-2}
$10^{-1.5}$
10^{-1}
$10^{-0.5}$
10^{0}
$10^{0.5}$
10^{1}
Concentration (uM)

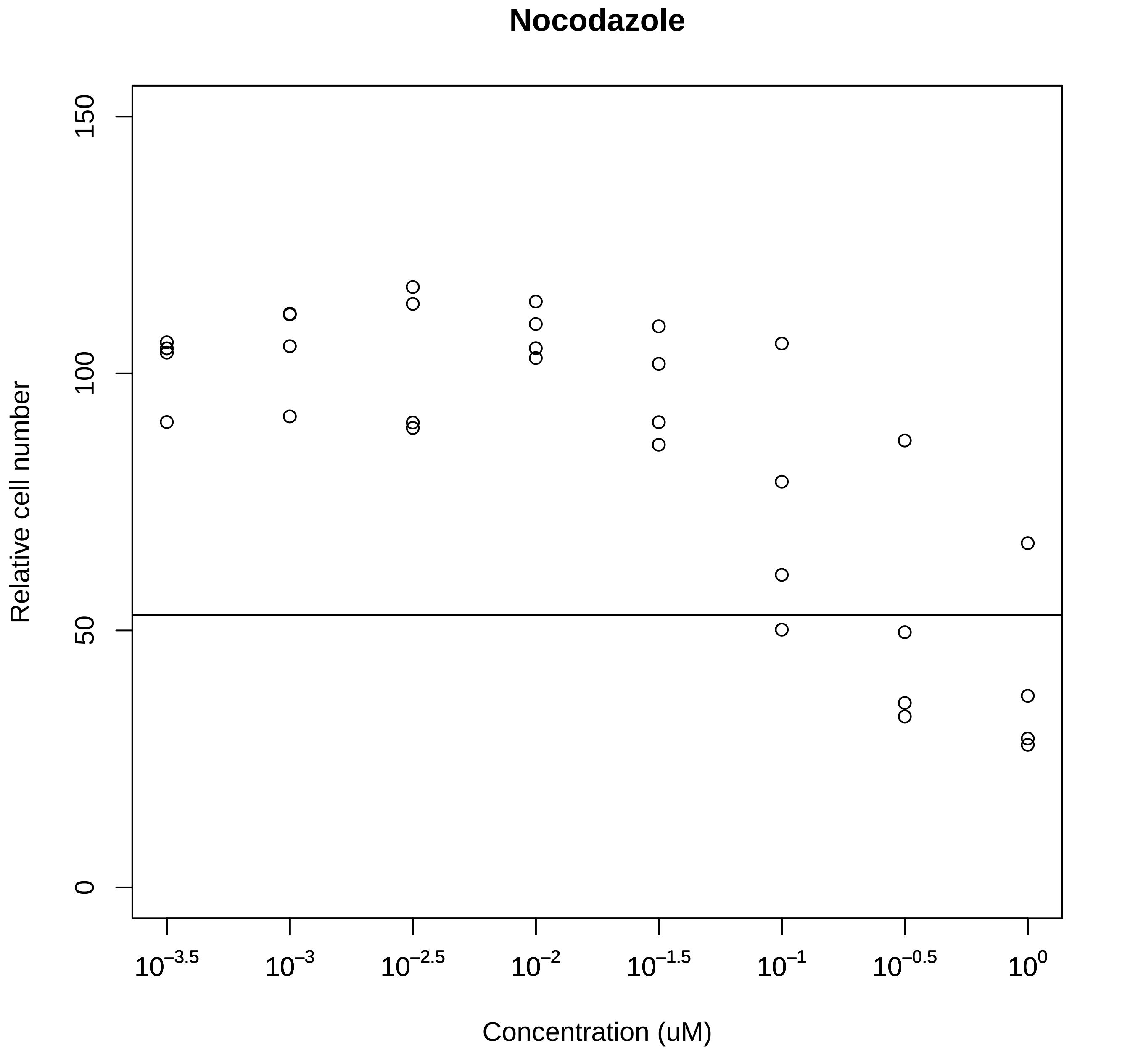

Nocodazole
Relative cell number
0
50
100
150
$10^{-3.5}$
10^{-3}
$10^{-2.5}$
10^{-2}
$10^{-1.5}$
10^{-1}
$10^{-0.5}$
10^{0}
Concentration (uM)

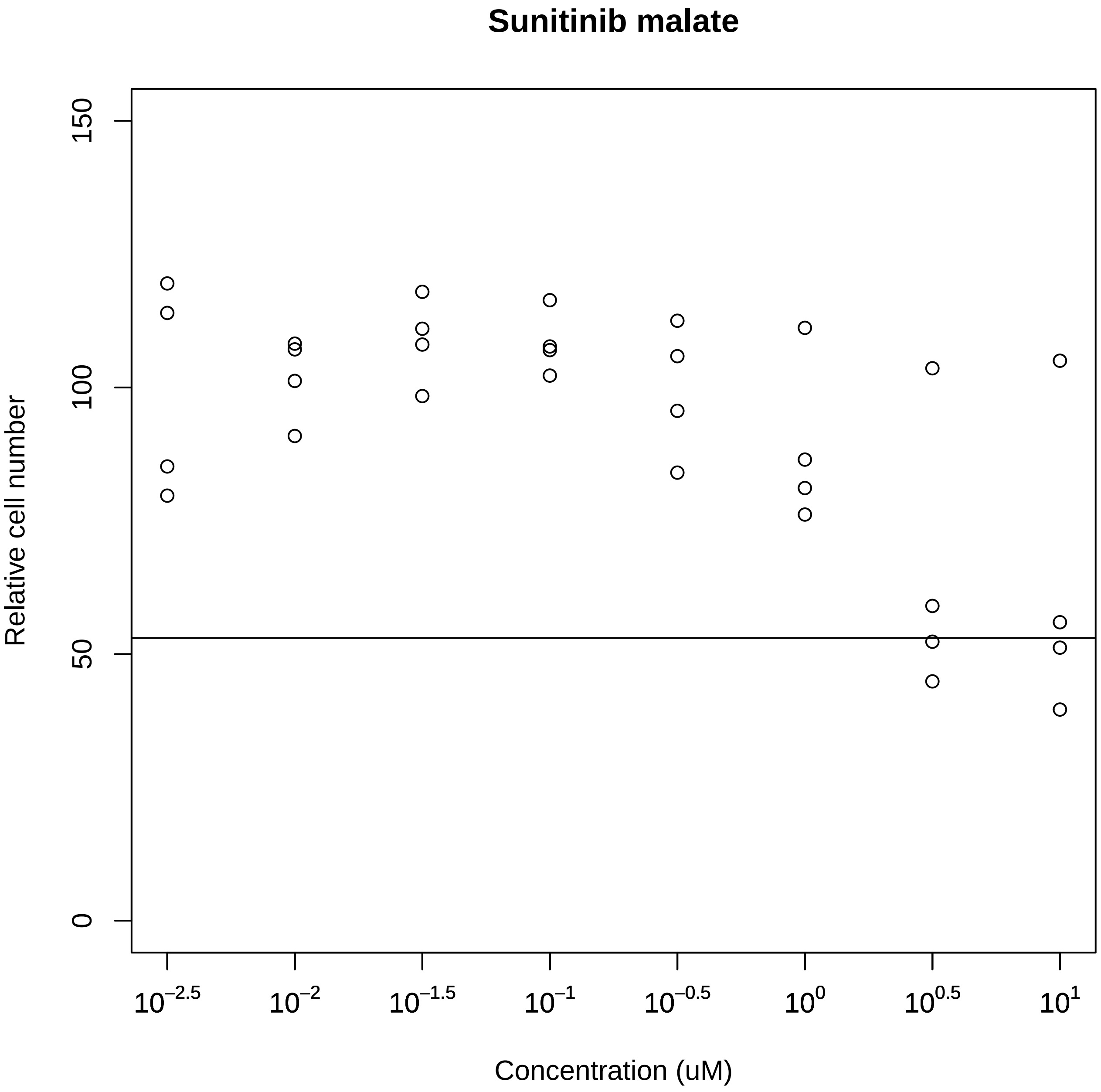

Sunitinib malate
Relative cell number
0
50
100
150
$10^{-2.5}$
10^{-2}
$10^{-1.5}$
10^{-1}
$10^{-0.5}$
10^{0}
$10^{0.5}$
10^{1}
Concentration (uM)

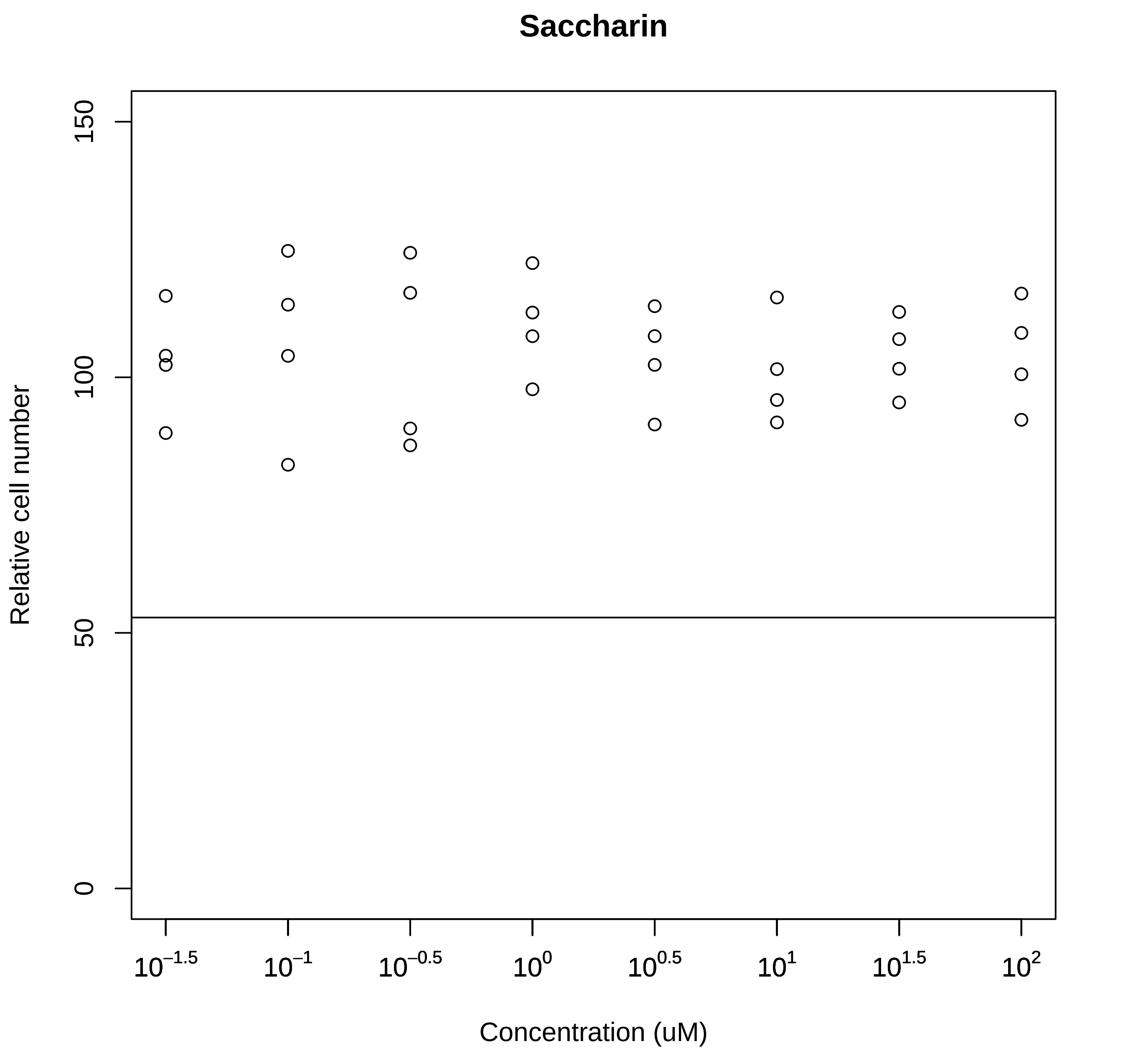
Saccharin
Relative cell number
150
100
50
0
$10^{-1.5}$
10^{-1}
$10^{-0.5}$
10^{0}
$10^{0.5}$
10^{1}
$10^{1.5}$
10^{2}
Concentration (uM)

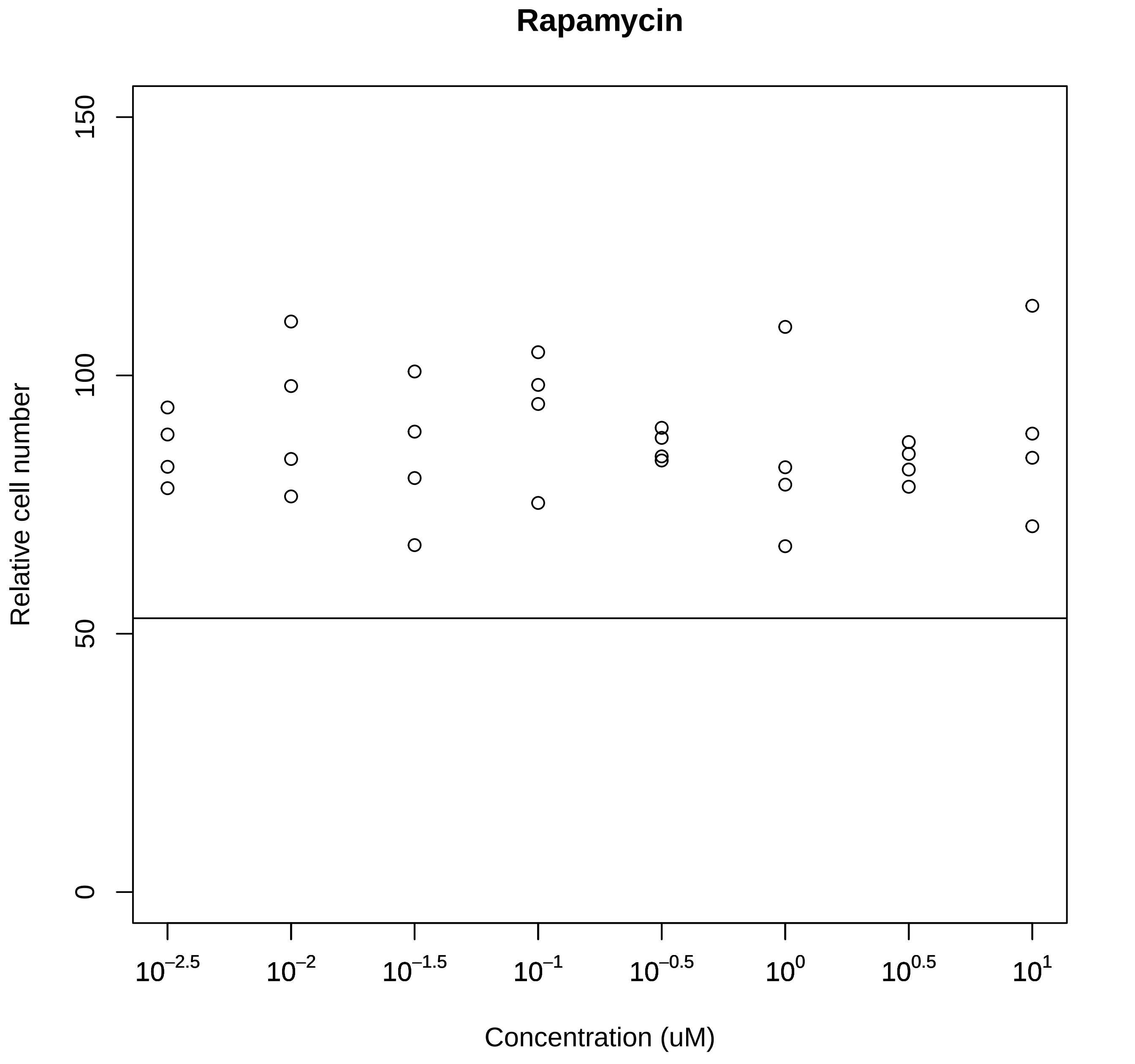

Rapamycin
Relative cell number
150
100
50
0
$10^{-2.5}$
10^{-2}
$10^{-1.5}$
10^{-1}
$10^{-0.5}$
10^{0}
$10^{0.5}$
10^{1}
Concentration (uM)

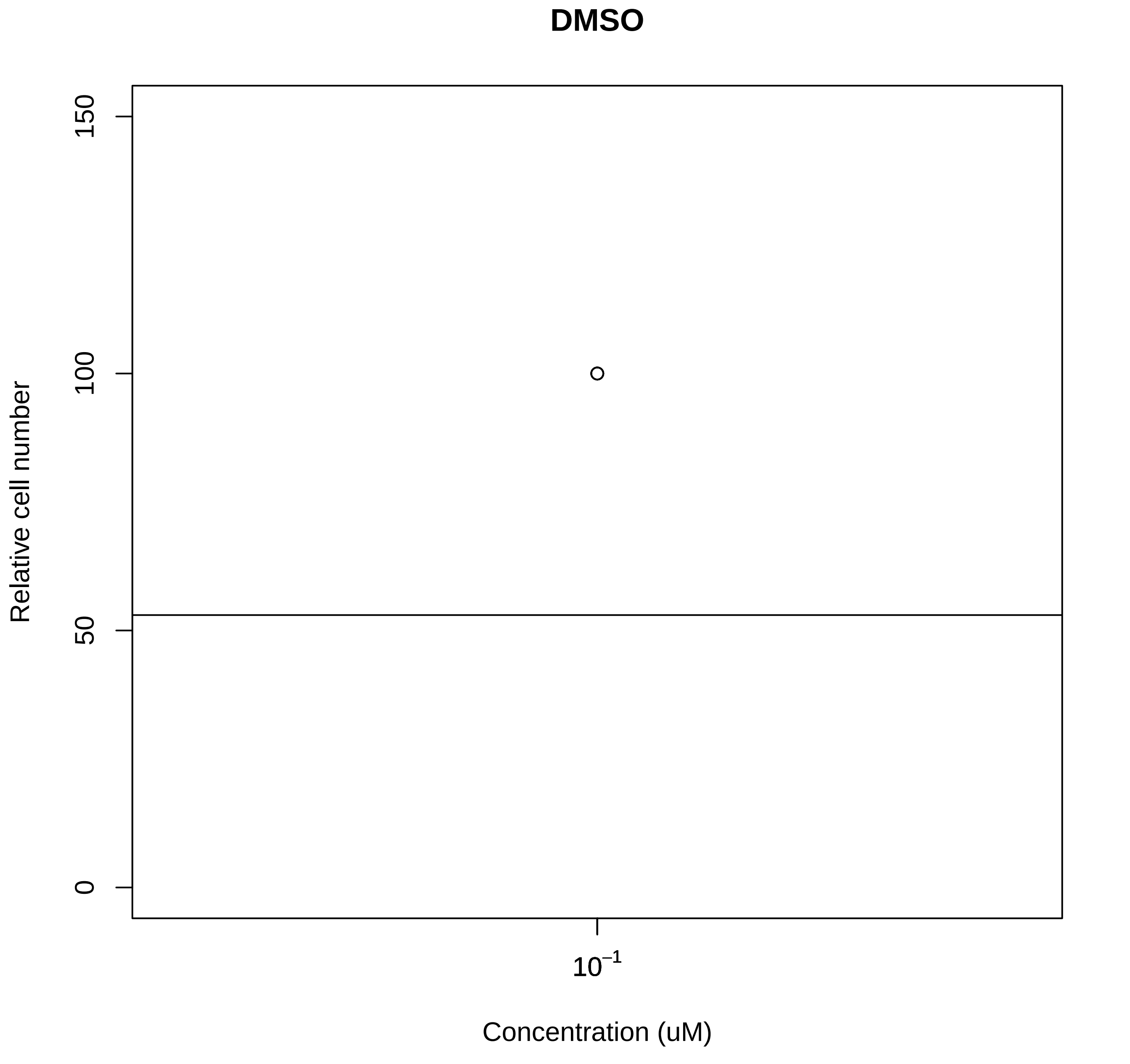

DMSO
150
100
50
0
Relative cell number
10^{-1}
Concentration (uM)
