# Supplementary material for: Cell Painting PLUS: expanding the multiplexing capacity of Cell Painting-based phenotypic profiling using iterative staining-elution cycles
Source: Nat Commun. 2025 Apr 24;16:3857. doi: 10.1038/s41467-025-58765-8 (PMC12022024; doi:10.1038/s41467-025-58765-8)
Supplement: Supplementary file 4 — Description of Additional Supplementary Files [file 41467_2025_58765_MOESM4_ESM.pdf]

SUPPLEMENTARY DATA

Supplementary Data 1. Excel file summarizing imaging setup, optimal imaging and elution conditions, and cost and time expenditure for the CPP and CP assay.

Supplementary Data 2. Excel file summarizing plate layout and concentrations of reference compounds.

Supplementary Data 3. Excel file summarizing CPP and CP features extracted from images using Harmony software for generation of compound activity profiles (feature-level).

Supplementary Data 4. Excel file summarizing relative sum of replicate-level differences of intra-/inter-plate variabilities for CPP and CP across all cell lines.

Supplementary Data 5. Excel file summarizing CPP and CP features extracted from images using Harmony or Cell Profiler software for BMC modeling, and their assignment to feature categories using KNIME software for BMC modeling.

Supplementary Data 6. Detailed overview of CPP image analysis sequence using Harmony software.

Supplementary Data 7. Detailed overview of CP image analysis sequence using Harmony software.

Supplementary Data 8. Relative cell numbers (PercentCells) of MCF7 cells captured at 20x magnification in CP or CPP and analyzed using Harmony or Cell Profiler image analysis software for all reference compounds.

Supplementary Data 9. Relative cell numbers (PercentCells) of HepG2 cells captured at 20x magnification in CPP and analyzed using Harmony image analysis software for all reference compounds.

Supplementary Data 10. Relative cell numbers (PercentCells) of U2OS cells captured at 20x magnification in CPP and analyzed using Harmony image analysis software for all reference compounds.

Supplementary Data 11. Relative cell numbers (PercentCells) of RPTEC cells captured at 20x magnification in CPP and analyzed using Harmony image analysis software for all reference compounds.

Supplementary Data 12. High-resolution versions of CPP compound activity profile heatmaps for MCF-7 cells captured at 20x magnification.

Supplementary Data 13. High-resolution versions of CPP compound activity profile heatmaps for MCF-7 cells captured at 40x magnification.

Supplementary Data 14. High-resolution versions of CP compound activity profile heatmaps for MCF-7 cells captured at 20x magnification.

Supplementary Data 15. High-resolution versions of CP compound activity profile heatmaps for MCF-7 cells captured at 40x magnification.

Supplementary Data 16. High-resolution versions of CPP compound activity profile heatmaps for HepG2 cells captured at 20x magnification.

Supplementary Data 17. High-resolution versions of CPP compound activity profile heatmaps for U2OS cells captured at 20x magnification.

Supplementary Data 18. High-resolution versions of CPP compound activity profile heatmaps for RPTEC cells captured at 20x magnification.

Supplementary Data 19. CP and CPP image analysis sequences for Harmony software.

Supplementary Data 20. CPP image analysis sequences for Cell Profiler software.

Supplementary Data 21. CP and CPP data analysis pipelines for KNIME software.

Supplementary Data 22. R scripts used to analyze and visualize CP and CPP data.

Supplementary Data 23. BMC bar plots comparing the total number of in-/active BMC features from the cytoplasm, ring region, and membrane region that are related to AGP (CP), Actin (CPP), or Golgi (CPP) channels in MCF-7 cells

captured at 20x magnification and analyzed using Harmony image analysis software for all reference compounds. The right plots show the same data but grouped for different concentration ranges.

Supplementary Data 24. BMC bar plots comparing the total number of in-/active BMC features from the cytoplasm that are related to RNA/ER (CP), RNA (CPP), or ER (CPP) channels in MCF-7 cells captured at 20x magnification and analyzed using Harmony image analysis software for all reference compounds. The right plots show the same data but grouped for different concentration ranges.
